# Copper‐Catalyzed Carbonylative Cyclization of CO_2_: A Promising Approach for Synthesis of Flavone

**DOI:** 10.1002/advs.202415795

**Published:** 2025-02-07

**Authors:** Zijun Huang, Junyong Dong, Pengtao Liu, Yadi Yin, Bing Yi, Zhengjun Fang, Xiaolin Jiang, Yuehui Li

**Affiliations:** ^1^ Hunan Province Key Laboratory of Environmental Catalysis and Waste Rechemistry, College of Chemistry and Chemical Engineering Hunan Institute of Engineering Xiangtan 411104 P. R. China; ^2^ School of Pharmacy Shanghai University of Medicine and Health Sciences Shanghai 201318 P. R. China; ^3^ College of Smart Energy Shanghai Jiao Tong University Shanghai 200240 P. R. China; ^4^ Carbon‐Negative Synthetic Biology for Biomaterial Production from CO2 (CNSB) Campus for Research Excellence and Technological Enterprise (CREATE) 1 CREATE Way Singapore 138602 Singapore

**Keywords:** Carbon dioxide utilization, Carbonylation, CO_2_ mass transfer, Copper catalyst, Flavone

## Abstract

Flavones are an important class of building blocks for numerous biologically active molecules, pharmaceuticals, and natural products. Reductive carbonylation of CO_2_ is a powerful method to provide high‐value heterocycles quickly. However, examples of transition metal‐catalyzed carbonylation to produce flavones using CO_2_ are quite scarce, and the related copper‐catalyzed carbonylative cyclization of CO_2_ is not reported. Here, a general procedure is developed for the copper‐catalyzed carbonylative C(sp^3^)‐H bond synthesis of flavone using CO_2_ as the C1 source. Additionally, ^13^C‐labeled flavones are successfully synthesized using [^13^C]‐CO_2_, demonstrating significant inhibitor activity against MCF‐7 cells in antitumor assays. Mechanistic investigations suggest that the phenolic group accelerates CO_2_ mass transfer by promoting nucleophilic addition to DBU‐CO_2_ complexes, followed by selective intramolecular carbonylative cyclization.

## Graphical abstract

The study reports the first copper‐catalyzed carbonylative C(sp^3^)─H bond synthesis of flavone using CO_2_ as the C1 source. Various aryl iodides and 2‐hydroxyacetophenones tolerated well and transformed into flavone with moderate to good yields. ^13^C‐labeled flavones are successfully synthesized using [^13^C]‐CO_2_, demonstrating significant inhibitor activity against MCF‐7 cells in antitumor assays.

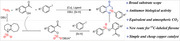

## 1 Introduction

**Scheme 1:**
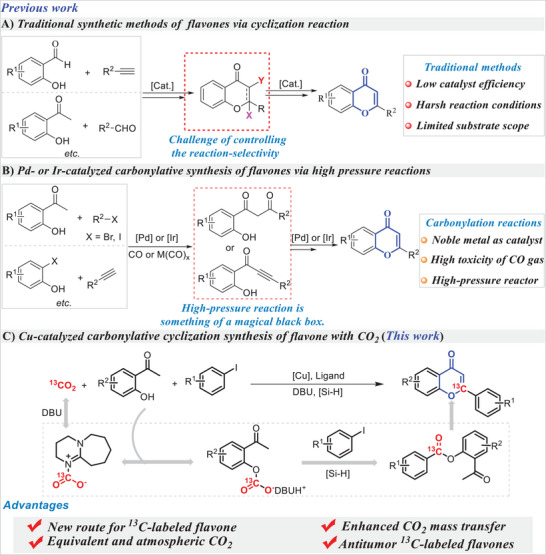
Background and the design.

Flavones are an important class of flavonoids occurring in heterocyclic scaffolds with a variety of medicines and bioactive natural molecules,^[^ ^1^, ^2^ ^]^ such as anti‐cancer, antioxidant, anti‐allergic, antibacterial, and anti‐inflammatory activities. The development of methodologies for designing and developing flavones, which allows for rapid diversification to give more functionalized flavones, is of great significance. A number of classical synthetic routes can be used to produce flavones, including the following: Baker‐Venkataraman reaction,^[^ ^3^, ^4^ ^]^ Claisen‐Schmidt reaction,^[^ ^5^ ^]^ Karl von‐ Auwers reaction,^[^ ^6^ ^]^ Allan‐Robinson reaction,^[^ ^7^, ^8^ ^]^ Algar‐Flynn‐Oyamada reaction,^[^ ^9^ ^]^ Kostanecki‐Robinson reaction,^[^ ^10^, ^11^ ^]^ etc.^[^ ^12^, ^13^, ^14^, ^15^, ^16^, ^17^, ^18^ ^]^ There are, however, drawbacks to these procedures, such as their low catalyst efficiency, limited substrate scope and harsh reaction conditions (**Scheme** **1**A). Thus, the development of novel and more efficient catalytic systems to address these issues still remains a great challenge.

For the synthesis of heterocyclic compounds, transition metal‐catalyzed carbonylation is one of the most effective and economical procedures.^[^ ^19^, ^20^, ^21^, ^22^, ^23^, ^24^, ^25^, ^26^, ^27^ ^]^ Palladium or iridium‐catalyzed carbonylative synthesis of flavone has been reported as it utilizes CO gas or metal carbonyl as carbonyl source (Scheme 1B).^[^ ^12^, ^28^, ^29^, ^30^, ^31^, ^32^, ^33^, ^34^, ^35^, ^36^, ^37^, ^38^, ^39^, ^40^, ^41^, ^42^, ^43^ ^]^ These simple but efficient carbonylation reactions were clearly demonstrated for the efficient and selective preparation of flavones. But the synthetic strategies are of limited application due to the use of toxic CO gas as C1 resource, noble metal as catalyst and high‐pressure reactors. Catalytic conversion of CO_2_, which is a low‐cost, abundant, nontoxic, and recyclable C1 feedstock, into valuable chemicals are of great value and attractive in synthesis.^[^ ^44^, ^45^, ^46^, ^47^, ^48^, ^49^ ^]^ Transition metal‐catalyzed carboxylation with CO_2_ has become a straightforward and sustainable method to prepare valuable heterocyclic scaffolds.^[^ ^50^, ^51^, ^52^ ^]^ However, it is still highly challenging and limited to produce complicated heterocyclic compounds. Particularly, developing efficient and economical methods for isotope‐labeled heterocyclic compounds with biological activities is difficult and challenging. To our knowledge, non‐noble metal catalyzed carbonylation with CO_2_ to give flavones has not been reported.

Since an intramolecular reaction is more efficient than an intermolecular one, we thought that carbonylative cyclization of CO_2_ could proceed much faster if it were carried out intramolecularly. Using nucleophilic characters of phenolic group, it is envisaged that combining the fast nucleophilic addition of phenolic compounds to silyl formate and the subsequent intramolecular instead of intermolecular carbonylative cyclization of C(sp^3^)‐H bonds could achieve this purpose. Motivated by this concept, we herein demonstrate a copper‐catalyzed carbonylative C(sp^3^)‐H bond synthesis of flavone using CO_2_ as the C1 source (Scheme 1C).

## 2 Results

### 2.1 Reaction Conditions Optimization

At the beginning of this study, iodobenzene **1a** and 2‐hydroxyacetophenone **2a** were chosen as model substrates by using CuCl and **L1** (Xantphos, **Table** **1**) as the catalyst system with 1,8‐diazabicyclo[5.4.0]‐7‐Undecene (DBU) as the base in dimethyl sulfoxide (DMSO) under CO_2_ atmosphere at 120 °C to evaluate the feasibility of the carbonylation reaction. We initially screened copper catalysts using Xantphos as the ligand (Table 1, entries 1–3, and Tables S1, Supporting Information). Experimental results show that CuCl with Xantphos showed better catalytic activity (85%). IPr·CuCl and IMes·CuCl was able to provide **4aa** in 77% and 59% yield (Table 1, entries 2 and 3). When monodentate ligands were used, no target product was detected (Table 1, entries 4 and 5). The use of Nixantphos (**L2**) and Sixantphos (**L3**) led to decreased yields for this transformation. Other ligands tested, including Xantphos‐type, bidentate phosphine and nitrogen‐based ligands, were all less effective (Table 1, entries 6–10 and Table S2, Supporting Information). Then, other organic bases and inorganic bases were added to the reaction, but no or little of the desired product **4aa** was observed (Table 1, entries 11–14). By altering the reaction temperature, a decreased yield was observed (Table S3, Supporting Information). Reducing the load of the DBU resulted in a significant decrease in yield. Moreover, when the amount of PMHS was decreased or increased, yields of 72% and 81% were achieved, respectively (Table S3, Supporting Information). We able to obtain a 64% yield of **4aa** under 10 mL CO_2_. No flavone product **4aa** was detected when PhSiH_3_, Et_3_SiH were used as the reductants. When the solvents toluene, THF, 1,4‐dioxane, and CH_3_CN were used, no flavone **4aa** was detected (Table S4, Supporting Information). Additionally, when the reaction was conducted without DBU or PMHS, the product **4aa** was not detected, indicating that DBU and PMHS played a significant role in the reaction (Table 1, entries 17 and 18). Preliminarily, CO_2_ has been identified as an important source of C1 (Table 1, entry 19).

**Table 1:** Optimization of reaction conditions.

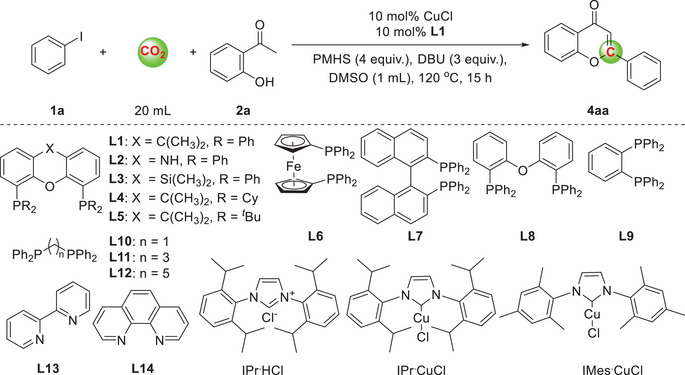					
Entry^a)^	[Cu]	Ligand	Base	Reductant	Yield [%]^b)^
1	CuCl	L1	DBU	PMHS	85 (81)^c)^
2	IPr^.^CuCl	**L1**	DBU	PMHS	77
3	IMes^.^CuCl	**L1**	DBU	PMHS	59
4	CuCl	PPh_3_	DBU	PMHS	N.D.
5	CuCl	PCy_3_	DBU	PMHS	N.D.
6	CuCl	**L2**‐**L5**	DBU	PMHS	0–51
7	CuCl	**L6**‐**L9**	DBU	PMHS	0–26
8	CuCl	**L10**‐**L12**	DBU	PMHS	trace
9	CuCl	**L13**‐**L14**	DBU	PMHS	N.D.
10	CuCl	IPr^.^HCl	DBU	PMHS	N.D.
11	CuCl	**L1**	NEt_3_	PMHS	N.D.
12	CuCl	**L1**	DABCO	PMHS	Trace
13	CuCl	**L1**	TBD	PMHS	47
14	CuCl	**L1**	K_2_CO_3_	PMHS	N.D.
15	CuCl	**L1**	DBU	PhSiH_3_	N.D.
16	CuCl	**L1**	DBU	Et_3_SiH	N.D.
17	CuCl	**L1**	−	PMHS	N.D.
18	CuCl	**L1**	DBU	−	N.D.
19 ^d)^	CuCl	**L1**	DBU	PMHS	N.D.

^a)^ Reaction conditions: iodobenzene **1a** (0.2 mmol), 2‐hydroxyacetophenone **2a** (0.24 mmol, 1.2 equiv.), [Cu] (10 mol%), ligand (10 mol%), base (0.6 mmol, 3 equiv.), reductant (Si‐H 4 equiv.), DMSO (1 mL), CO_2_ (20 mL), 120 °C, 20 h;

^b)^ Determined by GC using *n*‐tetradecane as internal standard;

^c)^ Isolated yield;

^d)^ N_2_ atmosphere. N.D. = Not Detected. See also Figure S1 (Supporting Information).

### 2.2 Substrate Scope of Iodobenzenes

**Scheme 2:**
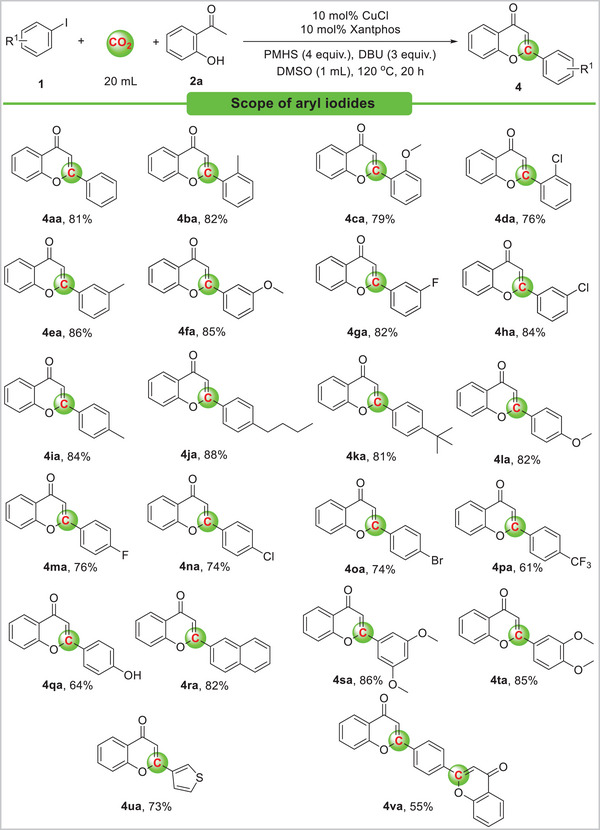
Carbonylation of aryl iodides and 2‐hydroxyacetophenone with CO_2_. Reaction conditions: aryl iodides **1** (0.2 mmol), 2‐hydroxyacetophenone **2a** (0.24 mmol,1.2 equiv.), CuCl (10 mol%), Xantphos (10 mol%), DBU (0.6 mmol, 3 equiv.), PMHS (Si‐H 4 equiv.), DMSO (1 mL), CO_2_ (20 mL), 120 °C, 20 h. Yields are those of isolated products.

Under optimal reaction conditions, we investigated the substrate scope of aryl iodides substituted with electron‐donating and electron‐withdrawing groups with CO_2_ and PMHS (**Scheme** **2**). With the tested aryl iodides, moderate to good yields of the corresponding flavones **4** were obtained. The corresponding flavones were obtained in good yields (**4ba**–**4da**) from aryl iodides substituted with electron‐donating and electron‐withdrawing groups. Moreover, *meta*‐substituted aryl iodides could also be successfully applied to this protocol (**1e**–**1h**). A variety of functional groups substituted in aryl iodides, such as methyl, methoxy, fluoro, and chloro, gave the corresponding products **4ea**–**4ha** in good yields under optimized reaction conditions. Furthermore, *para*‐substituted aryl iodides can also undergo carbonylation smoothly into their corresponding products (**4ia**–**4pa**). Due to electron‐withdrawing groups, 2‐(4‐(trifluoromethyl)phenyl)‐*4H*‐chromen‐4‐one **4pa** was obtained in a lower yield. Furthermore, when 4‐iodophenol **1q** was used as the reaction substrate, the corresponding flavone compound was obtained with a yield of 64%. However, when using 4‐aminophenyl iodide under standard conditions, the reaction failed to generate the desired product. 2‐hydroxyacetophenone **2a** can be reacted with 2‐iodonaphthalene **1r** to yield the product **4ra** in good yield. In addition, electron‐donating substituents (**1s**, **1t**) were tolerated, giving the corresponding products in good yields. 3‐iodothiophene **1u** was tested as well, and 73% yield of the flavone **4ua** was obtained. To our delight, upon using 1,4‐diiodobenzene **1v** as the reaction substrate, the reaction furnished the corresponding flavone product **4va** in 55% yield. Nevertheless, when bromobenzene or chlorobenzene was employed as reaction substrate, the corresponding flavone compounds could not be generated.

### 2.3 Substrate Scope of the Substituted 2‐Hydroxyacetophenone

**Scheme 3:**
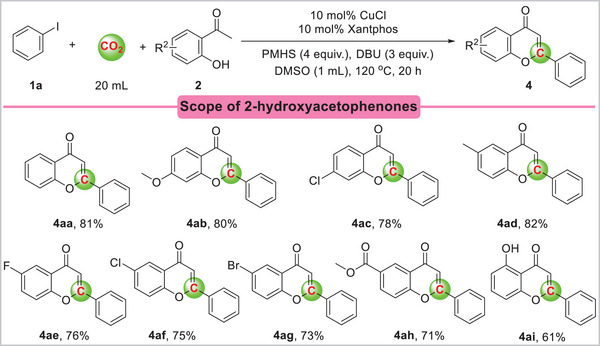
Carbonylation of iodobenzene and the substituted 2‐hydroxyacetophenone with CO_2_. Reaction conditions: iodobenzene **1a** (0.2 mmol), substituted 2‐hydroxyacetophenone **2** (0.24 mmol,1.2 equiv.), CuCl (10 mol%), Xantphos (10 mol%), DBU (0.6 mmol, 3 equiv.), PMHS (Si‐H 4 equiv.), DMSO (1 mL), CO_2_ (20 mL), 120 °C, 20 h. Yields are those of isolated products.

Various substituted 2‐hydroxyacetophenones were evaluated to further explore the substrate scope of this copper‐catalyzed carbonylation. 2‐hydroxyacetophenone containing different functional groups showed good reactivity, giving the corresponding flavones in good yields (**Scheme** **3**). In addition, various functional groups, including methoxy, chloro, methyl, fluoro, bromo, and methyl formate, could be successfully applied to this protocol, resulting in efficient synthesis of the corresponding products (**4ab**‐**4ah**) in 71–82% yields. Compared to 2‐ hydroxyacetophenones with electron‐donating groups, 2‐hydroxyacetophenones with electron‐withdrawing groups gave lower flavone yields. When 1‐(2,6‐dihydroxyphenyl)ethan‐1‐one **2i** was used as the reaction substrate, the yield decreased and the flavone compound was obtained with a yield of 61%.

**Scheme 4:**
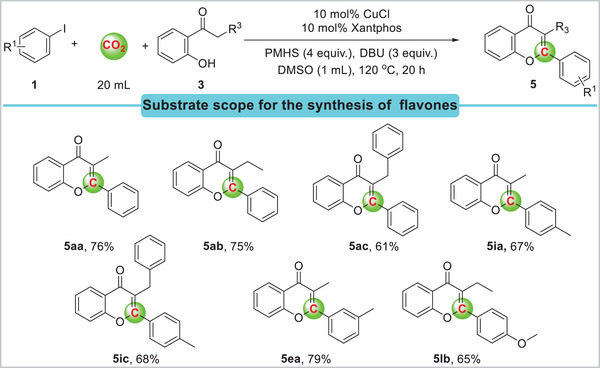
Carbonylation of iodobenzenes and 2‐hydroxyacetophenone derivatives with CO_2_. Reaction conditions: aryl iodides **1** (0.2 mmol), 2‐hydroxyacetophenone derivatives **3** (0.24 mmol,1.2 equiv.), CuCl (10 mol%), Xantphos (10 mol%), DBU (0.6 mmol, 3 equiv.), PMHS (Si‐H 4 equiv.), DMSO (1 mL), CO_2_ (20 mL), 120 °C, 20 h. Yields are those of isolated products.

Furthermore, several kinds of 2‐hydroxyacetophenone derivatives were tolerated under optimized reaction conditions and gave good yields (**Scheme** **4**). 2‐Hydroxypropiophenone **3a** and 2‐hydroxybutyrophenone **3b** were used in carbonylation reaction, and products **5aa** and **5ab** were produced in 76% and 75% yields, respectively. 2‐Hydroxy‐3‐phenylpropiophenone **3c** was well tolerated, giving the corresponding flavones **5ac** and **5ic** in 61% and 68% isolated yield, respectively. In addition, 2‐hydroxypropiophenone **3a** and 3‐iodotoluene **1e** were tested, yielding 79% of the desired product **5ea**.

### 2.4 Synthetic Applications

**Scheme 5:**
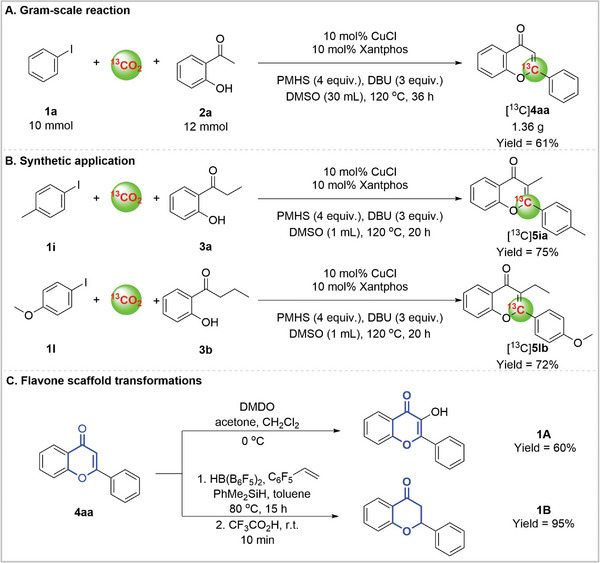
Synthetic applications.

To further explore the utilities of copper‐catalyzed carboxylation, we applied it in the synthesis of ^13^C‐labeled flavone using [^13^C]CO_2_ (**Scheme** **5**). A gram‐scale (10 mmol) experiment was successfully performed to provide 2‐phenyl‐*4H*‐chromen‐4‐one‐2‐^13^ *C* ([^13^C] **4aa**) with a 61% isolated yield by employing CuCl and Xantphos. The gram‐scale reaction could successfully proceed without the significant erosion in the yield, which indicated the high efficiency of copper‐catalyzed carboxylation of iodobenzene and 2′‐hydroxyacetophenone using [^13^C]CO_2_. Biologically active ^13^C‐labeled 3‐methyl‐2‐(p‐tolyl)‐*4H*‐chromen‐4‐one‐2‐^13^ *C* ([^13^C]**5ia**) and 3‐ethyl‐2‐(4‐methoxyphenyl)‐*4H*‐chromen‐4‐one‐2‐^13^ *C* ([^13^C]**5lb**) were produced in good yield via carboxylation of aryl iodides (**1i** and **1l**) in one pot, respectively. These examples not only illustrate that the source of the carbon in the ^13^C‐labeled flavone, but also give an effective tool to introduce carbon labeling in the flavone backbones for the preparation of pharmaceuticals. The applicability of this protocol was showcased by the synthetic transformation of 2‐phenyl‐4H‐chromen‐4‐one **4aa** into 3‐hydroxy‐2‐phenyl‐*4H*‐chromen‐4‐one **1A** and 2‐phenylchroman‐4‐one **1B**, respectively. Moderate to excellent yields were obtained.

### 2.5 Biologically Active Interest of ^13^C‐Labeled Flavones

**Figure 1:**
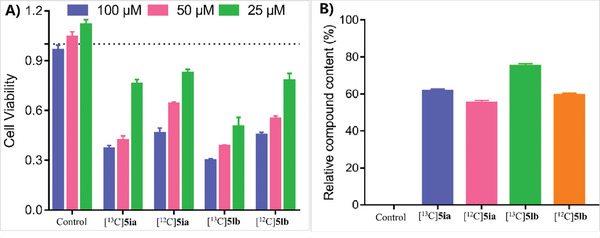
Biologically active interest of ^13^C‐labeled flavones. A) Cell viability of MCF‐7 after various treatments (*n* = 3) by three corresponding products at different concentration. B) Intracellular uptake of two products by MCF‐7 cells at 12h after various treatments by 50 µm [^13^C]**5ia** and [^13^C]**5lb**, respectively (*n* = 3).

There are a number of compounds that use flavone scaffolds in order to elicit different pharmacological properties at different targets, with different substitution patterns.^[^ ^1^, ^2^ ^]^ Pharmaceutical development frequently uses C‐13 instead of tritium due to its high metabolic stability.^[^ ^53^, ^54^ ^]^ The substituent groups at the C3 position of the flavone core, such as methyl and ethyl, can alter the compound's lipophilicity, solubility and intermolecular forces, thereby affecting their biological activity.^[^ ^1^ ^]^ We tested the antitumor bio‐activity of two C3‐substituted compounds, [^13^C]**5ia** and [^13^C]**5lb**, and both exhibit good inhibitory activity against MCF‐7 (**Figure** **1**A). Further findings indicate that the two ^13^C‐labeled flavones, compared to the [^12^C] flavones, show a significant increase in intracellular concentration after 12 h of incubation (Figure 1B).

### 2.6 Mechanistic Studies

**Scheme 6:**
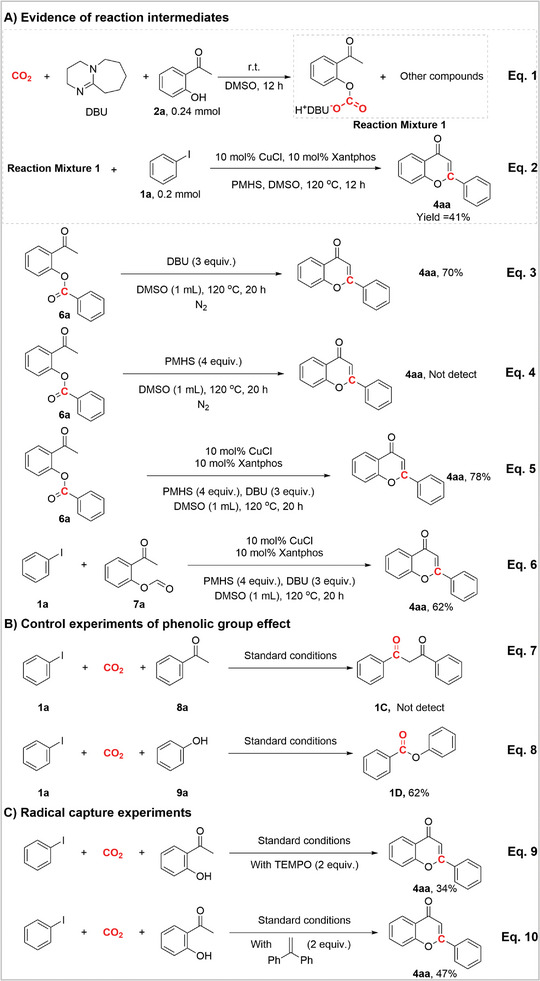
Preliminary Mechanistic Studies. A) Evidence of reaction intermediates; B) Control experiments of phenolic group effect; C) Radical capture experiment.

A series of mechanistic experiments were conducted to better understand the mechanism (**Scheme** **6**). Using CO_2_ and 2‐hydroxyacetophenone **2a** in the presence of DBU, in situ‐formed reaction mixture **1** was obtained. Then, the carbonylation reaction with CuCl and Xantphos as catalyst, the flavone product **4aa** was obtained with a 41% yield (S2 Equations 1 and 2, Scheme 6). By using 2‐acetylphenyl benzoate **6a** as the substrate and DBU as the base, flavone 4aa could be produced with 70% yield (Equation 3, Scheme 6). However, when PMHS was used without DBU, no target product was detected (S4 Equation 4, Scheme 6). Under standard reaction conditions, the reaction system can yield the flavone product with 78% yield (Equation 5, Scheme 6). The results suggest that 2‐acetylphenyl benzoate **6a** might be the key intermediate in the copper‐catalyzed carboxylation reaction. In addition, when iodobenzene **1a** and 2‐acetylphenyl formate **7a** react under standard conditions, the flavone product can be obtained with a yield of 62% (Equation 6, Scheme 6). With iodobenzene **1a** and acetophenone **8a** as substrates, 1,3‐diphenylpropane‐1,3‐dione **1C** was not detected in the presence of CO_2_ (Equation 7, Scheme 6). Then, the iodobenzene **1a** reacted with phenol **9a** under standard conditions, giving phenyl benzoate **1D** in 62% yield (Equation 8, Scheme 6). These findings reveal that the phenolic group played significant roles in the carbonylation. The carboxylation reaction proceeded smoothly with several radical trapping reagents, such as TEMPO (2,2,6,6‐tetramethylpiperidinooxy)and 1,1‐diphenylethylene. Based on these results, radical intermediates may not be involved in carboxylation (Equations 9 and 10, Scheme 6).

**Figure 2:**
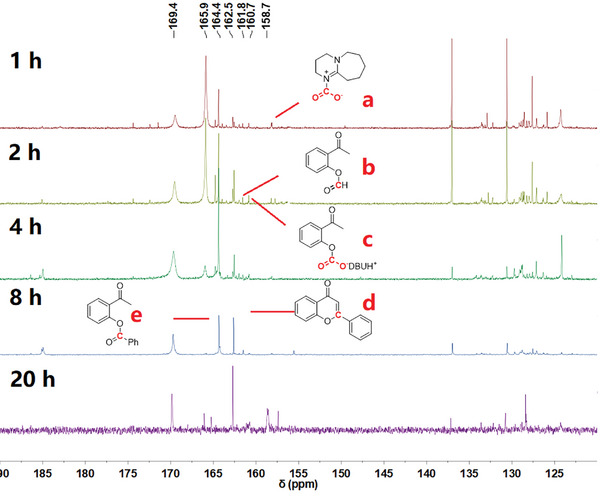
^13^C NMR spectra of the reactions. Reaction conditions: CuCl (0.04 mmol), Xantphos (0.04 mmol), iodobenzene **1a** (0.4 mmol), 2‐hydroxyacetophenone **2a** (0.5 mmol), PMHS (Si‐H: 2.0 mmol), [D_6_]DMSO (2.0 mL), ^13^CO_2_, 120 °C.

To further understand the reaction mechanism, in situ ^13^C NMR experiments were performed using [^13^C] CO_2_ with CuCl and Xantphos at different times on the reaction between iodobenzene **1a** and 2‐hydroxyacetophenone **2a** in [D_6_]DMSO (**Figure** **2**; details of the NMR experiments and full spectra are described in the Supporting Information). According to the ^13^C NMR spectrum, additional signals appeared at 158.7, 160.7, 162.5, 164.4, 165.9, and 169.4 ppm, which may be assigned to DBU‐CO_2_ intermediates (**a**), 2‐acetylphenyl formate (**b**), *O*‐acetophenone carbamates intermediates (**c**), 2‐phenyl‐*4H*‐chromen‐4‐one **4aa** (**d**), 2‐acetylphenyl benzoate (**e**), silyl formate species and copper formate species, respectively (Figure 2).^[^ ^55^, ^56^, ^57^, ^58^ ^]^ 2‐acetylphenyl formate **7a** was prepared and tested. Without CO_2_, it was converted to the desired product in 62% GC yield. The formation of **7a** as the intermediate via reduction of intermediate **D** cannot be excluded. However, the signal of **7a** (at 160.7 ppm) is very weak during the in situ NMR experiment. It is well‐known that aryl formats can act as CO surrogate in the presence of transition metals. However, the formation of CO and the subsequent carbonylation might be not responsible in this work due to the very low concentration of **7a**. Furthermore, CO was not detected by GC analysis for the gas phase of the reaction mixture. As the in situ reaction time extended, the ^13^C NMR signal of 2‐phenyl‐*4H*‐chromen‐4‐one‐2‐^13^ *C* ([^13^C]**4aa**) grew increasingly intense, demonstrating the formation of the ^13^C‐labeled 2‐phenyl‐*4H*‐chromen‐4‐one‐2‐^13^ *C*. Based on control experiments and in situ NMR experimental results, phenolic group plays a key role in ensuring high efficiency and selectivity for copper catalyzed carbonylation.

**Figure 3:**
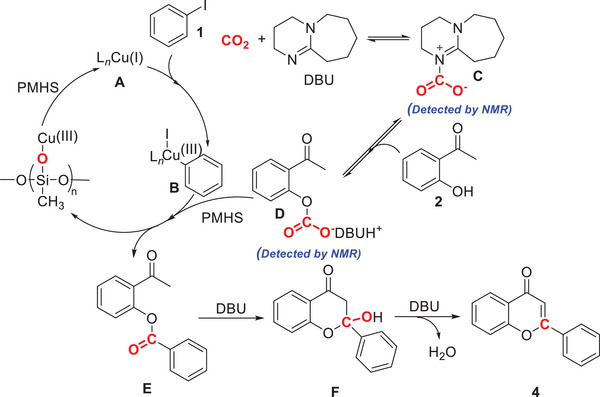
Proposed reaction mechanism.

Based on experimental results and previous reports,^[^ ^28^, ^29^, ^30^, ^31^, ^32^, ^33^, ^34^, ^35^, ^36^, ^37^, ^38^, ^39^, ^40^, ^41^, ^42^, ^43^, ^46^, ^55^, ^56^, ^57^, ^58^ ^]^ a proposed mechanism is shown in **Figure** **3**. First, active [LCu(I)] complex **A** is generated from the CuCl and Xantphos. Following the oxidative addition of [LCu(I)] species to aryl iodides, aryl copper species **B** are formed. 2‐hydroxyacetophenone **2** react with DBU‐CO_2_ intermediate **C** from CO_2_ and DBU to produce *O*‐aryl carbamates intermediates **D**. Further transformation yields intermediate **E** via the reaction of Aryl copper species **B** and aryl carbamates intermediates **D** in the presence of PMHS, whereby copper siliconate intermediate is released and further reduced by PMHS to regenerate [LCu(I)] complex **A**. Finally, intermediate **E** undergoes an intramolecular aldol condensation to yield intermediate **F**. Intermediate **F** is then dehydrated to give flavone **4** in the presence of DBU.

## 3 Conclusion

In summary, we have developed a promising approach, which uses a phenolic group to facilitate the carbonylative cyclization of CO_2_ for efficient and highly selective synthesis of flavone. It was found that various aryl iodides and 2‐hydroxyacetophenones tolerated well and transformed into flavone with moderate to good yields. By using easily available^13^C‐labeled CO_2_ as the starting materials, diversified ^13^C‐labeled flavone derivatives were prepared in moderate yields. Finally, a possible reaction pathway involving CO_2_ and PMHS is proposed.

## 4 Experimental Section

### General Procedure for Condition Optimization

Under nitrogen atmosphere, [Cu] (10 mol%, 0.02 mmol), ligand (10 mol%, 0.02 mmol), iodobenzene **1a** (0.2 mmol), 2‐hydroxyacetophenone **2a** (0.24 mmol), base, solvent (1 mL), silane and a stirring bar were added into a 10 mL oven‐dried sealed glass tube in glovebox (as shown in Figure S1, Supporting Information). After being sealed, the glass tube was brought out of the glovebox, and CO_2_ was injected by syringe. Then the mixture was stirred for 20 h in a pre‐heated‐to‐120 °C alloyed block. After the reaction was completed, the tube was cooled to room temperature, and the pressure was carefully released. The residual silane was quenched with HCl (2 m). The mixture was extracted with ethyl acetate. The combined organic layers were washed with brine and dried over anhydrous Na_2_SO_4_. The yield of were measured by GC analysis using *n*‐tetradecane as the internal standard.

Full experimental details and characterization of new compounds can be found in the Supplementary Information.

## Conflict of Interest

The authors declare no conflict of interest.

## Supporting information



Supporting Information

## Data Availability

The data that support the findings of this study are available in the supplementary material of this article.
