# Supplementary material for: Copper‐Catalyzed Carbonylative Cyclization of CO2: A Promising Approach for Synthesis of Flavone
Source: Adv Sci (Weinh). 2025 Feb 7;12(13):2415795. doi: 10.1002/advs.202415795 (PMC11967808; doi:10.1002/advs.202415795)
Supplement: Supplementary file 1 — Supporting Information [file ADVS-12-2415795-s001.pdf]

ADVANCED SCIENCE
Open Access

Supporting Information

for *Adv. Sci.*, DOI 10.1002/advs.202415795

Copper-Catalyzed Carbonylative Cyclization of CO_2: A Promising Approach for Synthesis of Flavone

Zijun Huang, Junyong Dong, Pengtao Liu, Yadi Yin, Bing Yi, Zhengjun Fang, Xiaolin Jiang* and Yuehui Li**

Supporting Information

Copper-catalyzed carbonylative cyclization of CO_2: A promising approach for synthesis of flavone

Zijun Huang,,[a] Junyong Dong,[a] Pengtao Liu,[a] Yadi Yin,[a] Bing Yi,[a] Zhengjun Fang,[a] Xiaolin Jiang,*,[b] and Yuehui Li*,[c]*

[a] Hunan Province Key Laboratory of Environmental Catalysis and Waste Rechemistry, College of Chemistry and Chemical Engineering, Hunan Institute of Engineering, 411104 Xiangtan, P.R. China

[b] School of Pharmacy, Shanghai University of Medicine and Health Sciences, Shanghai 201318, P.R. China

[c] College of Smart Energy, Shanghai Jiao Tong University, 200240, Shanghai, P.R. China

[d] Carbon-Negative Synthetic Biology for Biomaterial Production from CO_2 (CNSB), Campus for Research Excellence and Technological Enterprise (CREATE), 1 CREATE Way, Singapore, 138602, Singapore

E-mail: huangzijun@hnie.edu.cn
E-mail: spujxl@163.com
E-mail: liyuehui@sjtu.edu.cn

Contents

1. General information.. 3

2. Optimization of the reaction conditions...................... 4

3. Procedures for applications... 7

4. Mechanism study 10

5. Characterization data.. 21

6. References 37

7. Copies of spectra 39

1. General information

Unless otherwise stated, all the materials were purchased from commercial suppliers and were used as received. PMHS (poly(methylhydrosiloxane), H%:1.5-1.55%) was from Adamas. CO_2 (99.995%) was purchased from Messer (Wujiang, China). $[^{13}C]CO_2$ (99 atom% ^{13}C) was purchased from WUHAN NEWRADAR SPECIAL GAS Co., LTD. Solvents were dried by solvent purification system from LC Technology Solution Inc. Flash chromatography was performed on silica gel. The products were characterized by ^1H NMR, ^{13}C NMR, and HRMS spectroscopy. All ^1H and ^{13}C NMR spectra were recorded on Bruker 400 MHz spectrometer. The NMR chemical shift values refer to $CDCl_3$ (δ (^1H), 7.26 ppm; δ (^{13}C), 77.16 ppm); $(CD_3)_2SO$ (δ (^1H), 2.50 ppm; δ (^{13}C), 39.52 ppm). Data are reported as follows: chemical shift, multiplicity (s = singlet, d = doublet, t = triplet, q = quartet, p = pentet, s = sextet, h = heptet, m = multiplet, br = broad), coupling constants (Hz) and integration. GC-MS data were obtained SHIMADZU GCMS-QP 2010 Plus, GC data were obtained SHIMADZU GC-2030, HRMS data were obtained on Agilent 6530 spectrometer. All measurements were carried out at room temperature unless otherwise stated.

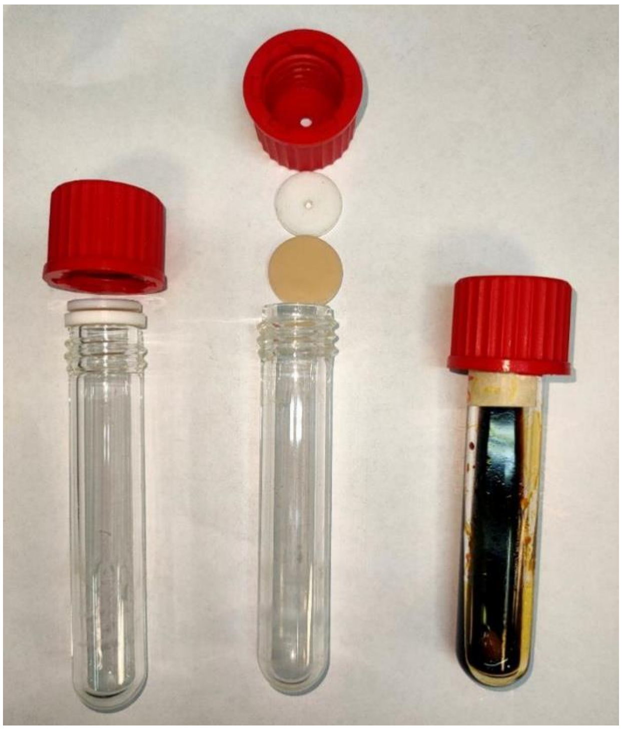

Figure S1. The sealed glass tube used in reactions of iodobenzene and 2-hydroxyacetophenone with CO_2.

2. Optimization of the reaction conditions

General Procedure: Under nitrogen atmosphere, [Cu] (10 mol%, 0.02 mmol), ligand (10 mol%, 0.02 mmol), iodobenzene **1a** (0.2 mmol), 2-hydroxyacetophenone **2a** (0.24 mmol), base, solvent (1 mL), silane and a stirring bar were added into a 10 mL oven-dried sealed glass tube in glovebox (as shown in **Figure S1**). After sealed, the glass tube was brought out of the glovebox and CO_2 was injected by syringe. Then the mixture was stirred for 20 hours in a pre-heated-to-120 °C alloyed block. After the reaction was completed, the glass tube was cooled to room temperature and released the gas carefully. Then, *n*-tetradecane was added into the vial. Yield was determined by GC using *n*-tetradecane as an internal standard.

Table S1**.** Optimization of copper source.[a]

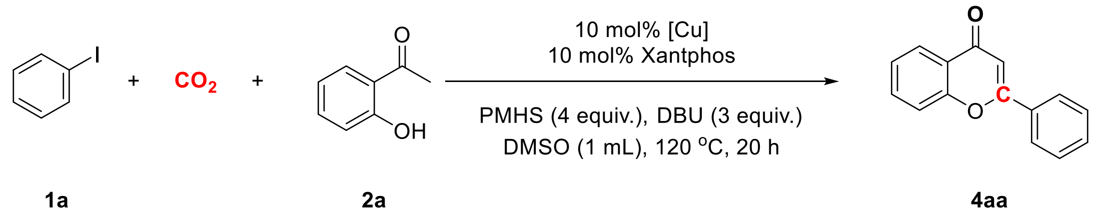

Entry	[Cu] source	Yield (%)
**1**	**CuCl**	**85**
2	IPr·CuCl	77
3	IMes·CuCl	59
4	CuBr	52
5	CuI	18
6	CuOAc	27
7	$CuCl_2$	68
8	$CuBr_2$	43
9	$Cu(acac)_2$	36
10	$Cu(OTf)_2$	45
11	$CuSO_4$	14

[a] Reaction conditions: **1a** (0.2 mmol), **2a** (0.24 mmol,1.2 equiv.), [Cu] (10 mol%), Xantphos (10 mol%), DBU (0.6 mmol, 3 equiv.), PMHS (Si-H 4 equiv.), DMSO (1 mL), CO_2 (20 mL), 120 °C, 20 h. Yield determined by GC using *n*-tetradecane as the internal standard. PMHS = Poly(methylhydrosiloxane). DBU = 1,8-Diazabicyclo [5.4.0] undec-7-ene. DMSO = Dimethyl sulfoxide. acac = acetylacetonate. OTf = trifluoromethanesulfonate.

Table S2. Optimization of ligands.[a]

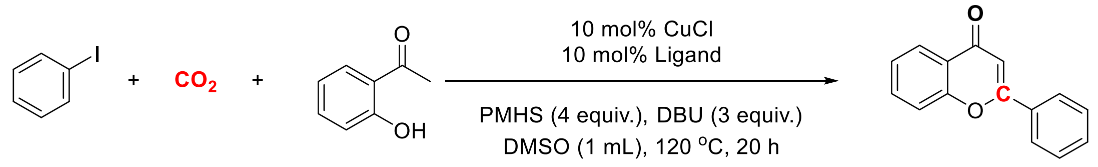

1a **2a** **4aa**

Entry	Ligand	Yield (%)
**1**	**L1**	**85**
2	**L2**	51
3	**L3**	43
4	**L4**	N.D.
5	**L5**	N.D.
6	**L6**	12
7	**L7**	26
8	**L8**	trace
9	**L9**	N.D.
10	**L10**	trace
11	**L11**	trace
12	**L12**	trace
13	**L13**	N.D.
14	**L14**	N.D.
15	PPh_3	N.D.
16	PCy_3	N.D.
17	IPr·HCl	N.D.

[a] Reaction conditions: **1a** (0.2 mmol), **2a** (0.24 mmol,1.2 equiv.), CuCl (10 mol%), Ligand (10 mol%), DBU (0.6 mmol, 3 equiv.), PMHS (Si-H 4 equiv.), DMSO (1 mL), CO_2 (20 mL), 120 °C, 20 h. Yield determined by GC using *n*-tetradecane as the internal standard. N.D. = Not detected.

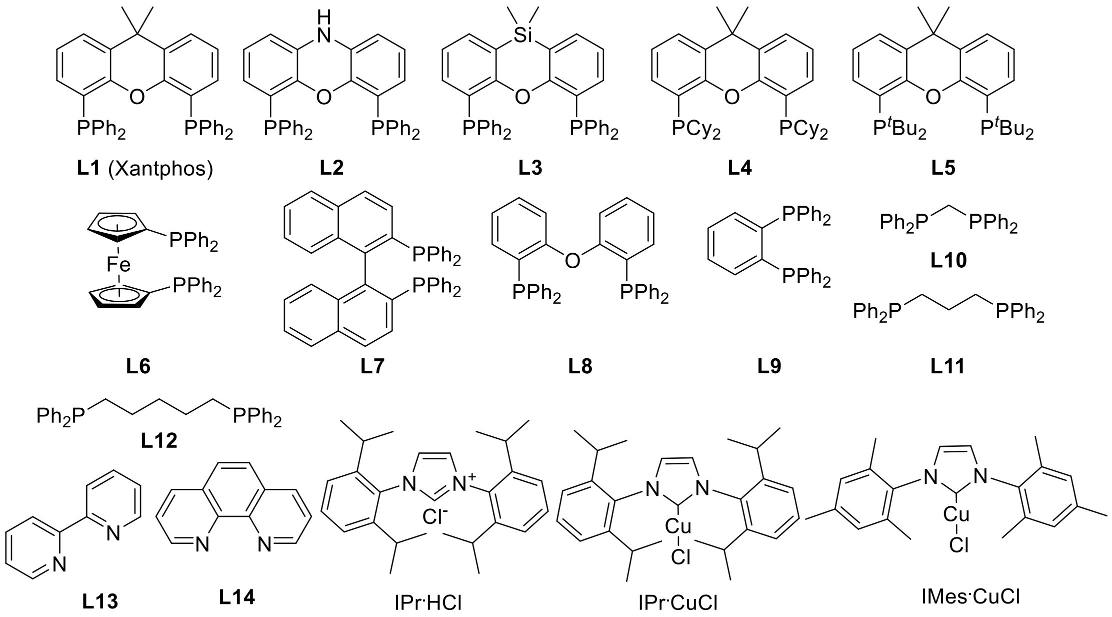

Table S3. Optimization of other factors.

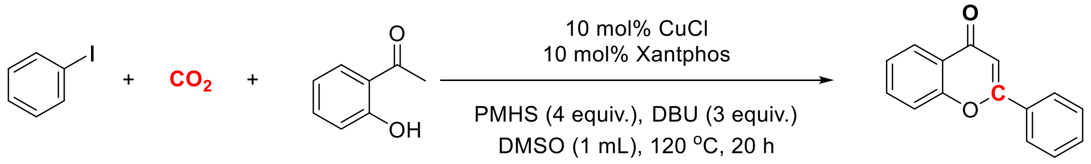

1a 2a 4aa

Entry	Deviation from standard conditions	Yield (%)
**1**	**None**	**85**
2	2 equiv. DBU instead of 3 equiv. DBU	65
3	3 equiv. PMHS instead of 4 equiv. PMHS	72
4	6 equiv. PMHS instead of 4 equiv. PMHS	81
5	140 °C instead of 120 °C	83
6	100 °C instead of 120 °C	31
7	10 bar CO_2 instead of 20 mL CO_2	56
8	10 mL CO_2 instead of 20 mL CO_2	64

[a] Reaction conditions: **1a** (0.2 mmol), **2a** (0.24 mmol,1.2 equiv.), CuCl (10 mol%), Xantphos (10 mol%), DBU (0.6 mmol, 3 equiv.), PMHS (Si-H 4 equiv.), solvents (1 mL), CO_2 (20 mL), 120 °C, 20 h. Yield determined by GC using *n*-tetradecane as the internal standard.

Table S4**.** Optimization of solvents. [a]

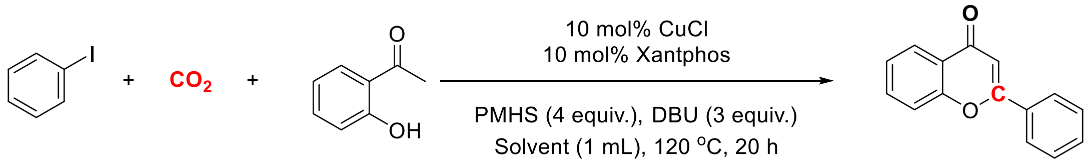

1a 2a 4aa

Entry	solvent	Yield (%)
**1**	**DMSO**	**85**
2	DMF	11
3	NMP	trace
4	Toluene	N.D.
5	THF	N.D.
6	1,4-dioxane	N.D.
7	CH_3CN	N.D.

[a] Reaction conditions: **1a** (0.2 mmol), **2a** (0.24 mmol,1.2 equiv.), CuCl (10 mol%), Xantphos (10 mol%), DBU (0.6 mmol, 3 equiv.), PMHS (Si-H 4 equiv.), solvents (1 mL), CO_2 (20 mL), 120 °C, 20 h. Yield determined by GC using *n*-tetradecane as the internal standard. DMF = *N*,*N*-Dimethylformamide. NMP = *N*-Methyl-2-pyrrolidone. THF = Tetrahydrofuran.

3. Procedures for applications

3.1 Gram-scale reaction

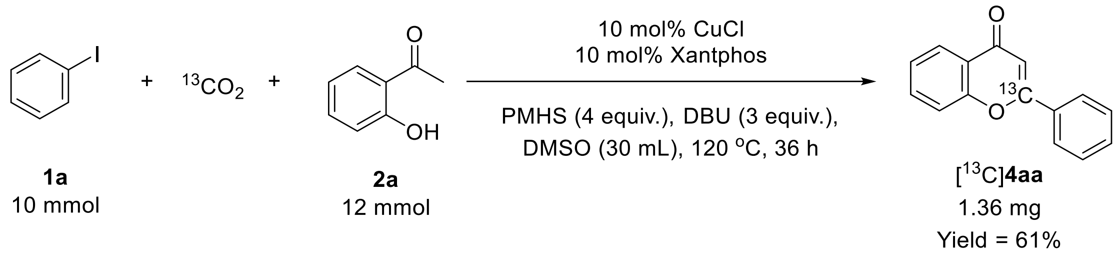

General Procedure: Under nitrogen atmosphere, the **1a** (10 mmol), **2a** (12 mmol), CuCl (10 mol%, 1 mmol), Xantphos (10 mol%, 1 mmol), DBU (30 mmol), DMSO (30 mL), PMHS (Si-H: 40 mmol) and a stirring bar were added into a vial (250 mL) in glovebox. After sealed, the vial was brought out of the glovebox under nitrogen atmosphere. The vial was placed in an alloyed plate, which was then transferred into an autoclave (250 mL) under nitrogen flow. Then, the autoclave was pressurized with $^{13}CO_2$ gas to 3 bars. The reaction was performed at 120 °C for 36 h. After the reaction finished, the autoclave was cooled to room temperature and the pressure was carefully released. The residual silane was quenched with HCl (2 M) and extracted with ethyl acetate. The combined organic layers were washed with brine and dried over anhydrous Na_2SO_4 and concentrated in vacuo. The residue was purified by flash chromatography on silica gel (eluting with petroleum ether /ethyl acetate = 20/1 to 5/1) to give the product [^{13}C]**4aa** (white solid, 61% yield).

3.2 Synthetic application

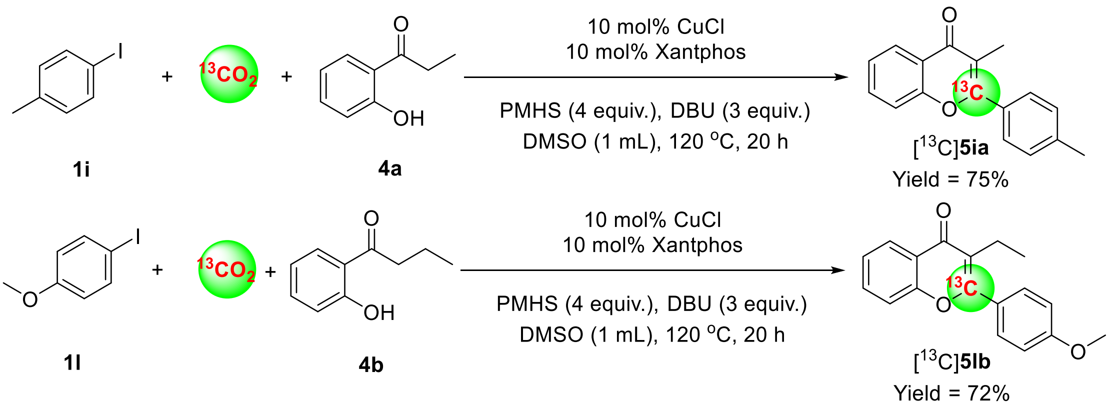

General Procedure:

Under nitrogen atmosphere, CuCl (10 mol%, 0.02 mmol), Xantphos (10 mol%, 0.02 mmol), **1** (0.2 mmol), **4** (0.24 mmol), DBU (0.6 mmol), DMSO (1 mL), PMHS (Si-H 4 equiv.) and a stirring bar were added into a 10 mL oven-dried sealed glass tube in glovebox (as shown in **Figure S1**). After sealed, the glass tube was brought out of the glovebox and [^{13}C]CO_2 was injected by syringe. Then the mixture was stirred for 20 hours in a pre-heated-to-120 °C alloyed block. After the reaction was completed, the glass tube was cooled to room temperature and the pressure was carefully released. The residual silane was quenched with HCl (2 M). The mixture was extracted with ethyl acetate. The combined organic layers were washed with brine and dried over anhydrous Na_2SO_4. The residue was purified by flash chromatography on silica gel (eluting with petroleum ether /ethyl acetate = 20/1 to 5/1) to give the product [^{13}C]**5**.

3.3 Flavone scaffold transformations

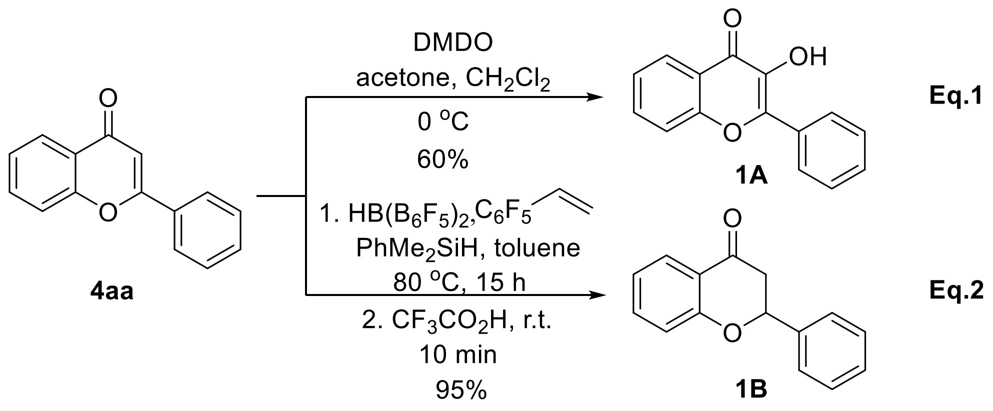

General experimental procedure for 3-hydroxy-2-phenyl-*4H*-chromen-4-one (Eq.1): Under nitrogen atmosphere, 2-phenyl-*4H*-chromen-4-one (**4aa**, 2.0 mmol) and a stirring bar were added into a 100 mL oven-driedglass bottle. CH_2Cl_2 (20 mL) and dimethyldioxirane (DMDO) / acetone was added until the substrate disappeared. The mixture was stirred at a temperature of 0 °C throughout the night and subsequently concentrated under vacuum conditions. The residue was purified by flash chromatography on silica gel (eluting with petroleum ether /ethyl acetate = 10/1 to 5/1) to give the product.

General experimental procedure for 2-phenylchroman-4-one (Eq.2): Under nitrogen atmosphere, $HB(C_6F_5)_2$ (0.01 mmol), 2,3,4,5,6-pentafluorostyrene (0.01 mmol), and dry toluene (0.10 mL) were added in a nitrogen atmosphere glovebox. The resulting mixture was stirred for 5 min at room temperature to afford a catalyst solution (0.10 M). To a sealing tube (10 mL), catalyst solution (0.0004 mmol, 4 μL, 0.1 M), $PhMe_2SiH$ (0.5 mmol), 2-phenyl-*4H*-chromen-4-one (**4aa,** 0.4 mmol), and dry toluene (0.8 mL) were added. The reaction mixture was stirred at 80 °C for 15 h, and then was cooled to room temperature followed by addition of trifluoroacetic acid (0.5 mmol). The resulting mixture stirred at room temperature for 10 min. The mixture was extracted with ethyl acetate. The combined organic layers were washed with brine and dried over anhydrous Na_2SO_4. The residue was purified by flash chromatography on silica gel (eluting with petroleum ether /ethyl acetate = 100/1-50/1) to give the product.

3.4 Biologically active interest of ^{13}C-labeled flavones

Cell culture

Human breast cancer cells (MCF-7) were provided by the Institute of Basic Medical Sciences, Chinese Academy of Medical Sciences (Beijing, China). Cells were cultured using DMEM supplemented with 10% fetal bovine serum in a 37°C incubator containing 5% CO_2. Cells were passaged when they grew to 80%-90% of the culture dish. All cells were grown to the third generation and then experimented.

Cytotoxicity assay

MCF-7 cells (4×10^3 cells/well) were seeded in 96-well plates and incubated for 24 h. After incubation, the cells were treated with DMEM containing different compounds for 48 h. The cells were incubated with 100 μL of 10% CCK-8/DMEM solution instead of the medium and protected from light for 1 h. After incubation, the absorbance of the cells was measured by using an enzyme meter at 450 nm excitation wavelength.

HPLC-MS analysis

MCF-7 cells (8×10^4 cells/well) were inoculated in six-well plates and incubated for 24 h. The original medium was replaced with fresh DMEM medium containing equal volume of PBS or test compound, respectively. The cells were collected by incubation for 12 h. The supernatant was removed and washed twice in PBS, and then added with cell lysate, and then centrifuged to extract the supernatan for HPLC-MS detection.

4. Mechanism study

4.1 Control experiments

Experimental procedure:

Under nitrogen atmosphere, the CuCl (0.02 mmol), Xantphos (0.02 mmol), PMHS (Si-H: 1.0 mmol), DBU (1.2 mmol), DMSO-*d*$_6$ (1 mL), 2'-hydroxyacetophenone (0.4 mmol) and a stirring bar were added to a 10 mL oven-dried sealed glass tube in glovebox. After sealed, the glass tube was brought out of the glovebox and CO_2 (20 mL) was injected by syringe. Then the mixture was stirred for 6 hours in a pre-heated-to-120 °C alloyed block. After the reaction was completed, the tube was cooled to room temperature and the pressure was carefully released. After the reaction finished, the tube was cooled to room temperature and the pressure was carefully released. Under nitrogen atmosphere, the **1a** (0.2 mmol), CuCl (0.02 mol), Xantphos (0.02 mol), PMHS (Si-H: 1.0 mmol) were added successively. The reaction mixture was stirred for another 12 h in a pre-heated-to-120 °C alloyed block. After the reaction was completed, the glass tube was cooled to room temperature and released the gas carefully. Then, *n*-tetradecane was added to the vial. Yield was determined by GC using *n*-tetradecane as an internal standard.

4.2 *In situ* NMR study for ^{13}C NMR spectra of the carbonylation reactions

1)

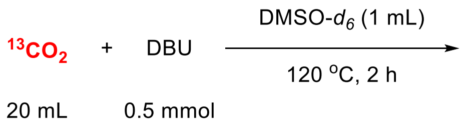

Procedure of NMR experiments:

Under nitrogen atmosphere, DBU (0.5 mmol), DMSO-d_6 (1 mL) and a stirring bar were added to a 10 mL oven-dried sealed glass tube in glovebox. After sealed, the glass tube was brought out of the glovebox and [^{13}C]CO_2 (20 mL) was injected by syringe. Then the mixture was stirred for 2 hours in a pre-heated-to-120 °C alloyed block. After the reaction was completed, the glass tube was cooled to room temperature and the pressure was carefully released. The reaction mixture was transferred into the NMR tube. At Bruker 400 MHz, then the ^{13}C NMR spectra were collected.

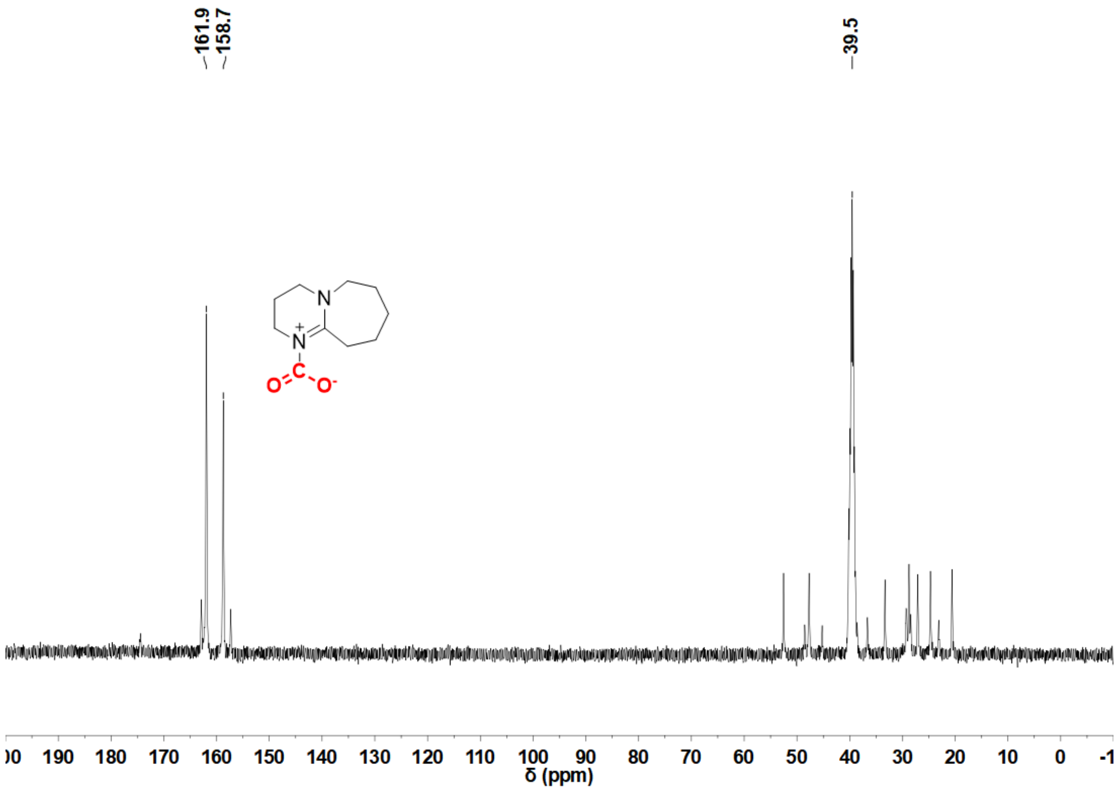

2)

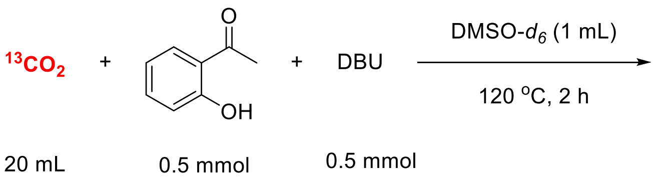

Under nitrogen atmosphere, 2-hydroxyacetophenone (0.5 mmol), DBU (0.5 mmol), DMSO-*d*6 (1 mL) and a stirring bar were added to a 10 mL oven-dried sealed glass tube in glovebox. After sealed, the glass tube was brought out of the glovebox and [^{13}C]CO_2 (20 mL) was injected by syringe. Then the mixture was stirred for 2 hours in a pre-heated-to-120 °C alloyed block. After the reaction was completed, the glass tube was cooled to room temperature and the pressure was carefully released. The reaction mixture was transferred into the NMR tube. At Bruker 400 MHz, then the ^{13}C NMR spectra were collected.

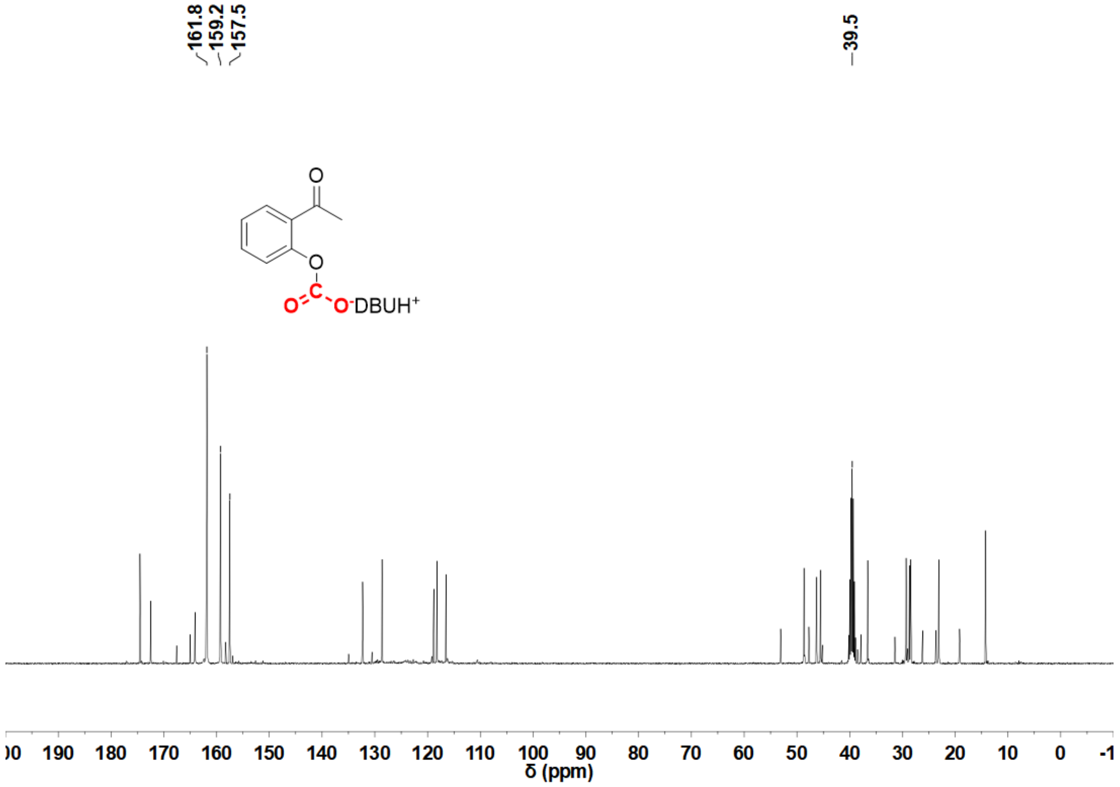

3)

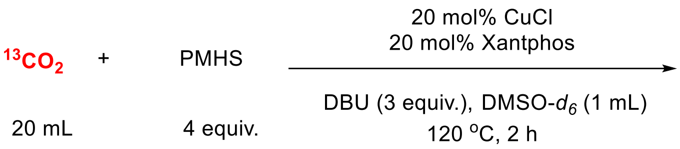

Procedure of NMR experiments:

Under nitrogen atmosphere, CuCl (0.04 mmol), Xantphos (0.04 mmol), PMHS (Si-H:

2.0 mmol), DBU (1.2 mmol), DMSO-d_6 (1 mL) and a stirring bar were added to a 10 mL oven-dried sealed glass tube in glovebox. After sealed, the glass tube was brought out of the glovebox and [^{13}C]CO_2 (20 mL) was injected by syringe. Then the mixture was stirred for 2 hours in a pre-heated-to-120 °C alloyed block. After the reaction was completed, the glass tube was cooled to room temperature and the pressure was carefully released. The reaction mixture was transferred into the NMR tube. At Bruker 400 MHz, then the ^{13}C NMR spectra were collected.

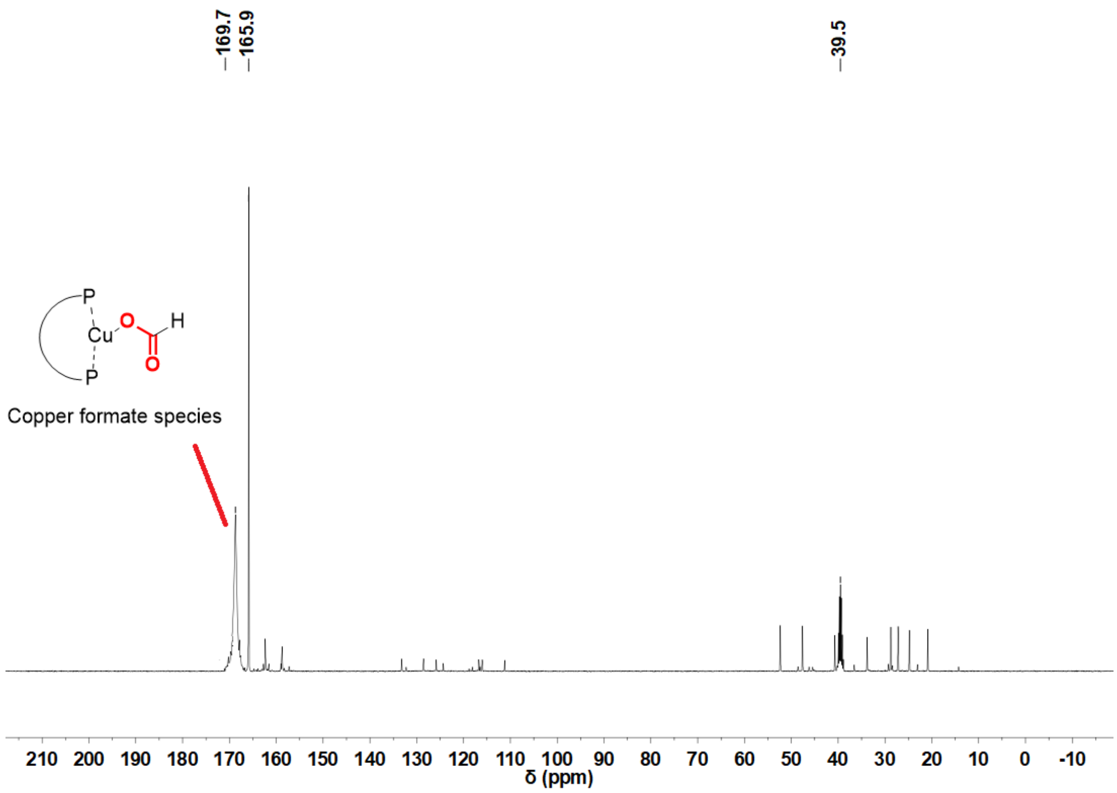

Figure S2. *In situ* ^{13}C NMR spectruma

4)

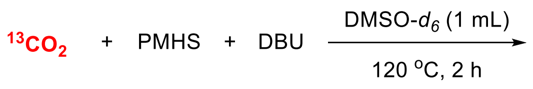

Procedure of NMR experiments:

Under nitrogen atmosphere, PMHS (Si-H: 2.0 mmol), DBU (1.2 mmol), DMSO-d_6 (1 mL) and a stirring bar were added to a 10 mL oven-dried sealed glass tube in glovebox. After sealed, the glass tube was brought out of the glovebox and [^{13}C]CO_2 (20 mL) was injected by syringe. Then the mixture was stirred for 2 hours in a pre-heated-to-120 °C

alloyed block. After the reaction was completed, the glass tube was cooled to room temperature and the pressure was carefully released. The reaction mixture was transferred into the NMR tube. At Bruker 400 MHz, then the ^{13}C NMR spectra were collected.

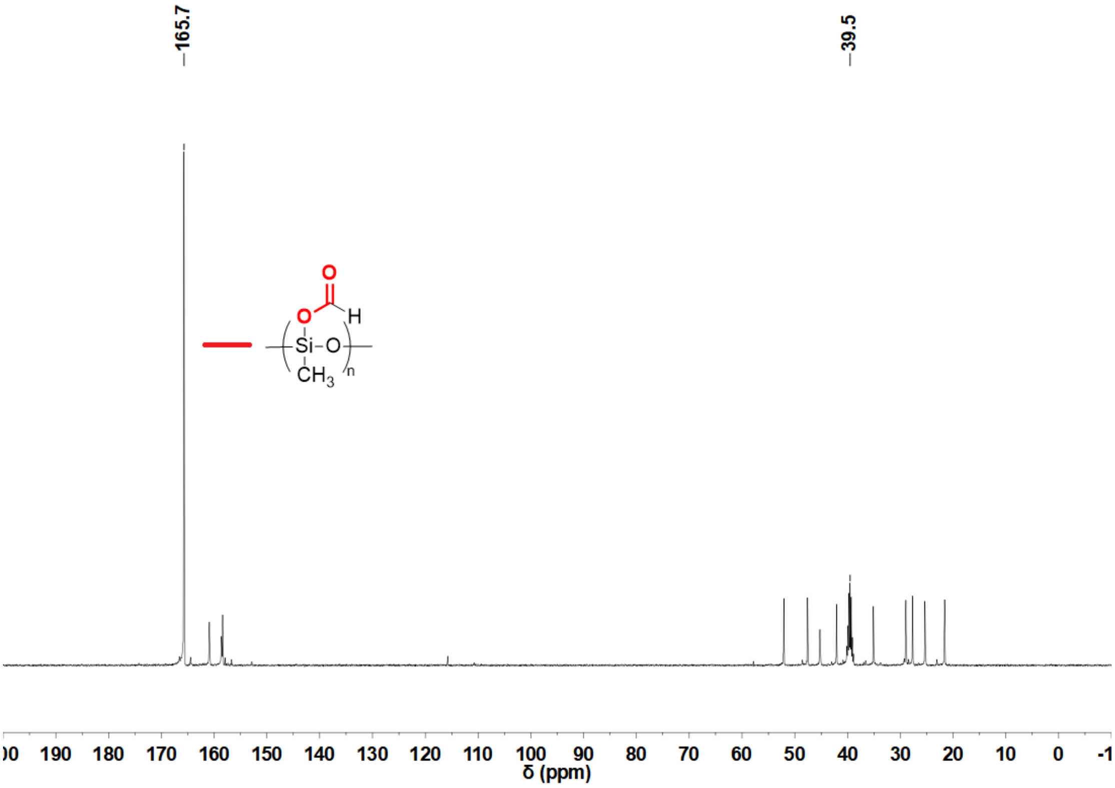

Figure S3. *In situ* ^{13}C NMR spectruma

5)

In situ NMR study for ^{13}C NMR spectra of the Copper-catalyzed carbonylative cyclization of CO_2

Procedure of NMR experiments:

Under nitrogen atmosphere, CuCl (0.04 mmol), Xantphos (0.04 mmol), PMHS (Si-H: 2.0 mmol), DBU (1.2 mmol), DMSO-d_6 (2 mL), iodobenzene (0.4 mmol), 2-hydroxyacetophenone (0.5 mmol) and a stirring bar were added to a 10 mL oven-dried sealed glass tube in glovebox. After sealed, the glass tube was brought out of the glovebox and [^{13}C]CO_2 (20 mL) was injected by syringe. Then the mixture was stirred for1、2、4、8、20 hours in a pre-heated-to-120 °C alloyed block. After the reaction was completed, the glass tube was cooled to room temperature and the pressure was

carefully released. The reaction mixture was transferred into the NMR tube. At Bruker 400 MHz, then the ^{13}C NMR spectra were collected.

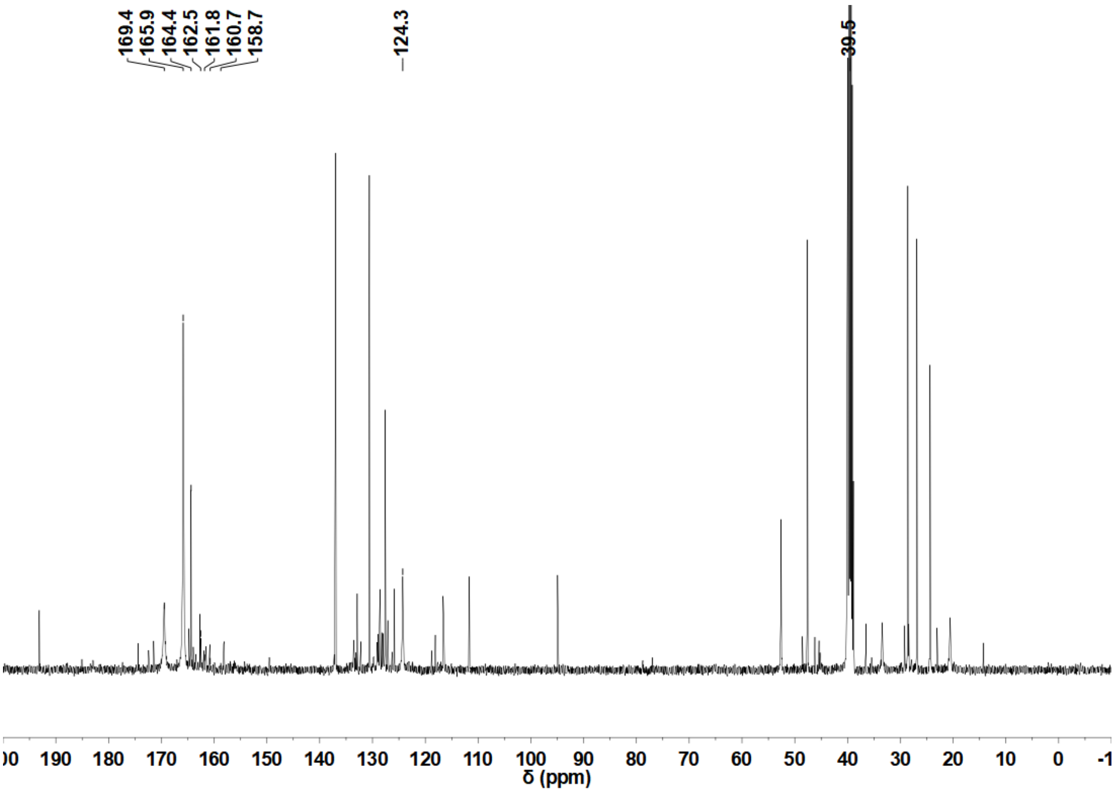

Figure S4. *In situ* ^{13}C NMR spectra (1 h). The CuCl (0.04 mmol) and Xantphos (0.04 mmol) were added to a NMR tube in the glovebox. Then iodobenzene (0.4 mmol, **1a**), 2'-hydroxyacetophenone (0.5 mmol, **2a**), PMHS (Si-H: 2.0 mmol) and [D$_6$]DMSO (2 mL) were injected by syringe. At room temperature, the tube was flushed with [^{13}C]CO_2 gas for three times. The tube was heated to 120 °C for 1 h.

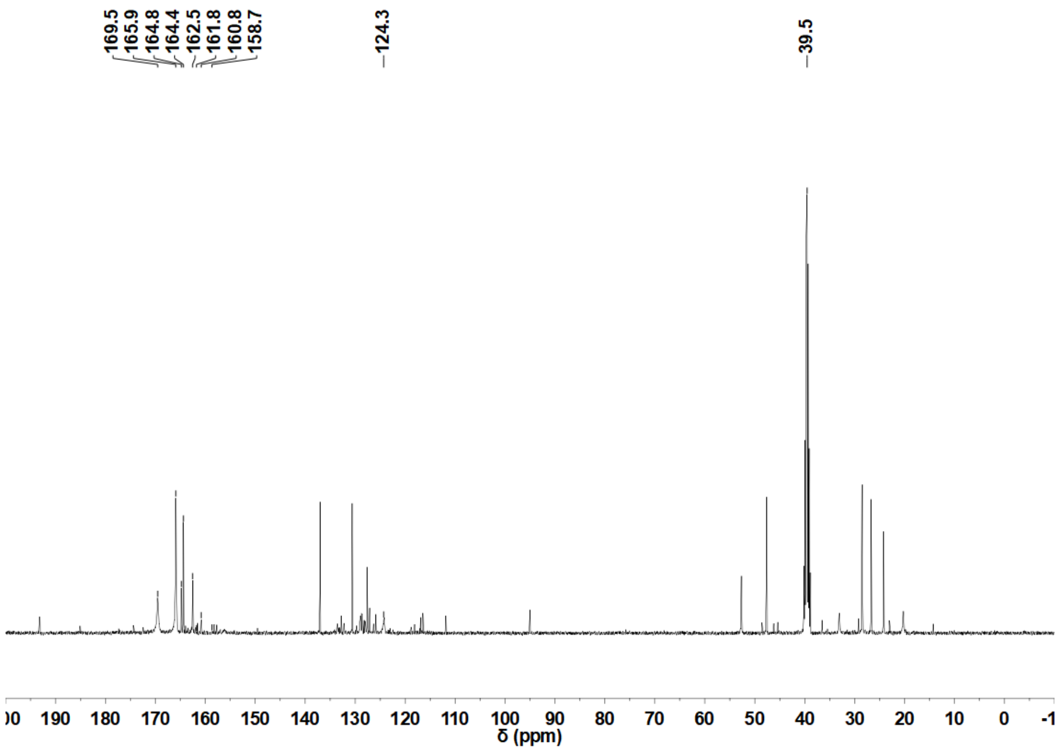

Figure S5**.** *In situ* ^{13}C NMR spectra (2 h). The CuCl (0.04 mmol) and Xantphos (0.04 mmol) were added to a NMR tube in the glovebox. Then iodobenzene (0.4 mmol, **1a**), 2'-hydroxyacetophenone (0.5 mmol, **2a**), PMHS (Si-H: 2.0 mmol) and [D$_6$]DMSO (2 mL) were injected by syringe. At room temperature, the tube was flushed with [^{13}C]CO_2 gas for three times. The tube was heated to 120 °C for 2 h.

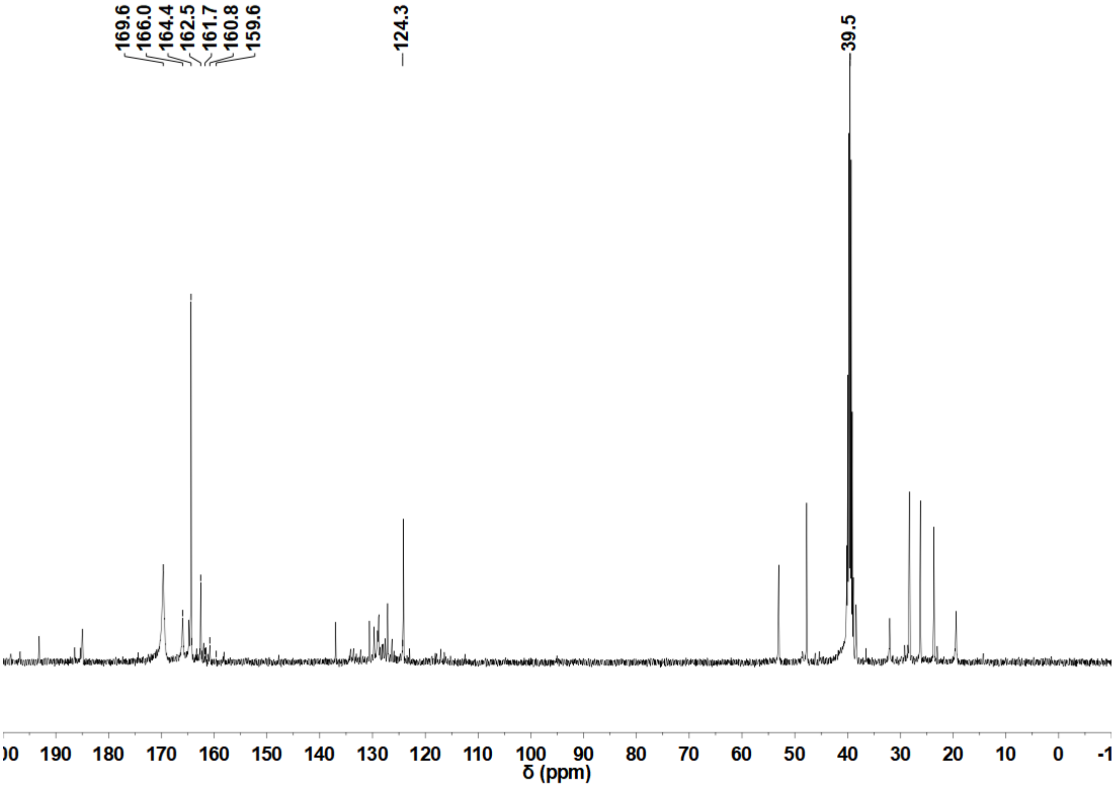

Figure S6**.** *In situ* ^{13}C NMR spectra (4 h). The CuCl (0.04 mmol) and Xantphos (0.04 mmol) were added to a NMR tube in the glovebox. Then iodobenzene (0.4 mmol, **1a**), 2'-hydroxyacetophenone (0.5 mmol,

2a), PMHS (Si-H: 2.0 mmol) and [D$_6$]DMSO (2 mL) were injected by syringe. At room temperature, the tube was flushed with [^{13}C]CO_2 gas for three times. The tube was heated to 120 °C for 4 h.

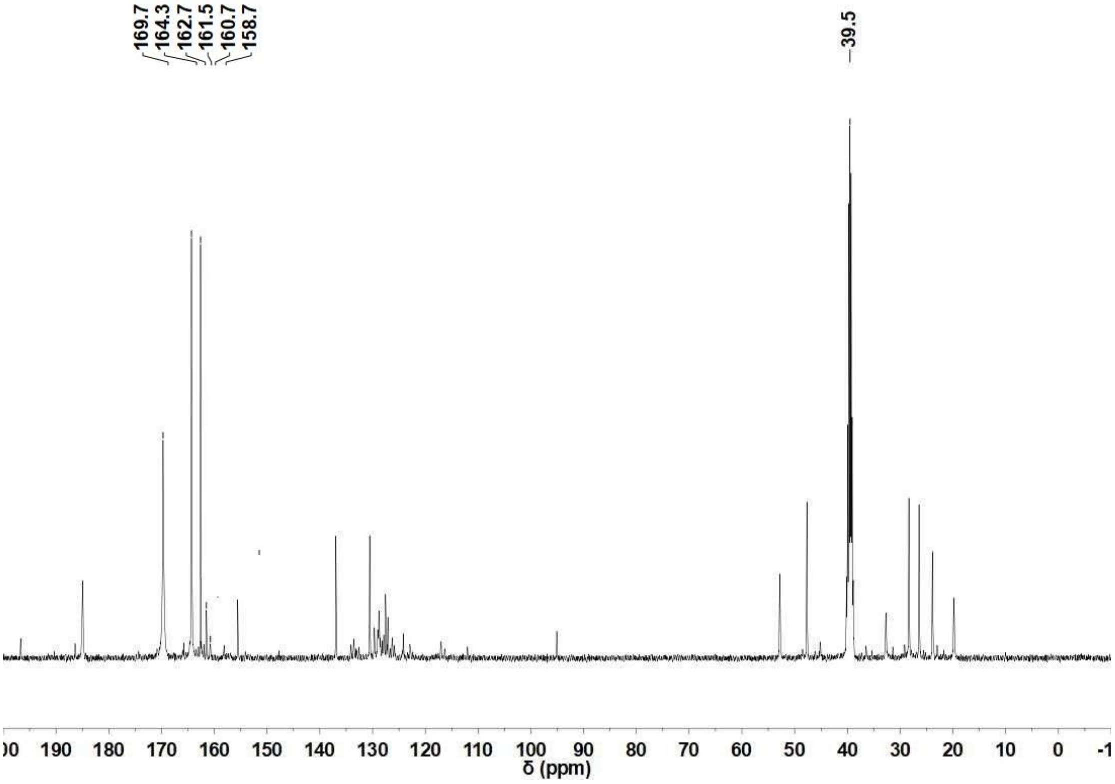

Figure S7. *In situ* ^{13}C NMR spectra (8 h). The CuCl (0.04 mmol) and Xantphos (0.04 mmol) were added to a NMR tube in the glovebox. Then iodobenzene (0.4 mmol, **1a**), 2'-hydroxyacetophenone (0.5 mmol, **2a**), PMHS (Si-H: 2.0 mmol) and [D$_6$]DMSO (2 mL) were injected by syringe. At room temperature, the tube was flushed with [^{13}C]CO_2 gas for three times. The tube was heated to 120 °C for 8 h.

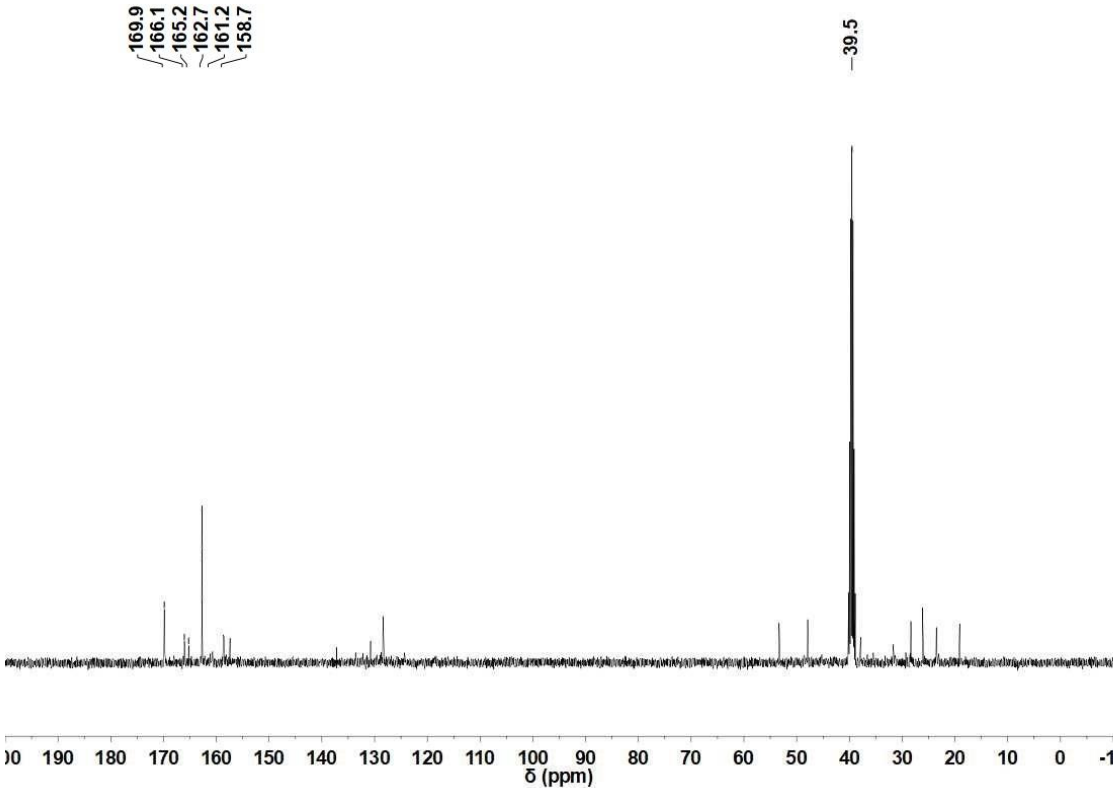

Figure S8. *In situ* ^{13}C NMR spectra (20 h). The CuCl (0.04 mmol) and Xantphos (0.04 mmol) were added to a NMR tube in the glovebox. Then iodobenzene (0.4 mmol, **1a**), 2'-hydroxyacetophenone (0.5 mmol, **2a**), PMHS (Si-H: 2.0 mmol) and [D_6]DMSO (2 mL) were injected by syringe. At room temperature, the tube was flushed with [^{13}C]CO_2 gas for three times. The tube was heated to 120 °C for 20 h.

^{13}C NMR spectra of 2-phenyl-*4H*-chromen-4-one, 2-acetylphenyl benzoate, 2-acetylphenyl formate

The characteristic peak of ^{13}C NMR spectrum of 2-phenyl-*4H*-chromen-4-one is at 162.5 ppm.

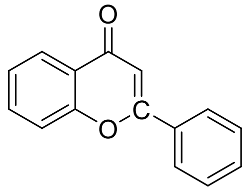

2-phenyl-4*H*-chromen-4-one

^{13}C NMR (100 MHz, DMSO-d_6) δ 177.1, 162.5, 155.8, 134.3, 131.8, 131.1, 129.1, 126.3, 125.5, 124.8, 123.3, 118.5, 106.9.

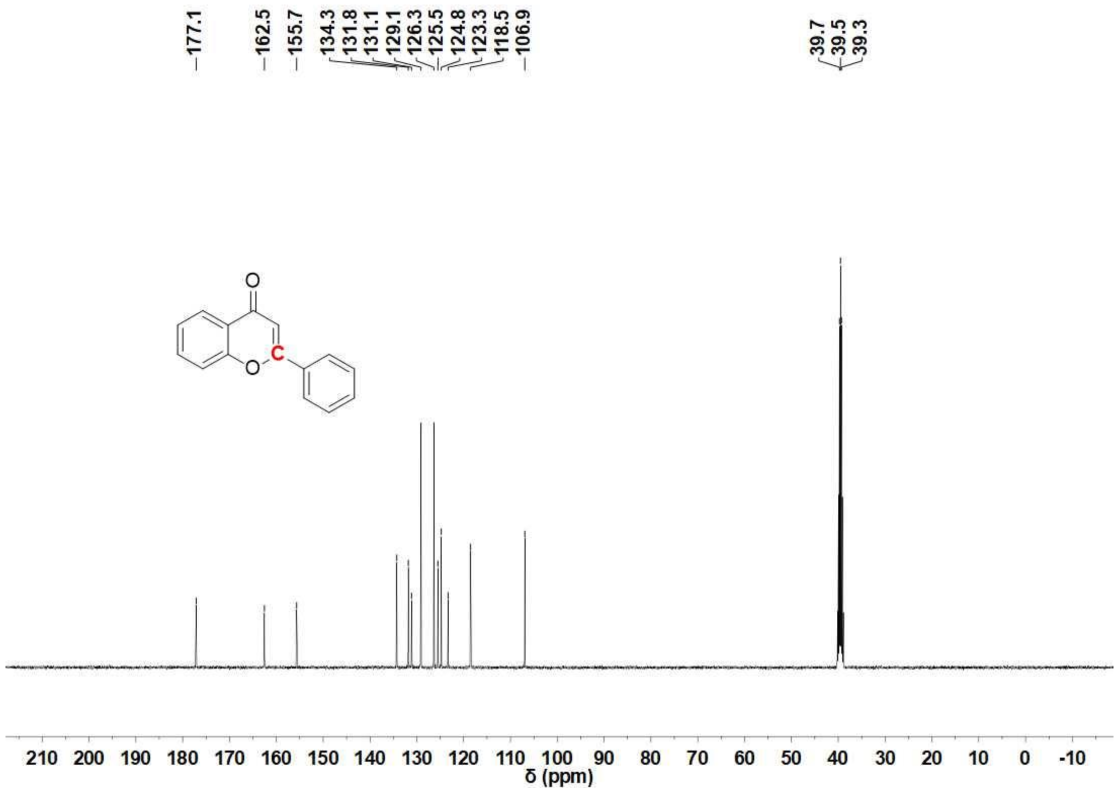

Figure S9

The characteristic peak of ^{13}C NMR spectrum of 2-acetylphenyl benzoate is at 164.6 ppm

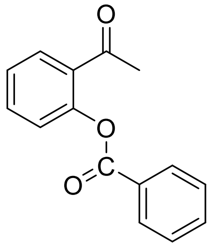

2-acetylphenyl benzoate

^{13}C NMR (101 MHz, DMSO-d_6) δ 197.4, 164.6, 148.6, 134.0, 133.7, 130.8, 130.5, 129.9, 129.9, 129.0, 126.4, 124.1, 29.5.

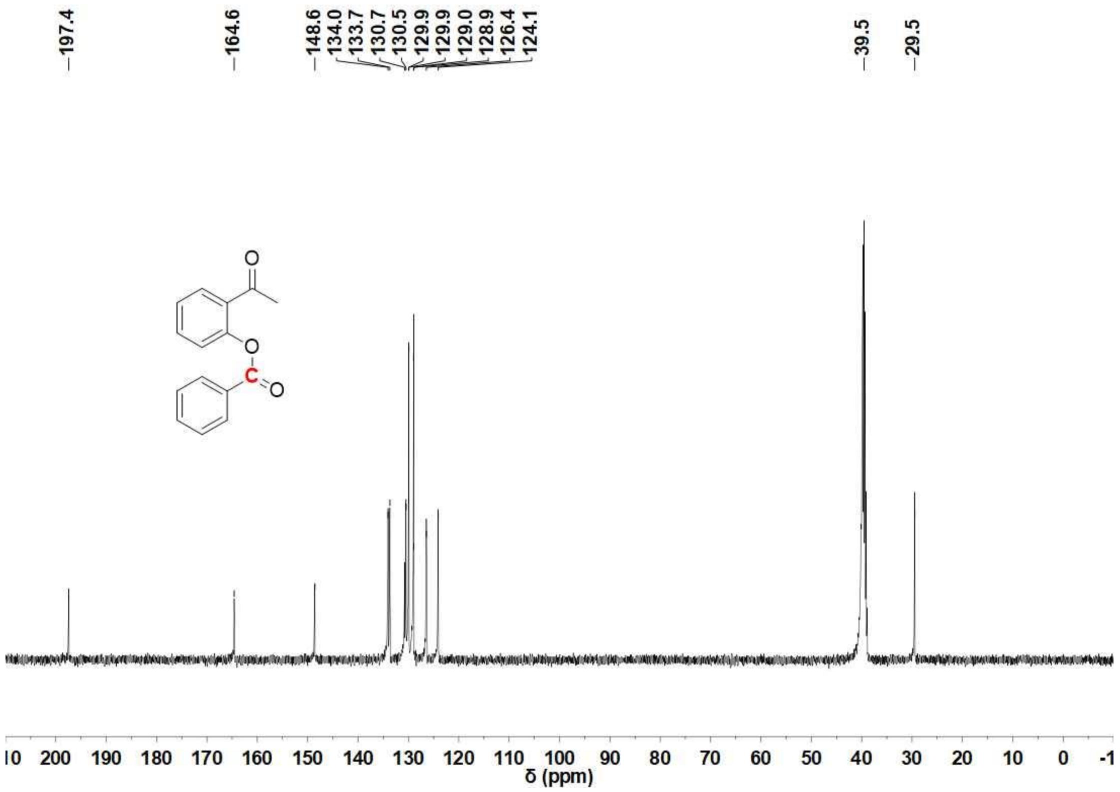

Figure S10

The characteristic peak of ^{13}C NMR spectrum of 2-acetylphenyl formate is at 160.7 ppm.

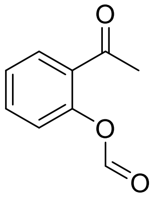

2-acetylphenyl formate

^{13}C NMR (100 MHz, DMSO-d_6) δ 160.7, 133.7, 130.4, 126.6, 123.5, 29.8.

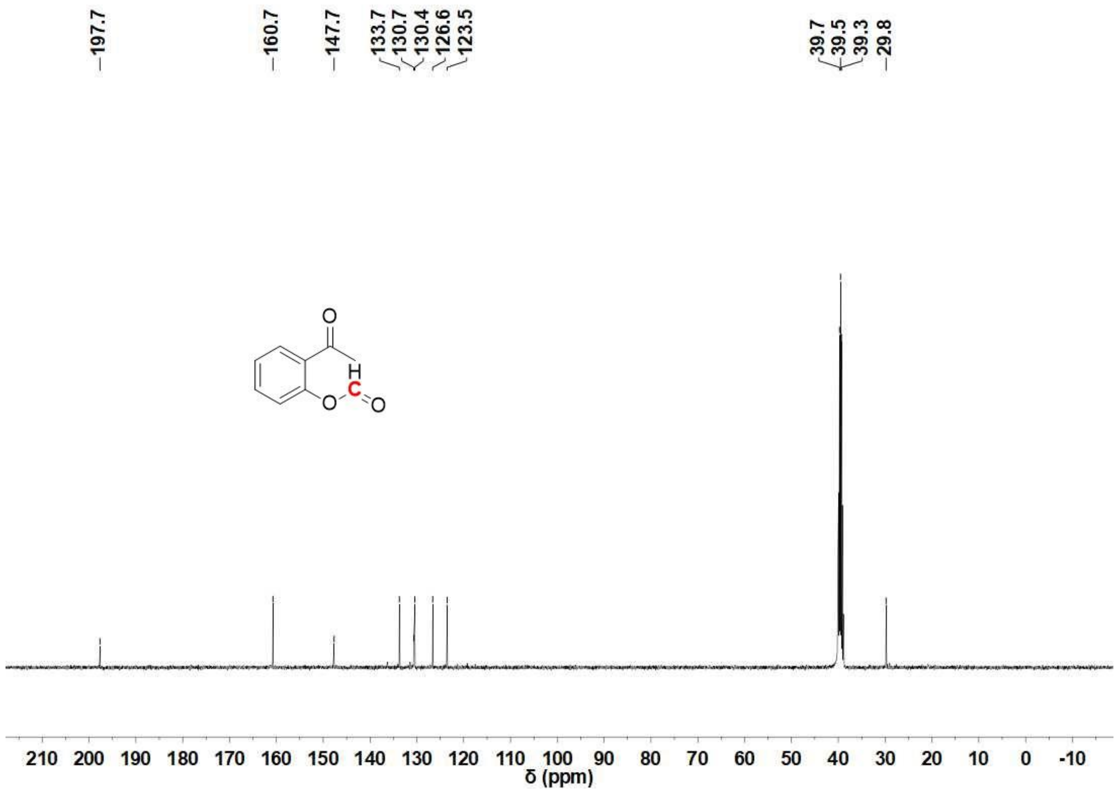

Figure S11

5 Characterization data

Experimental procedure:

Under nitrogen atmosphere, CuCl (10 mol%, 0.02 mmol), Xantphos (10 mol%, 0.02 mmol), **1** (0.2 mmol), **2** or **3** (0.24 mmol), DBU (0.6 mmol), DMSO (1 mL), PMHS (Si-H 4 equiv.) and a stirring bar were added into a 10 mL oven-dried sealed glass tube in glovebox (as shown in **Figure S1**). After sealed, the glass tube was brought out of the glovebox and CO_2 (20 mL) was injected by syringe. Then the mixture was stirred for 20 hours in a pre-heated-to-120 °C alloyed block. After the reaction was completed, the glass tube was cooled to room temperature and the pressure was carefully released. The residual silane was quenched with HCl (2 M). The mixture was extracted with ethyl acetate. The combined organic layers were washed with brine and dried over anhydrous Na_2SO_4. The residue was purified by flash chromatography on silica gel (eluting with petroleum ether /ethyl acetate = 20/1 to 5/1) to give the product **4aa**. Unless otherwise stated, the synthetic method is similar to the preparation of flavones **4aa**.

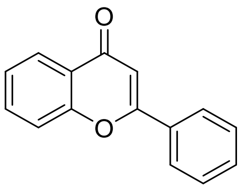

4aa

2-phenyl-4*H*-chromen-4-one

Purified by flash chromatography on silica gel eluting with petroleum ether /ethyl acetate = 20/1 to 5/1. Yield: 81%; white solid; m.p.: 102-104 °C.

^{1}H NMR (400 MHz, Chloroform-*d*) δ 8.23 – 8.21 (m, 1H), 7.98 – 7.86 (m, 2H), 7.69 (t, *J* = 7.7 Hz, 1H), 7.57 – 7.53 (m, 2H), 7.52 – 7.50 (m, 3H), 7.40 (t, *J* = 7.4 Hz, 1H), 6.84 (s, 1H).

^{13}C NMR (100 MHz, Chloroform-*d*) δ 178.7, 163.6, 156.3, 133.9, 131.7, 131.7, 129.1, 126.3, 125.7, 125.3, 123.9, 118.1, 107.5.

Data in accordance with those reported in ref. [1-2].

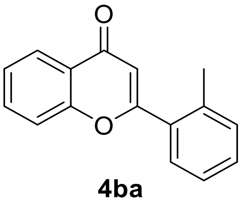

4ba

2-(*o*-tolyl)-4*H*-chromen-4-one

Purified by flash chromatography on silica gel eluting with petroleum ether /ethyl acetate = 20/1 to 5/1. Yield: 82%; colorless oil.

^{1}H NMR (400 MHz, Chloroform-*d*) δ 8.27 (dd, *J* = 7.9, 1.7 Hz, 1H), 7.72 – 7.68 (m, 1H), 7.55 – 7.49 (m, 2H), 7.47 – 7.40 (m, 2H), 7.34 – 7.32 (m, 2H), 6.50 (s, 1H), 2.49 (s, 3H).

^{13}C NMR (100 MHz, Chloroform-*d*) δ 178.5, 166.3, 156.6, 136.9, 133.9, 132.8, 131.4, 130.9, 129.4, 126.4, 125.9, 125.4, 123.9, 118.2, 112.1, 20.7.

Data in accordance with those reported in ref. [1-2].

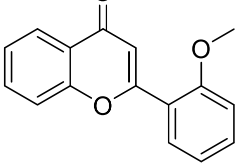

4ca

2-(2-methoxyphenyl)-4*H*-chromen-4-one

Purified by flash chromatography on silica gel eluting with petroleum ether /ethyl acetate = 20/1 to 5/1. Yield: 79%; yellow solid；m.p.:100-102 °C.

^{1}H NMR (400 MHz, Chloroform-*d*) δ 8.24 (dd, *J* = 8.0, 1.7 Hz, 1H), 7.91 (dd, *J* = 7.8, 1.7 Hz, 1H), 7.70 – 7.66 (m, 1H), 7.55 – 7.51 (m, 1H), 7.50 – 7.48 (m, 1H), 7.45 – 7.37 (m, 1H), 7.18 (s, 1H), 7.12 – 7.10 (m, 1H), 7.05 (d, *J* = 8.4 Hz, 1H), 3.93 (s, 3H).

^{13}C NMR (100 MHz, Chloroform-*d*) δ 179.2, 161.2, 158.2, 156.7, 133.8, 132.6, 129.4, 125.8, 125.1, 123.9, 120.9, 120.9, 118.2, 112.7, 111.9, 55.8.

Data in accordance with those reported in ref. [3].

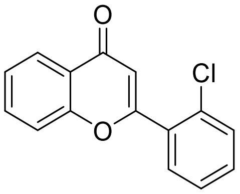

4da

2-(2-chlorophenyl)-4*H*-chromen-4-one

Purified by flash chromatography on silica gel eluting with petroleum ether /ethyl acetate = 20/1 to 5/1.

Yield: 76%; yellow solid m.p.: 112-114 °C

^{1}H NMR (400 MHz, Chloroform-*d*) δ 8.30 – 8.22 (m, 1H), 7.73 – 7.69 (m, 1H), 7.64 (d, *J* = 7.4 Hz, 1H), 7.53 (t, *J* = 7.4 Hz, 2H), 7.48 – 7.40 (m, 3H), 6.67 (s, 1H).

^{13}C NMR (100 MHz, Chloroform-*d*) δ 178.4, 162.9, 156.7, 134.1, 131.9, 130.9, 130.8, 127.2, 125.9, 125.5, 124.2, 118.3, 113.4.

Data in accordance with those reported in ref. [3].

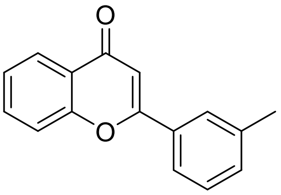

4ea

2-(*m*-tolyl)-4*H*-chromen-4-one

Purified by flash chromatography on silica gel eluting with petroleum ether /ethyl acetate = 20/1 to 5/1.

Yield: 84%; white solid; m.p.: 103-106 °C.

^{1}H NMR (400 MHz, Chloroform-*d*) δ 8.11 – 8.09 (m, 1H), 7.58 – 7.54 (m, 3H), 7.45 – 7.43 (m, 1H), 7.33 – 7.24 (m, 2H), 7.20 (d, *J* = 7.6 Hz, 1H), 6.72 (s, 1H), 2.31 (s, 3H).

^{13}C NMR (101 MHz, Chloroform-*d*) δ 179.0, 164.1, 156.4, 139.0, 134.0, 132.7, 131.7, 129.1, 127.0, 125.8, 125.4, 123.9, 123.7, 118.2, 107.49, 21.6.

Data in accordance with those reported in ref. [1-2].

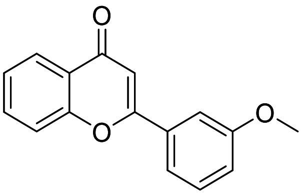

4fa

2-(3-methoxyphenyl)-4*H*-chromen-4-one

Purified by flash chromatography on silica gel eluting with petroleum ether /ethyl acetate = 20/1 to 5/1.

Yield: 85%; white solid; m.p.: 93-95 ºC.

^{1}H NMR (400 MHz, Chloroform-*d*) δ 8.25 – 8.22 (m, 1H), 7.74 – 7.68 (m, 1H), 7.58 (d, *J* = 8.5 Hz, 1H), 7.55 – 7.49 (m, 1H), 7.47 – 7.40 (m, 3H), 7.10 – 7.07 (m, 1H), 6.83 (s, 1H), 3.90 (s, 3H).

^{13}C NMR (100 MHz, Chloroform-*d*) δ 178.7, 160.2, 156.4, 134.0, 133.2, 130.3, 125.8, 125.4, 118.9, 118.3, 117.4, 111.9, 107.9, 55.6.

Data in accordance with those reported in ref. [4].

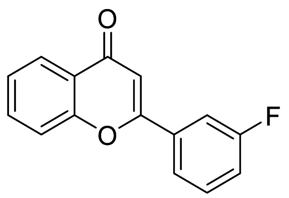

4ga

2-(3-fluorophenyl)-4*H*-chromen-4-one

Purified by flash chromatography on silica gel eluting with petroleum ether /ethyl acetate = 20/1 to 5/1. Yield: 82%; white solid; m.p.: 96-98 °C.

^{1}H NMR (400 MHz, Chloroform-*d*) δ 8.24 (dd, *J* = 7.9, 1.6 Hz, 1H), 7.76 – 7.69 (m, 2H), 7.66 – 7.63 (m, 1H), 7.58 (d, *J* = 8.4 Hz, 1H), 7.53 – 7.46 (m, 1H), 7.44 (t, *J* = 7.5 Hz, 1H), 7.28 – 7.22 (m, 1H), 6.84 (s, 1H).

^{13}C NMR (100 MHz, Chloroform-*d*) δ 178.5, 163.2 (d, *J* = 233.9 Hz) 161.9, 156.3, 134.2, 134.1, 134.1, 130.9 (d, *J* = 8.4 Hz), 125.8 (d, *J* = 46.4 Hz), 124.0, 122.1 (d, *J* = 3.3 Hz), 118.7 (d, *J* = 21.2 Hz), 118.2, 113.5 (d, *J* = 23.9 Hz), 108.3.

^{19}F NMR (377 MHz, Chloroform-*d*) δ -111.16.

Data in accordance with those reported in ref. [5].

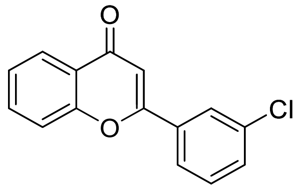

4ha

2-(3-chlorophenyl)-4*H*-chromen-4-one

Purified by flash chromatography on silica gel eluting with petroleum ether /ethyl acetate = 20/1 to 5/1. Yield: 84%; white solid; m.p.: 110-112 °C.

^{1}H NMR (400 MHz, Chloroform-*d*) δ 8.20 (d, *J* = 7.6 Hz, 1H), 7.88 (s, 1H), 7.75 (d, *J* = 7.6 Hz, 1H), 7.69 (t, *J* = 7.7 Hz, 1H), 7.55 (d, *J* = 8.5 Hz, 1H), 7.48 (d, *J* = 8.1 Hz, 1H), 7.41 (q, *J* = 7.5 Hz, 2H), 6.82 (s, 1H).

^{13}C NMR (100 MHz, Chloroform-*d*) δ 178.6, 162.0, 156.2, 135.3, 134.2, 133.5, 131.7, 130.4, 126.4, 125.8, 125.6, 124.5, 118.2, 108.4.

Data in accordance with those reported in ref. [6].

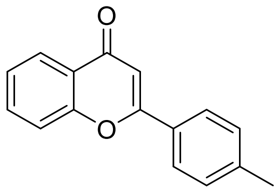

4ia

2-(*p*-tolyl)-4*H*-chromen-4-one

Purified by flash chromatography on silica gel eluting with petroleum ether /ethyl acetate = 20/1 to 5/1.
Yield: 84%; white solid; m.p.: 108-109 °C.

^{1}H NMR (400 MHz, Chloroform-*d*) δ 8.24 (dd, *J* = 7.9, 1.7 Hz, 1H), 7.81 – 7.78 (m, 2H), 7.69 – 7.65 (m, 1H), 7.56 – 7.51 (m, 1H), 7.41 – 7.37 (m, 1H), 7.30 – 7.28 ((m, 2H)), 6.89 (s, 1H), 2.42 (s, 3H).

^{13}C NMR (101 MHz, Chloroform-*d*) δ 179.1, 164.2, 156.4, 142.6, 134.0, 130.9, 129.9, 126.4, 125.8, 125.4, 123.8, 118.2, 106.8.

Data in accordance with those reported in ref. [1-2].

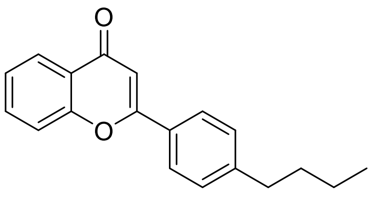

4ja

2-(4-butylphenyl)-4*H*-chromen-4-one

Purified by flash chromatography on silica gel eluting with petroleum ether /ethyl acetate = 20/1 to 5/1.
Yield: 88%; colorless oil.

^{1}H NMR (400 MHz, Chloroform-*d*) δ 8.23 (d, *J* = 7.9 Hz, 1H), 7.82 (d, *J* = 7.9 Hz, 2H), 7.68 (t, *J* = 7.8 Hz, 1H), 7.55 (d, *J* = 8.4 Hz, 1H), 7.40 (t, *J* = 7.5 Hz, 1H), 7.31 (d, *J* = 7.9 Hz, 2H), 6.81 (s, 1H), 2.68 (t, *J* = 7.7 Hz, 2H), 1.67 – 1.59 (m, 2H), 1.45 – 1.33 (m, 3H), 0.94 (t, *J* = 7.4 Hz, 3H).

^{13}C NMR (100 MHz, Chloroform-*d*) δ 178.7, 163.9, 156.4, 147.4, 133.8, 129.2, 129.1, 126.4, 125.7, 125.3, 124.0, 118.2, 107.0, 35.7, 33.4, 22.4, 14.0.

HRMS (ESI): Calcd. For $C_{19}H_{19}O_2$ [M+H]$^+$: 279.1379, Found: 279.1375.

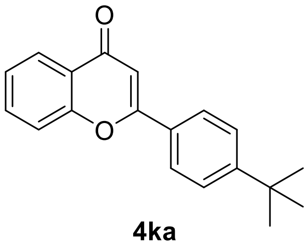

4ka

2-(4-(*tert*-butyl)phenyl)-4*H*-chromen-4-one

Purified by flash chromatography on silica gel eluting with petroleum ether /ethyl acetate = 20/1 to 5/1.
Yield: 81%; white solid; m.p.: 108-109 °C.

^{1}H NMR (400 MHz, Chloroform-*d*) δ 8.26 – 8.23 (m, 1H), 7.90 – 7.84 (m, 2H), 7.72 – 7.68 (m, 1H), 7.59 – 7.52 (m, 3H), 7.44 – 7.40 (m, 1H), 6.83 (s, 1H), 1.37 (s, 9H).

^{13}C NMR (100 MHz, Chloroform-*d*) δ 178.8, 163.9, 156.4, 155.5, 133.9, 129.0, 126.3, 126.2, 125.8, 125.3, 124.0, 118.2, 107.1, 35.2, 31.2.

Data in accordance with those reported in ref. [2].

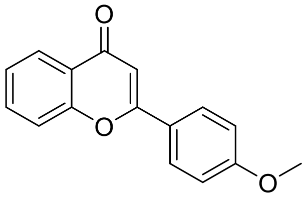

4la

2-(4-methoxyphenyl)-4*H*-chromen-4-one

Purified by flash chromatography on silica gel eluting with petroleum ether /ethyl acetate = 20/1 to 5/1. Yield: 82%. white solid; m.p.: 162-164 °C.

^{1}H NMR (400 MHz, Chloroform-*d*) δ 8.21 (d, *J* = 7.5 Hz, 1H), 7.87 (d, *J* = 8.6 Hz, 2H), 7.67 (t, *J* = 7.4 Hz, 1H), 7.54 (d, *J* = 8.2 Hz, 1H), 7.39 (t, *J* = 7.2 Hz, 1H), 7.01 (d, *J* = 8.6 Hz, 2H), 6.74 (s, 1H), 3.87 (s, 3H).

^{13}C NMR (101 MHz, Chloroform-*d*) δ 163.61, 162.54, 156.29, 133.72, 128.13, 125.75, 125.21, 124.1, 118.1, 114.6, 106.2, 55.6.

Data in accordance with those reported in ref. [1-2].

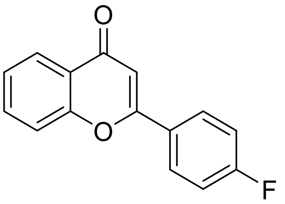

4ma

2-(4-fluorophenyl)-4*H*-chromen-4-one

Purified by flash chromatography on silica gel eluting with petroleum ether /ethyl acetate = 20/1 to 5/1. Yield: 76%; white solid; m.p.: 140 - 142 °C.

^{1}H NMR (400 MHz, Chloroform-*d*) δ 8.23 (dd, *J* = 7.9, 1.6 Hz, 1H), 7.98 – 7.87 (m, 2H), 7.73 – 7.71 (m, 1H), 7.56 (d, *J* = 8.6 Hz, 1H), 7.43 (t, *J* = 7.5 Hz, 1H), 7.21 (t, *J* = 8.6 Hz, 2H), 6.80 (s, 1H).

^{13}C NMR (101 MHz, Chloroform-*d*) δ 178.6, 163.7 (d, *J* = 252.1 Hz), 162.7, 156.3, 134.1, 128.7 (d, *J* = 8.6 Hz), 128.0 (d, *J* = 3.2 Hz), 125.9, 125.5, 123.9, 118.1, 116.4 (d, *J* = 22.2 Hz), 107.4.

^{19}F NMR (377 MHz, Chloroform-*d*) δ -107.25.

Data in accordance with those reported in ref. [1, 5].

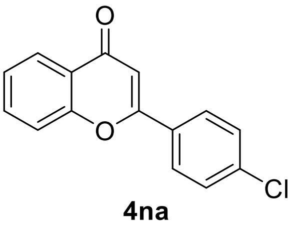

4na

2-(4-chlorophenyl)-4*H*-chromen-4-one

Purified by flash chromatography on silica gel eluting with petroleum ether /ethyl acetate = 20/1 to 5/1. Yield: 74%; white solid; m.p.: 188-190 °C.

^{1}H NMR (400 MHz, Chloroform-*d*) δ 8.24 – 8.21 (m, 1H), 7.90 – 7.82 (m, 2H), 7.73 – 7.69 (m, 1H), 7.57 – 7.55 (m, 1H), 7.53 – 7.47 (m, 2H), 7.45 – 7.41 (m, 1H), 6.79 (s, 1H).

^{13}C NMR (101 MHz, Chloroform-*d*) δ 178.3, 162.3, 156.2, 137.9, 133.9, 130.3, 129.4, 127.6, 125.8, 125.4, 123.9, 118.1, 107.7.
Data in accordance with those reported in ref. [1-2].

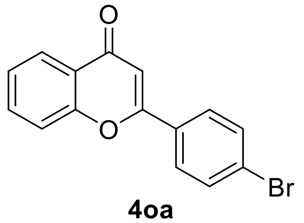

4oa

2-(4-bromophenyl)-4*H*-chromen-4-one

Purified by flash chromatography on silica gel eluting with petroleum ether /ethyl acetate = 20/1 to 5/1.
Yield: 74%; white solid; m.p.: 178-189 °C.
^{1}H NMR (400 MHz, Chloroform-*d*) δ 8.22 – 8.20 (m, 1H), 7.91 – 7.81 (m, 2H), 7.72 – 7.67 (m, 1H), 7.56 – 7.54 (m, 1H), 7.52 – 7.45 (m, 2H), 7.44 – 7.38 (m, 1H), 6.78 (s, 1H).
^{13}C NMR (101 MHz, Chloroform-*d*) δ 178.3, 162.3, 156.2, 133.9, 132.7, 129.4, 127.6, 125.8, 125.4, 123.9, 118.1, 107.7.
Data in accordance with those reported in ref. [2].

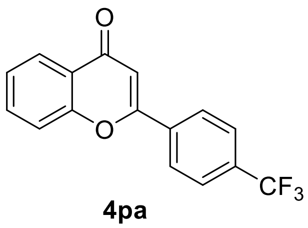

4pa

2-(4-(trifluoromethyl)phenyl)-4*H*-chromen-4-one

Purified by flash chromatography on silica gel eluting with petroleum ether /ethyl acetate = 20/1 to 5/1.
Yield: 61%. white solid; m.p.: 139-140 °C.
^{1}H NMR (400 MHz, Chloroform-*d*) δ 8.25 (dd, *J* = 7.9, 1.7 Hz, 1H), 8.05 (d, *J* = 8.2 Hz, 2H), 7.80 (d, *J* = 8.2 Hz, 2H), 7.77 – 7.70 (m, 1H), 7.60 (d, *J* = 8.3 Hz, 1H), 7.49 – 7.44 (m, 1H), 6.88 (s, 1H).
^{13}C NMR (100 MHz, Chloroform-*d*) δ 178.4, 161.8, 156.4, 135.4, 134.3, 133.3 (q, *J* = 32.7 Hz), 126.8, 126.1 (q, *J* =3.7 Hz), 125.7, 125.1, 124.1, 123.3 (q, *J* = 270.6 Hz), 118.3, 108.9.
^{19}F NMR (377 MHz, Chloroform-*d*) δ -62.97.
Data in accordance with those reported in ref. [1-2].

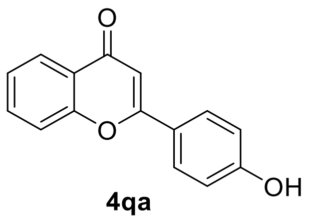

4qa

2-(4-hydroxyphenyl)-4*H*-chromen-4-one

Purified by flash chromatography on silica gel eluting with petroleum ether /ethyl acetate = 20/1 to 5/1.
Yield: 64%; yellow solid; m.p.: 268-270 ^oC.
^{1}H NMR (400 MHz, DMSO-d_6) δ 8.03 (d, J = 7.9 Hz, 1H), 7.96 (d, J = 8.8 Hz, 2H), 7.83 – 7.76 (m, 1H), 7.73 (d, J = 7.8 Hz, 1H), 7.50 – 7.44 (m, 1H), 6.94 (d, J = 8.8 Hz, 2H), 6.86 (s, 1H).
^{13}C NMR (100 MHz, DMSO-d_6) δ 177.1, 163.2, 161.1, 155.7, 134.2, 128.5, 125.5, 124.8, 123.4, 121.7, 118.5, 116.1, 104.9.
Data in accordance with those reported in ref. [5]

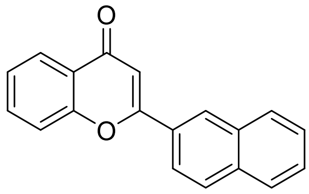

4ra

2-(naphthalen-2-yl)-4*H*-chromen-4-one

Purified by flash chromatography on silica gel eluting with petroleum ether /ethyl acetate = 20/1 to 5/1.
Yield: 82%; white solid; m.p.: 141-143 °C.
^{1}H NMR (400 MHz, Chloroform-*d*) δ 8.28 (bs, 1H), 8.09 (dd, J = 7.9, 1.7 Hz, 1H), 7.81 – 7.68 (m, 4H), 7.57 – 7.53 (m, 1H), 7.48 – 7.38 (m, 3H), 7.28 – 7.24 (m 1H), 6.81 (s, 1H).
^{13}C NMR (101 MHz, Chloroform-*d*) δ 178.9, 163.7, 156.4, 134.8, 134.1, 133.0, 129.7, 129.2, 129.1, 128.8, 127.9, 127.2, 127.1, 125.9, 125.5, 123.9, 122.2, 122.6, 118.2, 107.8.
Data in accordance with those reported in ref. [1].

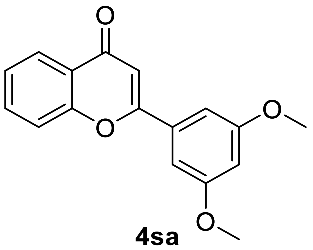

4sa

2-(3,5-dimethoxyphenyl)-4*H*-chromen-4-one

Purified by flash chromatography on silica gel eluting with petroleum ether /ethyl acetate = 20/1 to 5/1.
Yield: 86%; white solid; m.p.: 141-143 °C.
^{1}H NMR (400 MHz, Chloroform-*d*) δ 8.09 (d, J = 7.5 Hz, 1H), 7.62 – 7.52 (m, 1H), 7.42 (t, J = 5.8 Hz, 1H), 7.29 (t, J = 7.6 Hz, 1H), 6.87 (d, J = 2.0 Hz, 2H), 6.64 (s, 1H), 6.46 (p, J = 2.3 Hz, 1H), 3.74 (s, 6H).
^{13}C NMR (101 MHz, Chloroform-*d*) δ 178.1, 162.8, 160.9, 155.2, 133.6, 133.3, 125.4, 125.0, 123.7, 118.0, 107.5, 104.2, 103.2, 55.3.
Data in accordance with those reported in ref. [1].

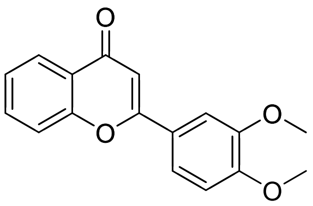

4ta

2-(3,4-dimethoxyphenyl)-4*H*-chromen-4-one

Purified by flash chromatography on silica gel eluting with petroleum ether /ethyl acetate = 20/1 to 5/1. Yield: 85%; white solid; m.p.: 118–120 ºC.

^{1}H NMR (400 MHz, Chloroform-*d*) δ 8.14 – 8.11 (m, 1H), 7.66 – 7.53 (m, 1H), 7.47 – 7.44 (m, 2H), 7.35 – 7.27 (m, 2H), 6.88 (d, *J* = 8.5 Hz, 1H), 6.66 (s, 1H), 3.90 (s, 3H), 3.88 (s, 3H).

^{13}C NMR (101 MHz, Chloroform-*d*) δ 178.5, 163.3, 156.1, 152.0, 149.2, 133.5, 125.5, 124.1, 119.9, 117.9, 111.1, 108.7, 106.6, 56.0.

Data in accordance with those reported in ref. [4].

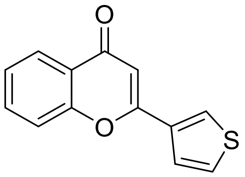

4ua

2-(thiophen-3-yl)-4*H*-chromen-4-one

Purified by flash chromatography on silica gel eluting with petroleum ether /ethyl acetate = 20/1 to 5/1. Yield: 73%; white solid; m.p.: 112-113 ºC.

^{1}H NMR (400 MHz, Chloroform-*d*) δ 8.23 (dd, *J* = 8.0, 1.7 Hz, 1H), 8.04 – 8.03 (m, 1H), 7.72 – 7.67 (m, 1H), 7.56 – 7.42 (m, 4H), 6.72 (s, 1H).

^{13}C NMR (101 MHz, Chloroform-*d*) δ 178.83, 159.9, 156.2, 134.0, 127.6, 127.2, 125.9, 125.4, 125.2, 124.0, 118.1, 107.2.

Data in accordance with those reported in ref. [2].

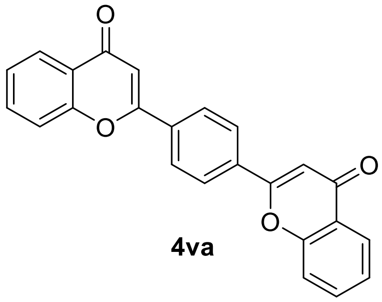

4va

4'-(2-Chromonyl)flavone

Purified by flash chromatography on silica gel eluting with petroleum ether /ethyl acetate = 20/1 to 5/1. Yield: 55%; white solid; m.p.: 298-299 ºC.

^{1}H NMR (400 MHz, Chloroform-*d*) δ 8.26 – 8.24 (m, 2H), 8.01 – 7.89 (m, 4H), 7.76 – 7.67 (m, 2H), 7.59 (d, *J* = 8.4 Hz, 2H), 7.43 (t, *J* = 7.4 Hz, 2H), 6.98 (s, 2H).

^{13}C NMR (101 MHz, Chloroform-*d*) δ 178.7, 163.6, 156.4, 133.9, 126.5, 125.9, 125.4, 124.5, 118.2, 107.8.
Data in accordance with those reported in ref. [12].

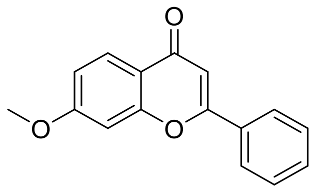

4ab
7-methoxy-2-phenyl-4*H*-chromen-4-one

Purified by flash chromatography on silica gel eluting with petroleum ether /ethyl acetate = 20/1 to 5/1.
Yield: 80%; white solid; m.p.: 109-110 °C.
^{1}H NMR (400 MHz, Chloroform-*d*) δ 8.12 (d, *J* = 8.7 Hz, 1H), 7.96 – 7.83 (m, 2H), 7.52 – 7.48 (m, 3H), 7.01 – 6.90 (m, 2H), 6.79 (s, 1H), 3.91 (s, 3H).
^{13}C NMR (100 MHz, Chloroform-*d*) δ 178.1, 164.3, 163.2, 158.1, 131.8, 131.5, 129.0, 127.1, 126.2, 117.7, 114.6, 107.4, 100.4, 55.9.
Data in accordance with those reported in ref. [2].

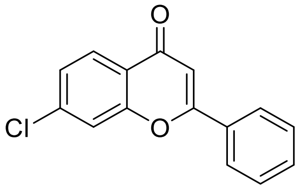

4ac
7-chloro-2-phenyl-4*H*-chromen-4-one

Purified by flash chromatography on silica gel eluting with petroleum ether /ethyl acetate = 20/1 to 5/1.
Yield: 78%; white solid; m.p.: 158-160 °C.
^{1}H NMR (400 MHz, Chloroform-*d*) δ 8.18 (d, *J* = 8.5 Hz, 1H), 7.96 – 7.87 (m, 2H), 7.62 (d, *J* = 1.9 Hz, 1H), 7.55 (mm, 4H), 7.40 (dd, *J* = 8.6, 1.9 Hz, 1H), 6.85 (s, 1H).
^{13}C NMR (101 MHz, Chloroform-*d*) δ 178.2, 164.2, 156.5, 140.2, 131.3, 130.4, 129.3, 127.3, 126.5, 126.4, 122.5, 118.4, 107.7.
Data in accordance with those reported in ref. [2].

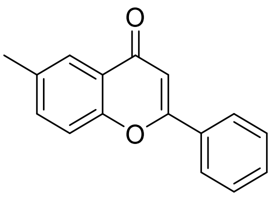

4ad
6-methyl-2-phenyl-4*H*-chromen-4-one

Purified by flash chromatography on silica gel eluting with petroleum ether /ethyl acetate = 20/1 to 5/1.
Yield: 82%; white solid; m.p.: 123-124 °C.

^{1}H NMR (400 MHz, Chloroform-*d*) δ 8.06 – 7.97 (s, 1H), 7.99 – 7.90 (m, 2H), 7.61 – 7.45 (m, 5H), 6.81 (s, 1H), 2.46 (s, 3H).
^{13}C NMR (100 MHz, Chloroform-*d*) δ 178.8, 163.4, 154.6, 135.3, 135.1, 131.9, 131.6, 129.1, 126.3, 125.1, 123.6, 117.9, 107.4, 21.0.
Data in accordance with those reported in ref. [2].

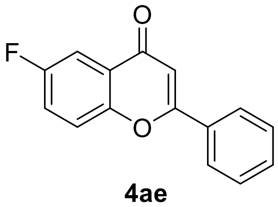

4ae

6-fluoro-2-phenyl-4*H*-chromen-4-one

Purified by flash chromatography on silica gel eluting with petroleum ether /ethyl acetate = 20/1 to 5/1. Yield: 76%; white solid; m.p.: 128-130 °C.
^{1}H NMR (400 MHz, Chloroform-*d*) δ 7.91 – 7.83 (m, 3H), 7.66 – 7.46 (m, 4H), 7.41 (td, *J* = 8.9, 8.4, 3.1 Hz, 1H), 6.80 (s, 1H).
^{13}C NMR (100 MHz, Chloroform-*d*) δ 177.8, 163.9, 159.8 (d, *J* = 245.1 Hz), 152.3, 131.7, 129.3, 126.5, 124.4 (d, *J* = 7.9 Hz), 122.1 (d, *J* = 25.4 Hz), 120.3 (d, *J* = 8.0 Hz), 110.8 (d, *J* = 23.8 Hz), 107.0.

^{19}F NMR (376 MHz, Chloroform-*d*) δ -115.05.

Data in accordance with those reported in ref. [2].

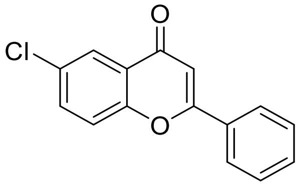

4af

6-chloro-2-phenyl-4*H*-chromen-4-one

Purified by flash chromatography on silica gel eluting with petroleum ether /ethyl acetate = 20/1 to 5/1. Yield: 75%; white solid; m.p.: 183-185 °C.
^{1}H NMR (400 MHz, Chloroform-*d*) δ 8.18 (d, *J* = 2.6 Hz, 1H), 7.94 – 7.85 (m, 2H), 7.63 (d, *J* = 2.6 Hz, 1H), 7.58 – 7.46 (m, 4H), 6.83 (s, 1H).
^{13}C NMR (100 MHz, Chloroform-*d*) δ 177.7, 164.2, 154.7, 134.3, 132.1, 131.5, 131.3, 129.3, 126.5, 124.8, 119.9, 107.4.
Data in accordance with those reported in ref. [2].

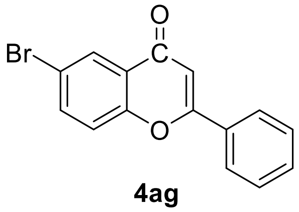

4ag

6-bromo-2-phenyl-4*H*-chromen-4-one

Purified by flash chromatography on silica gel eluting with petroleum ether /ethyl acetate = 20/1 to 5/1. Yield: 73%; white solid; m.p.: 190-191 ºC.

^{1}H NMR (400 MHz, Chloroform-*d*) δ 8.36 – 8.35 (m, 1H), 7.92 – 7.89 (m, 2H), 7.78 – 7.74 (m, 1H), 7.60 – 7.50 (m, 3H), 7.48 (d, *J* = 8.9 Hz, 1H), 6.85 (s, 1H).

^{13}C NMR (100 MHz, Chloroform-*d*) δ 177.6, 164.2, 155.2, 137.1, 132.2, 131.3, 129.3, 128.6, 126.5, 125.2, 120.2, 118.9, 107.5.

Data in accordance with those reported in ref. [2].

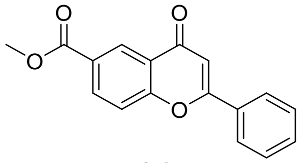

4ah

methyl 4-oxo-2-phenyl-4*H*-chromene-6-carboxylate

Purified by flash chromatography on silica gel eluting with petroleum ether /ethyl acetate = 20/1 to 5/1. Yield: 71%; white solid; m.p.: 180 - 182 ºC.

^{1}H NMR (400 MHz, Chloroform-*d*) δ 8.90 (d, *J* = 2.1 Hz, 1H), 8.39 – 8.35 (m, 2H), 7.93 – 7.91 (m, 2H), 7.64 (d, *J* = 8.8 Hz, 1H), 7.58 – 7.51 (m, 3H), 7.01 (d, *J* = 8.5 Hz, 1H), 3.95 (s, 3H).

^{13}C NMR (101 MHz, Chloroform-*d*) δ 178.9, 166.0, 164.8, 158.8, 135.0, 132.0, 131.9, 129.3, 128.3, 127.6, 126.6, 123.9, 118.8, 107.4, 52.1.

Data in accordance with those reported in ref. [7].

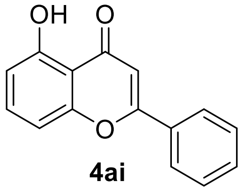

4ai

5-hydroxy-2-phenyl-4*H*-chromen-4-one

Purified by flash chromatography on silica gel eluting with petroleum ether /ethyl acetate = 20/1 to 5/1. Yield: 61%; yellow solid; m.p.: 160-162 ºC.

^{1}H NMR (400 MHz, Chloroform-*d*) δ 7.90 (d, *J* = 8.1 Hz, 2H), 7.54 (t, *J* = 8.2 Hz, 4H), 6.99 (d, *J* = 8.4 Hz, 1H), 6.80 (d, *J* = 8.2 Hz, 1H), 6.73 (s, 1H).

^{13}C NMR (100 MHz, Chloroform-*d*) δ 183.7, 164.7, 160.8, 156.5, 135.5, 132.2, 131.2, 129.2, 126.5, 111.5, 110.9, 107.2, 106.1.

Data in accordance with those reported in ref. [8].

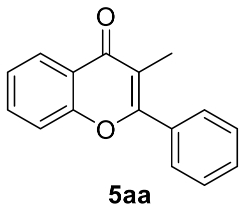

5aa

3-methyl-2-phenyl-4*H*-chromen-4-one

Purified by flash chromatography on silica gel eluting with petroleum ether /ethyl acetate = 20/1 to 5/1. Yield: 76%; white solid; m.p.: 103-105 °C.

^{1}H NMR (400 MHz, Chloroform-*d*) δ 8.28 – 8.26 (m, 1H), 7.68 – 7.63 (m, 3H), 7.55 – 7.52 (m, 3H), 7.48 – 7.45 (m, 1H), 7.43 – 7.39 (m, 1H), 2.18 (s, 3H).

^{13}C NMR (100 MHz, Chloroform-*d*) δ 179.2, 161.3, 156.3, 133.6, 130.4, 129.1, 128.6, 126.0, 124.9, 122.6, 118.0, 117.7, 12.0.

Data in accordance with those reported in ref. [2].

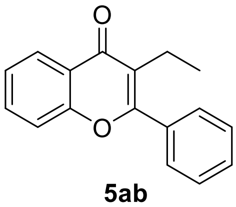

5ab

3-ethyl-2-phenyl-4*H*-chromen-4-one

Purified by flash chromatography on silica gel eluting with petroleum ether /ethyl acetate = 20/1 to 5/1. Yield: 75%; white solid; m.p.: 87-88 °C.

^{1}H NMR (400 MHz, Chloroform-*d*) δ 8.26 (m, 1H), 7.68 – 7.60 (m, 3H), 7.56 – 7.51 (m, 3H), 7.45 – 7.43 (m, 1H), 7.42 – 7.37 (m, 1H), 2.61 – 2.56 (m, 2H), 1.22 – 1.19 (m, 3H).

^{13}C NMR (100 MHz, Chloroform-*d*) δ 178.6, 161.7, 156.3, 133.7, 133.5, 130.3, 128.7, 126.0, 124.8, 123.6, 123.1, 118.0, 19.4, 14.0.

Data in accordance with those reported in ref. [9].

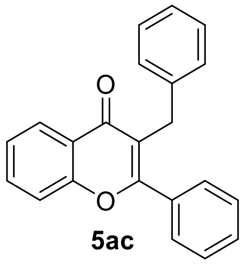

5ac

3-benzyl-2-phenyl-4*H*-chromen-4-one

Purified by flash chromatography on silica gel eluting with petroleum ether /ethyl acetate = 20/1 to 5/1. Yield: 75%; white solid; m.p.: 82-83 °C.

^{1}H NMR (400 MHz, Chloroform-*d*) δ 8.28 – 8.25 (m, 1H), 7.69 – 7.65 (m, 1H), 7.61 – 7.36 (m, 7H), 7.24 – 7.07 (m, 5H), 3.98 (s, 2H).

^{13}C NMR (101 MHz, Chloroform-*d*) δ 178.4, 163.1, 156.3, 140.3, 133.7, 133.4, 130.5, 128.8, 128.7, 128.5, 128.2, 126.3, 126.1, 125.0, 123.2, 120.7, 118.1, 31.37.

Data in accordance with those reported in ref. [10].

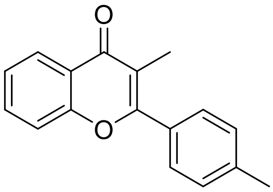

5ia

3-methyl-2-(*p*-tolyl)-4*H*-chromen-4-one

Purified by flash chromatography on silica gel eluting with petroleum ether /ethyl acetate = 20/1 to 5/1. Yield: 68%; white solid; m.p.: 125 - 127 °C.

^{1}H NMR (400 MHz, Chloroform-*d*) δ 8.41 (dd, *J* = 8.0, 1.4 Hz, 1H), 7.83 – 7.75 (m, 1H), 7.70 (d, *J* = 8.1 Hz, 2H), 7.60 (d, *J* = 8.4 Hz, 1H), 7.54 (d, *J* = 7.8 Hz, 1H), 7.48 (d, *J* = 8.0 Hz, 2H), 2.60 (s, 3H), 2.33 (s, 3H).

^{13}C NMR (100 MHz, Chloroform-*d*) δ 178.99, 161.28, 156.16, 130.65, 129.15, 128.92, 125.90, 124.40, 123.51, 122.52, 117.30, 22.73, 21.52.

HRMS (ESI): Calcd. For $C_{17}H_{15}O_2$ $[M+H]^+$: 251.1067, Found: 251.1070.

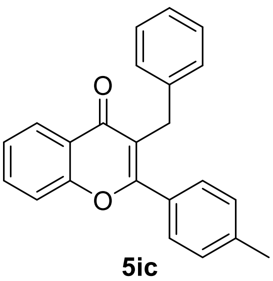

5ic

3-benzyl-2-(*p*-tolyl)-4*H*-chromen-4-one

Purified by flash chromatography on silica gel eluting with petroleum ether /ethyl acetate = 20/1 to 5/1. Yield: 68%; white solid; m.p.: 92 - 93 °C.

^{1}H NMR (400 MHz, Chloroform-*d*) δ 8.27 – 8.25 (m, 1H), 7.70 – 7.66 (m, 1H), 7.58 – 7.51 (m, 2H), 7.53 – 7.46 (m, 4H), 7.43 – 7.39 (m, 1H), 7.24 – 7.20 (m, 2H), 7.17 – 7.10 (m, 3H), 3.98 (s, 2H).

^{13}C NMR (100 MHz, Chloroform-*d*) δ 178.5, 163.2, 156.3, 140.2, 133.8, 133.3, 130.6, 128.8, 128.7, 128.5, 128.2, 126.3, 126.1, 125.1, 123.1, 120.7, 118.1, 31.4.

HRMS (ESI): Calcd. For $C_{23}H_{19}O_2$ $[M+H]^+$: 327.1379, Found: 327.1378.

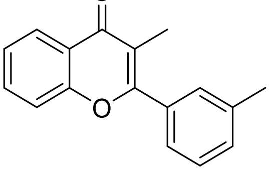

5ea

3-methyl-2-(*m*-tolyl)-4*H*-chromen-4-one

Purified by flash chromatography on silica gel eluting with petroleum ether /ethyl acetate = 20/1 to 5/1. Yield: 79%; white solid; m.p.: 103-105 °C.

^{1}H NMR (400 MHz, Chloroform-*d*) δ 8.26 (d, *J* = 8.0 Hz, 1H), 7.69 – 7.62 (m, 1H), 7.46 (d, *J* = 7.1 Hz, 2H), 7.44 – 7.39 (m, 3H), 7.33 (d, *J* = 6.7 Hz, 1H), 2.46 (s, 3H), 2.17 (s, 3H).

^{13}C NMR (100 MHz, Chloroform-*d*) δ 179.1, 161.4, 138.4, 133.5, 131.1, 129.5, 128.5, 126.3, 126.0, 124.8, 122.6, 118.0, 117.6, 21.6, 12.0.
Data in accordance with those reported in ref. [8].

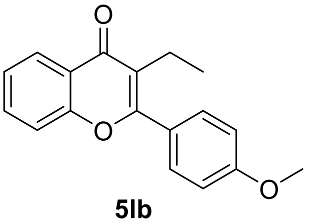

5lb

3-ethyl-2-(4-methoxyphenyl)-4*H*-chromen-4-one

Purified by flash chromatography on silica gel eluting with petroleum ether /ethyl acetate = 20/1 to 5/1. Yield: 65%; white solid; m.p.: 104-105 °C.
^1H NMR (400 MHz, Chloroform-*d*) δ 8.17 (d, *J* = 8.0 Hz, 1H), 7.34 (d, *J* = 8.4 Hz, 1H), 7.28 (d, *J* = 7.2 Hz, 1H), 7.36 – 7.26 (m, 3H), 6.95 (d, *J* = 8.4 Hz, 2H), 3.81 (s, 3H), 2.53 (q, *J* = 7.4 Hz, 2H), 1.20 – 1.30 (m, 3H).
^{13}C NMR (100 MHz, Chloroform-*d*) δ 178.6, 161.6, 161.1, 156.2, 133.3, 130.2, 126.0, 124.7, 123.1, 118.0, 114.0, 55.5, 19.5, 14.0.
HRMS (ESI): Calcd. For $C_{18}H_{17}O_3$ $[M+H]^+$: 281.1172, Found: 281.1170.

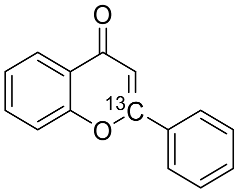

[^{13}C]**6aa**

2-phenyl-4*H*-chromen-4-one-2-^{13}*C*

Purified by flash chromatography on silica gel eluting with petroleum ether /ethyl acetate = 20/1 to 5/1. Yield: 68%; white solid; m.p.: 102-104 °C.
^1H NMR (400 MHz, Chloroform-*d*) δ 8.25 (dd, *J* = 7.9, 1.7 Hz, 1H), 7.96 – 7.93 (m, 2H), 7.75 – 7.68 (m, 1H), 7.60 – 7.52 (m, 4H), 7.46 – 7.42(m, 1H), 6.87 – 6.84 (m, 1H).
^{13}C NMR (101 MHz, Chloroform-*d*) δ 178.7, 163.6, 156.4, 133.9, 131.8, 129.2, 126.4, 125.9, 125.4, 124.1, 118.2, 107.7.
HRMS (ESI): Calcd. For $C_{14}{}^{13}CH_{11}O_2$ $[M+H]^+$: 224.0787, Found: 224.0782.

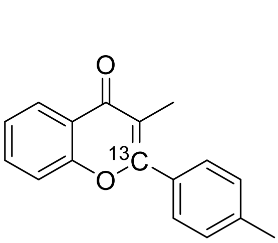

[^{13}C]**6ia**

3-methyl-2-(*p*-tolyl)-4*H*-chromen-4-one-2-^{13}*C*

Purified by flash chromatography on silica gel eluting with petroleum ether /ethyl acetate = 20/1 to 5/1. Yield: 75%; white solid; m.p.: 101-102 °C.

^{1}H NMR (400 MHz, Chloroform-*d*) δ 8.28 – 8.25 (m 1H), 7.68 – 7.63 (m, 1H), 7.57 – 7.54 (m, 2H), 7.46 (d, *J* = 8.4 Hz, 1H), 7.40 (d, *J* = 7.7 Hz, 1H), 7.33 (d, *J* = 7.9 Hz, 2H), 2.45 (s, 3H), 2.19 (s, 3H).

^{13}C NMR (100 MHz, Chloroform-*d*) δ 179.2, 161.5, 156.2, 140.7, 133.5, 129.3, 129.0, 126.0, 125.6, 124.8, 120.6, 118.0, 115.8, 21.6, 12.0.

HRMS (ESI): Calcd. For $C_{16}{}^{13}CH_{15}O_2$ $[M+H]^+$: 252.1100, Found: 252.1107.

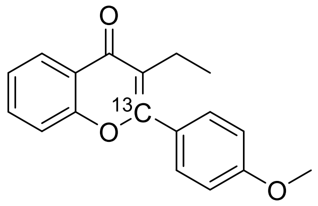

[^{13}C]**6lb**

3-ethyl-2-(4-methoxyphenyl)-4*H*-chromen-4-one-2-^{13}*C*

Purified by flash chromatography on silica gel eluting with petroleum ether /ethyl acetate = 20/1 to 5/1. Yield: 71%; white solid; m.p.: 105-106 °C.

^{1}H NMR (400 MHz, Chloroform-*d*) δ 8.30 – 8.20 (m, 1H), 7.70 – 7.53 (m, 3H), 7.45 – 7.43 (m, 1H), 7.27 – 7.26 (m, 1H), 7.06 – 7.02 (m, 2H), 3.89 (s, 3H), 2.64 – 2.60 (m, 2H), 1.29 – 1.28 (m, 3H).

^{13}C NMR (100 MHz, Chloroform-*d*) δ 178.7, 161.7, 161.1, 156.2, 133.4, 130.2, 126.0, 124.8, 123.3, 118.0, 114.0, 55.6, 19.5, 13.8.

HRMS (ESI): Calcd. For $C_{17}{}^{13}CH_{17}O_3$ $[M+H]^+$: 282.1206, Found: 282.1214.

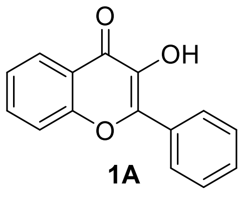

1A

3-hydroxy-2-phenyl-4*H*-chromen-4-one

Purified by flash chromatography on silica gel eluting with petroleum ether /ethyl acetate = 10/1 to 5/1. Yield: 60%; White solid; m.p.: 170-172 °C.

^{1}H NMR (400 MHz, Chloroform-*d*) δ 8.27 – 8.25 (m, 3H), 7.73 -7.70 (m, 1H), 7.59 (d, *J* = 8.5 Hz, 1H), 7.54 (t, *J* = 7.5 Hz, 2H), 7.48 (d, *J* = 7.2 Hz, 1H), 7.41 (t, *J* = 7.5 Hz, 1H), 7.13 (s, 1H).

^{13}C NMR (100 MHz, Chloroform-*d*) δ 173.6, 155.5, 145.1, 138.6, 133.8, 131.2, 130.3, 128.7, 127.9, 125.6, 124.6, 120.8, 118.4.

Data in accordance with those reported in ref. [11].

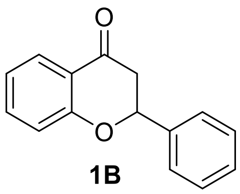

2-phenylchroman-4-one

Purified by flash chromatography on silica gel eluting with petroleum ether /ethyl acetate = 100/1-50/1.

Yield: 95%; yellow solid; m.p.: 77-78 ^{o}C.

^{1}H NMR (400 MHz, Chloroform-*d*) δ 7.95 – 7.92 (m, 1H), 7.56 – 7.36 (m, 6H), 7.07 – 7.04 (m, 2H), 5.50 – 5.46 (m, 1H), 3.13 – 3.05 (m, 1H), 2.91 – 2.86 (m, 1H).

^{13}C NMR (100 MHz, Chloroform-*d*) δ 192.1, 161.6, 138.8, 136.3, 129.0 128.9, 127.1, 126.3, 121.7, 121.0, 118.2, 79.7, 44.8.

Data in accordance with those reported in ref. [11].

6. References

[1] X.-F. Wu, H. Neumann, M. Beller, *Chem. Eur. J.* **2012**, *18*, 12595-12598.

[2] G. Xie, J. Zhan, M. Cai, B. Huang, *Synthesis* **2023**, *55*, 647-656.

[3] K. Cheng, J. Chen, L. Jin, J. Zhou, X. Jiang, C. Yu, *J. Chem. Res.* **2019**, 43, 392-398.

[4] M.-Y. Chang, M.-C. Tsai, C.-Y. Lin, *RSC Adv.* **2021**, *11*, 11655-11662.

[5] a) S. Xu, H. Sun, M. Zhuang, S. Zheng, Y. Jian, W. Zhang, Z. Gao, *Mol. Catal.* **2018**, *452*, 264-270; b) D. Yoshii, X. Jin, T. Yatabe, J.-y. Hasegawa, K. Yamaguchi, N. Mizuno, *Chem. Commun.* **2016**, *52*, 14314-14317.

[6] F. Stanek, M. Stodulski, *Tetrahedron Lett.* **2016**, *57*, 3841-3843.

[7] W. Mansour, M. Fettouhi, B. El Ali, *ACS Omega* **2020**, *5*, 32515-32529.

[8] a) B. S. Jayashree, A. Alam, Y. Nayak, D. V. Kumar, *Med. Chem. Res.* **2012**, *21*,1991 -1996; b)H. Y. Kim, E. Song, K. Oh, *Org. Lett.* **2017**, *19*, 312-315

[9] B. Li, J. Zhu, X. Zheng, W. Ti, Y. Huang, H. Yao, *J. Org. Chem.* **2023**, *88*, 548-558.

[10] X. Zhao, J. Zhou, S. Lin, X. Jin, R. Liu, *Org. Lett.* **2017**, *19*, 976-979.

[11] a) Y.-X. Chen, C.-H. Chang, C.-W. Li, J.-J. Chen, T.-L. Shih, *J. Chin. Chem. Soc.* **2023**, *70*, 1924–1936; b) D. Xia, W. Wang, X. Xu, Y. Zhu, Q. Li, J. Wang, Y. Zhang, W.-D. Zhang, *Chem. Eur. J.* **2024**, *30*, e202402607.

[12] D. C. G. A. Pinto, A. M. S. Silva, J. A. S. Cavaleiro, J. Elguero, *Eur. J. Org. Chem.* **2003**, 747-755.

7. Copies of spectra

2-phenyl-*4H*-chromen-4-one, **4aa**
400 MHz, $CDCl_3$

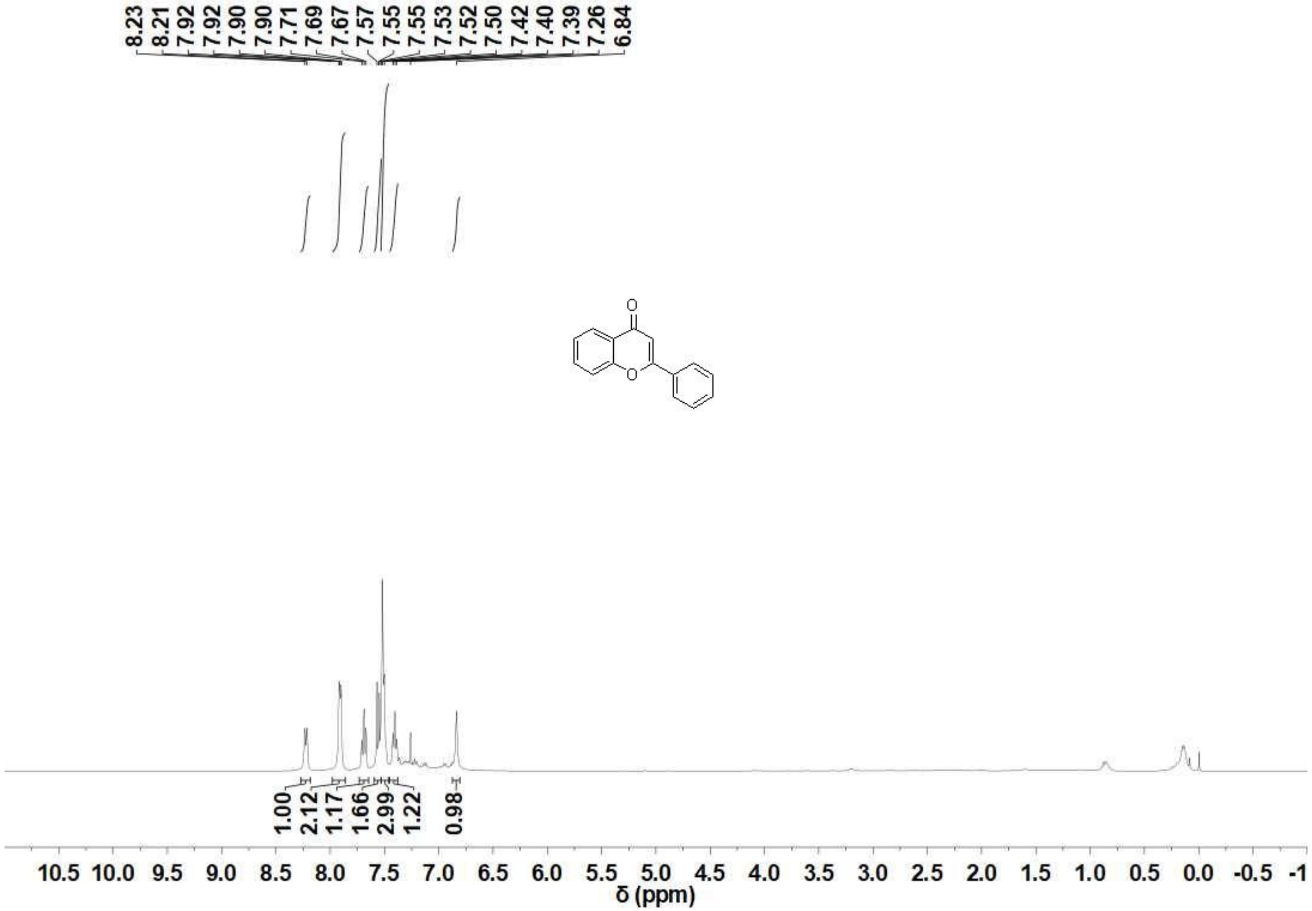

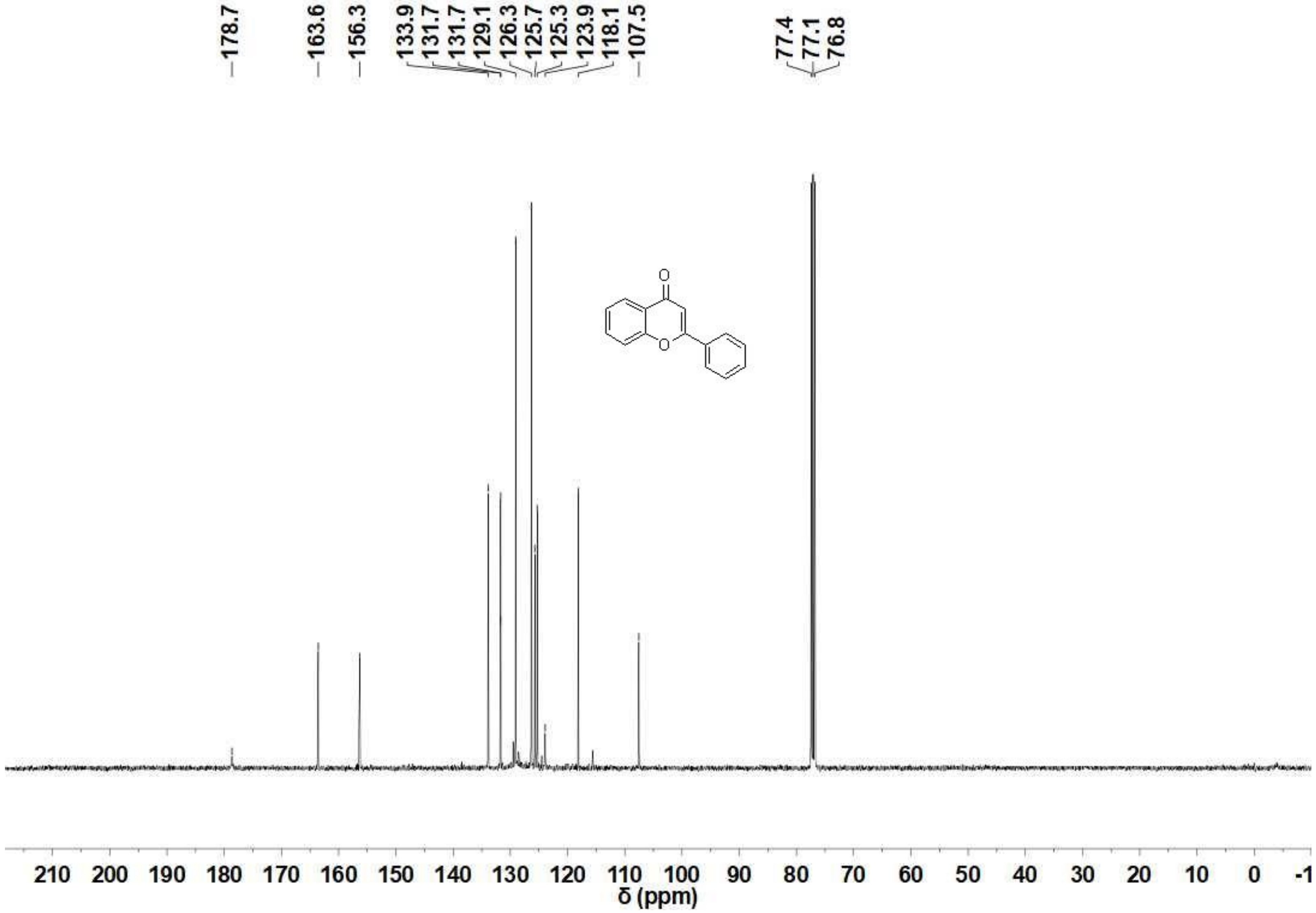
178.7
163.6
156.3
133.9
131.7
131.7
129.1
126.3
125.7
125.3
123.9
118.1
107.5
77.4
77.1
76.8
O
O
210 200 190 180 170 160 150 140 130 120 110 100 90 80 70 60 50 40 30 20 10 0 -1
δ (ppm)

2-(o-tolyl)-*4H*-chromen-4-one, **4ba**
400 MHz, $CDCl_3$

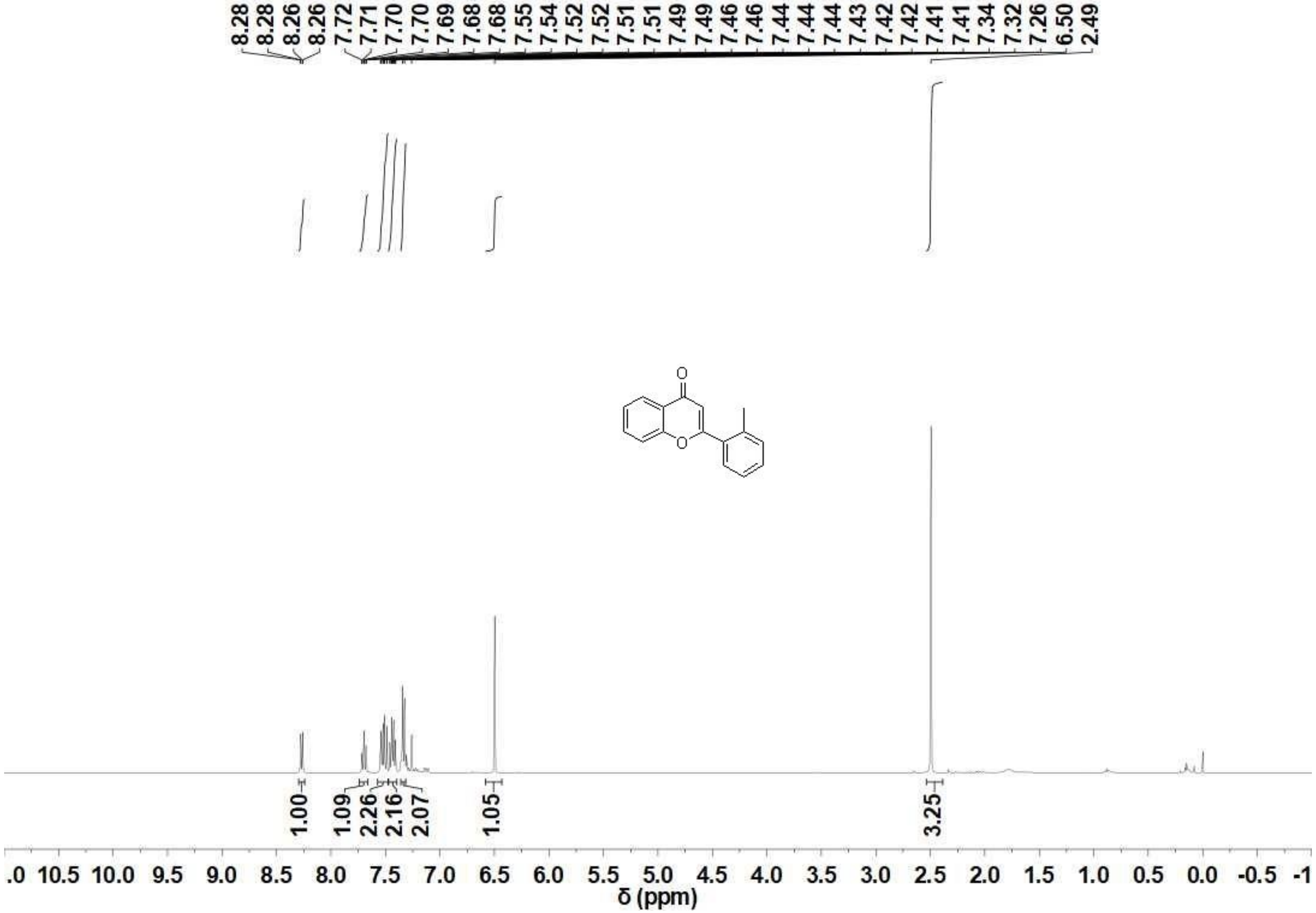

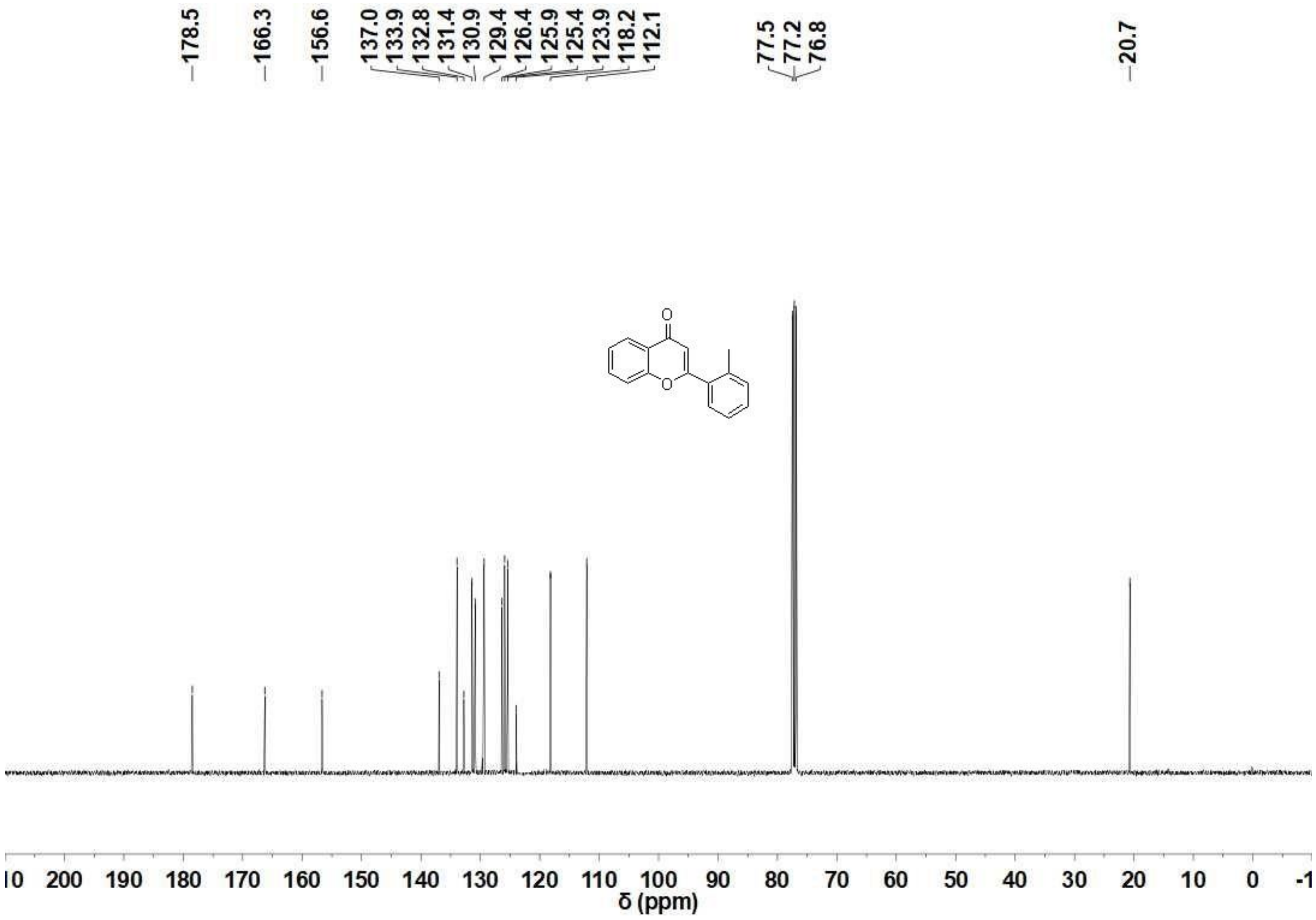
178.5
166.3
156.6
137.0
133.9
132.8
131.4
130.9
129.4
126.4
125.9
125.4
123.9
118.2
112.1
77.5
77.2
76.8
20.7
O
O
δ (ppm)

2-(2-methoxyphenyl)-*4H*-chromen-4-one, **4ca**
400 MHz, $CDCl_3$

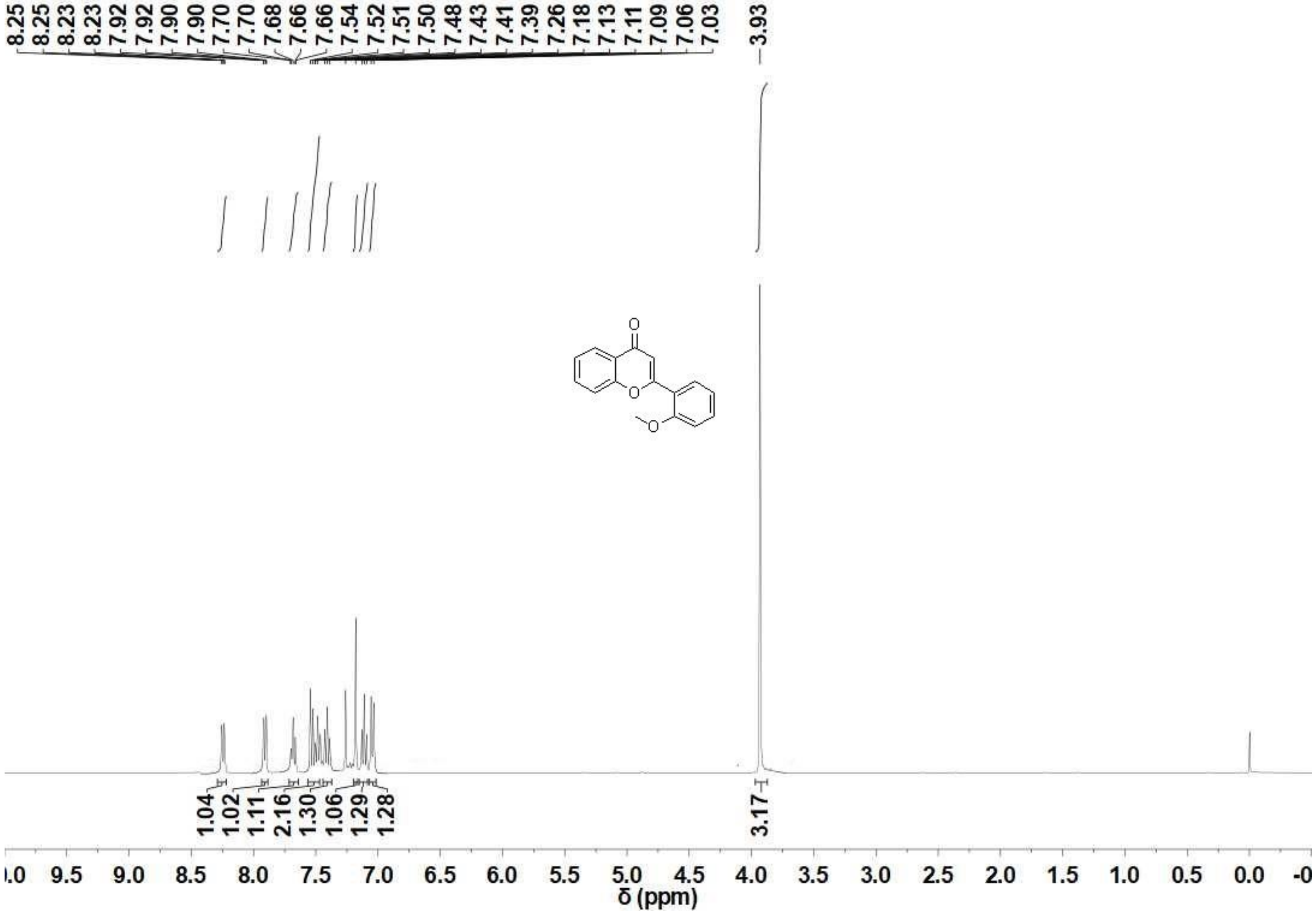

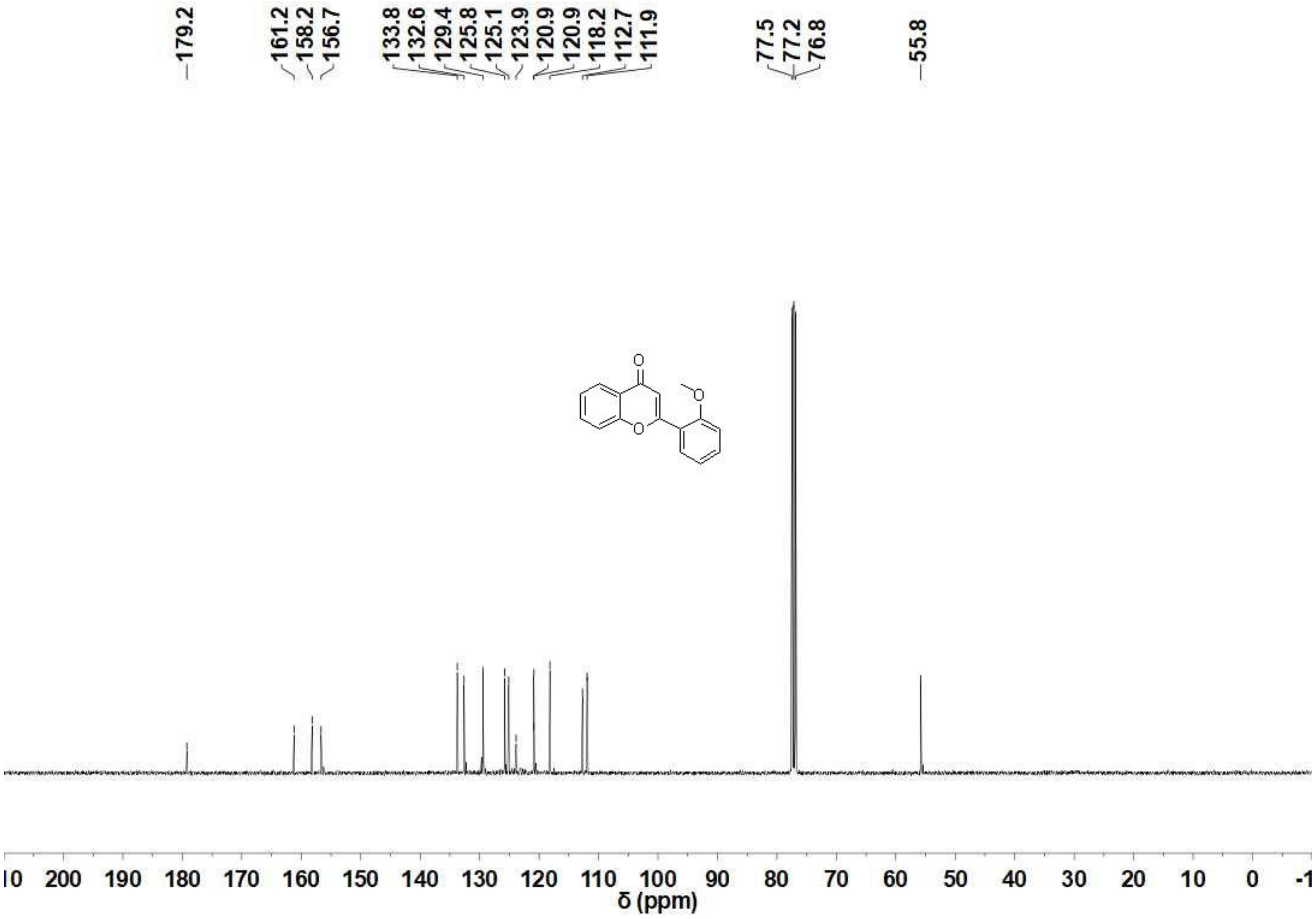

179.2
161.2
158.2
156.7
133.8
132.6
129.4
125.8
125.1
123.9
120.9
120.9
118.2
112.7
111.9
77.5
77.2
76.8
55.8
O
O
O
200 190 180 170 160 150 140 130 120 110 100 90 80 70 60 50 40 30 20 10 0
δ (ppm)

2-(2-chlorophenyl)-*4H*-chromen-4-one, **4da**

400 MHz, $CDCl_3$

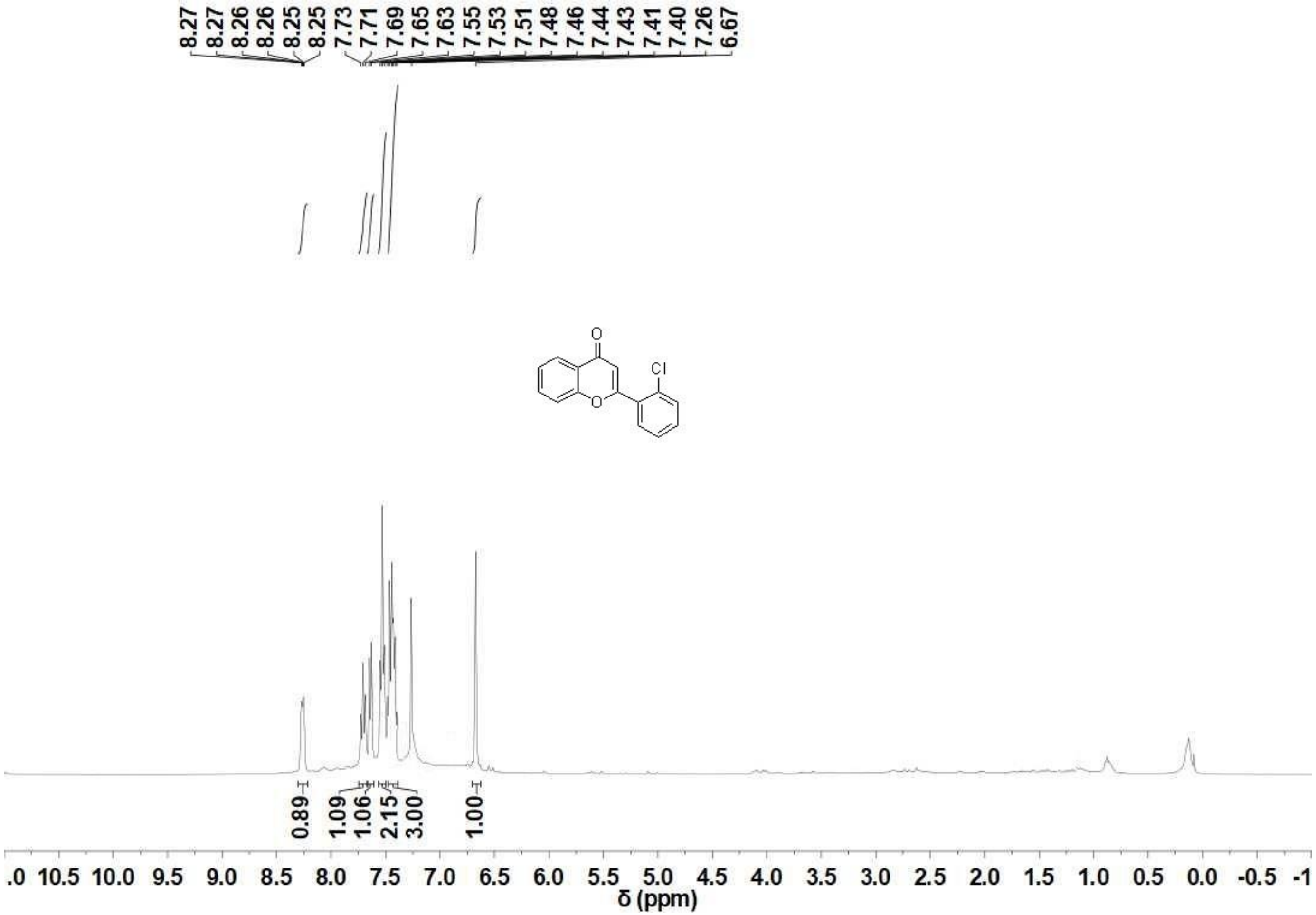

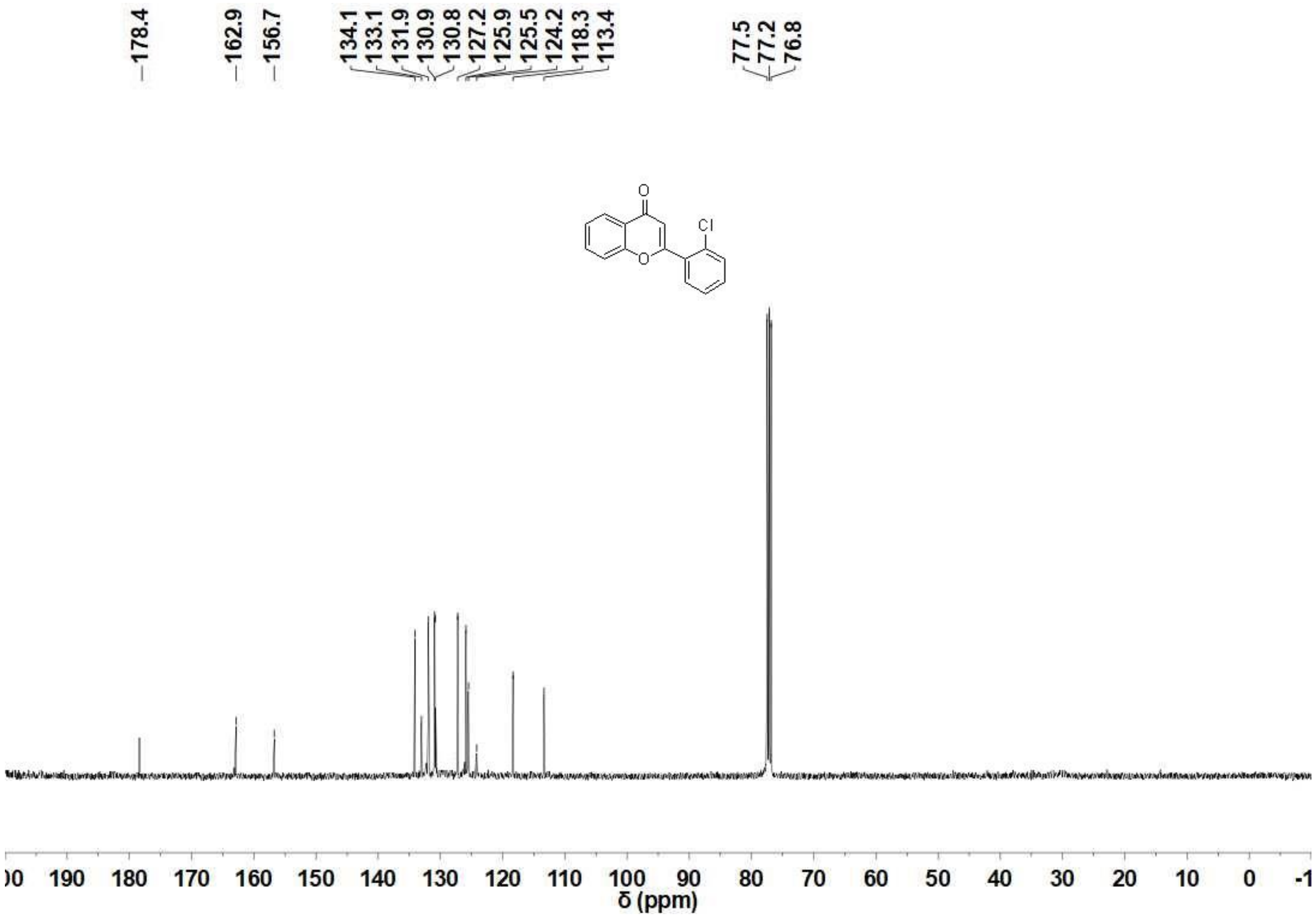
178.4
162.9
156.7
134.1
133.1
131.9
130.9
130.8
127.2
125.9
125.5
124.2
118.3
113.4
77.5
77.2
76.8
O
Cl
O
0 190 180 170 160 150 140 130 120 110 100 90 80 70 60 50 40 30 20 10 0 -1
δ (ppm)

2-(*m*-tolyl)-*4H*-chromen-4-one, **4ea**
400 MHz, $CDCl_3$

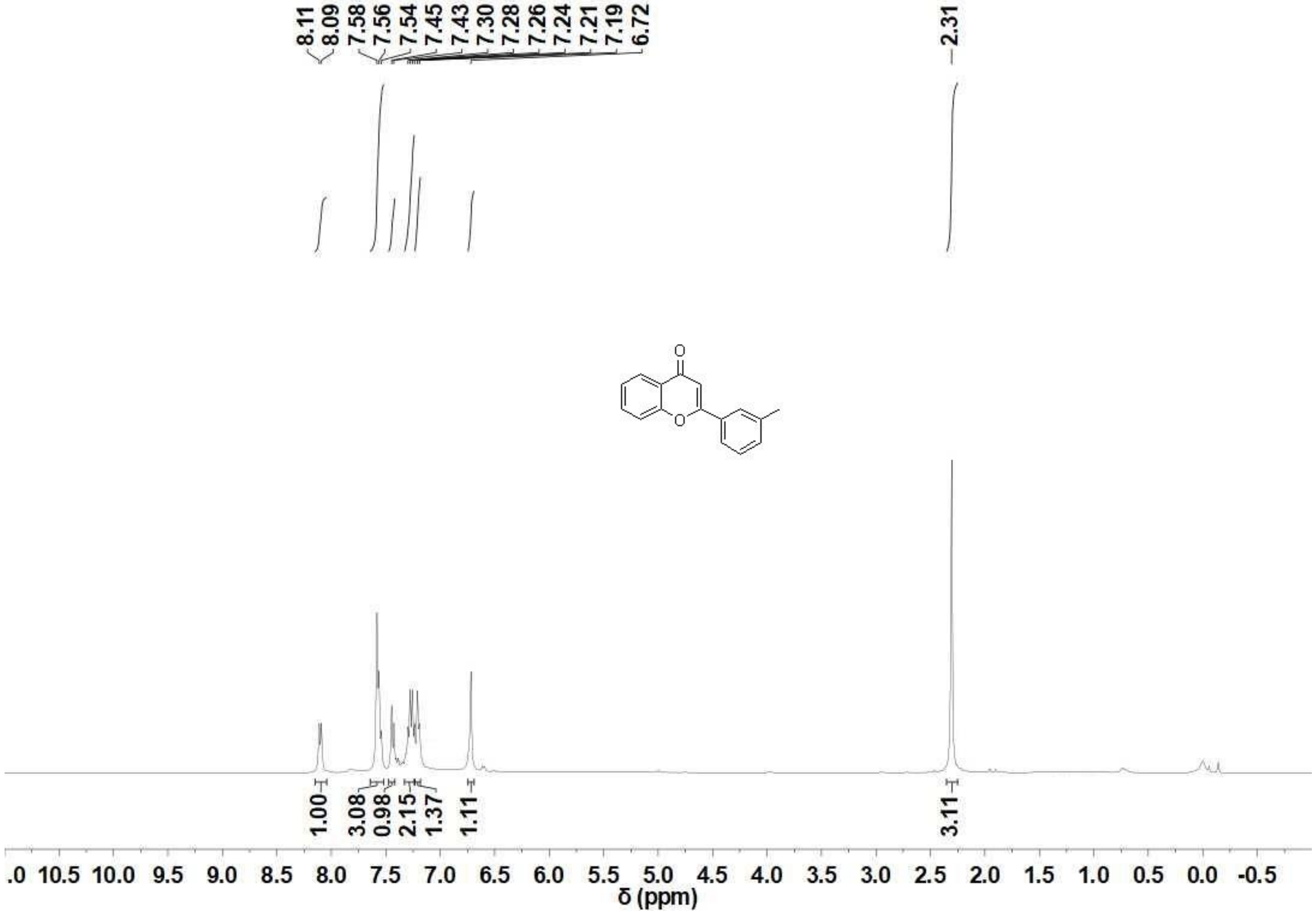

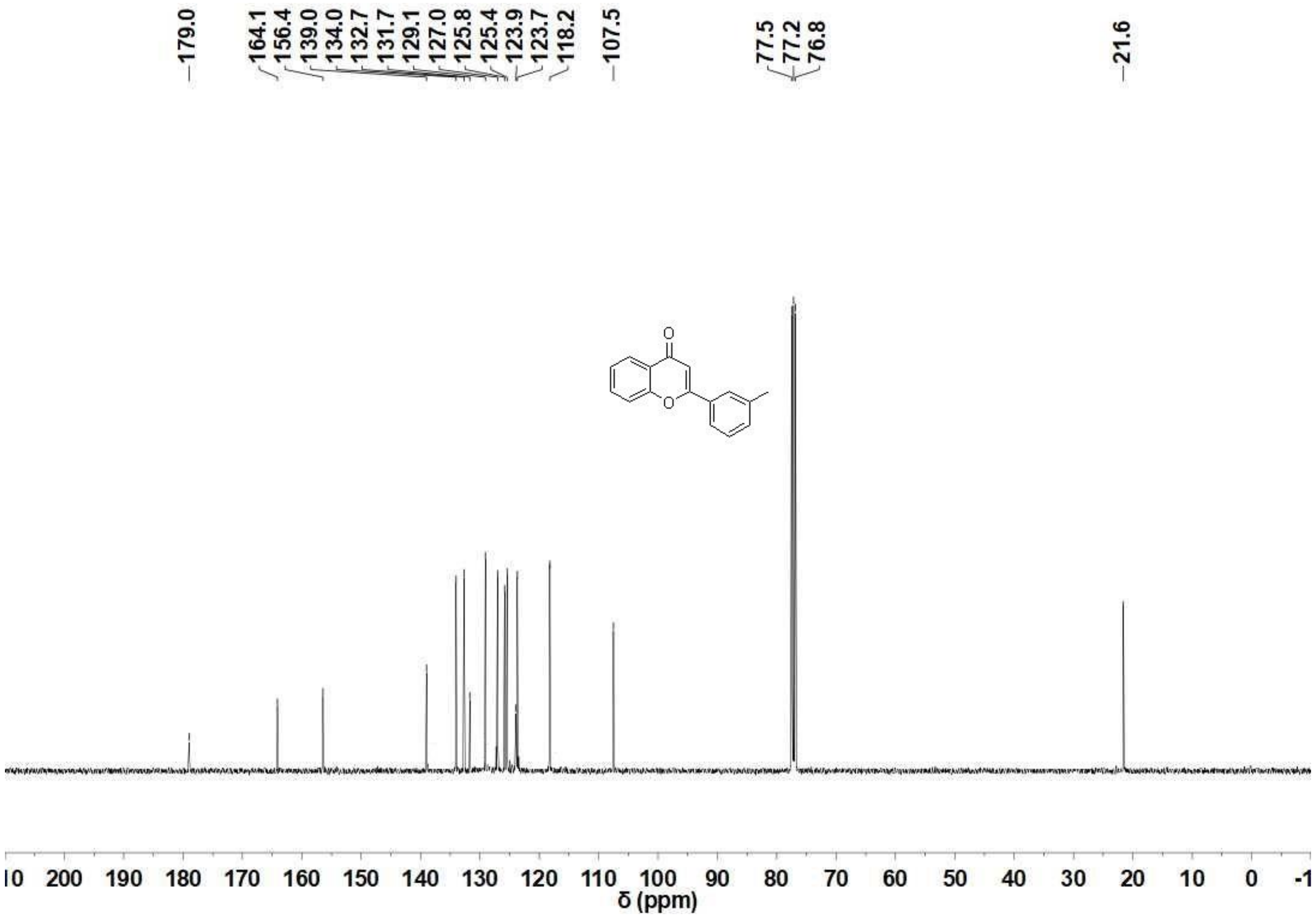
179.0
164.1
156.4
139.0
134.0
132.7
131.7
129.1
127.0
125.8
125.4
123.9
123.7
118.2
107.5
77.5
77.2
76.8
21.6
10 200 190 180 170 160 150 140 130 120 110 100 90 80 70 60 50 40 30 20 10 0 -1
δ (ppm)

2-(3-methoxyphenyl)-*4H*-chromen-4-one, **4fa**

400 MHz, $CDCl_3$

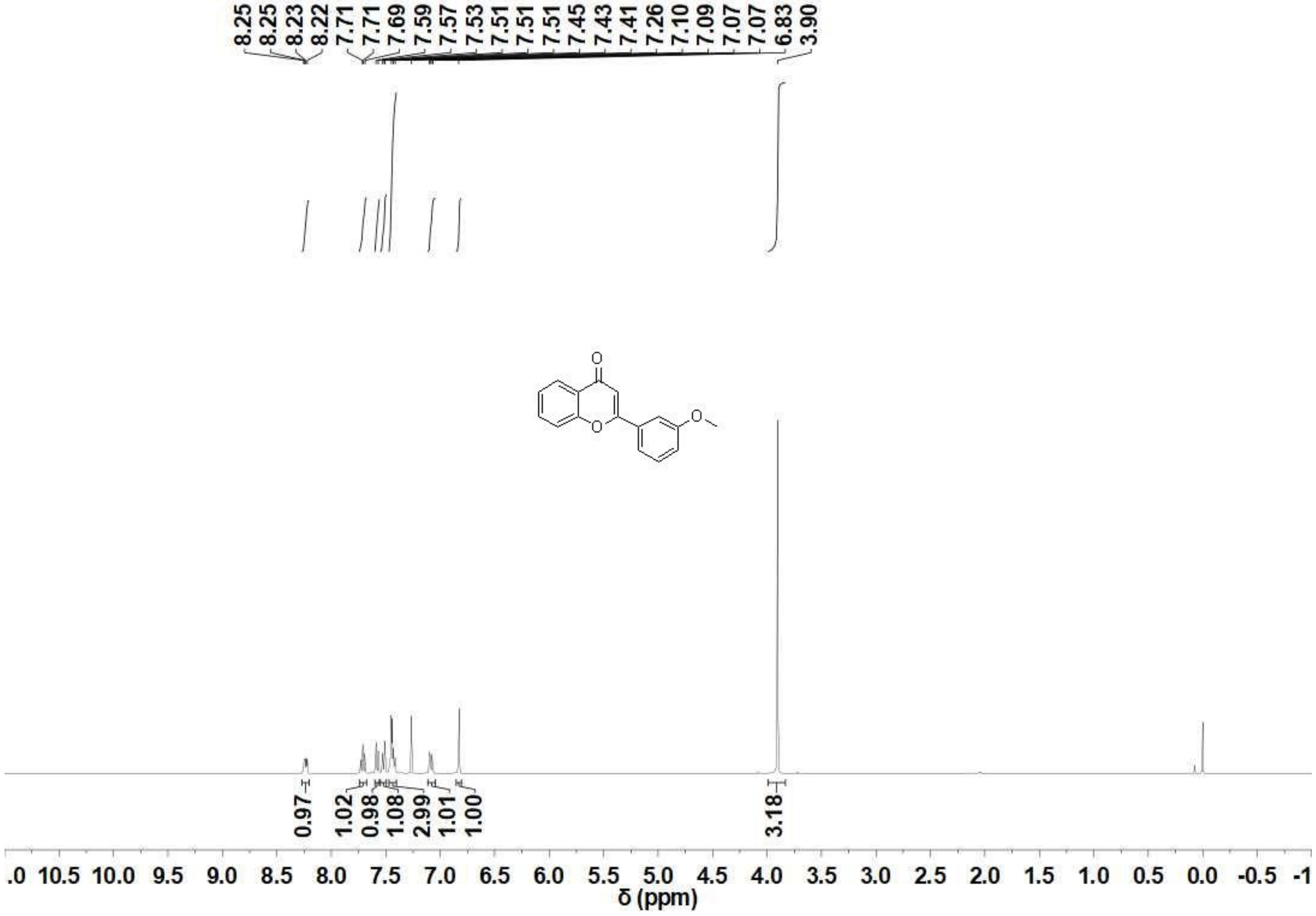

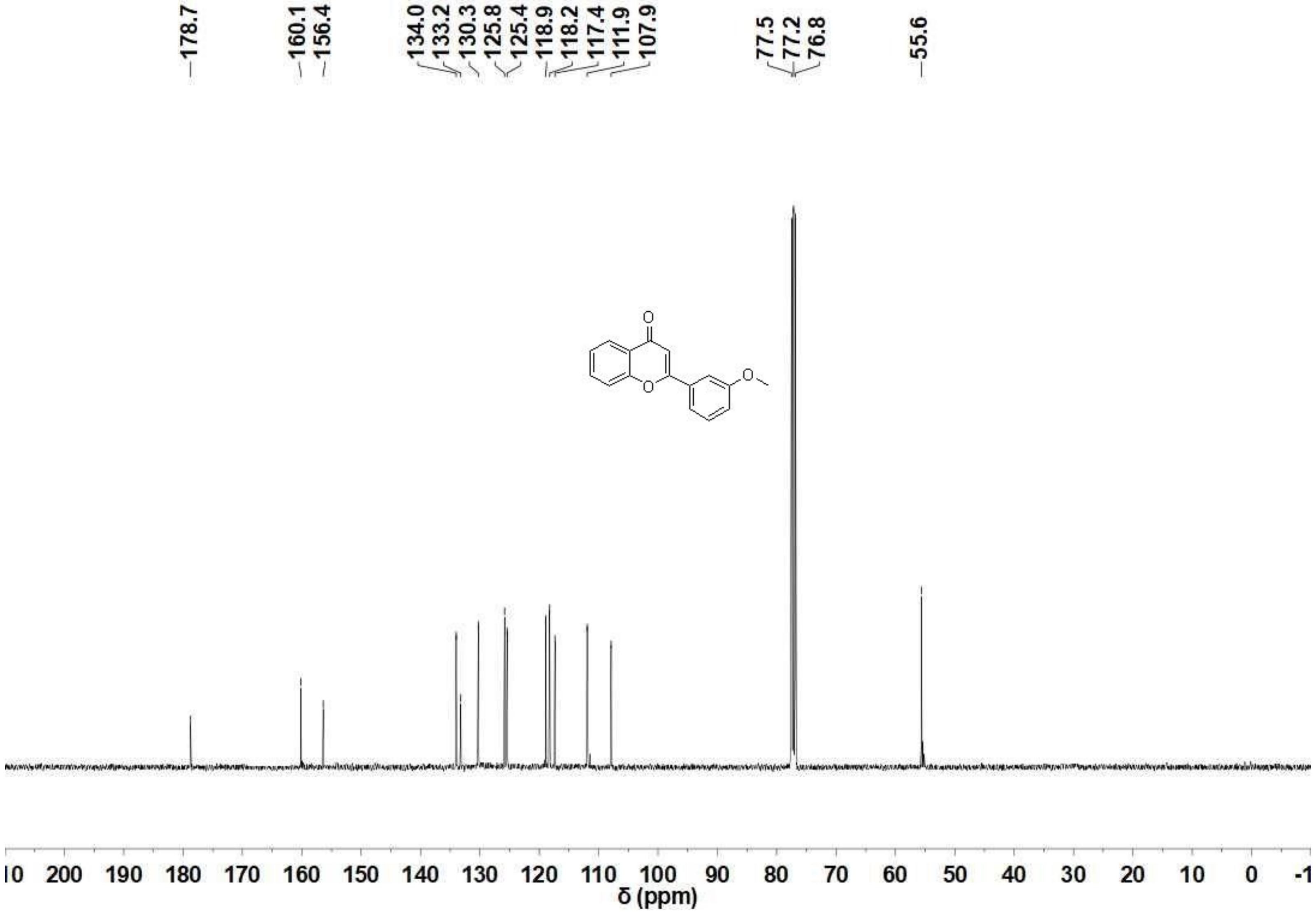
178.7
160.1
156.4
134.0
133.2
130.3
125.8
125.4
118.9
118.2
117.4
111.9
107.9
77.5
77.2
76.8
55.6
O
O
O
10 200 190 180 170 160 150 140 130 120 110 100 90 80 70 60 50 40 30 20 10 0 -1
δ (ppm)

2-(3-fluorophenyl)-*4H*-chromen-4-one, **4ga**

400 MHz, $CDCl_3$

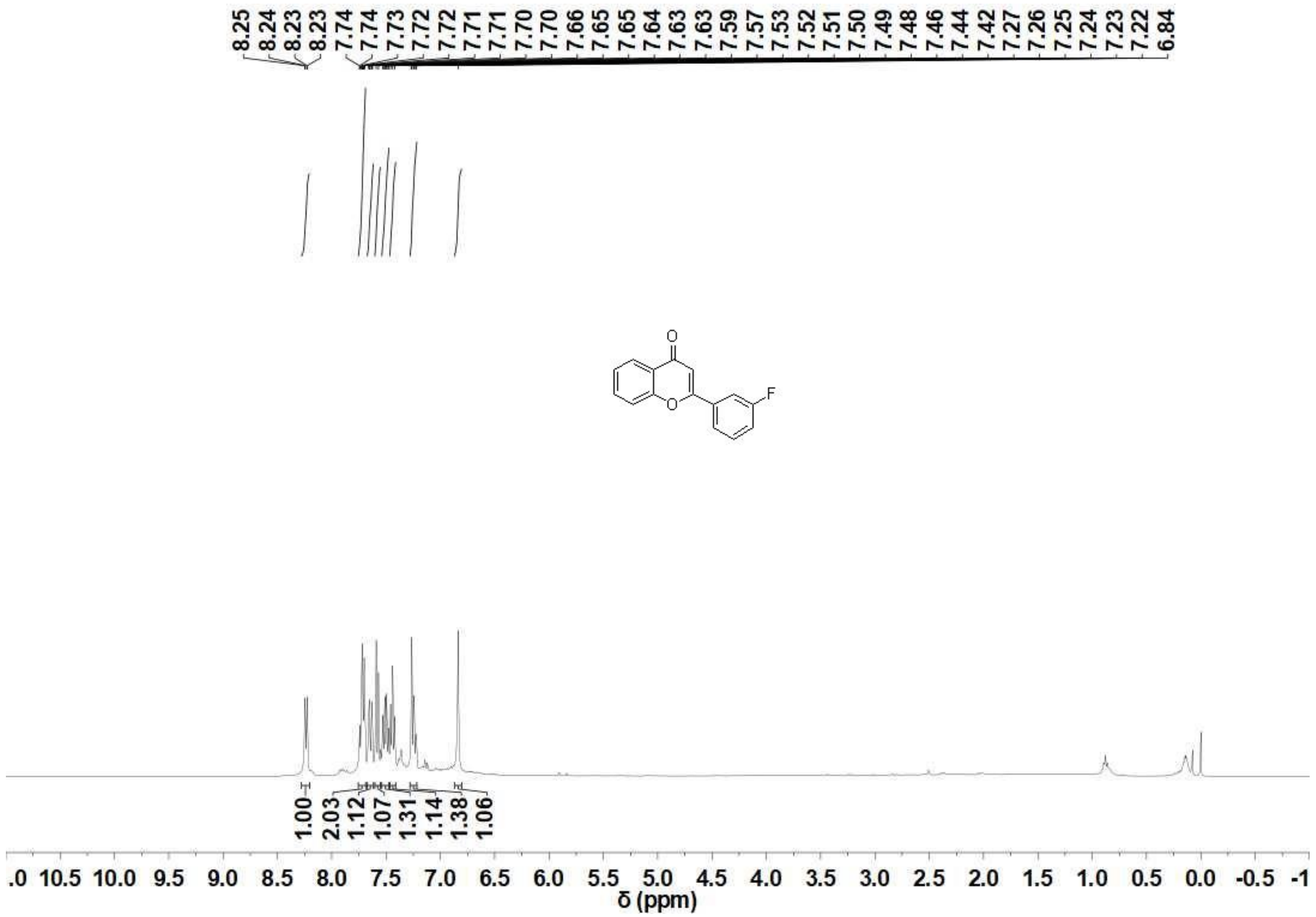

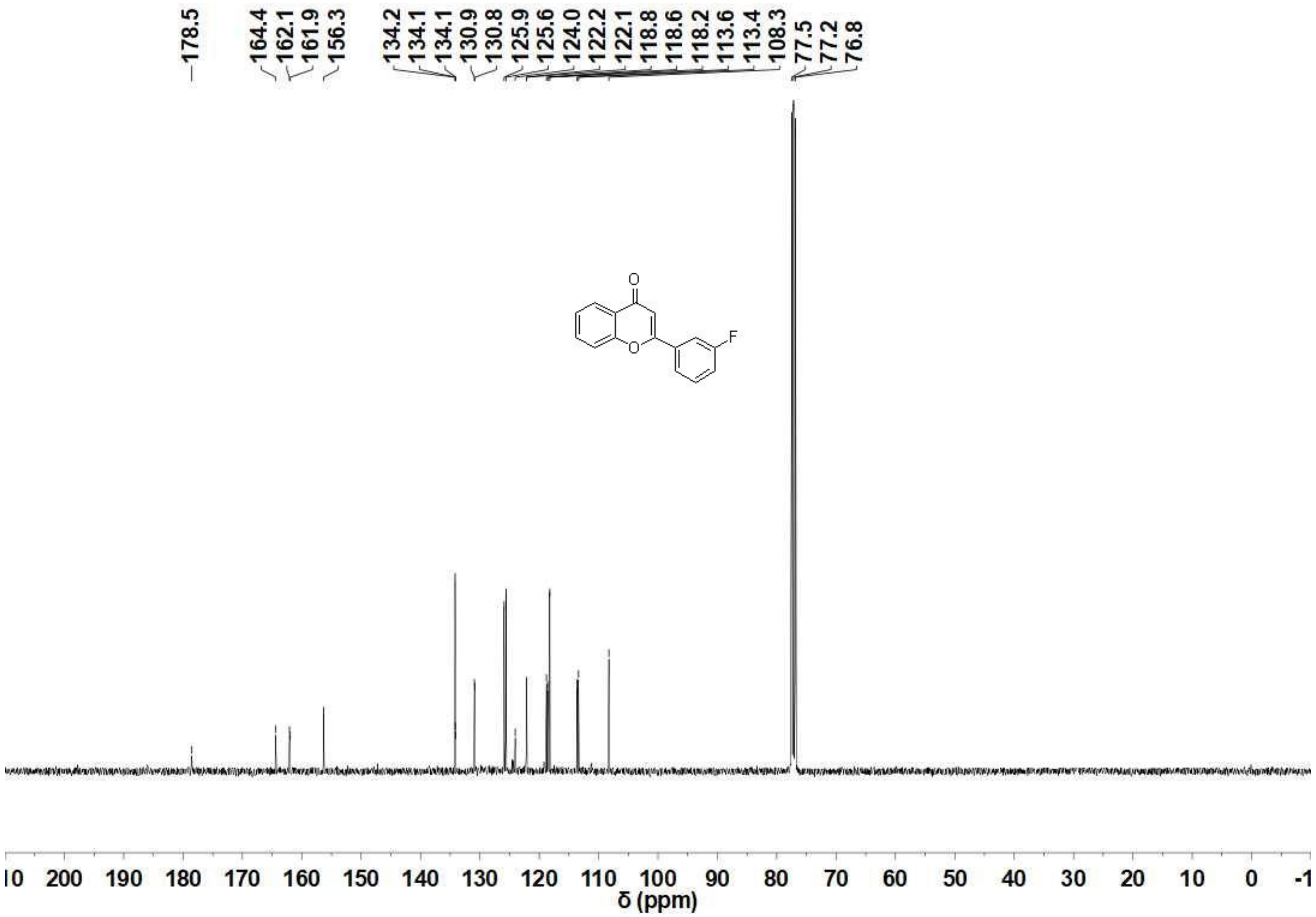
178.5
164.4
162.1
161.9
156.3
134.2
134.1
134.1
130.9
130.8
125.9
125.6
124.0
122.2
122.1
118.8
118.6
118.2
113.6
113.4
108.3
77.5
77.2
76.8
O
O
F
10 200 190 180 170 160 150 140 130 120 110 100 90 80 70 60 50 40 30 20 10 0 -1
δ (ppm)

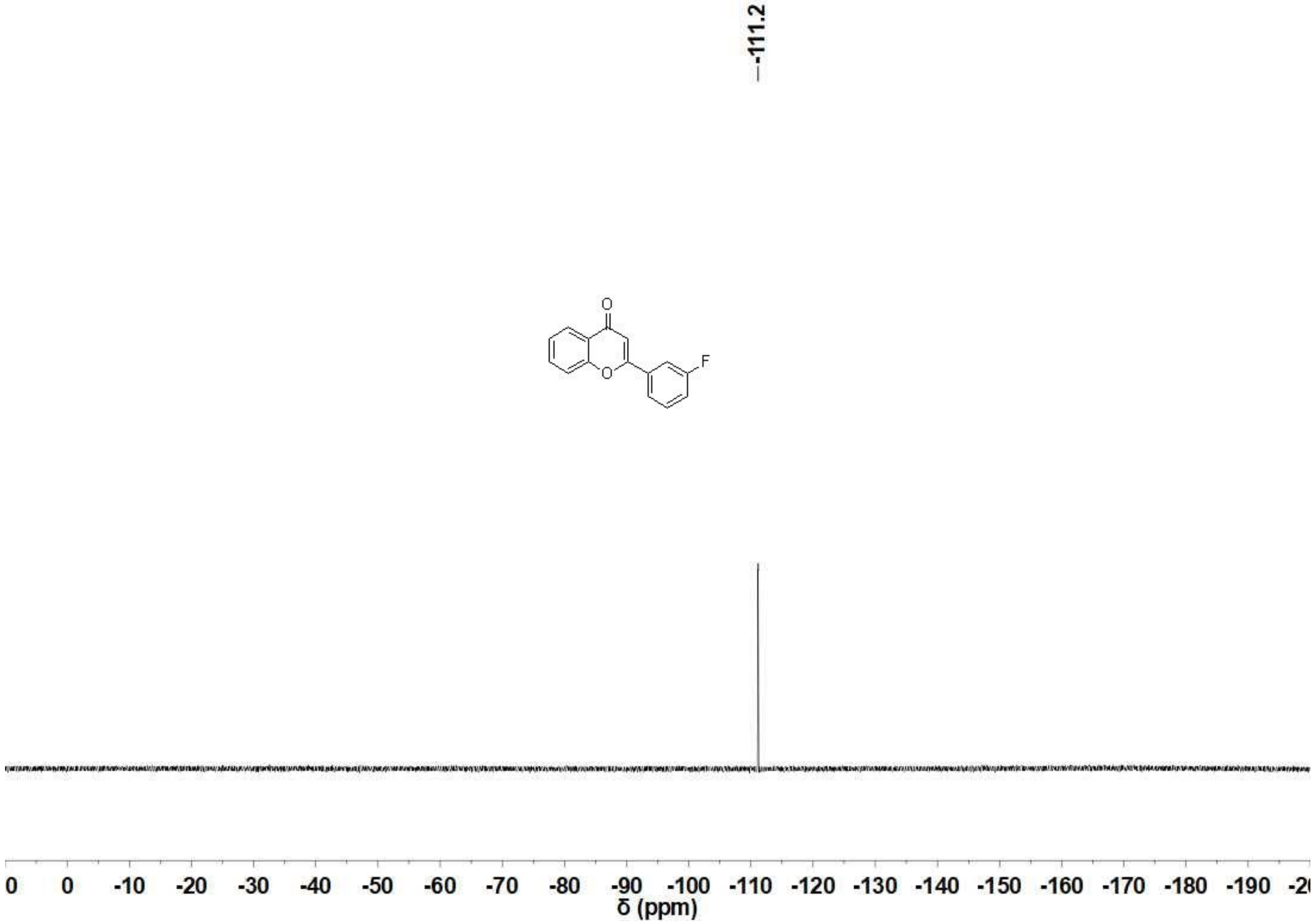
-111.2
O
F
0 0 -10 -20 -30 -40 -50 -60 -70 -80 -90 -100 -110 -120 -130 -140 -150 -160 -170 -180 -190
δ (ppm)

2-(3-chlorophenyl)-*4H*-chromen-4-one, **4ha**

400 MHz, $CDCl_3$

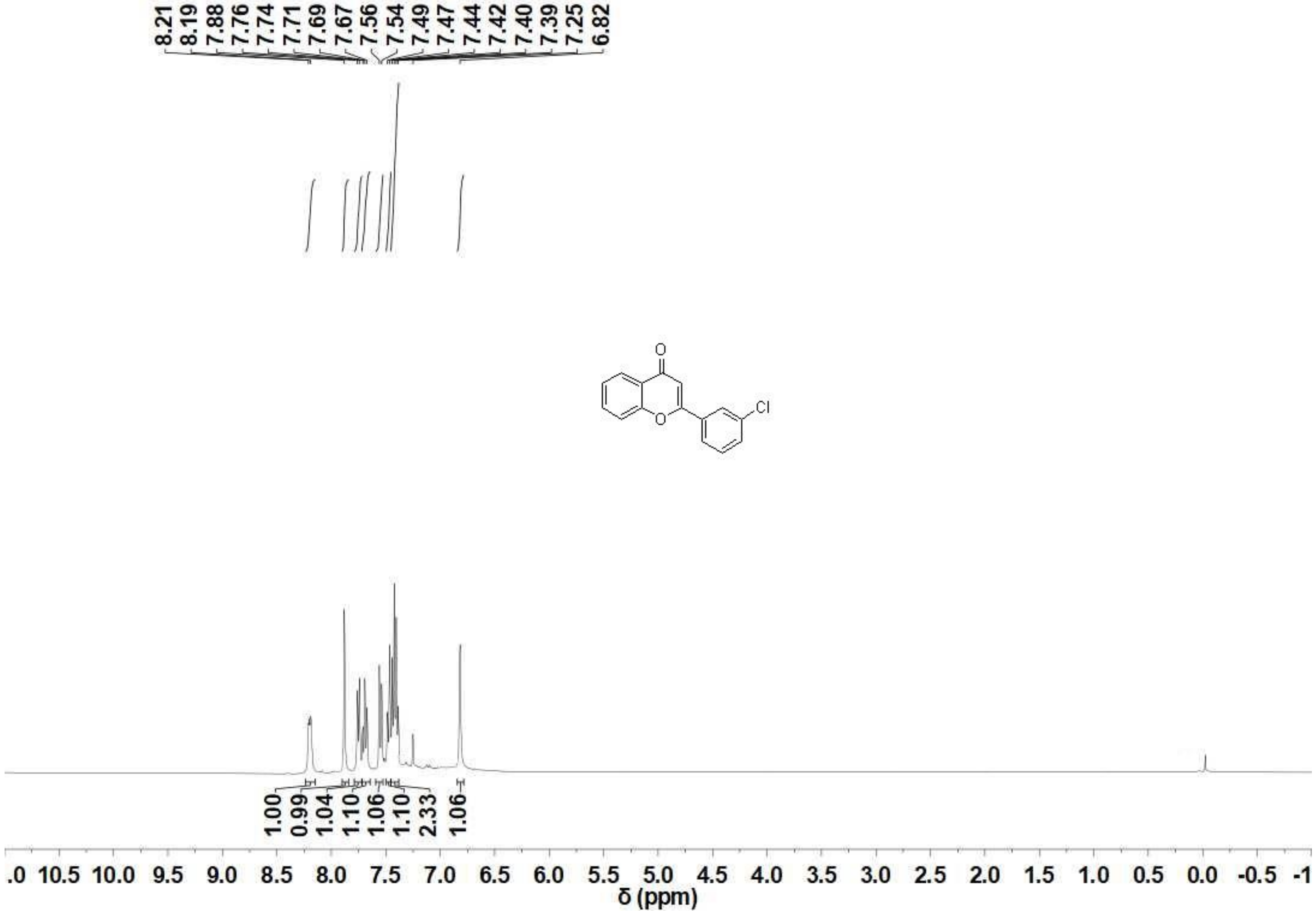

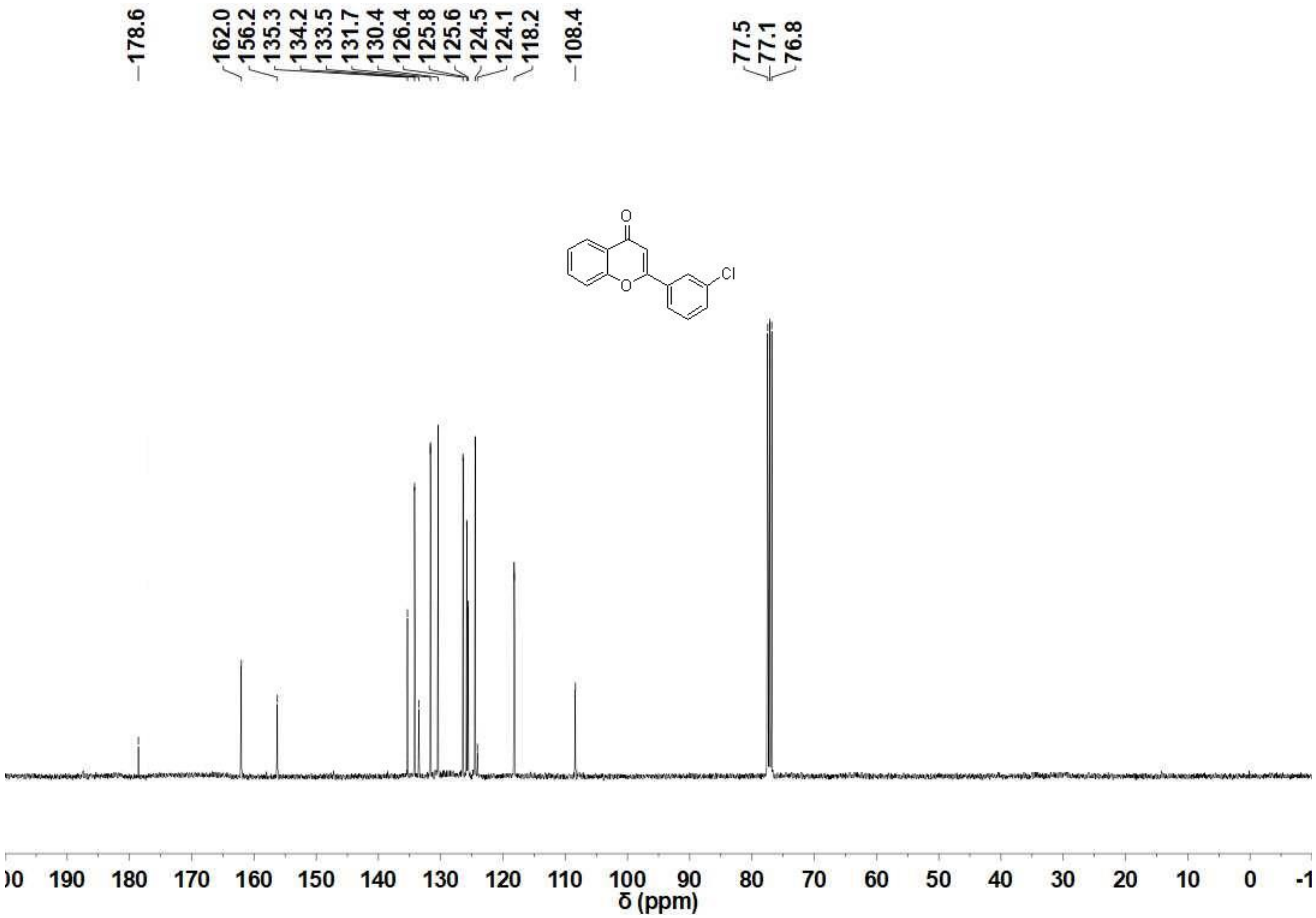

178.6
162.0
156.2
135.3
134.2
133.5
131.7
130.4
126.4
125.8
125.6
124.5
124.1
118.2
108.4
77.5
77.1
76.8
O
O
Cl
00
190
180
170
160
150
140
130
120
110
100
90
80
70
60
50
40
30
20
10
0
-1
δ (ppm)

2-(p-tolyl)-$4H$-chromen-4-one, **4ia**

400 MHz, $CDCl_3$

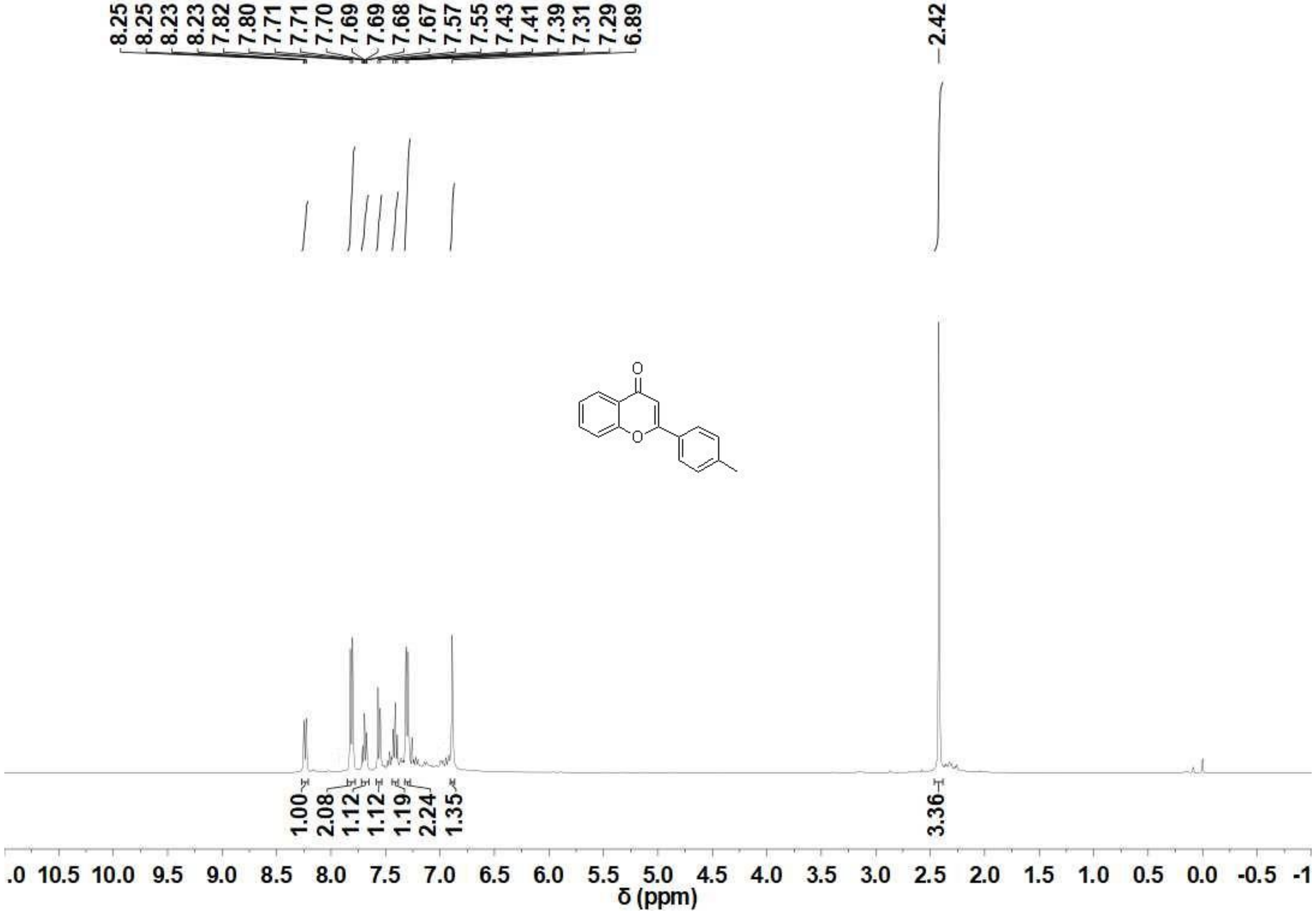

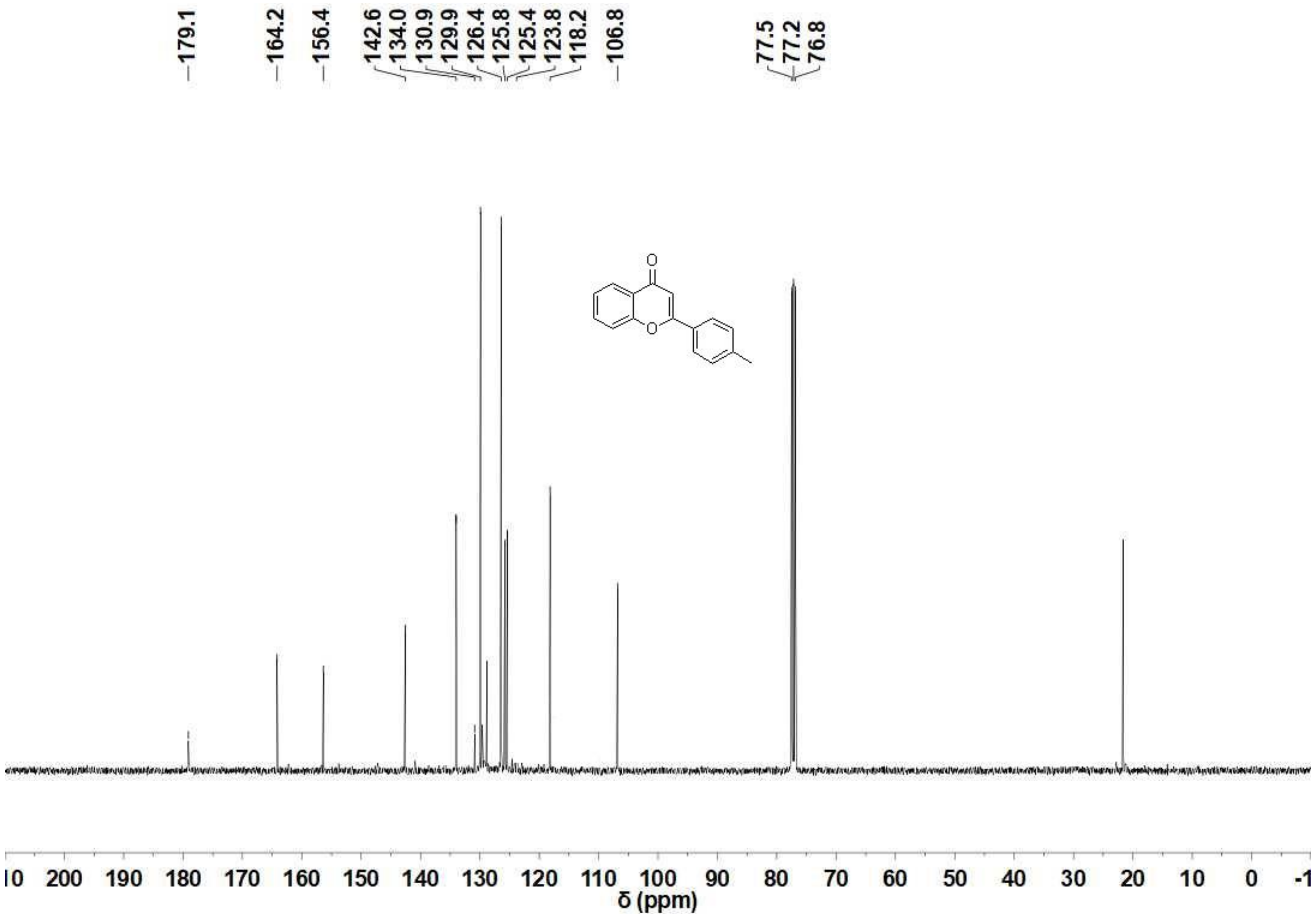
179.1
164.2
156.4
142.6
134.0
130.9
129.9
126.4
125.8
125.4
123.8
118.2
106.8
77.5
77.2
76.8
O
O
10 200 190 180 170 160 150 140 130 120 110 100 90 80 70 60 50 40 30 20 10 0 -1
δ (ppm)

2-(4-butylphenyl)-*4H*-chromen-4-one, **4ja**

400 MHz, $CDCl_3$

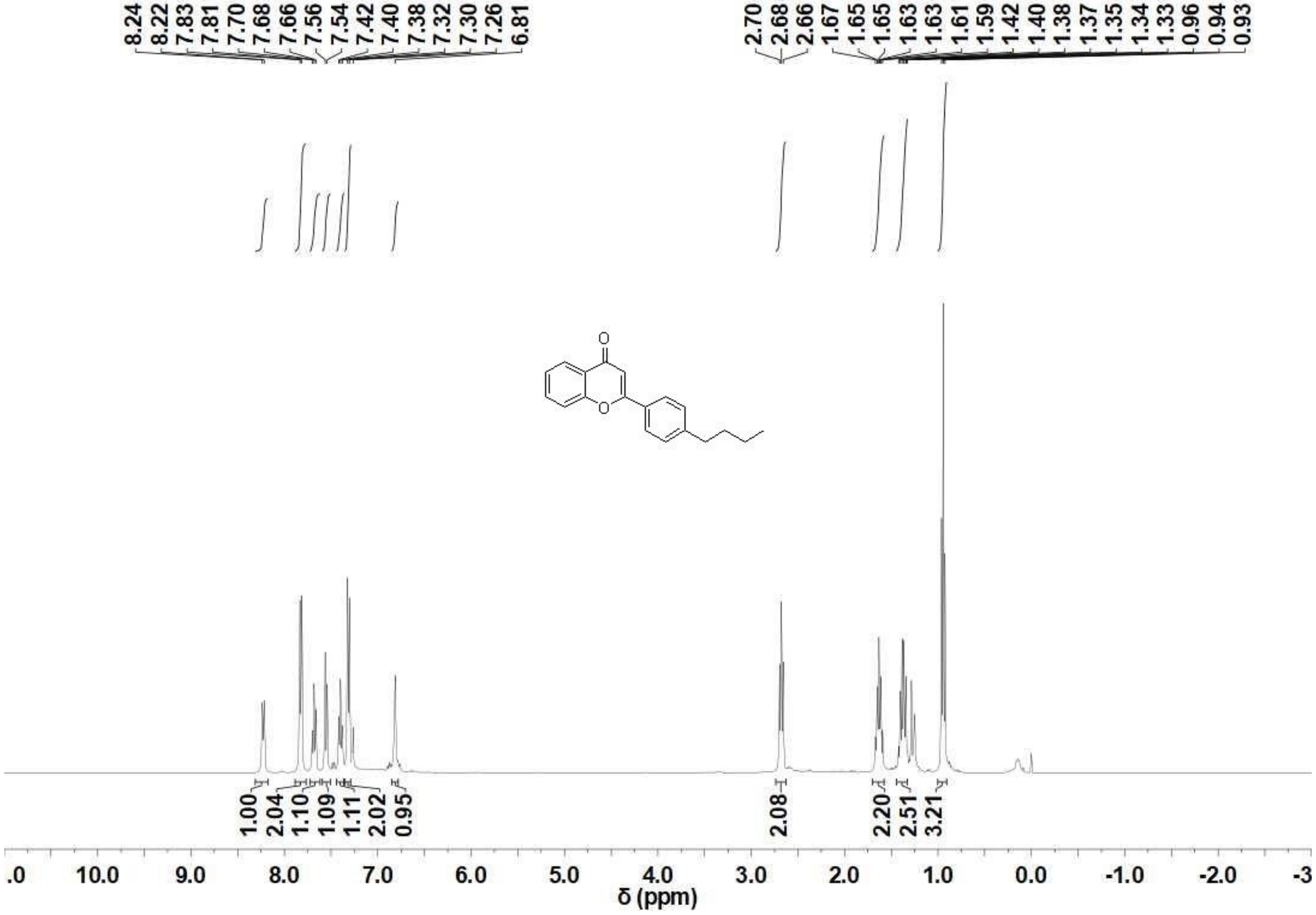

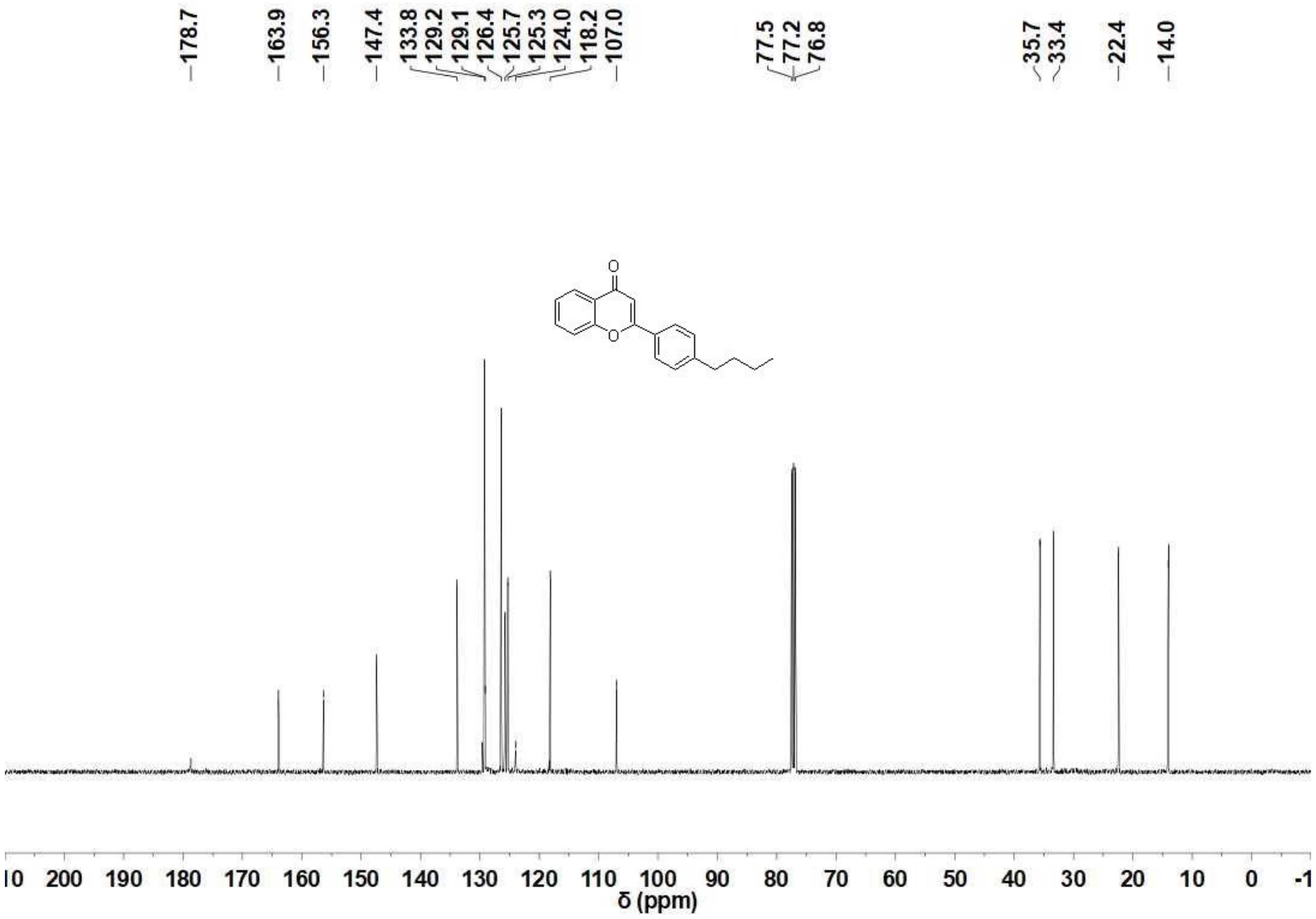
178.7
163.9
156.3
147.4
133.8
129.2
129.1
126.4
125.7
125.3
124.0
118.2
107.0
77.5
77.2
76.8
35.7
33.4
22.4
14.0
O
O
10 200 190 180 170 160 150 140 130 120 110 100 90 80 70 60 50 40 30 20 10 0 -1
δ (ppm)

2-(4-(*tert*-butyl)phenyl)-*4H*-chromen-4-one, **4ka**
400 MHz, $CDCl_3$

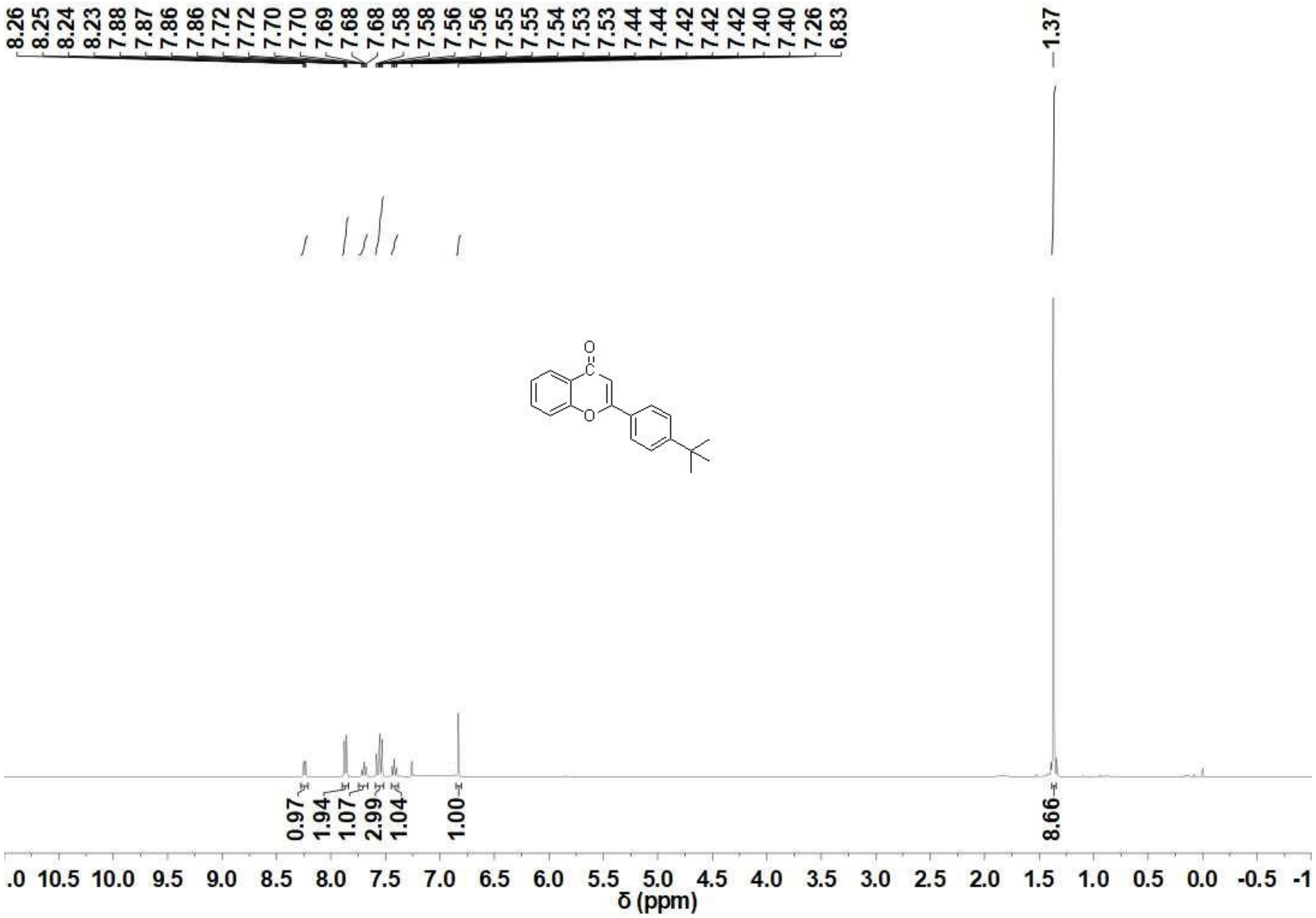

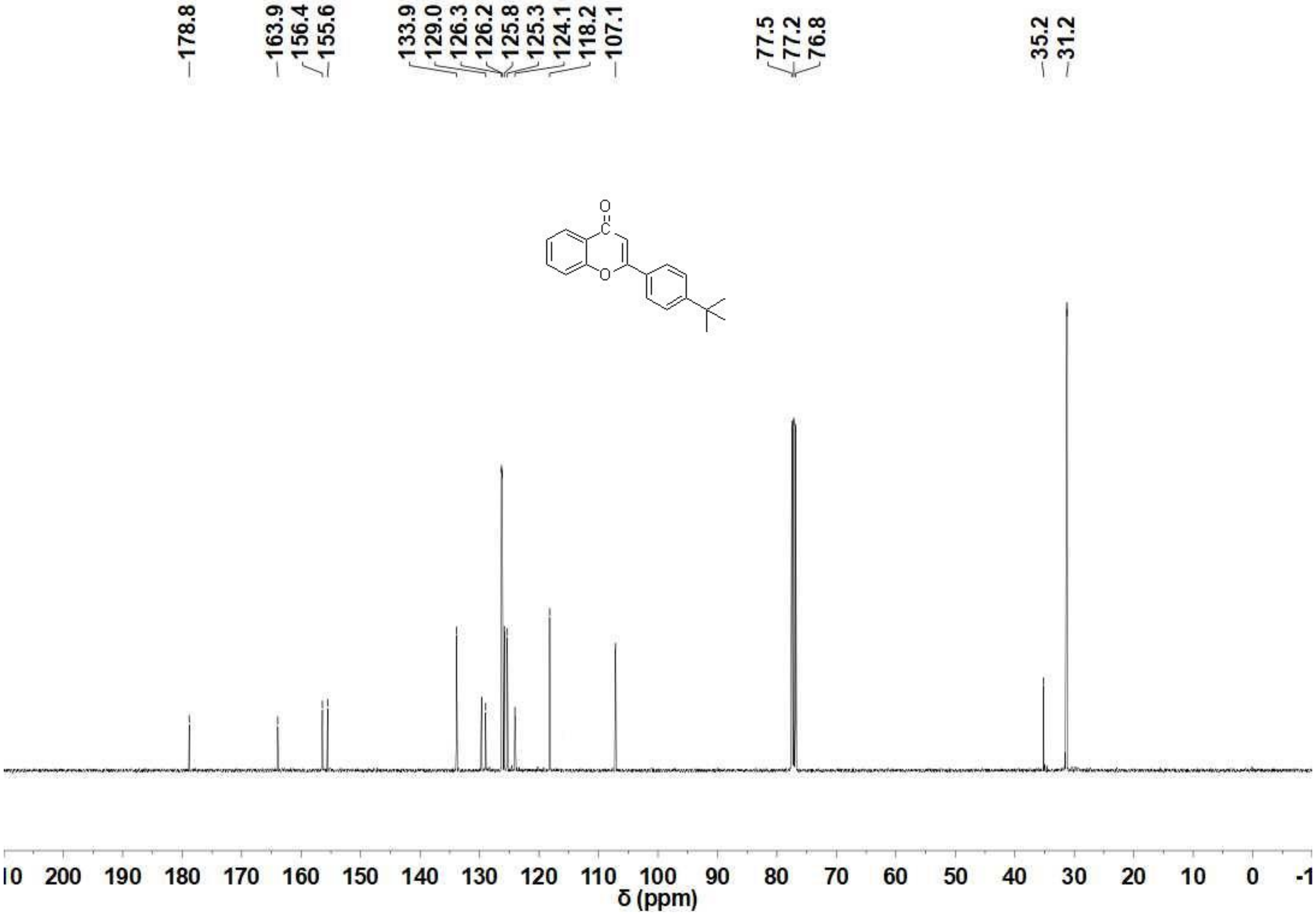
178.8
163.9
156.4
155.6
133.9
129.0
126.3
126.2
125.8
125.3
124.1
118.2
107.1
77.5
77.2
76.8
35.2
31.2
O
O
10 200 190 180 170 160 150 140 130 120 110 100 90 80 70 60 50 40 30 20 10 0 -1
δ (ppm)

2-(4-methoxyphenyl)-*4H*-chromen-4-one, **4la**

400 MHz, $CDCl_3$

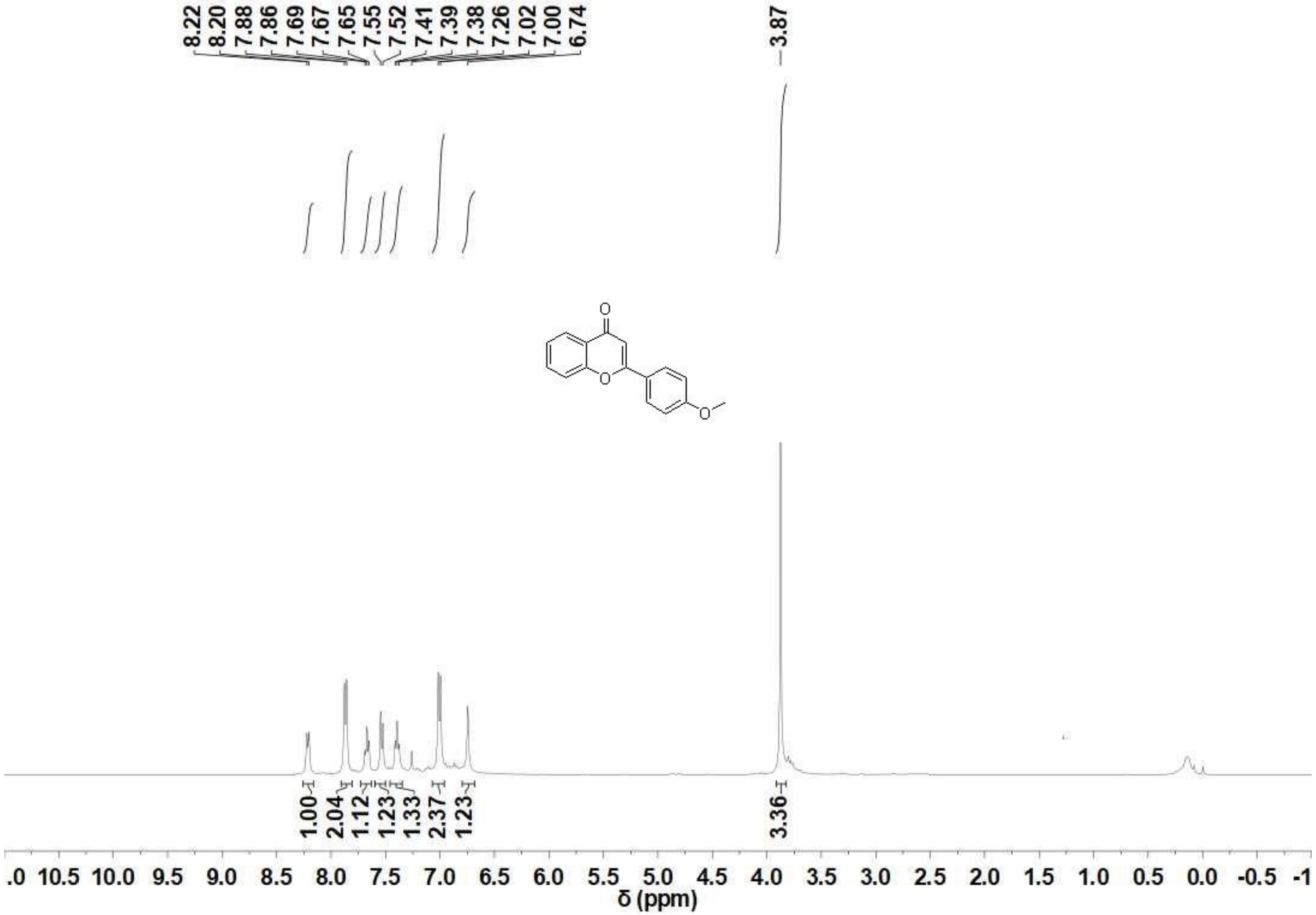

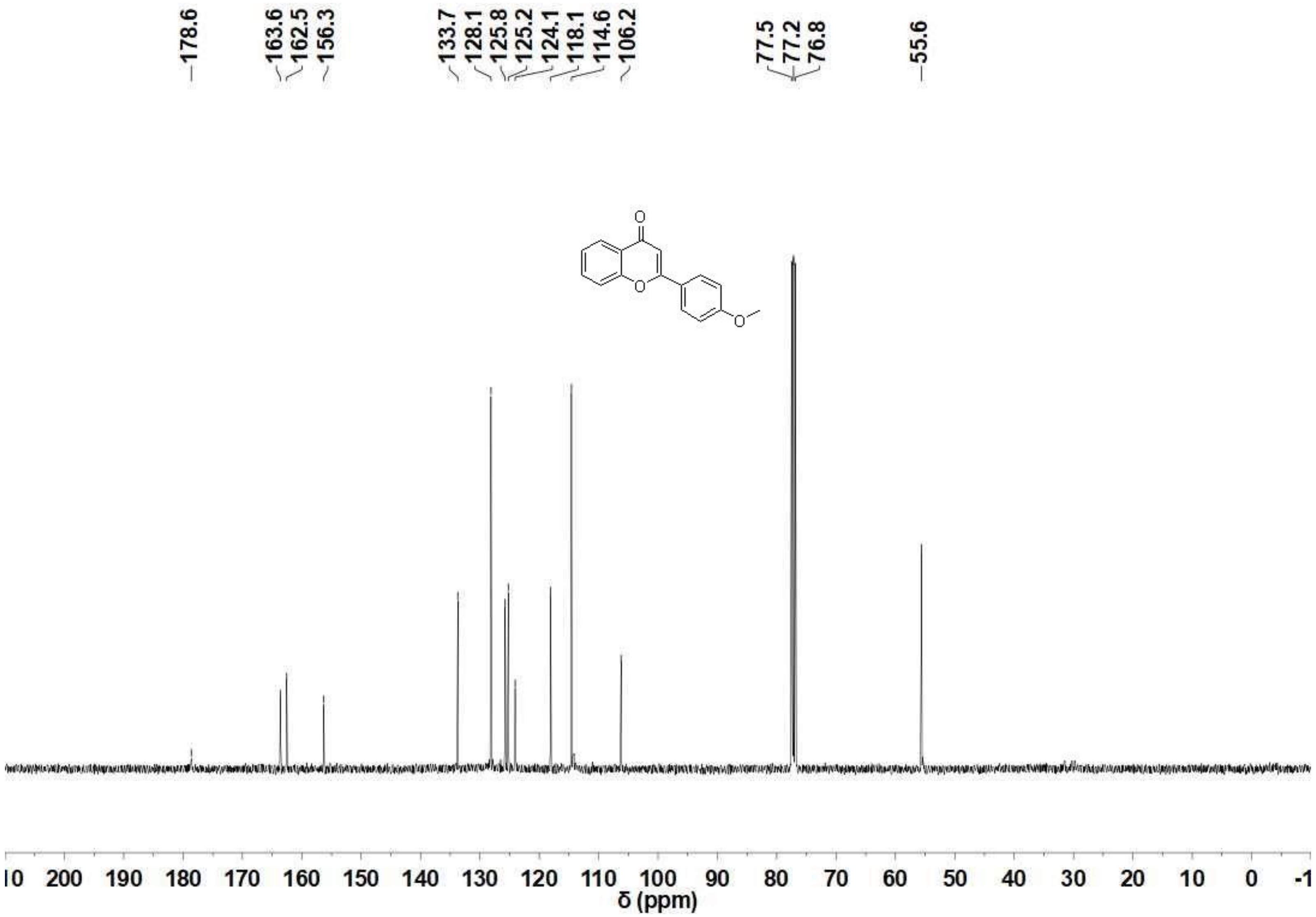
178.6
163.6
162.5
156.3
133.7
128.1
125.8
125.2
124.1
118.1
114.6
106.2
77.5
77.2
76.8
55.6
O
O
O
10 200 190 180 170 160 150 140 130 120 110 100 90 80 70 60 50 40 30 20 10 0 -1
δ (ppm)

2-(4-fluorophenyl)-*4H*-chromen-4-one, **4ma**
400 MHz, $CDCl_3$

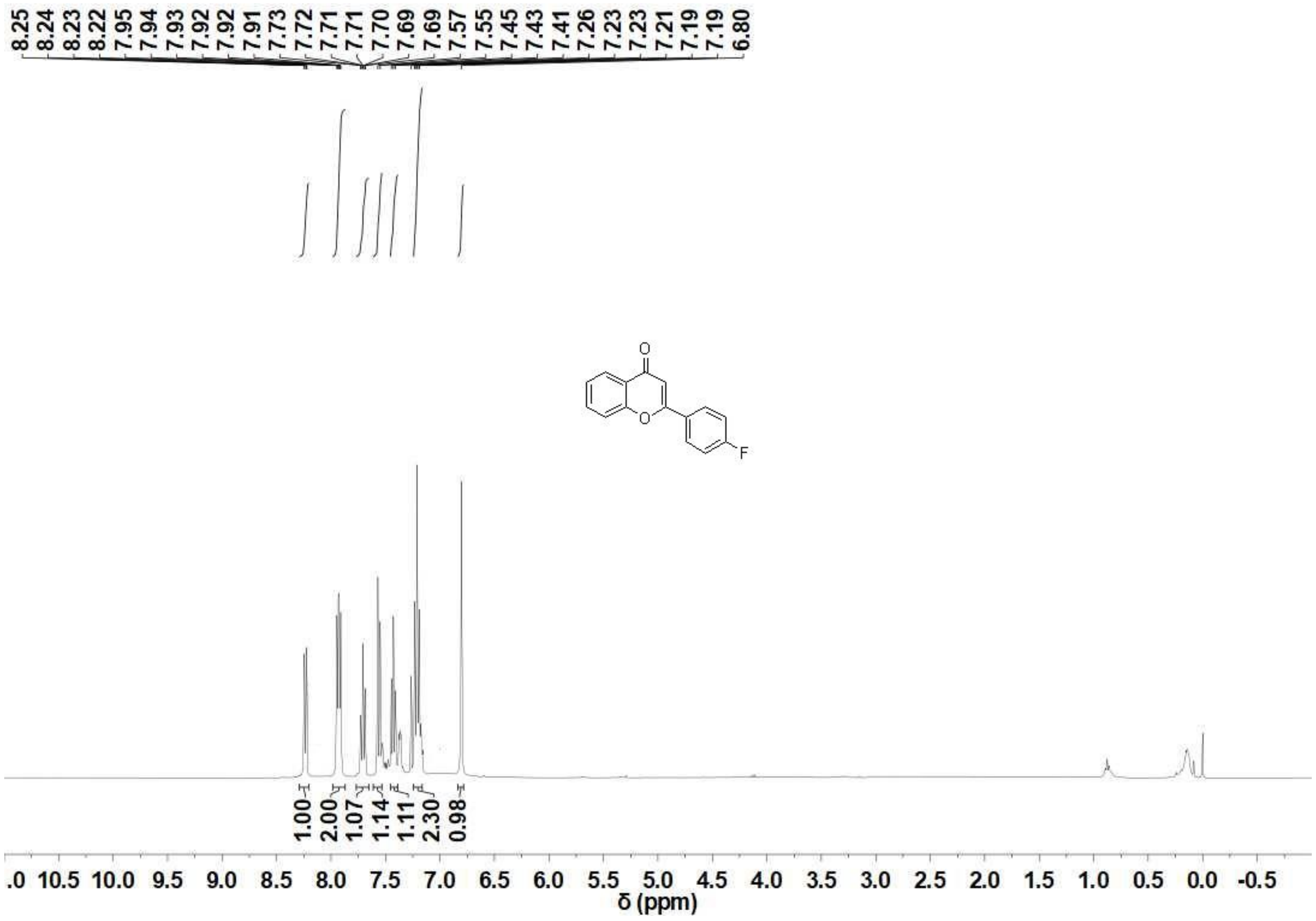

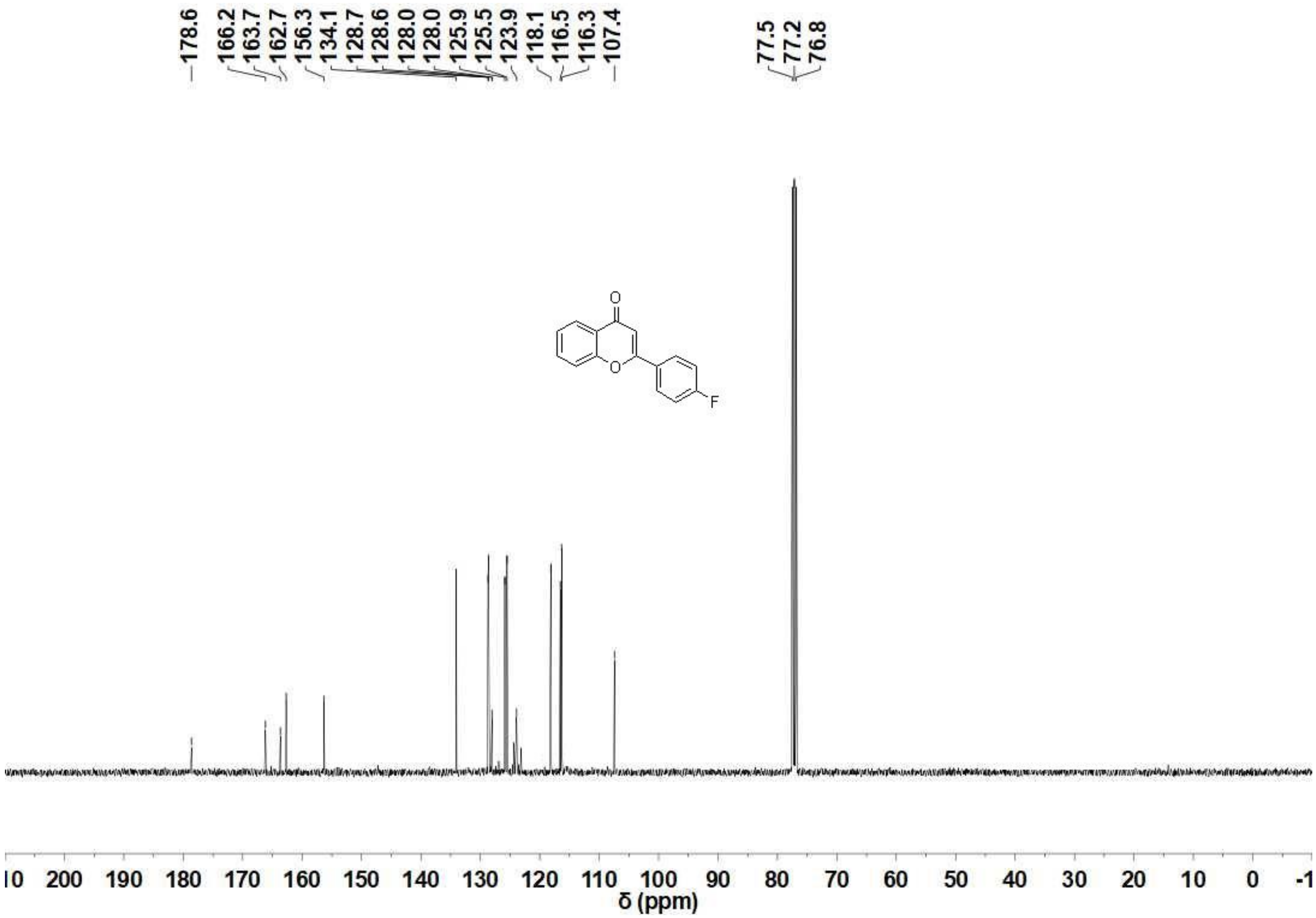
178.6
166.2
163.7
162.7
156.3
134.1
128.7
128.6
128.0
128.0
125.9
125.5
123.9
118.1
116.5
116.3
107.4
77.5
77.2
76.8
O
O
F
δ (ppm)

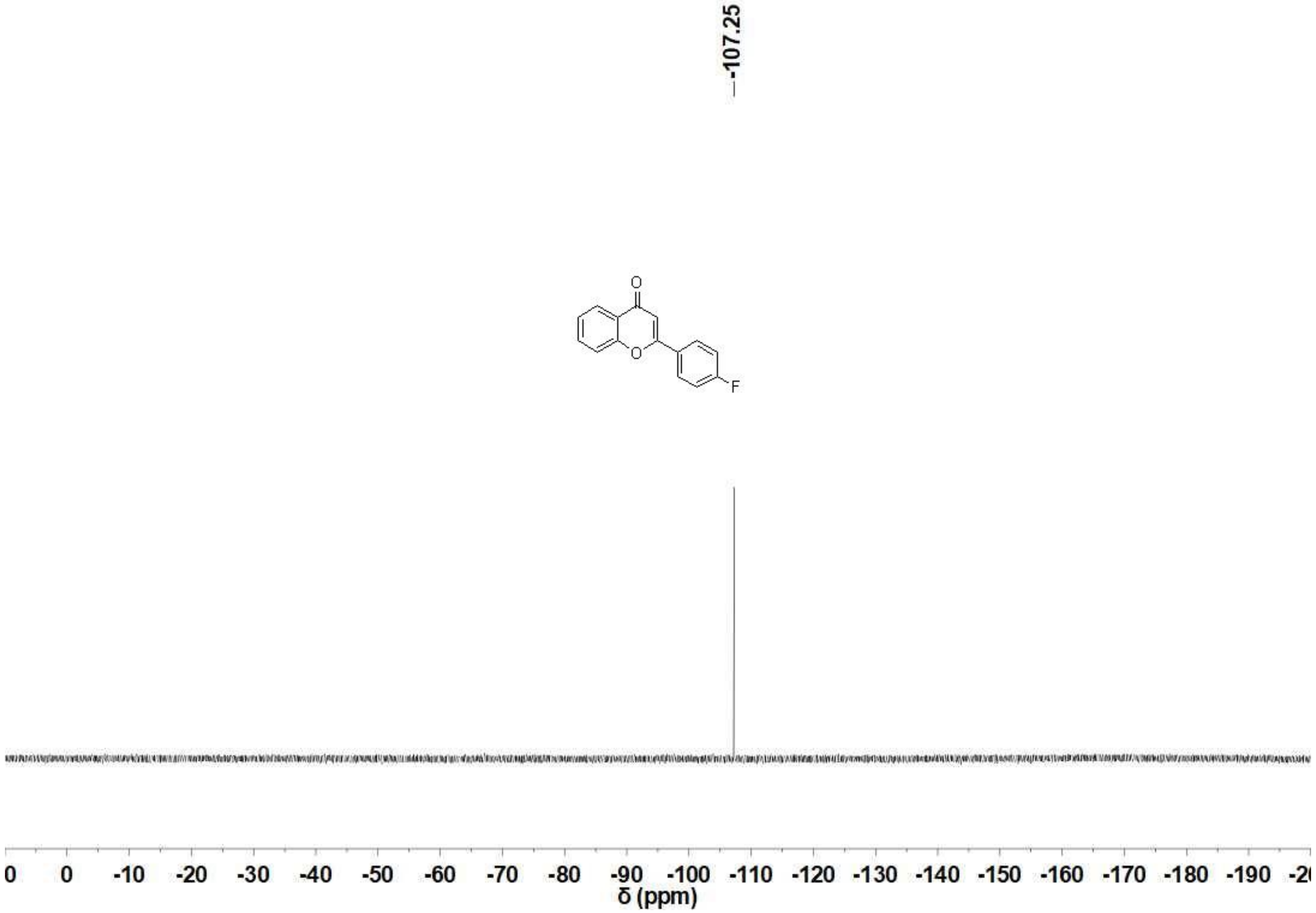
-107.25
O
O
F
0
0
-10
-20
-30
-40
-50
-60
-70
-80
-90
-100
-110
-120
-130
-140
-150
-160
-170
-180
-190
δ (ppm)

2-(4-chlorophenyl)-*4H*-chromen-4-one, **4na**

400 MHz, $CDCl_3$

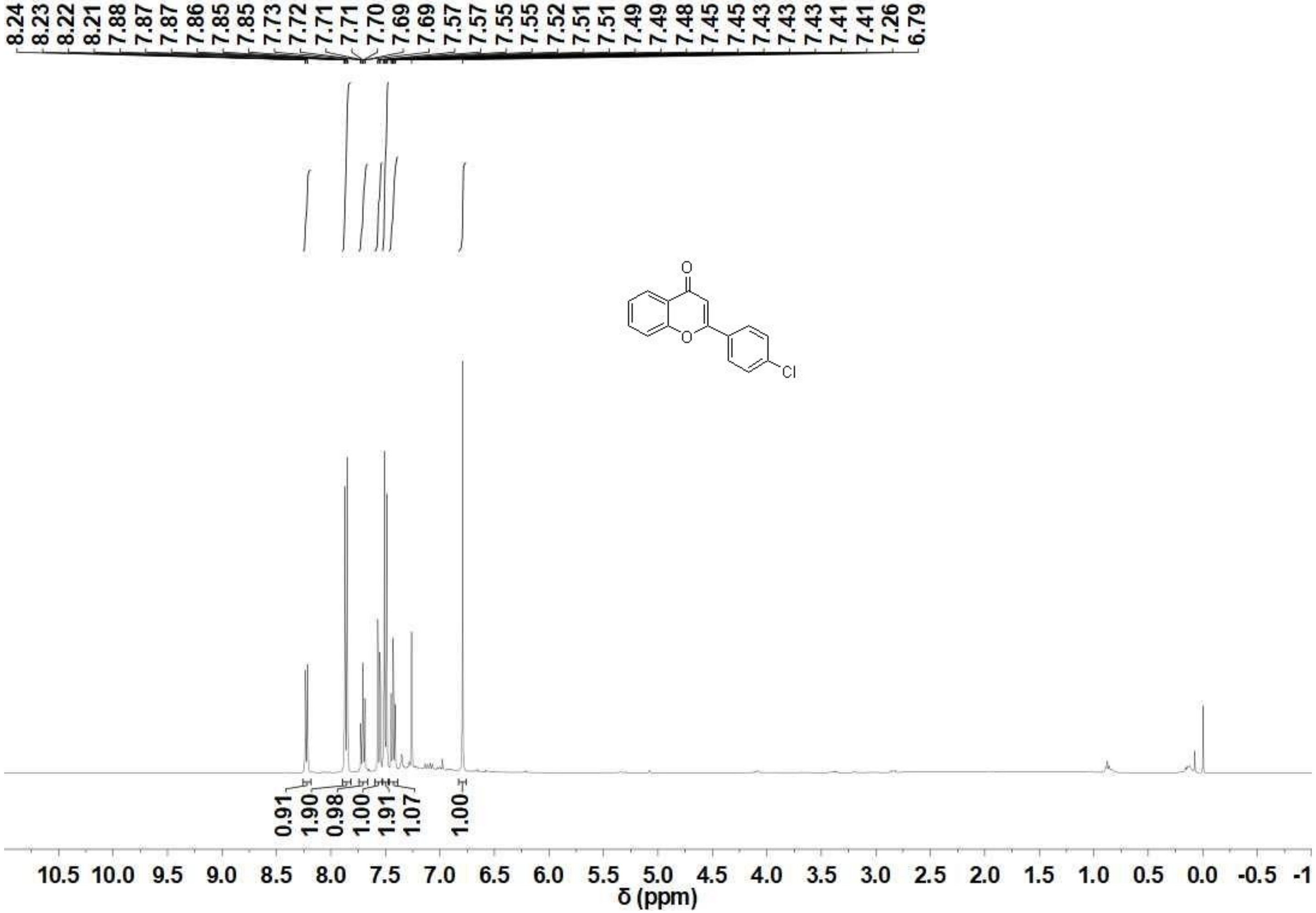

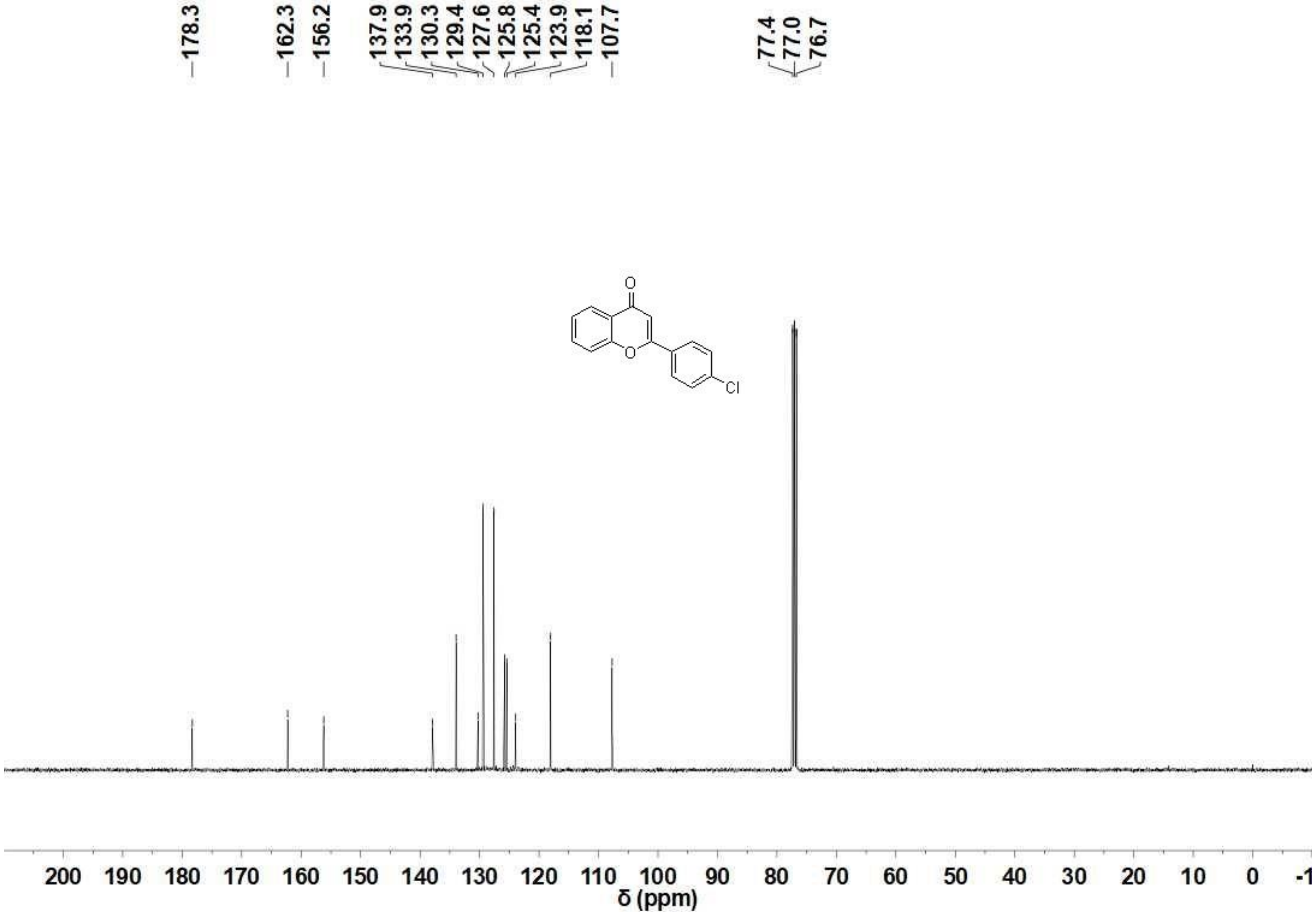
178.3
162.3
156.2
137.9
133.9
130.3
129.4
127.6
125.8
125.4
123.9
118.1
107.7
77.4
77.0
76.7
O
O
Cl
200 190 180 170 160 150 140 130 120 110 100 90 80 70 60 50 40 30 20 10 0 -1
δ (ppm)

2-(4-bromophenyl)-*4H*-chromen-4-one, **4oa**

400 MHz, $CDCl_3$

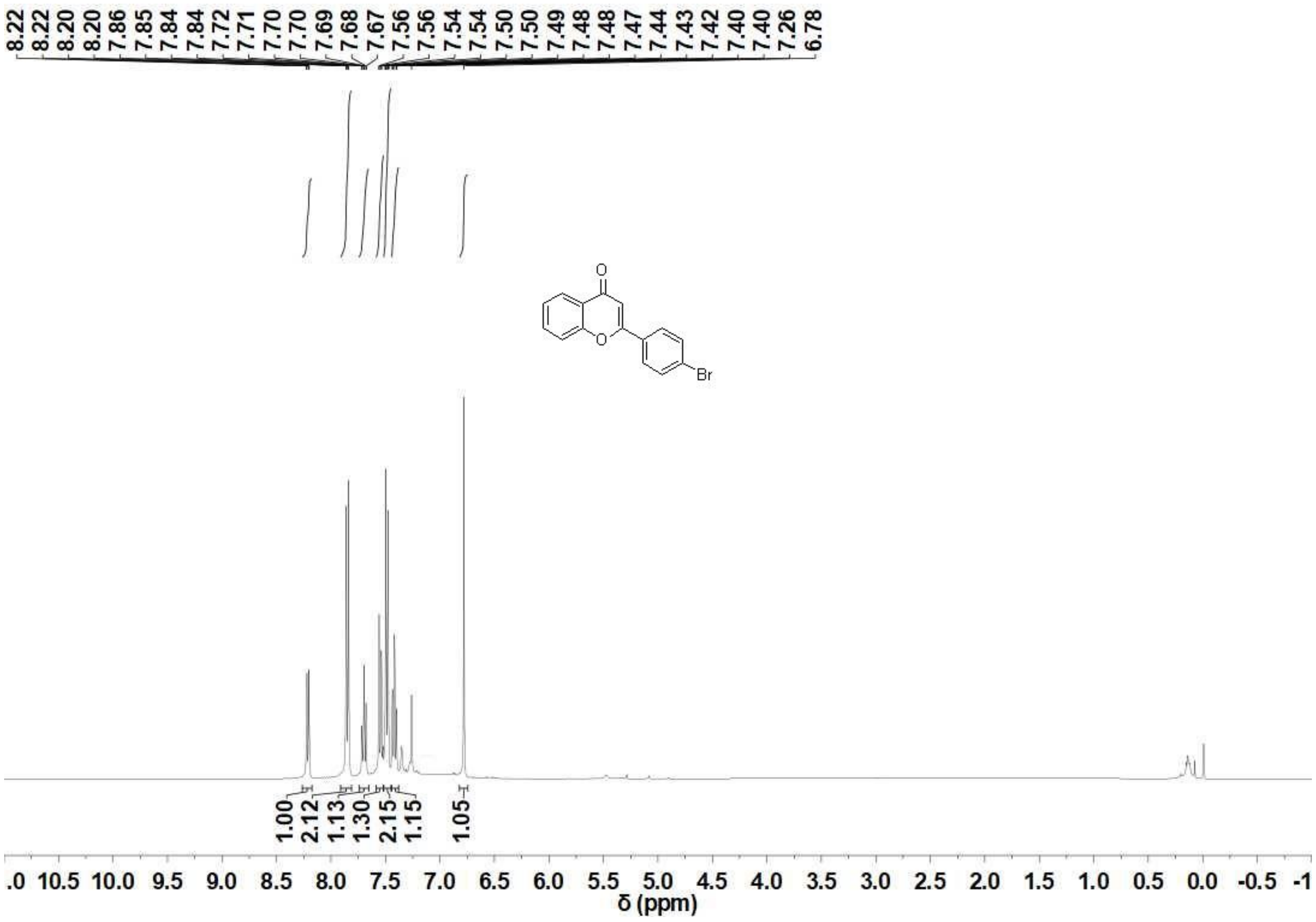

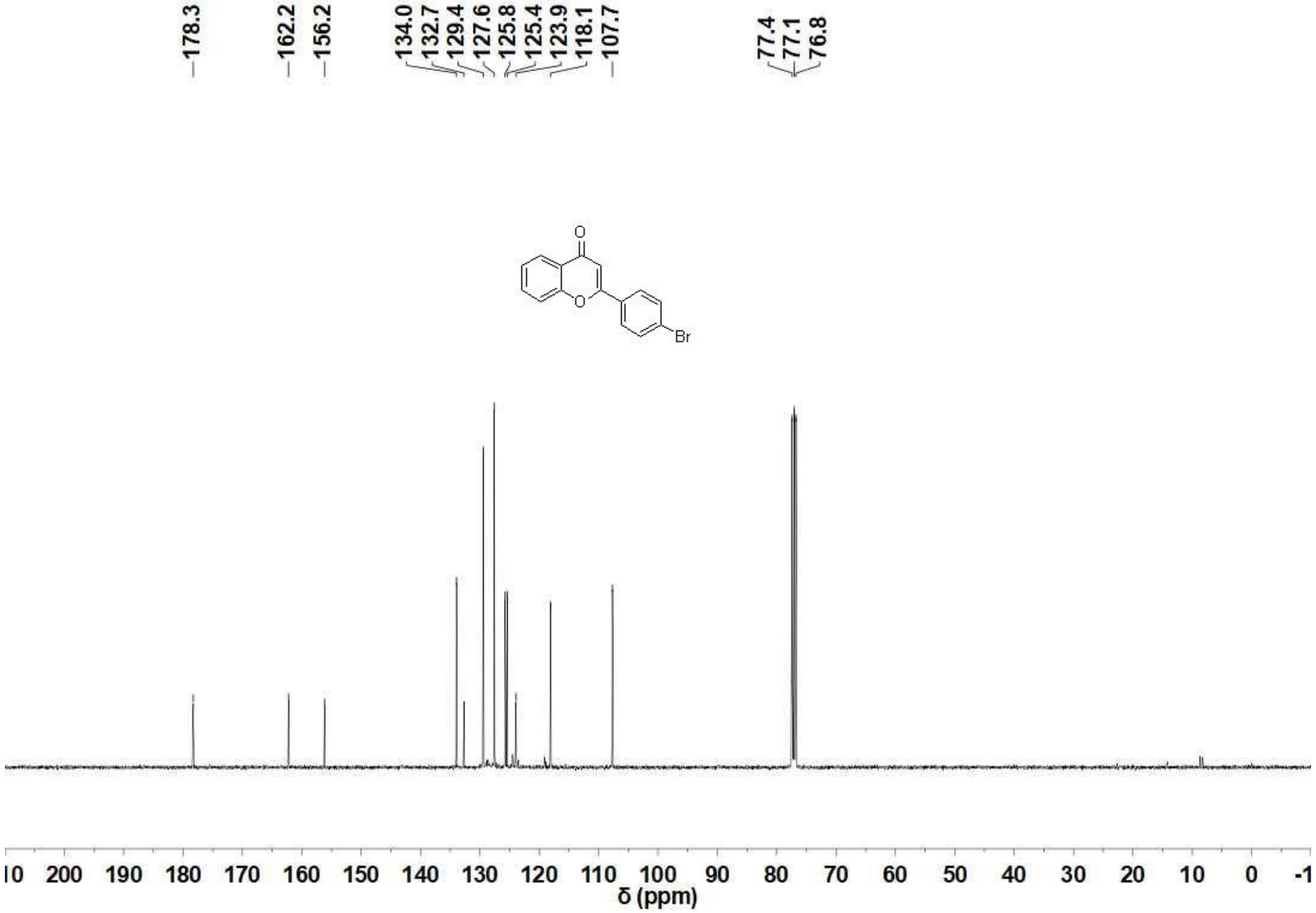
178.3
162.2
156.2
134.0
132.7
129.4
127.6
125.8
125.4
123.9
118.1
107.7
77.4
77.1
76.8
O
O
Br
10 200 190 180 170 160 150 140 130 120 110 100 90 80 70 60 50 40 30 20 10 0 -1
δ (ppm)

2-(4-(trifluoromethyl)phenyl)-*4H*-chromen-4-one, **4pa**

400 MHz, $CDCl_3$

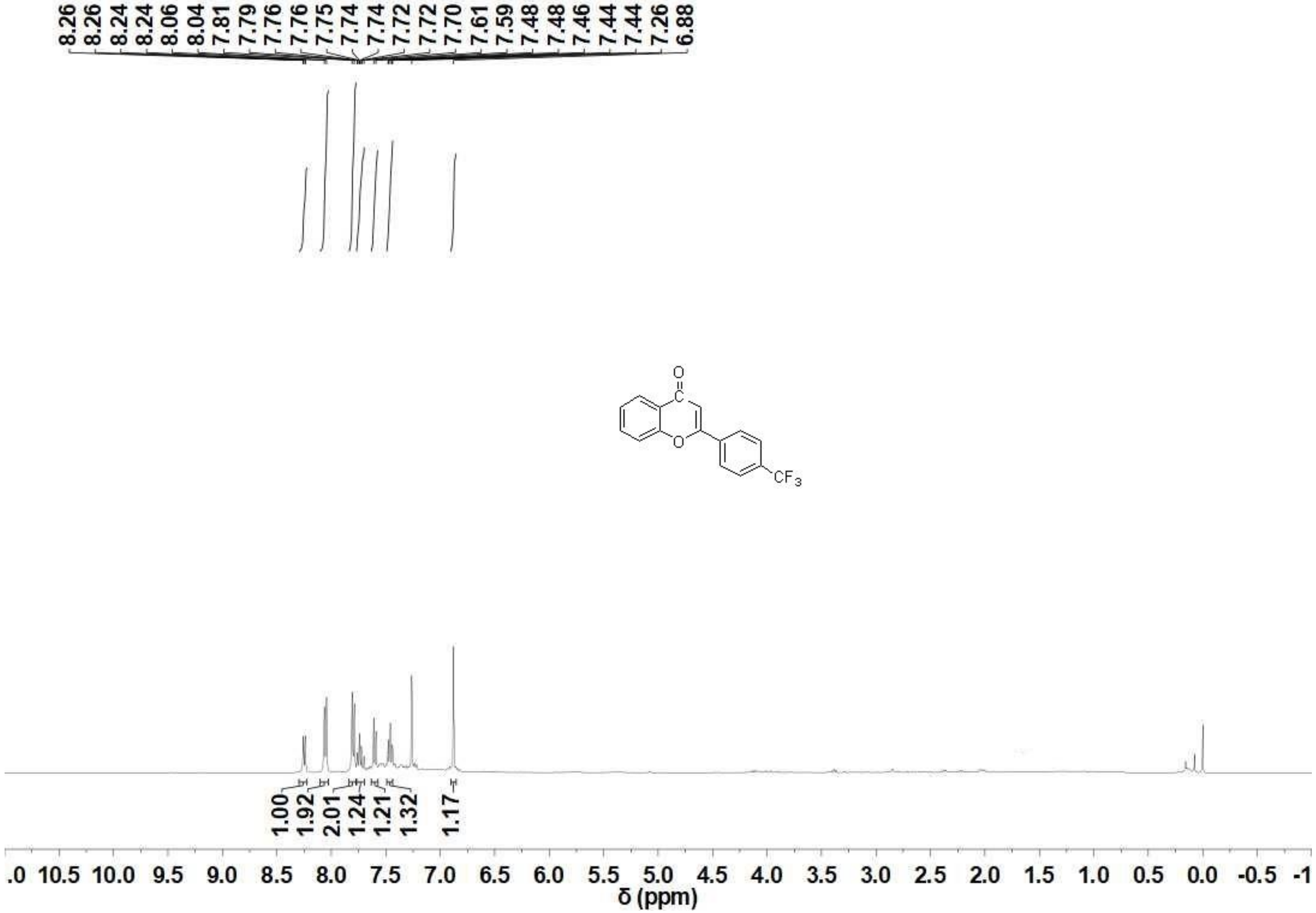

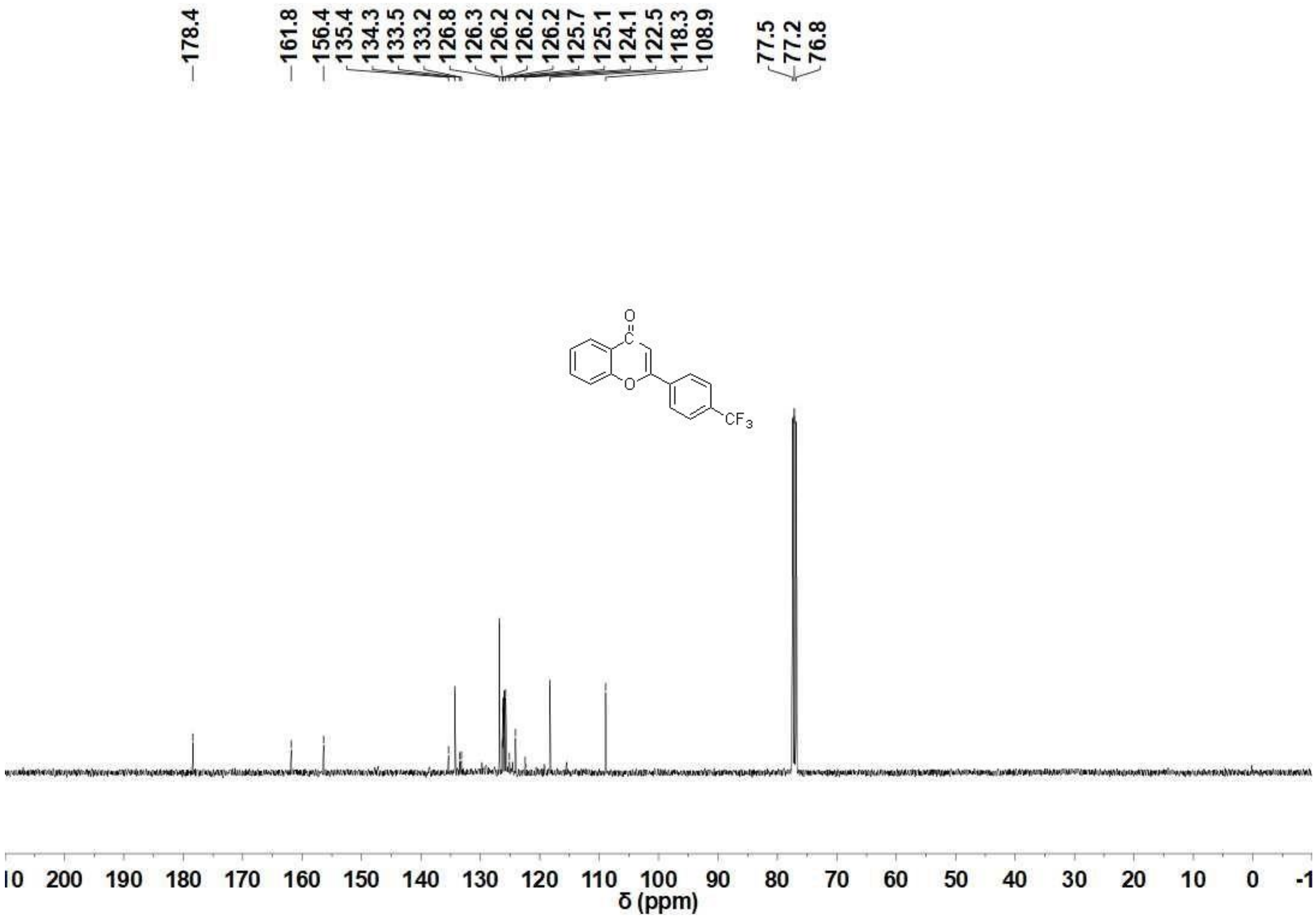
178.4
161.8
156.4
135.4
134.3
133.5
133.2
126.8
126.3
126.2
126.2
126.2
125.7
125.1
124.1
122.5
118.3
108.9
77.5
77.2
76.8
O
O
CF3
10
200
190
180
170
160
150
140
130
120
110
100
90
80
70
60
50
40
30
20
10
0
-1
δ (ppm)

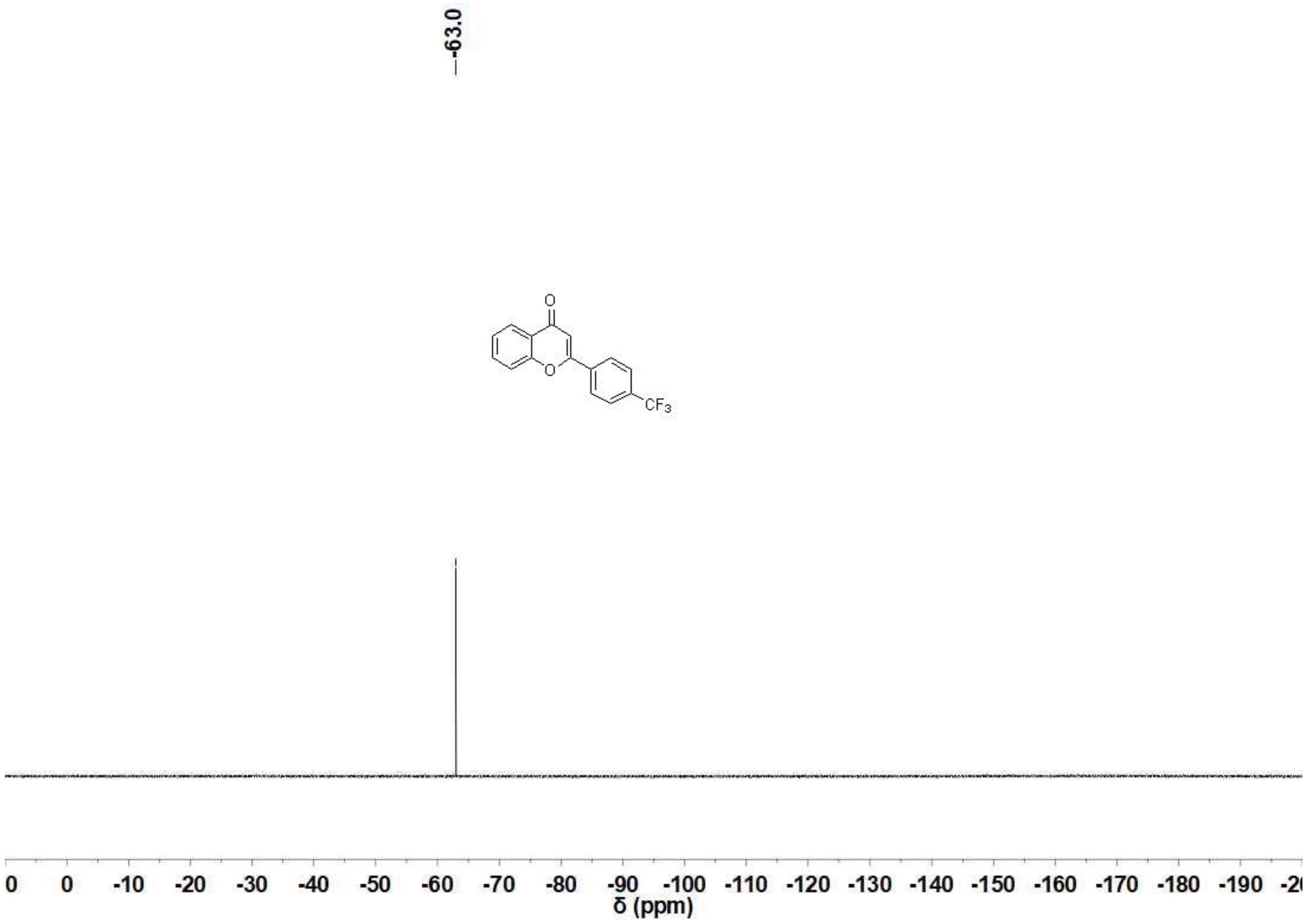
-63.0
O
O
CF3
0 0 -10 -20 -30 -40 -50 -60 -70 -80 -90 -100 -110 -120 -130 -140 -150 -160 -170 -180 -190
δ (ppm)

2-(4-hydroxyphenyl)-*4H*-chromen-4-one, **4qa**
400 MHz, DMSO-d_6

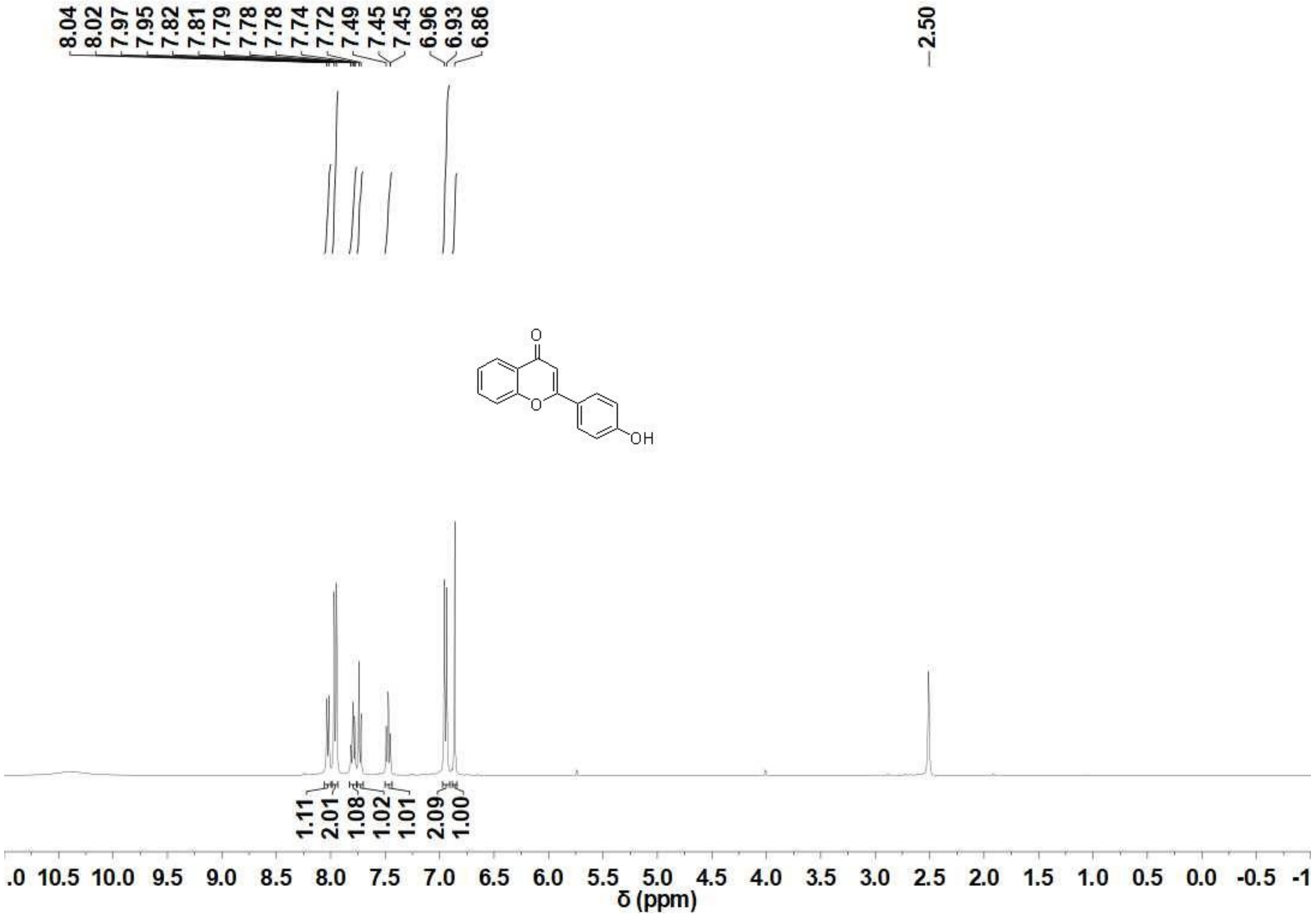

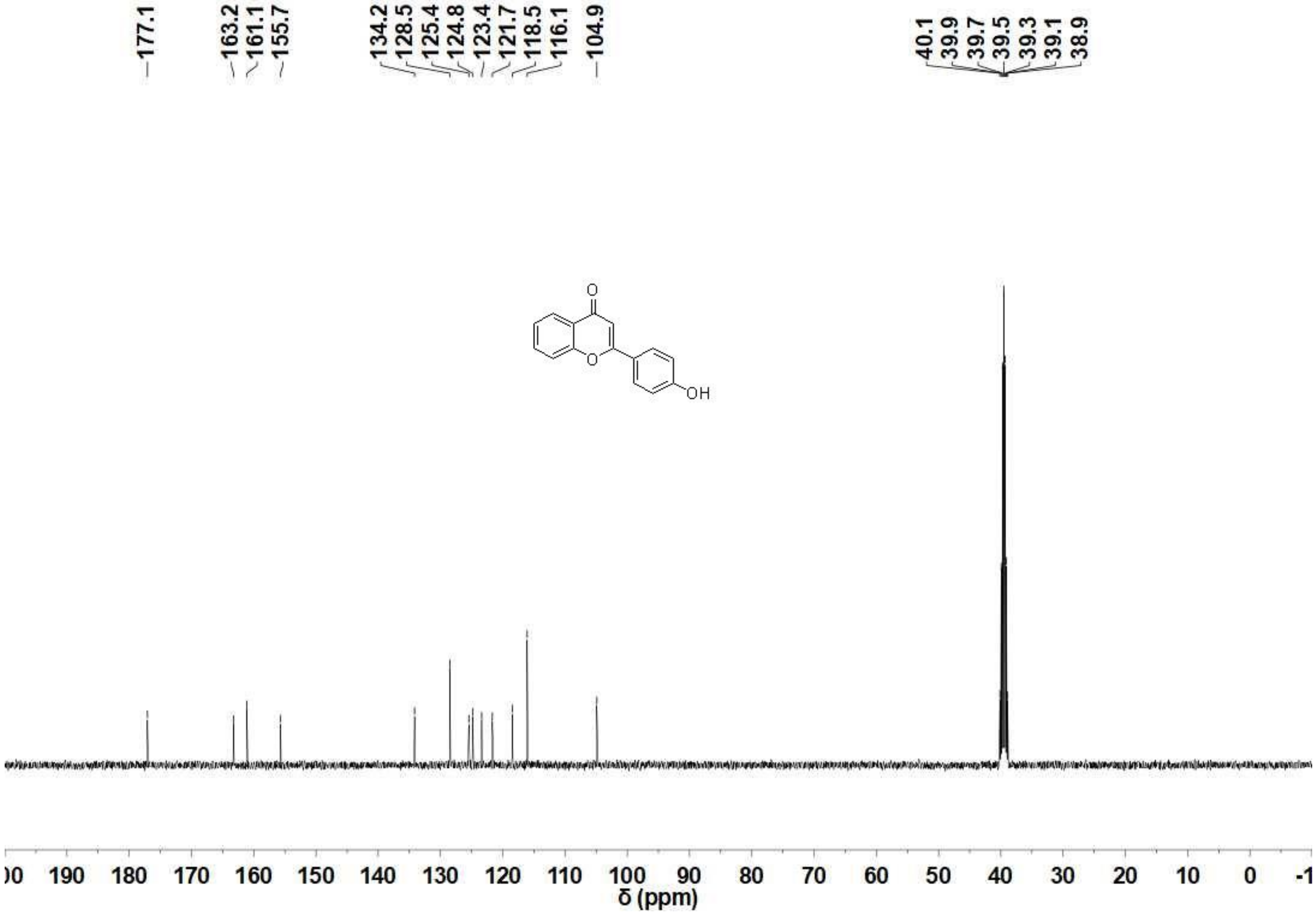

177.1
163.2
161.1
155.7
134.2
128.5
125.4
124.8
123.4
121.7
118.5
116.1
104.9
40.1
39.9
39.7
39.5
39.3
39.1
38.9
O
O
OH
190
180
170
160
150
140
130
120
110
100
90
80
70
60
50
40
30
20
10
0
δ (ppm)

2-(naphthalen-2-yl)-*4H*-chromen-4-one, **4ra**
400 MHz, $CDCl_3$

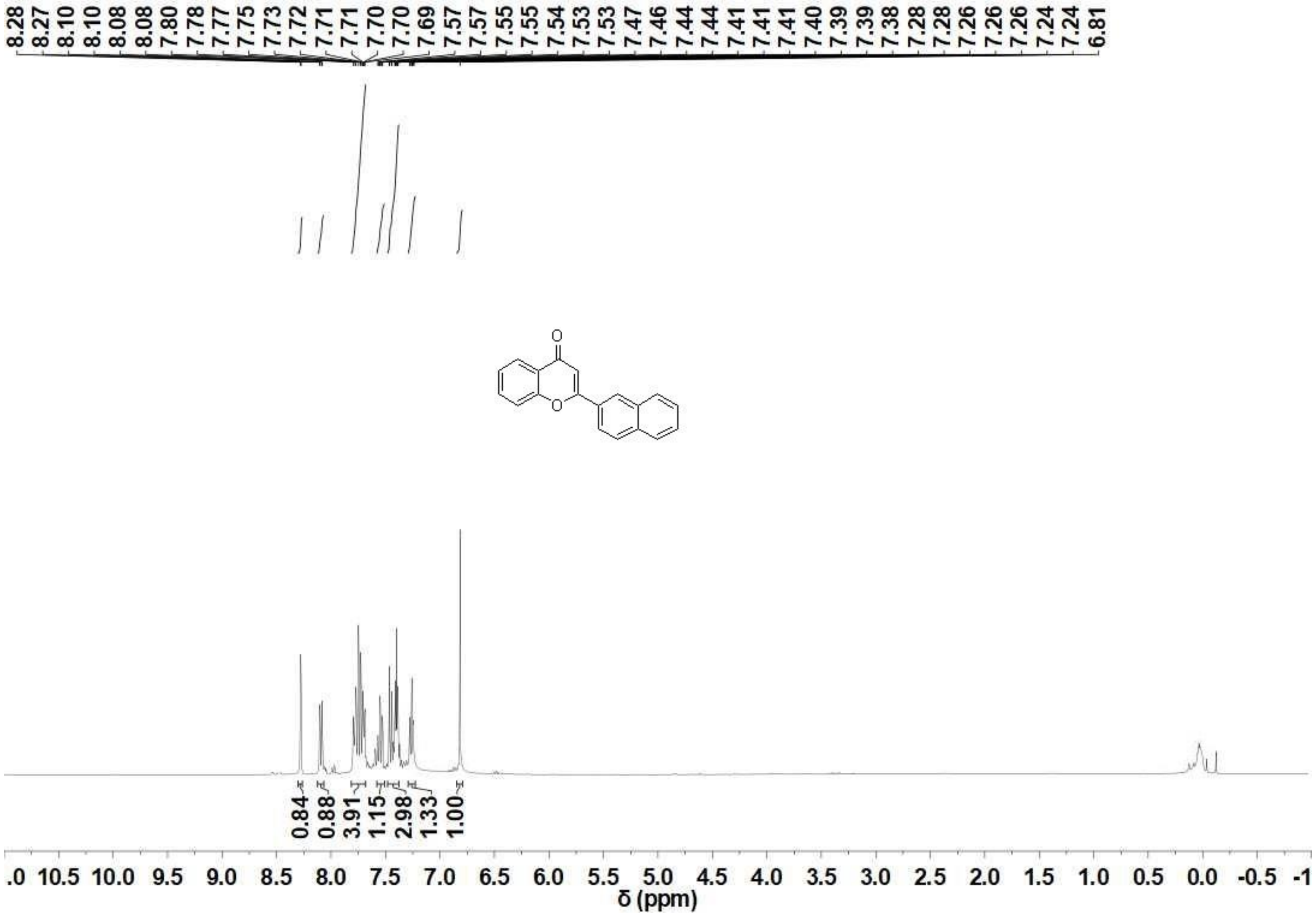

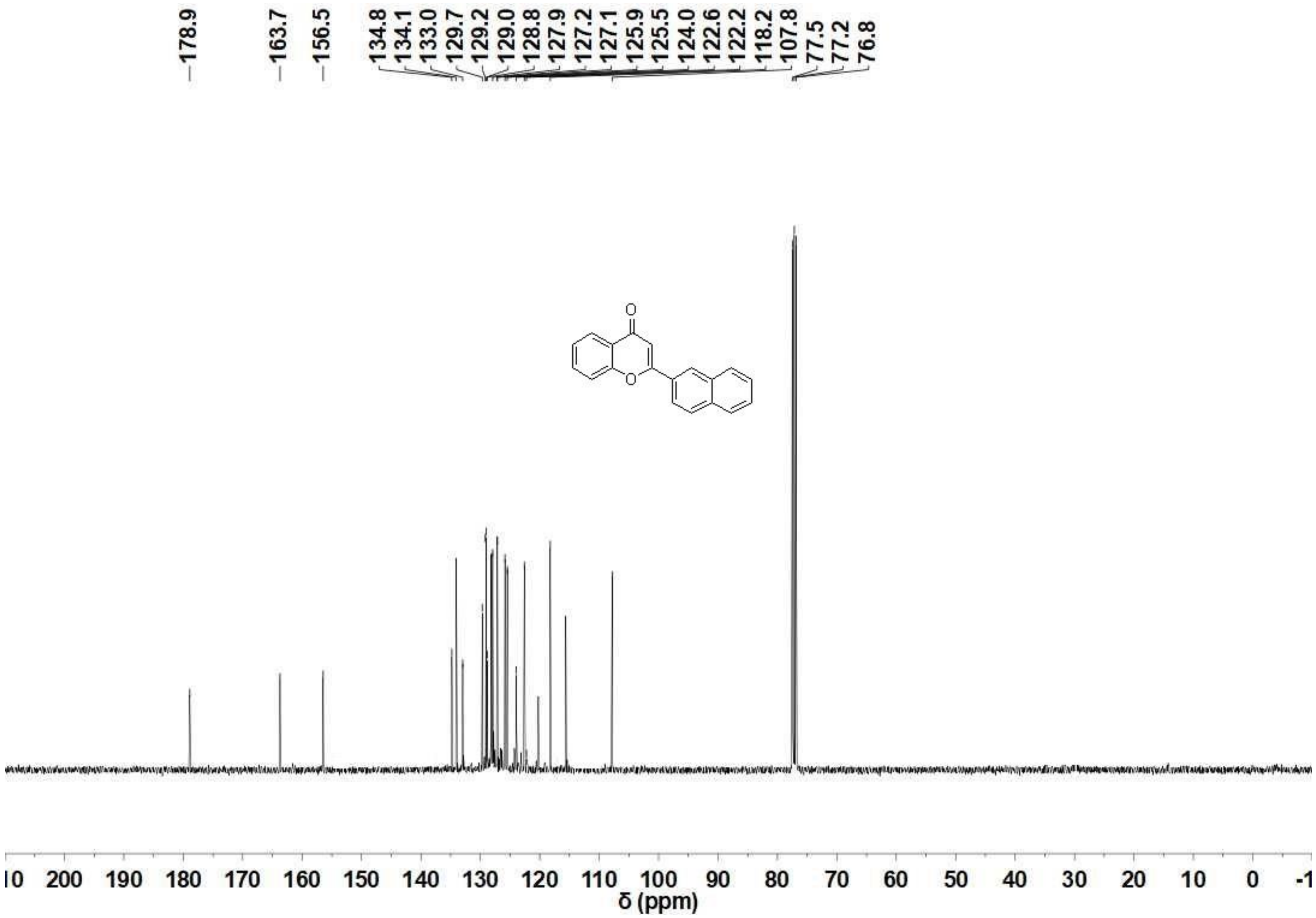
178.9
163.7
156.5
134.8
134.1
133.0
129.7
129.2
129.0
128.8
127.9
127.2
127.1
125.9
125.5
124.0
122.6
122.2
118.2
107.8
77.5
77.2
76.8
O
O
δ (ppm)

2-(3,5-dimethoxyphenyl)-*4H*-chromen-4-one, **4sa**
400 MHz, $CDCl_3$

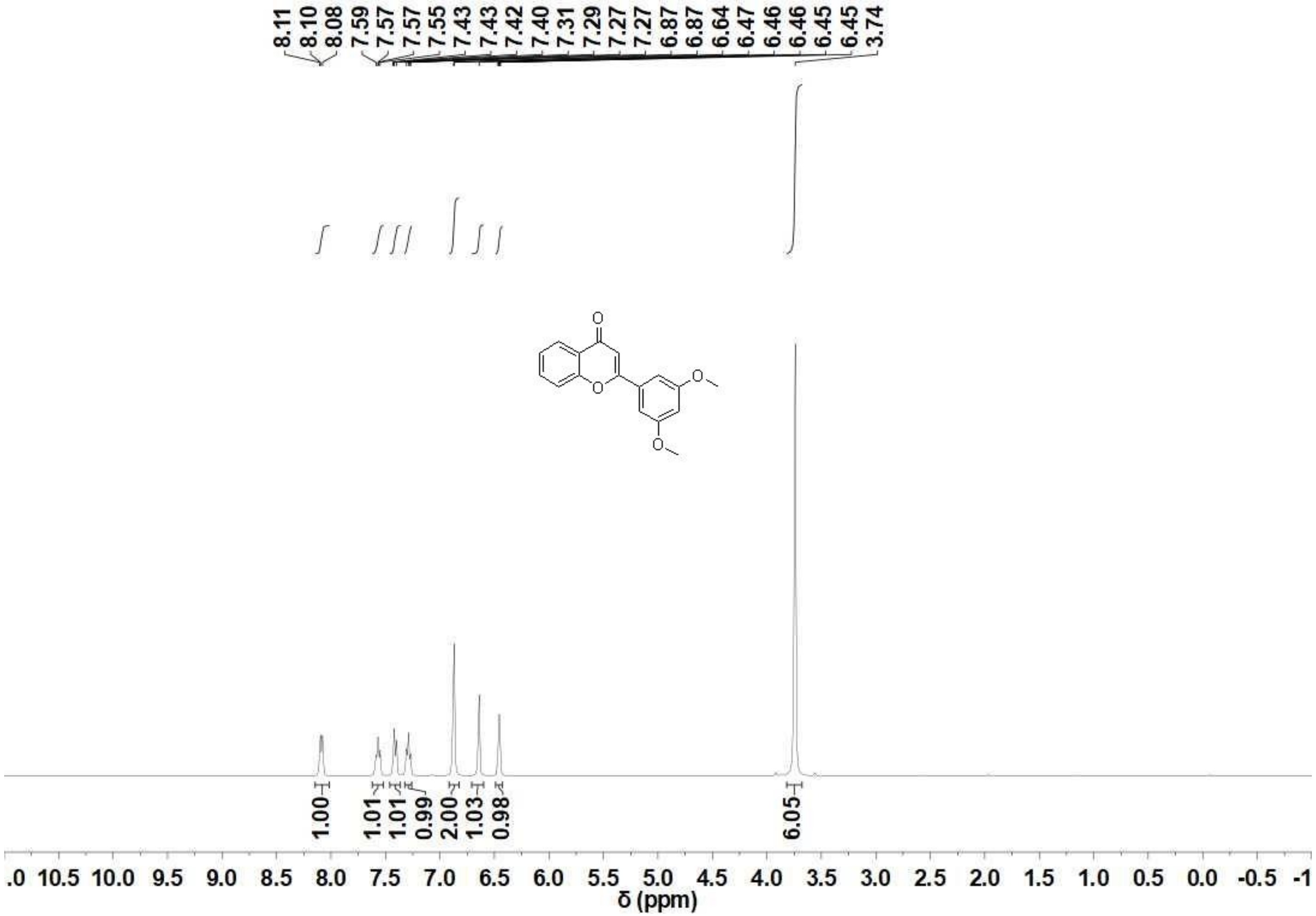

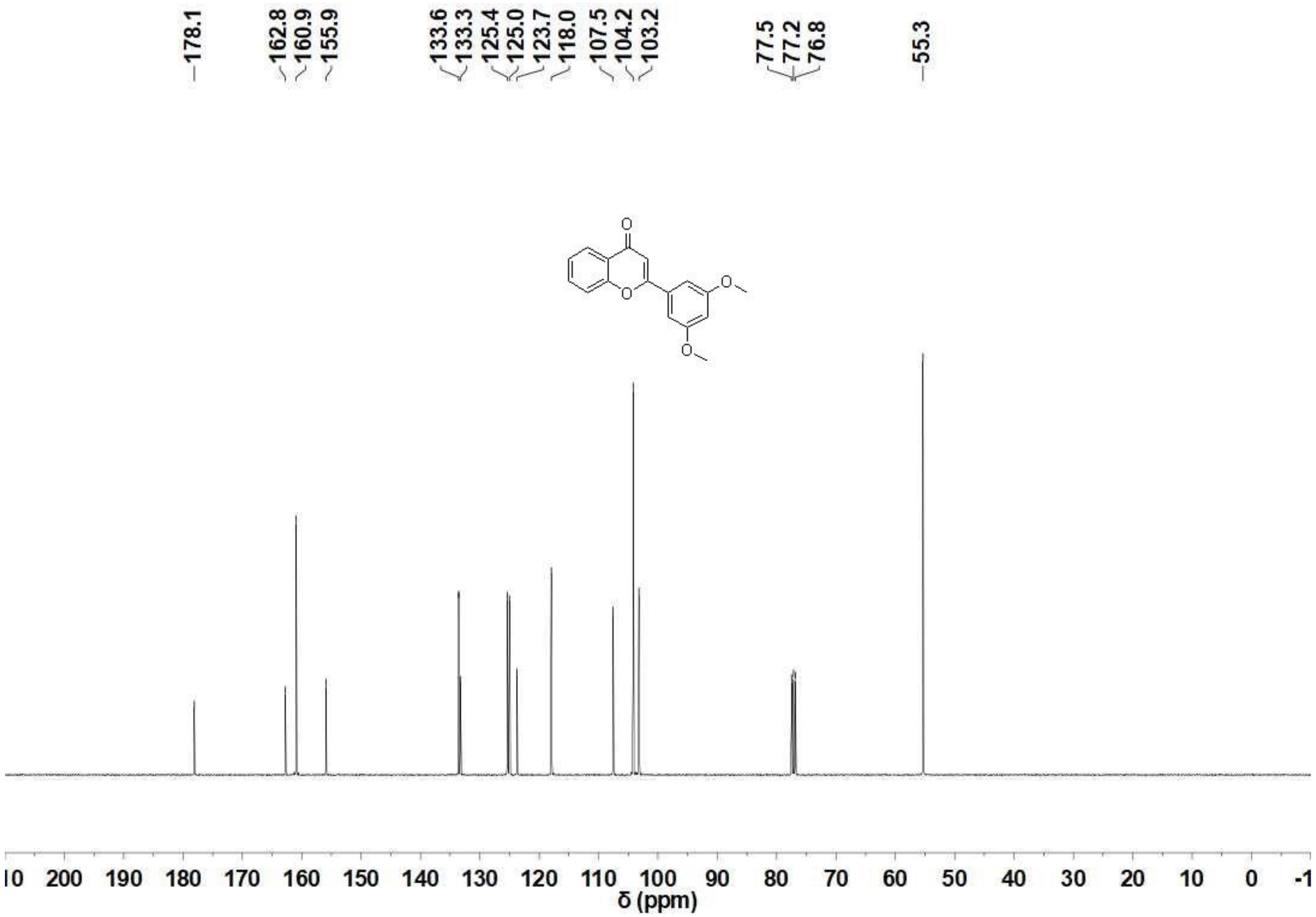
178.1
162.8
160.9
155.9
133.6
133.3
125.4
125.0
123.7
118.0
107.5
104.2
103.2
77.5
77.2
76.8
55.3
O
O
O
O
10
200
190
180
170
160
150
140
130
120
110
100
90
80
70
60
50
40
30
20
10
0
-1
δ (ppm)

2-(3,4-dimethoxyphenyl)-*4H*-chromen-4-one, **4ta**
400 MHz, $CDCl_3$

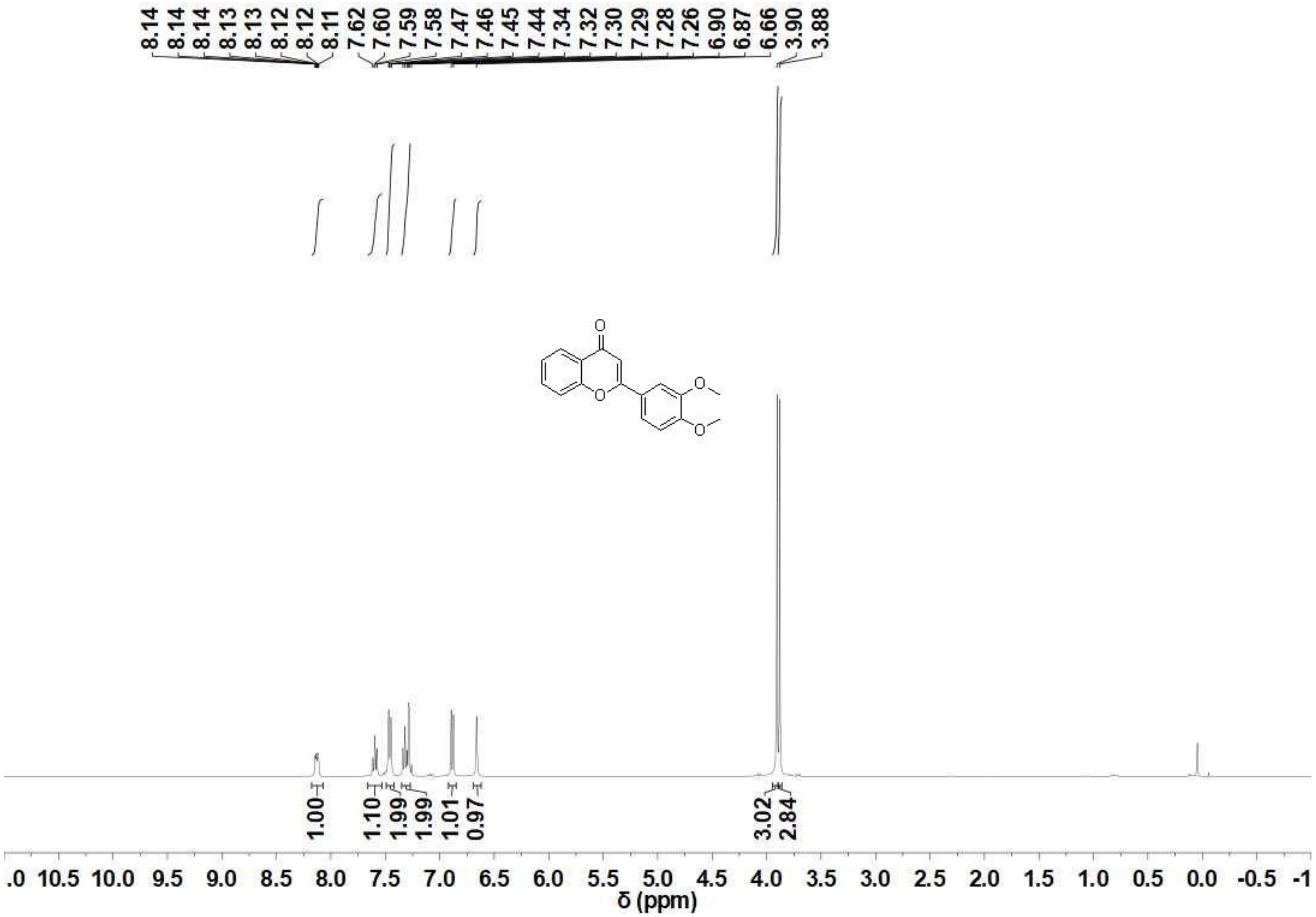

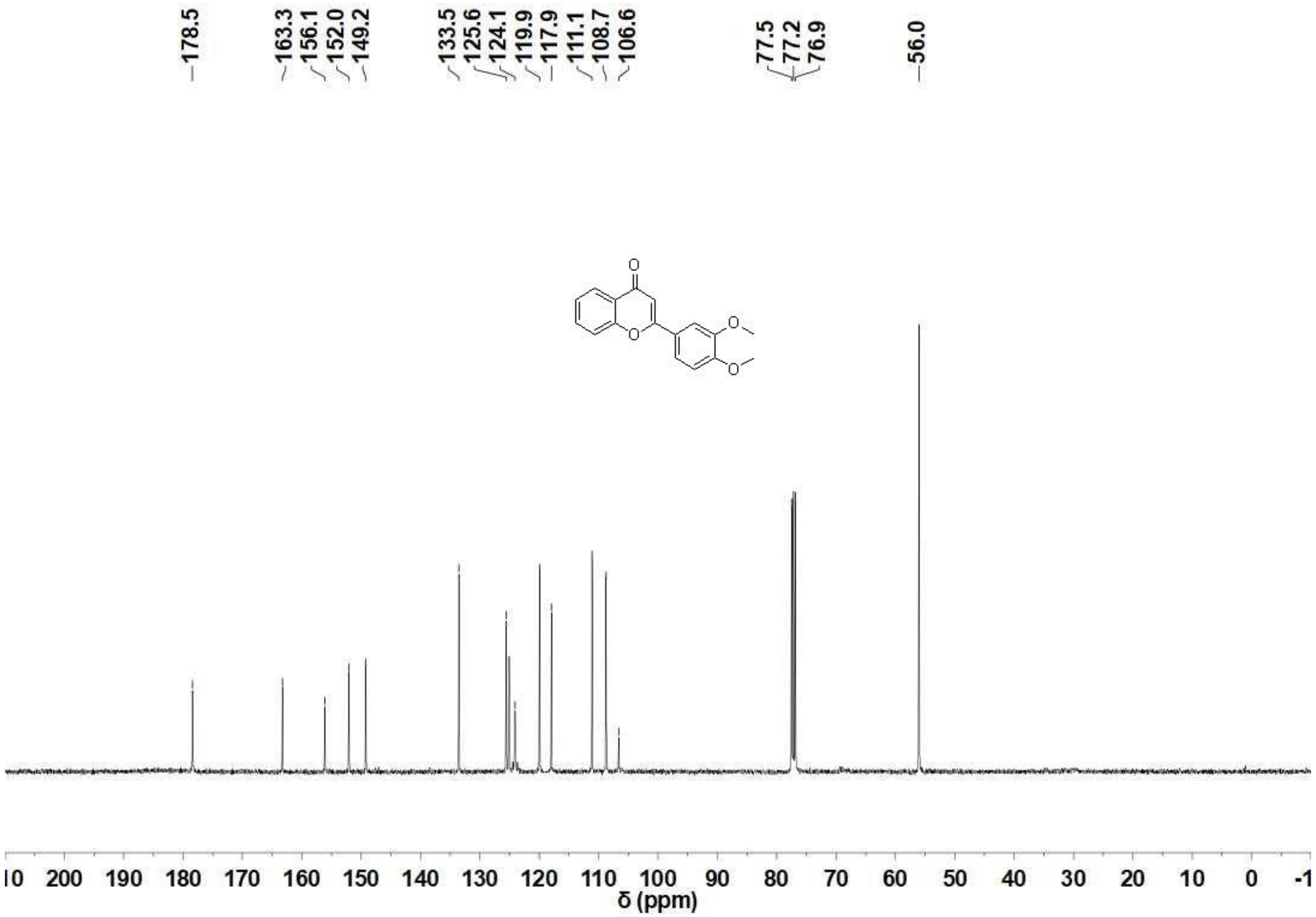

178.5
163.3
156.1
152.0
149.2
133.5
125.6
124.1
119.9
117.9
111.1
108.7
106.6
77.5
77.2
76.9
56.0
O
O
O
O
10 200 190 180 170 160 150 140 130 120 110 100 90 80 70 60 50 40 30 20 10 0 -1
δ (ppm)

2-(thiophen-3-yl)-*4H*-chromen-4-one, **4ua**
400 MHz, $CDCl_3$

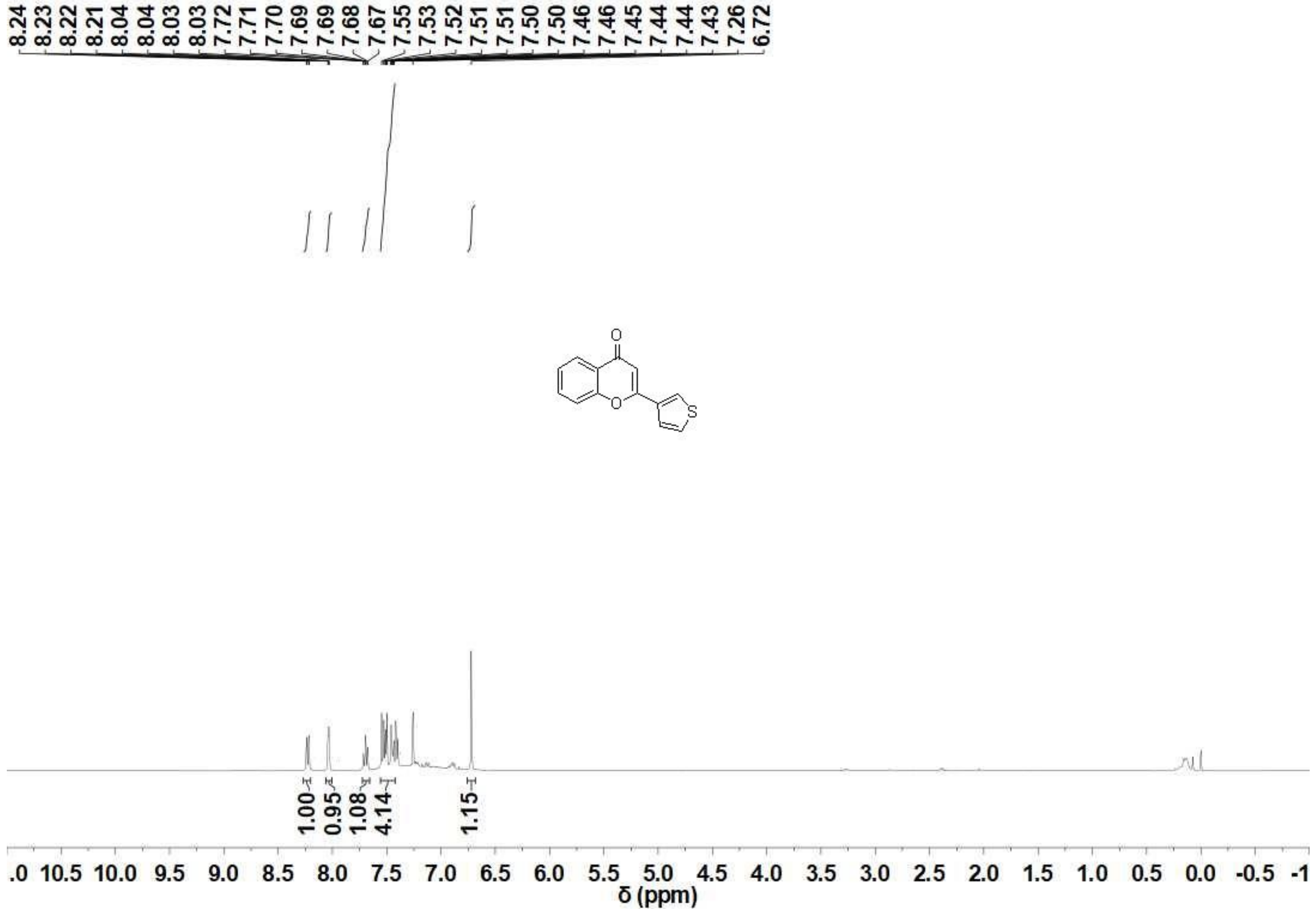

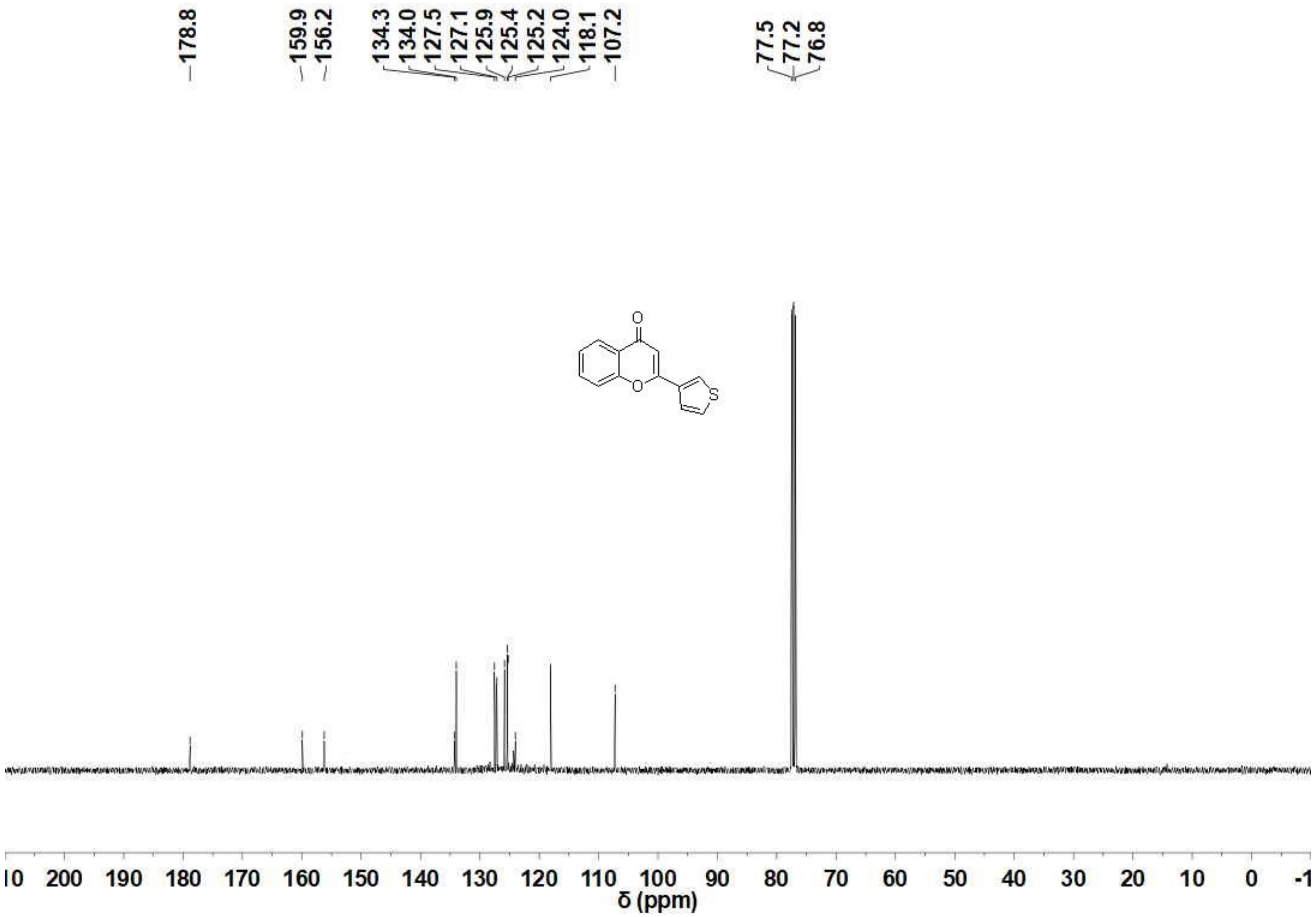

178.8
159.9
156.2
134.3
134.0
127.5
127.1
125.9
125.4
125.2
124.0
118.1
107.2
77.5
77.2
76.8
O
O
S
10 200 190 180 170 160 150 140 130 120 110 100 90 80 70 60 50 40 30 20 10 0 -1
δ (ppm)

2,2'-(1,4-phenylene)bis(*4H*-chromen-4-one), **4va**
400 MHz, $CDCl_3$

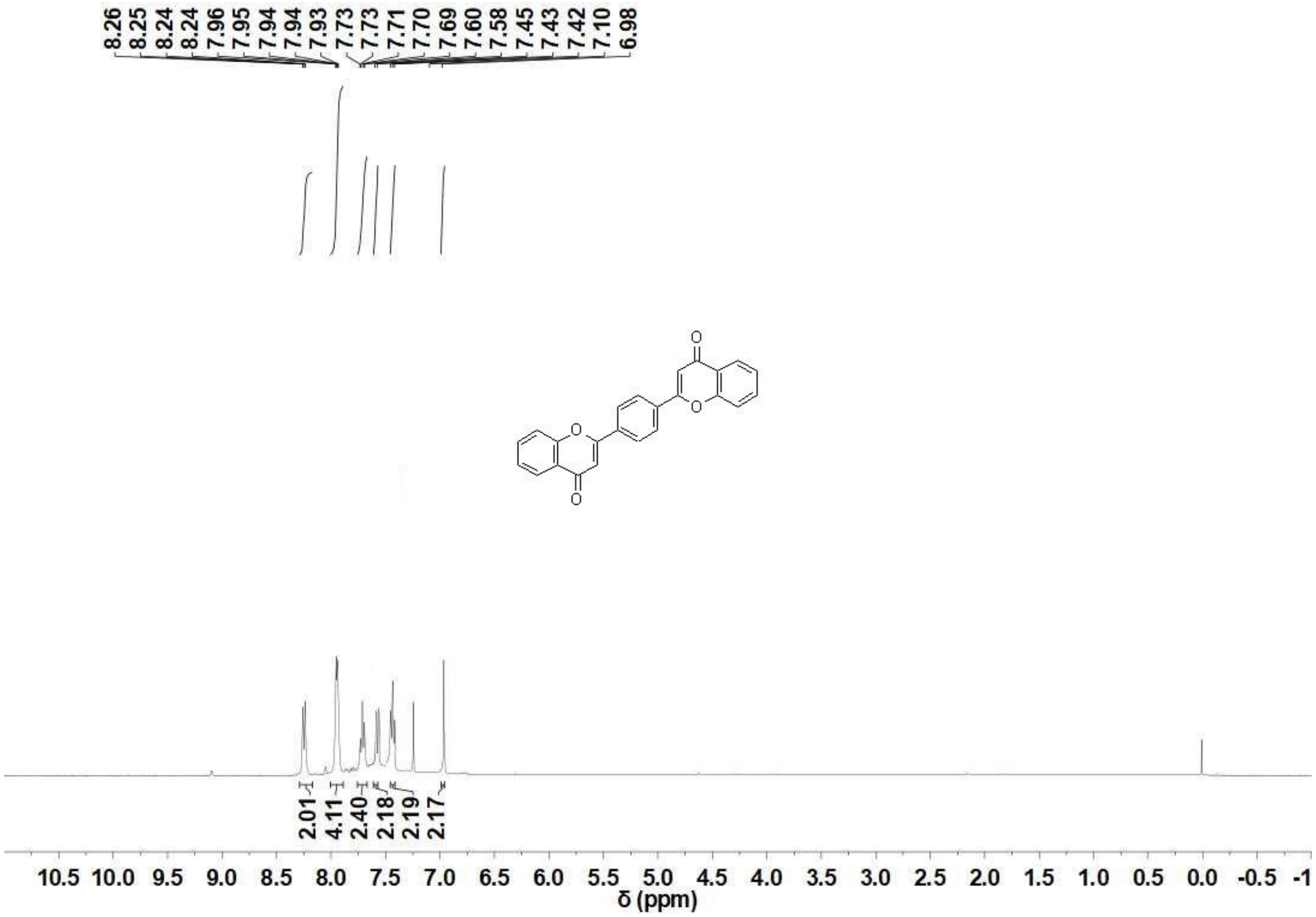

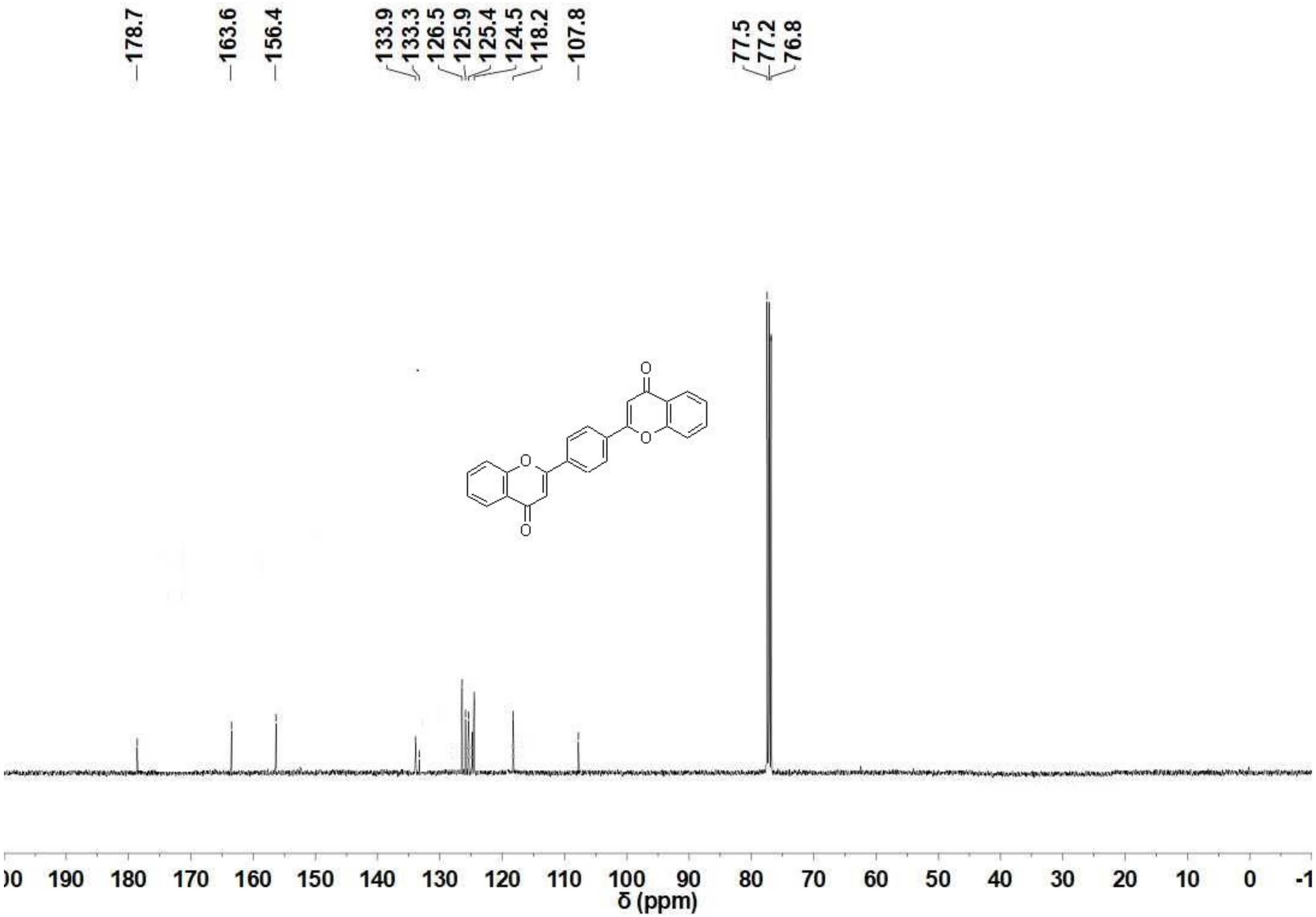
178.7
163.6
156.4
133.9
133.3
126.5
125.9
125.4
124.5
118.2
107.8
77.5
77.2
76.8
O
O
O
O
δ (ppm)

7-methoxy-2-phenyl-*4H*-chromen-4-one, **4ab**

400 MHz, $CDCl_3$

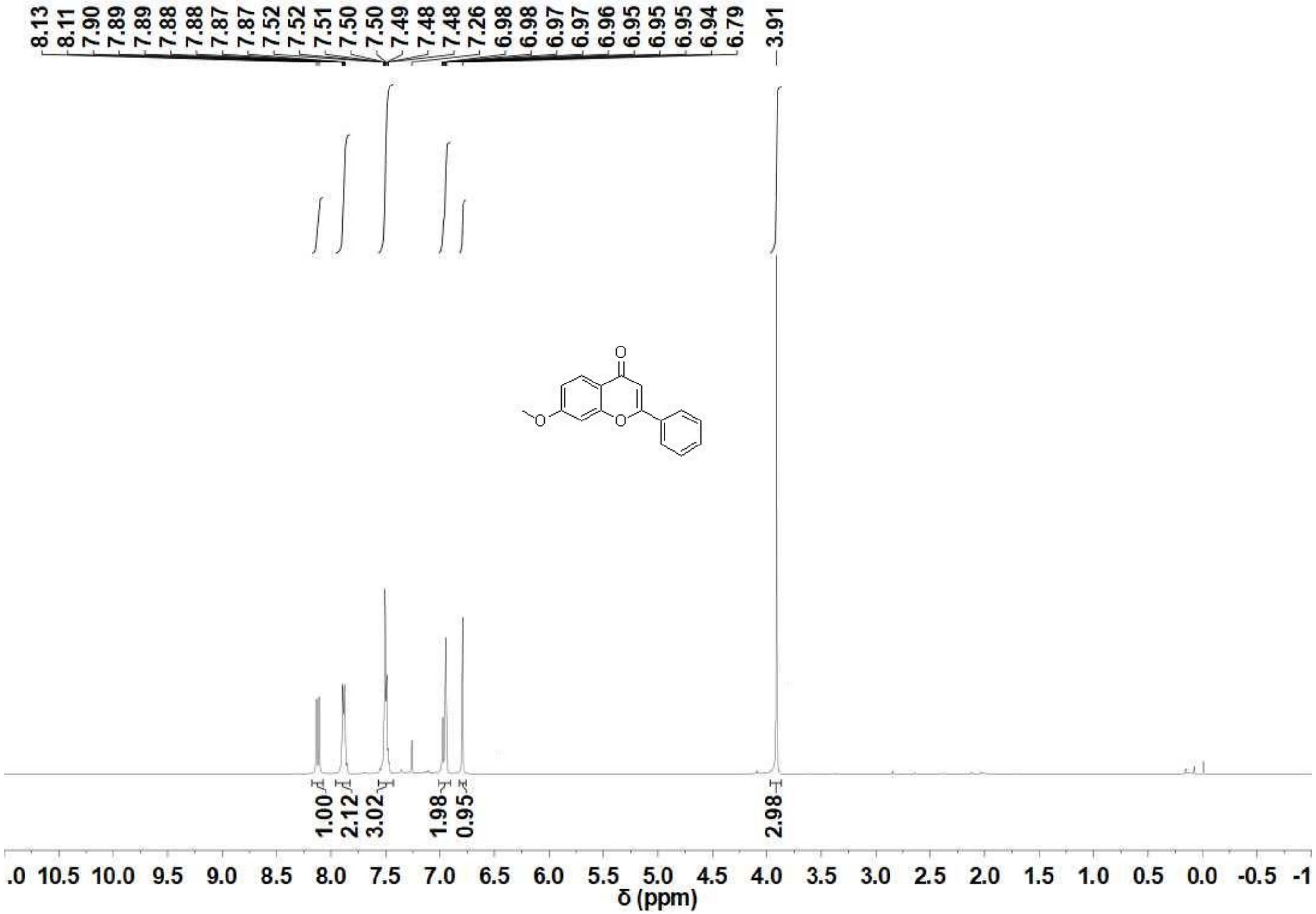

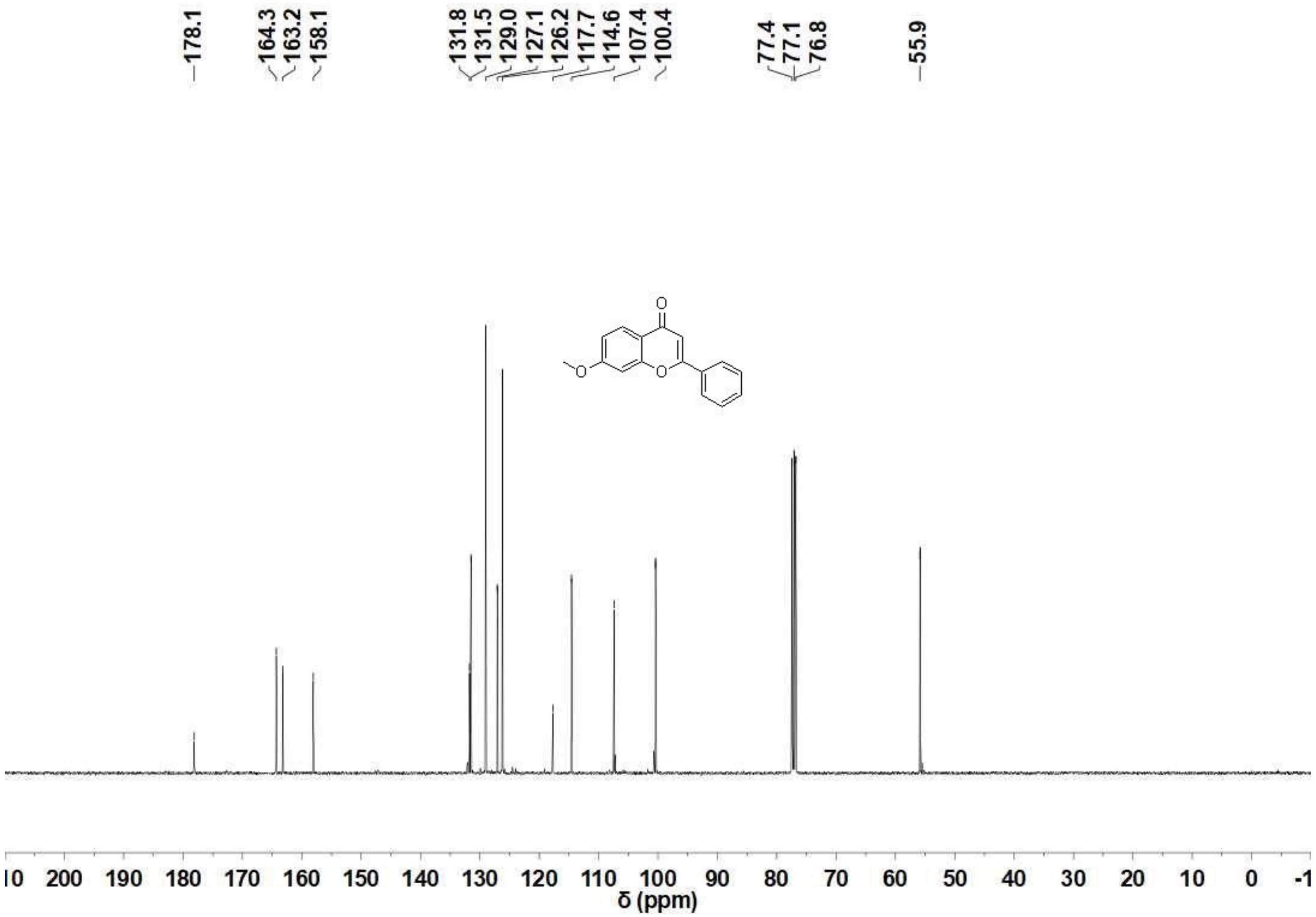

178.1
164.3
163.2
158.1
131.8
131.5
129.0
127.1
126.2
117.7
114.6
107.4
100.4
77.4
77.1
76.8
55.9
O
O
O
10
200
190
180
170
160
150
140
130
120
110
100
90
80
70
60
50
40
30
20
10
0
-1
δ (ppm)

7-chloro-2-phenyl-*4H*-chromen-4-one, **4ac**
400 MHz, $CDCl_3$

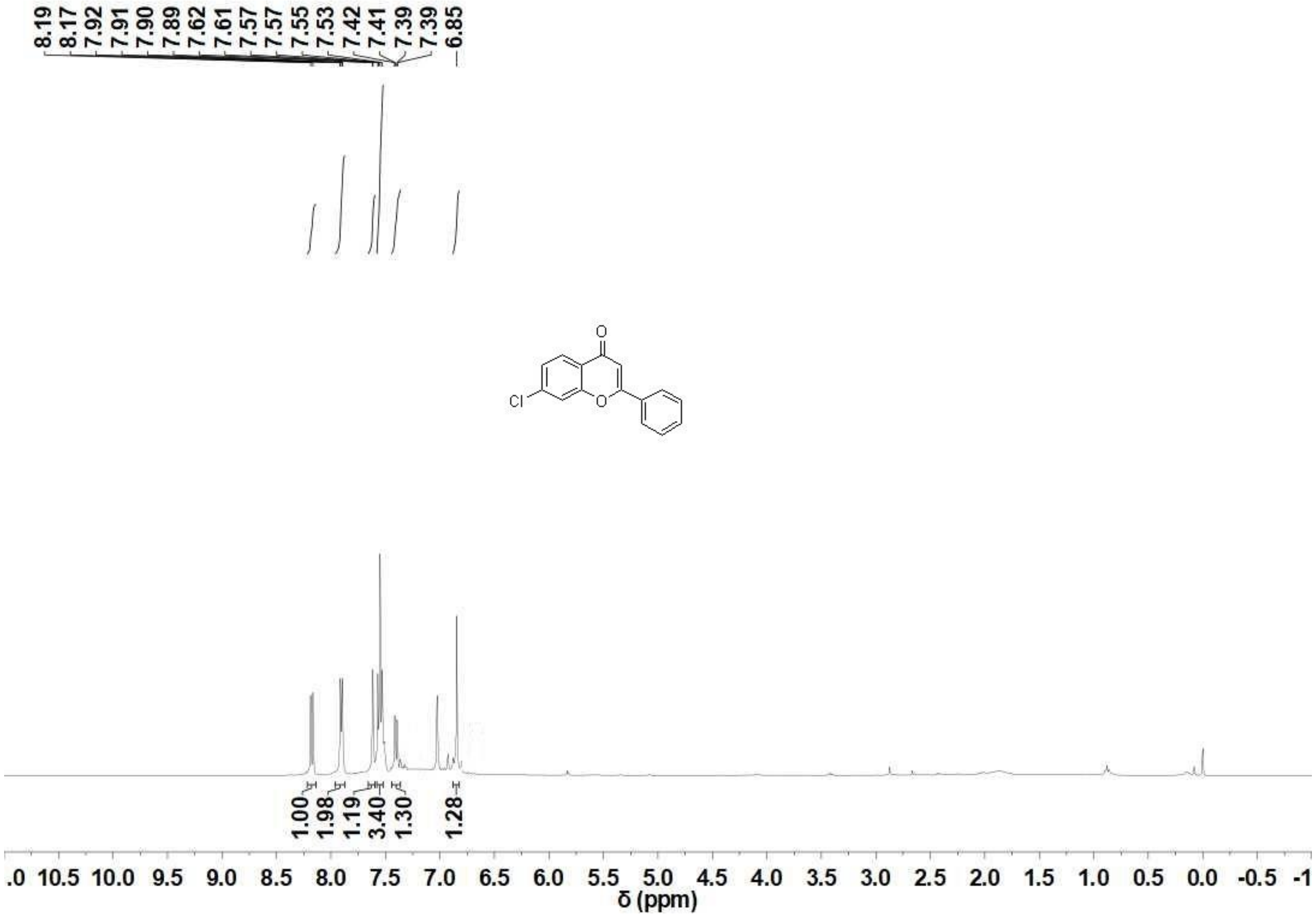

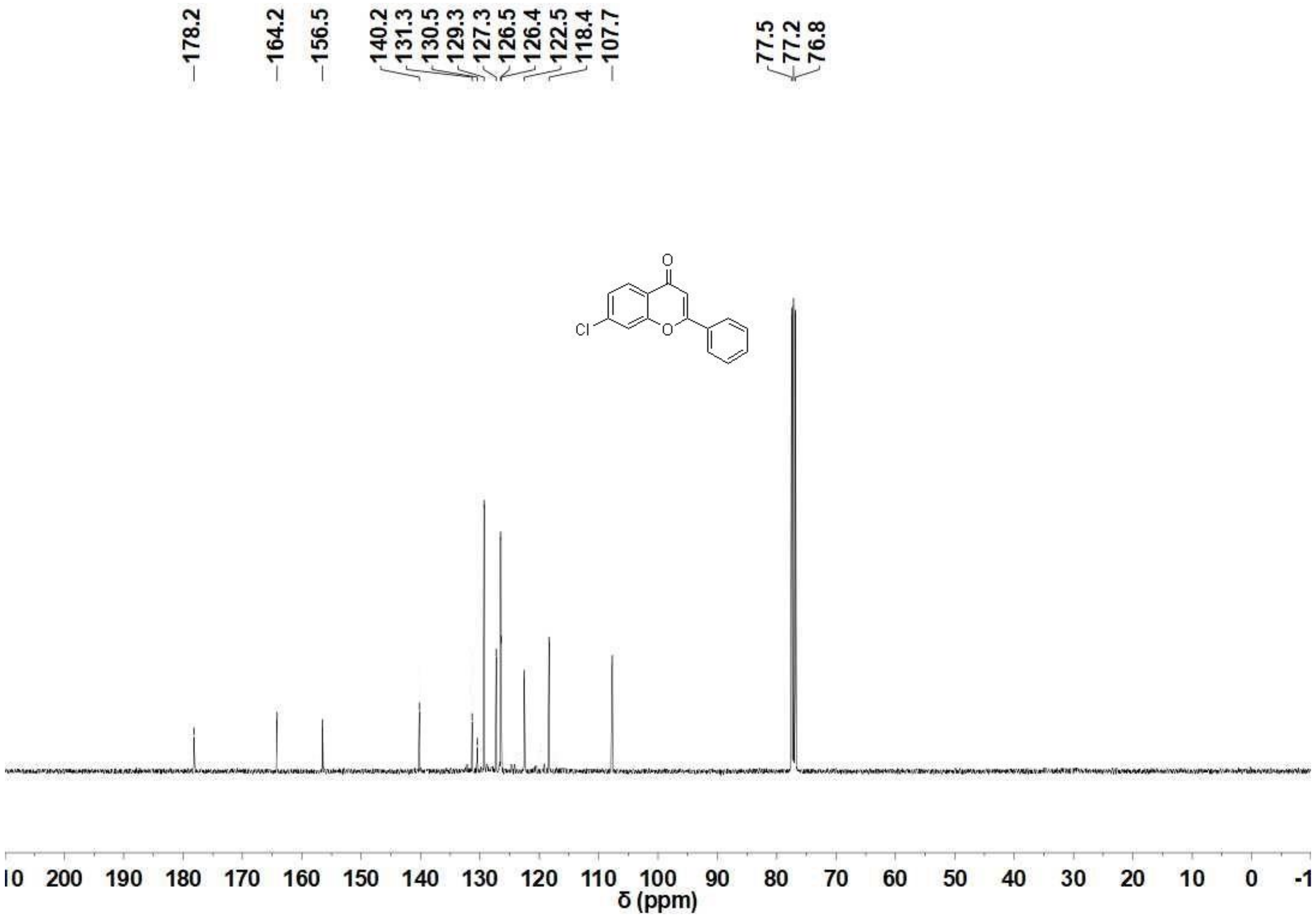
178.2
164.2
156.5
140.2
131.3
130.5
129.3
127.3
126.5
126.4
122.5
118.4
107.7
77.5
77.2
76.8
Cl
O
O
δ (ppm)

6-methyl-2-phenyl-*4H*-chromen-4-one, **4ad**
400 MHz, $CDCl_3$

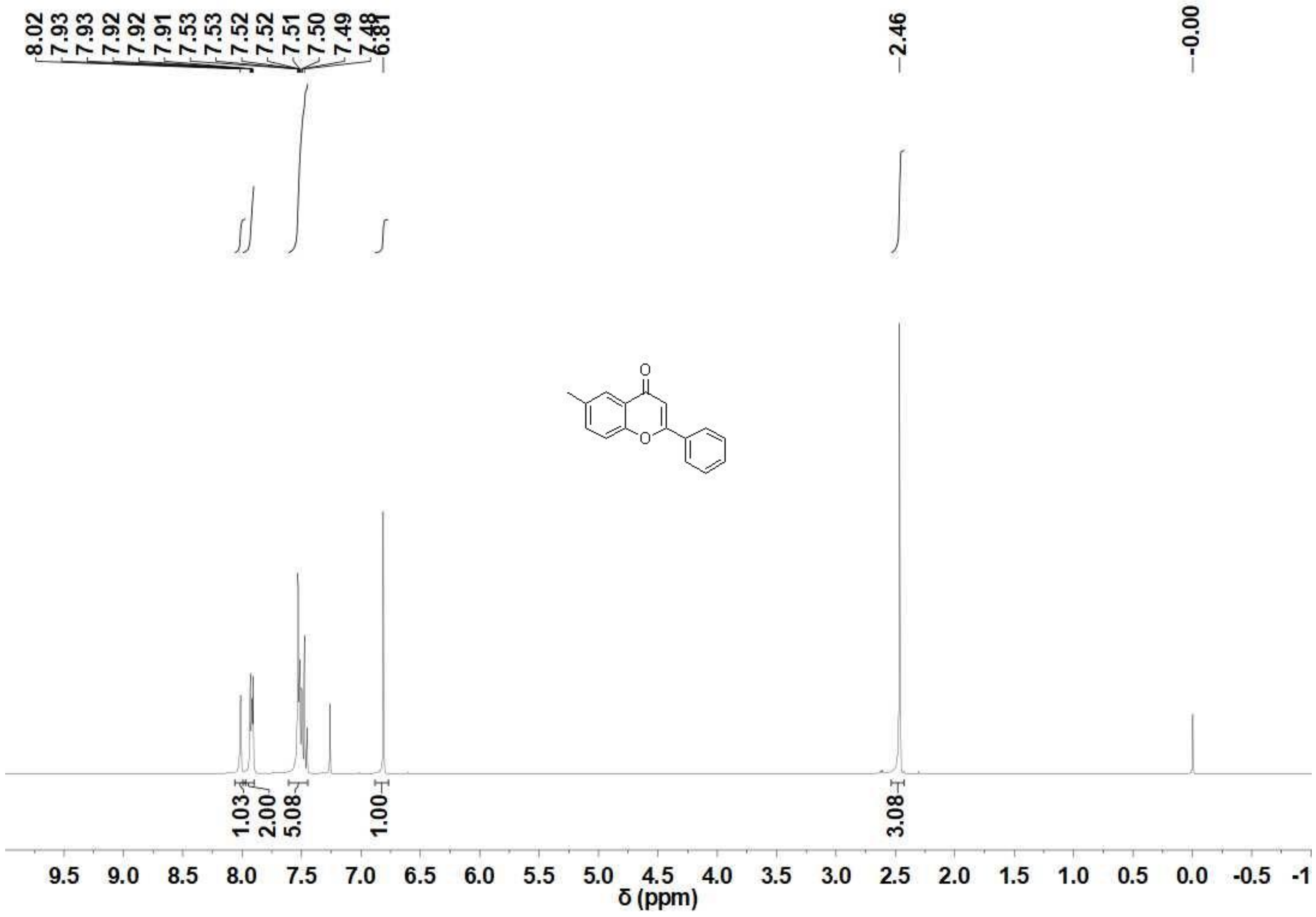

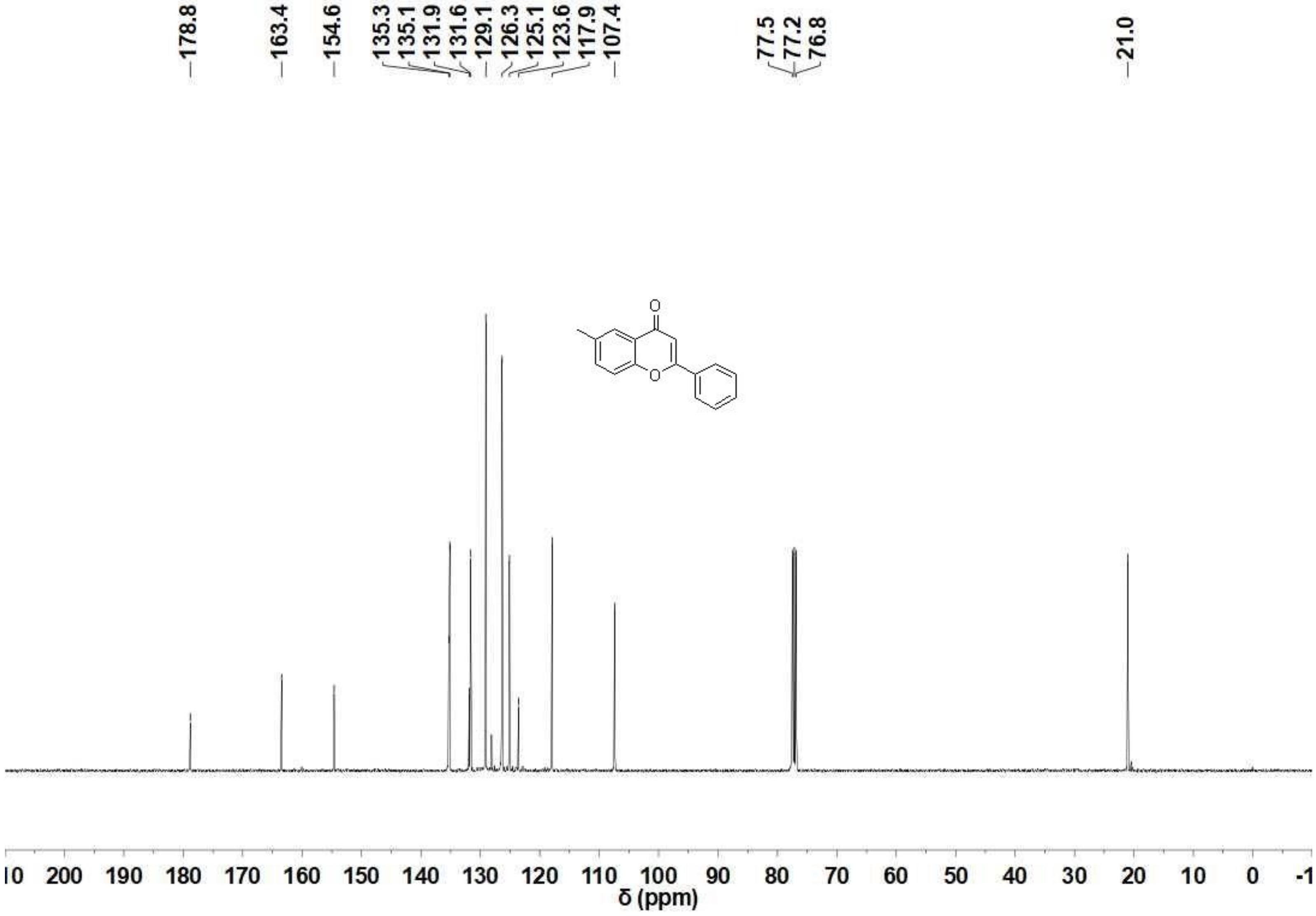
178.8
163.4
154.6
135.3
135.1
131.9
131.6
129.1
126.3
125.1
123.6
117.9
107.4
77.5
77.2
76.8
21.0
O
O
10
200
190
180
170
160
150
140
130
120
110
100
90
80
70
60
50
40
30
20
10
0
-1
δ (ppm)

6-fluoro-2-phenyl-*4H*-chromen-4-one, **4ae**
400 MHz, $CDCl_3$

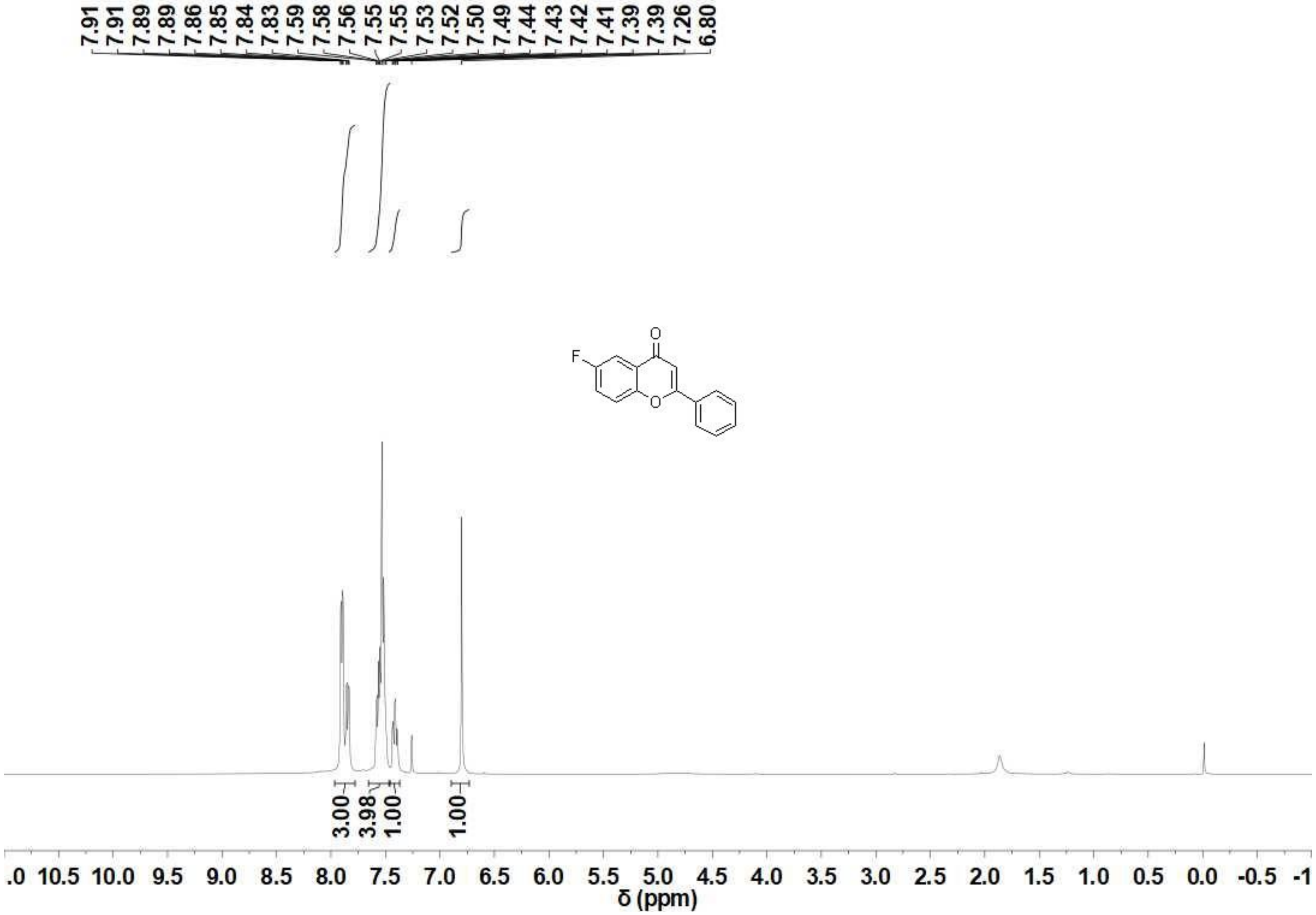

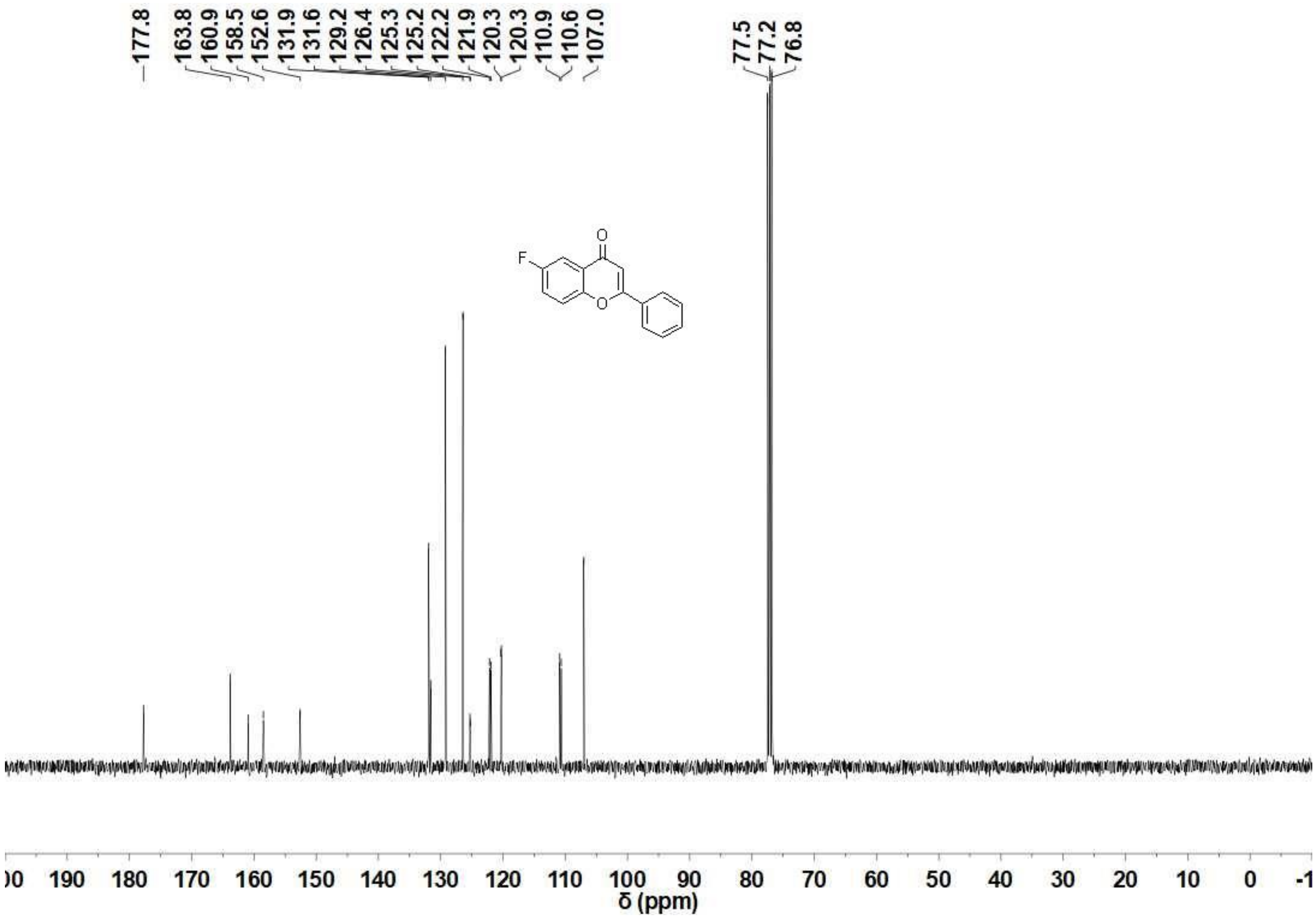
177.8
163.8
160.9
158.5
152.6
131.9
131.6
129.2
126.4
125.3
125.2
122.2
121.9
120.3
120.3
110.9
110.6
107.0
77.5
77.2
76.8
F
O
O
0 190 180 170 160 150 140 130 120 110 100 90 80 70 60 50 40 30 20 10 0 -1
δ (ppm)

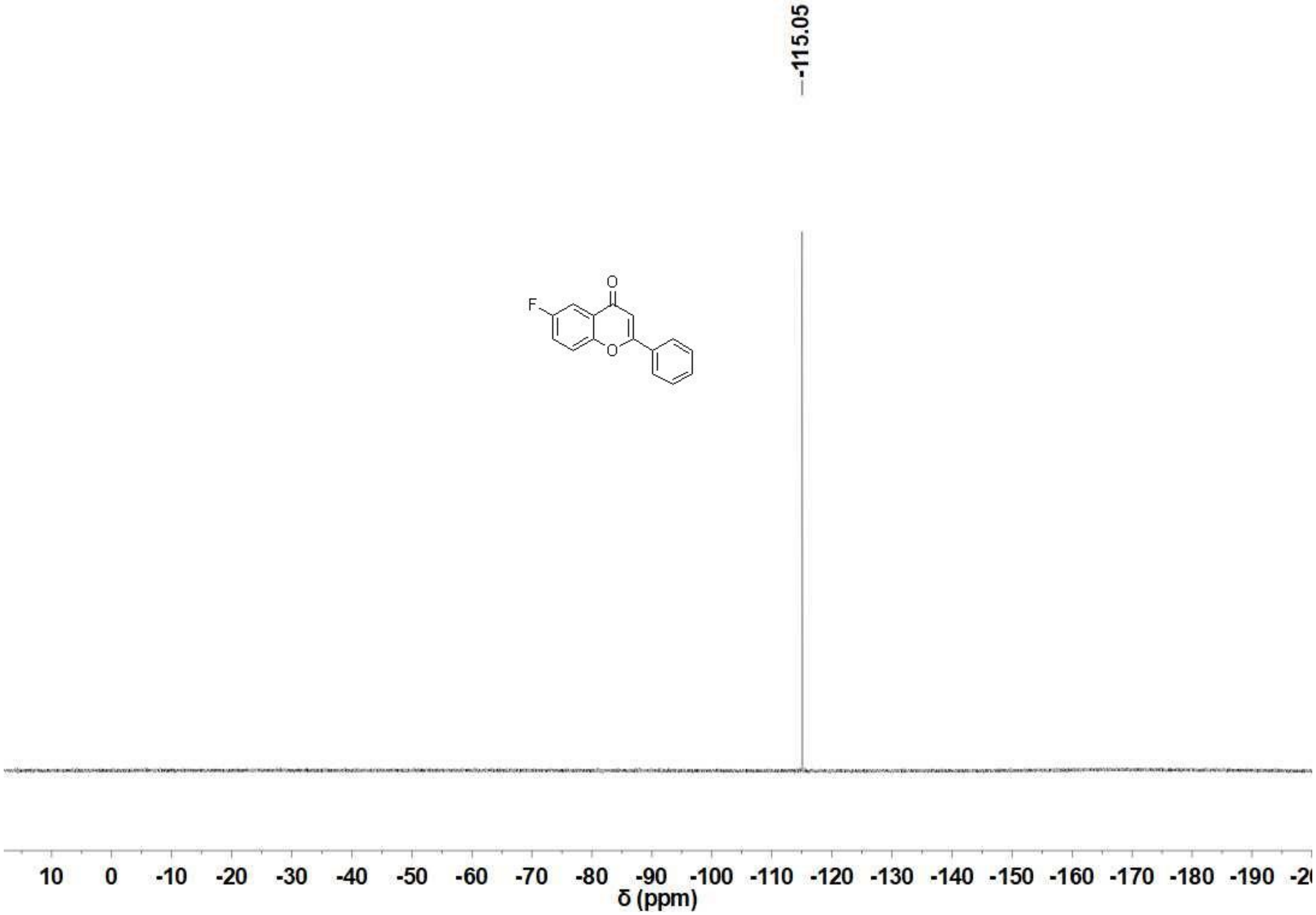

-115.05
F
O
O
10
0
-10
-20
-30
-40
-50
-60
-70
-80
-90
-100
-110
-120
-130
-140
-150
-160
-170
-180
-190
δ (ppm)

6-chloro-2-phenyl-*4H*-chromen-4-one, **4af**
400 MHz, $CDCl_3$

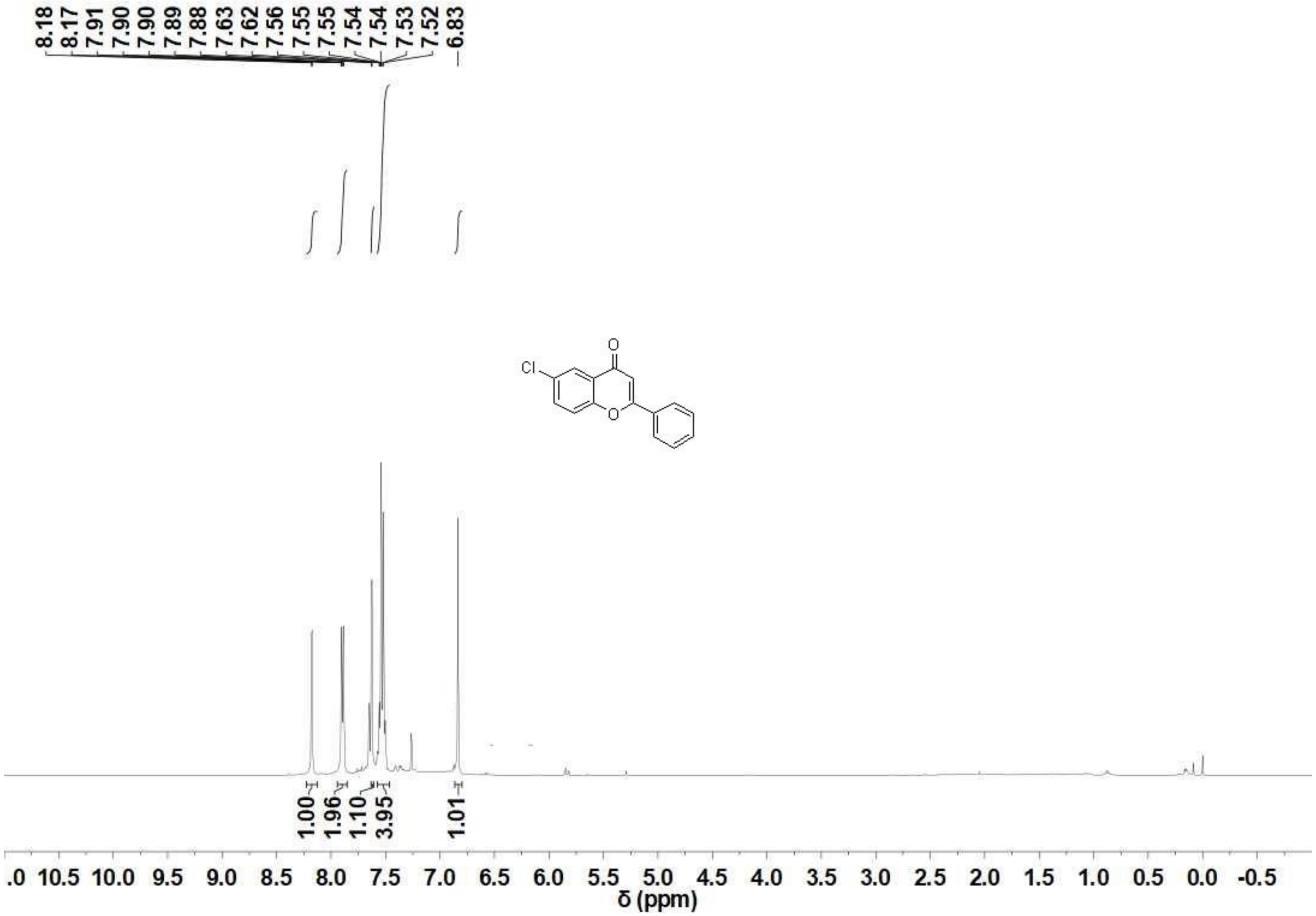

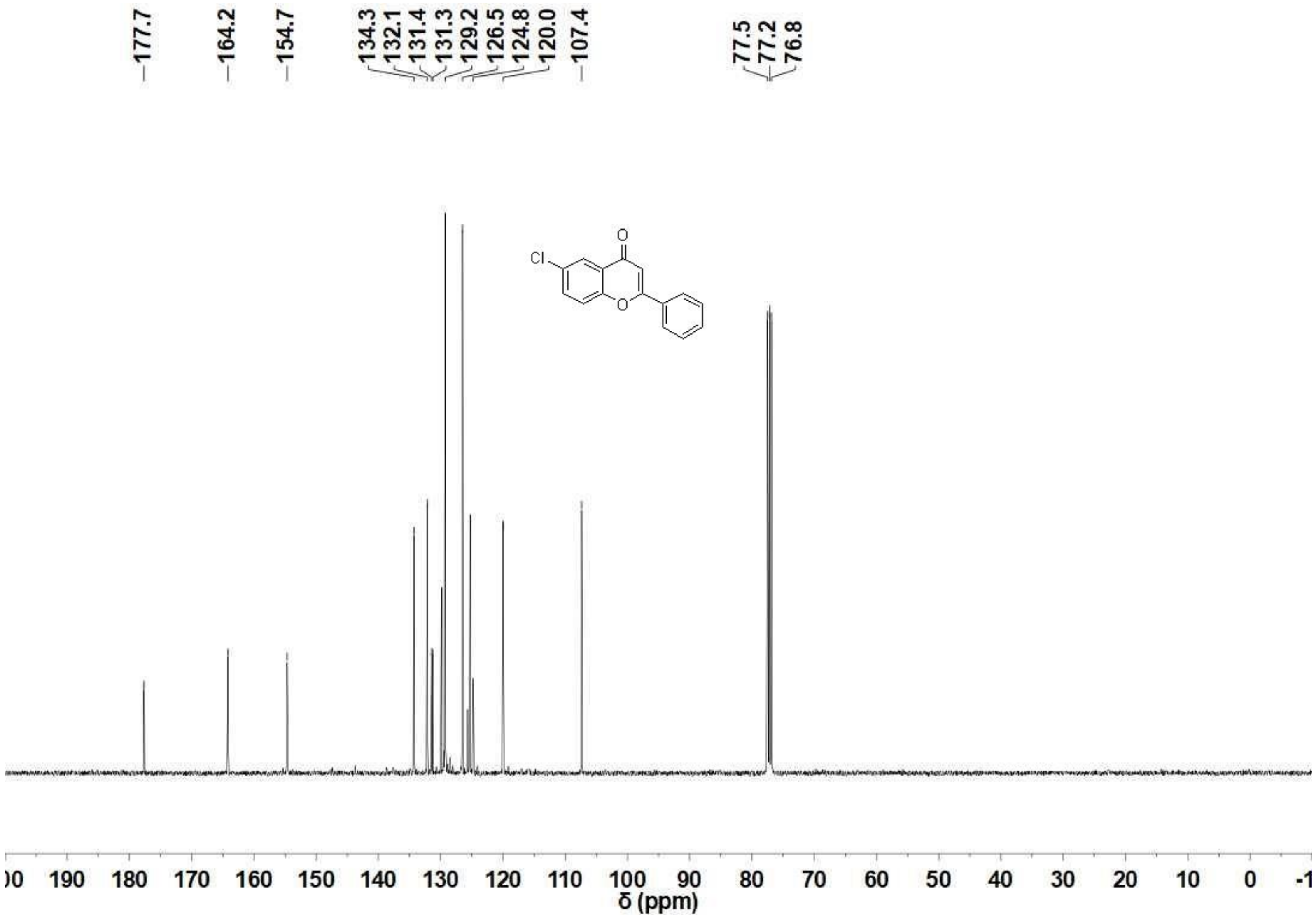
177.7
164.2
154.7
134.3
132.1
131.4
131.3
129.2
126.5
124.8
120.0
107.4
77.5
77.2
76.8
O
Cl
O
δ (ppm)

6-bromo-2-phenyl-*4H*-chromen-4-one, **4ag**

400 MHz, $CDCl_3$

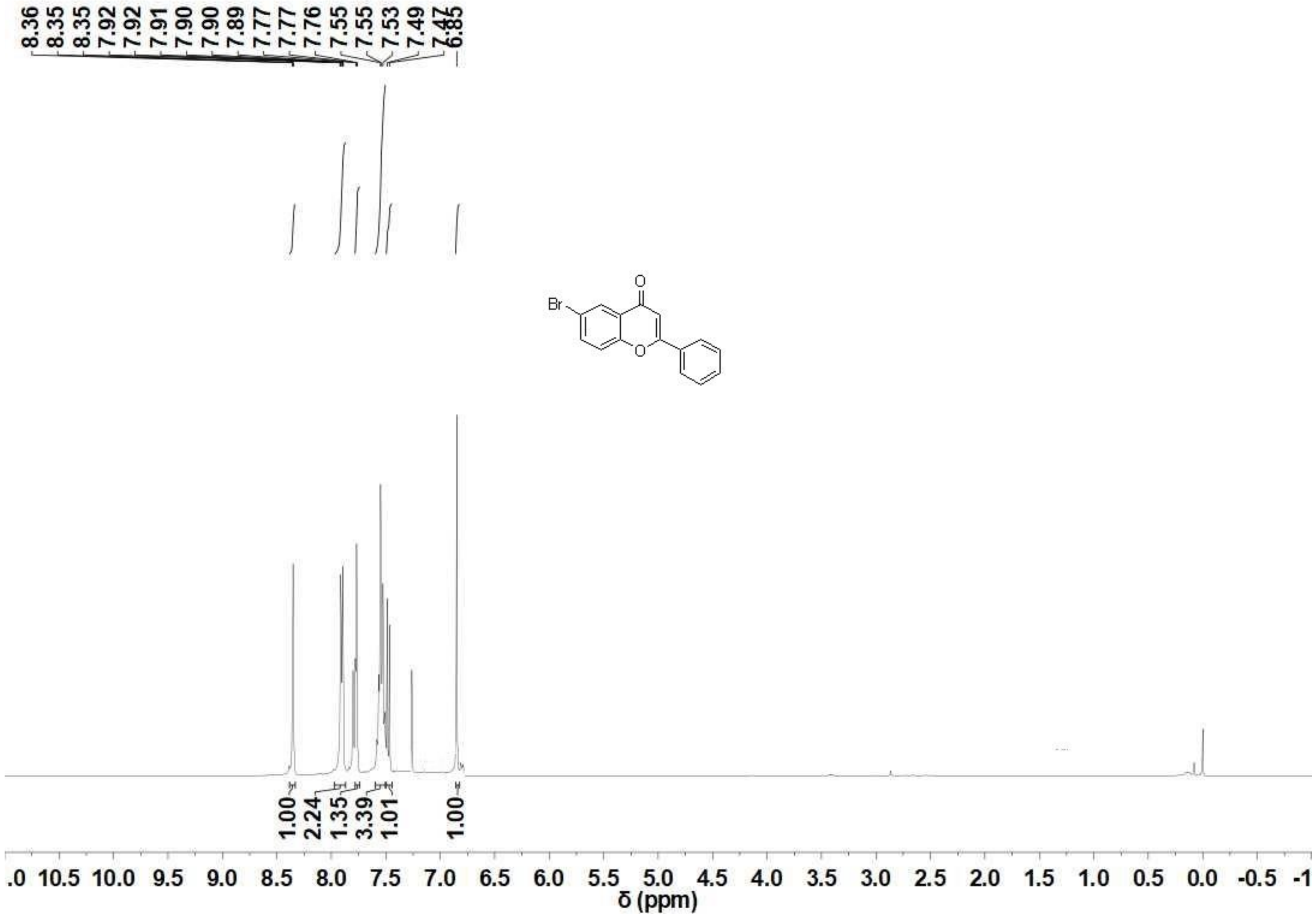

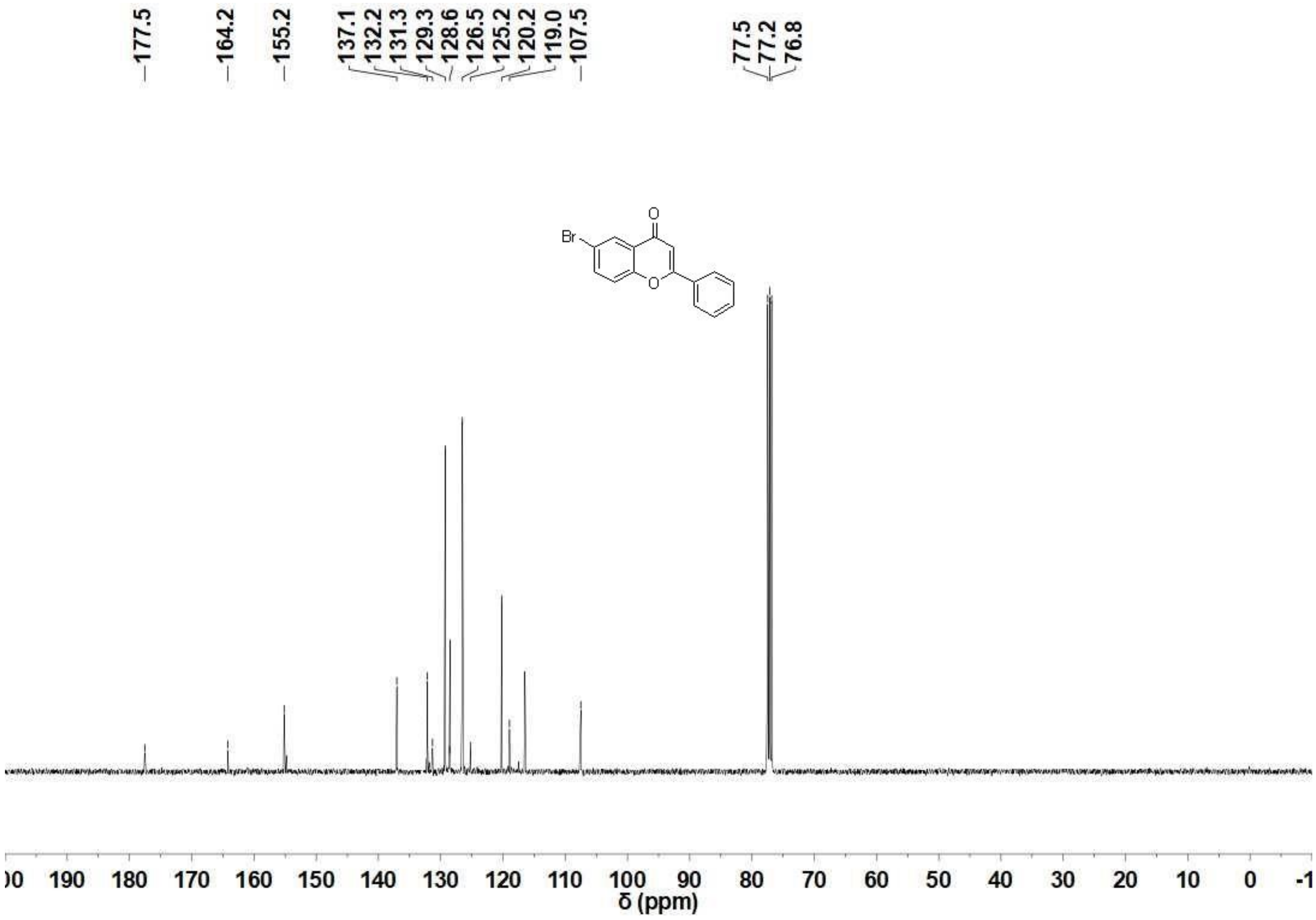
177.5
164.2
155.2
137.1
132.2
131.3
129.3
128.6
126.5
125.2
120.2
119.0
107.5
77.5
77.2
76.8
Br
O
O
190 180 170 160 150 140 130 120 110 100 90 80 70 60 50 40 30 20 10 0
δ (ppm)

methyl 4-oxo-2-phenyl-*4H*-chromene-6-carboxylate, **4ah**
400 MHz, $CDCl_3$

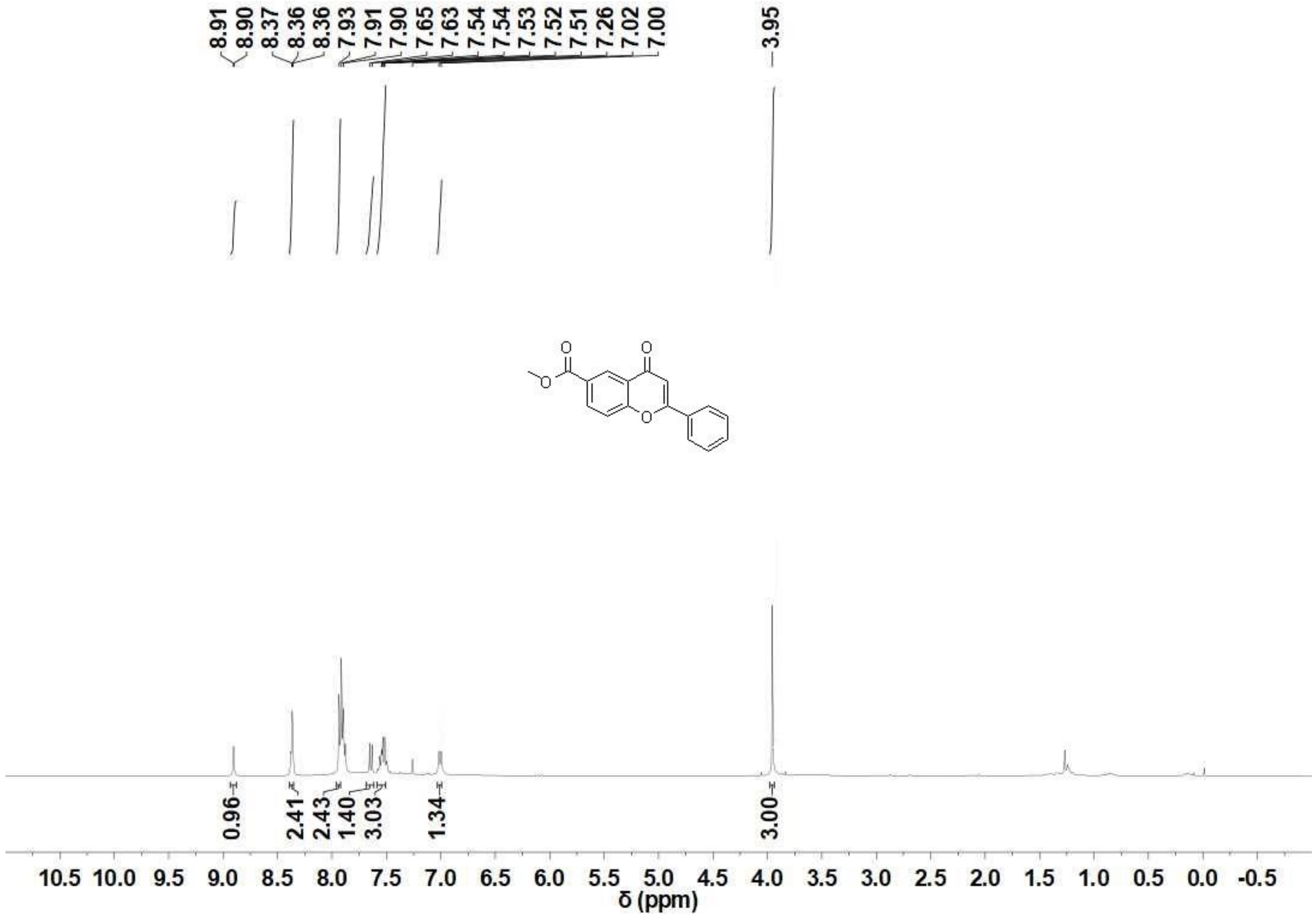

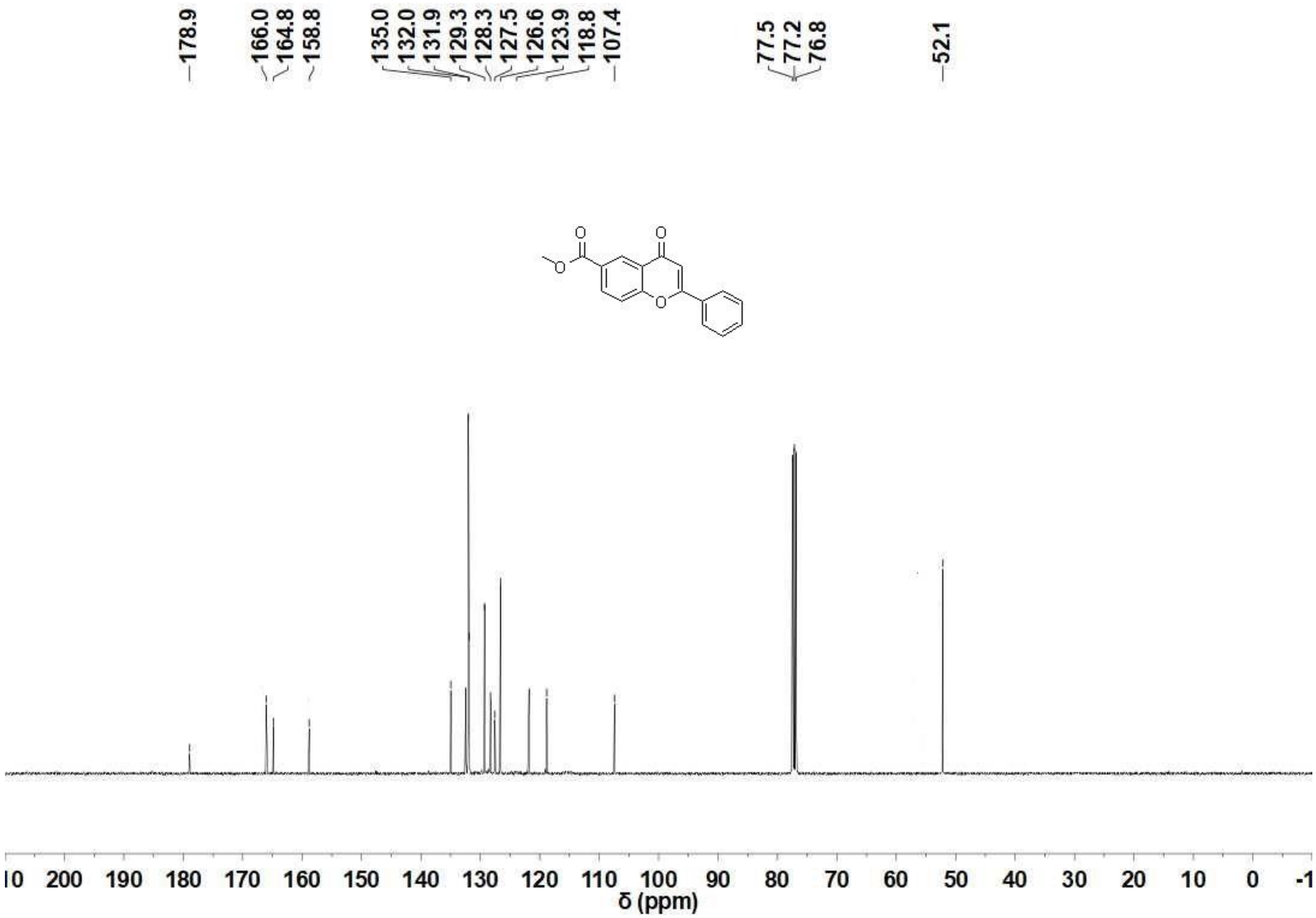
178.9
166.0
164.8
158.8
135.0
132.0
131.9
129.3
128.3
127.5
126.6
123.9
118.8
107.4
77.5
77.2
76.8
52.1
δ (ppm)

5-hydroxy-2-phenyl-*4H*-chromen-4-one, **4ai**
400 MHz, $CDCl_3$

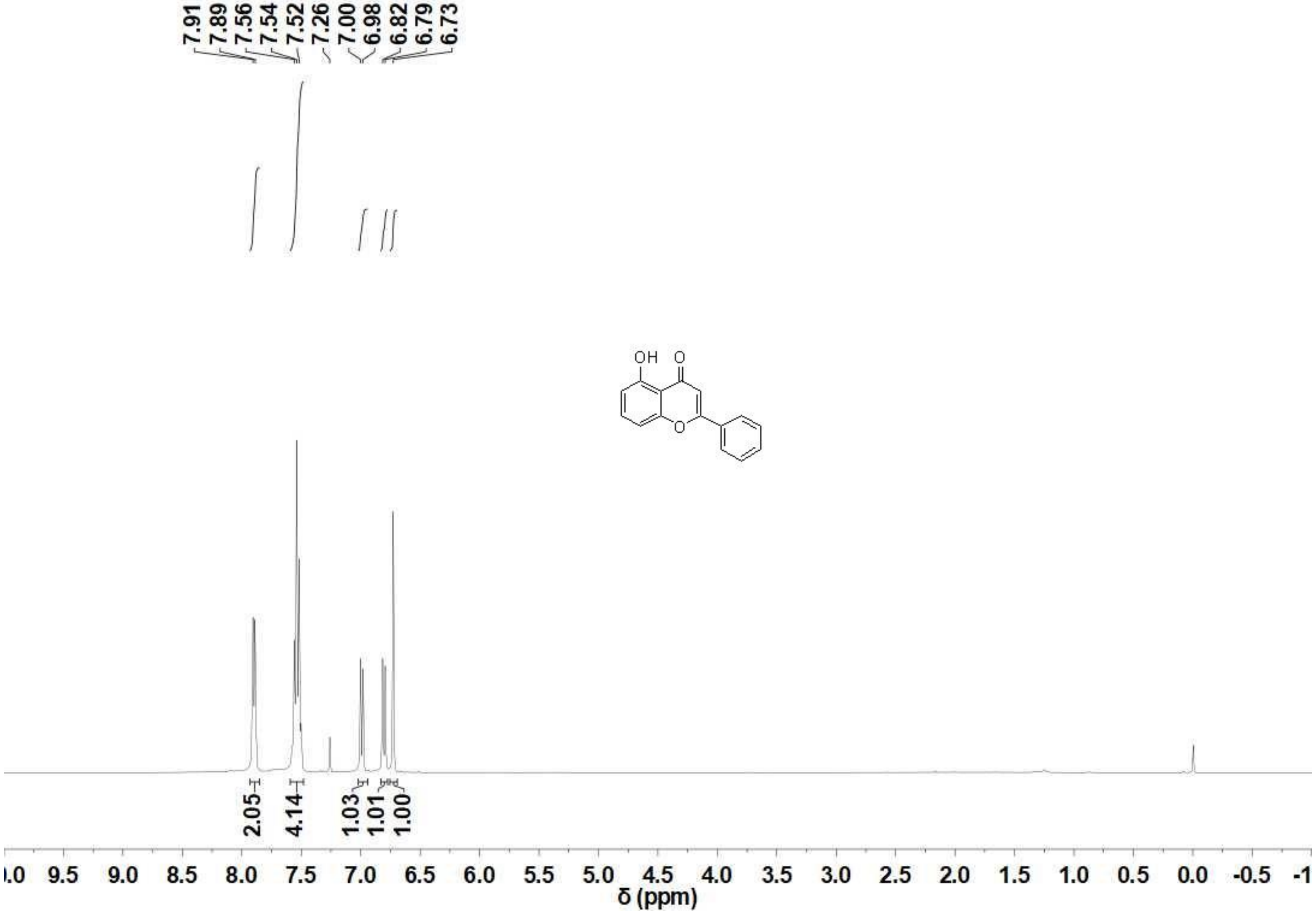

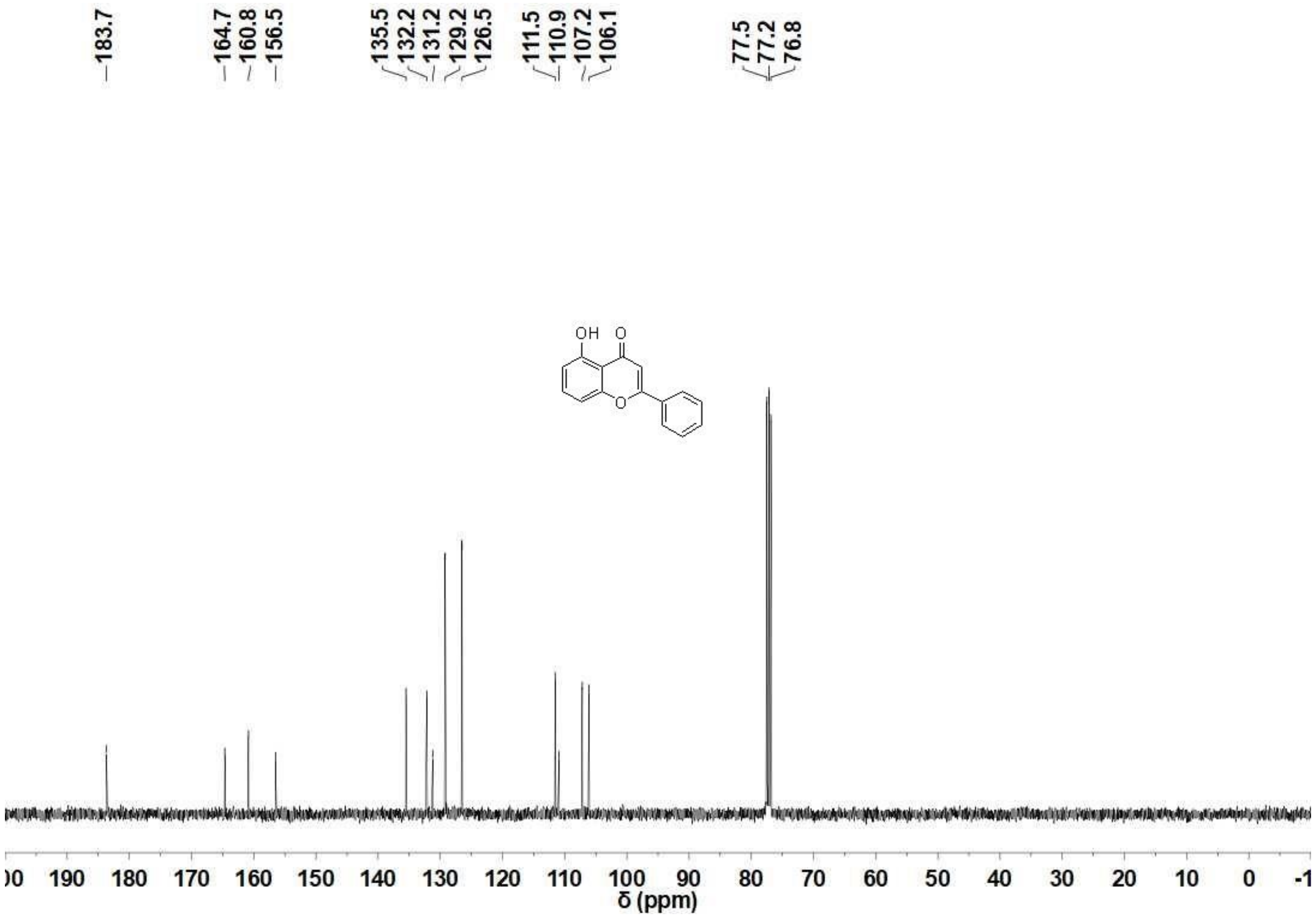
183.7
164.7
160.8
156.5
135.5
132.2
131.2
129.2
126.5
111.5
110.9
107.2
106.1
77.5
77.2
76.8
OH
O
O
190 180 170 160 150 140 130 120 110 100 90 80 70 60 50 40 30 20 10 0
δ (ppm)

3-methyl-2-phenyl-*4H*-chromen-4-one, **5aa**
400 MHz, $CDCl_3$

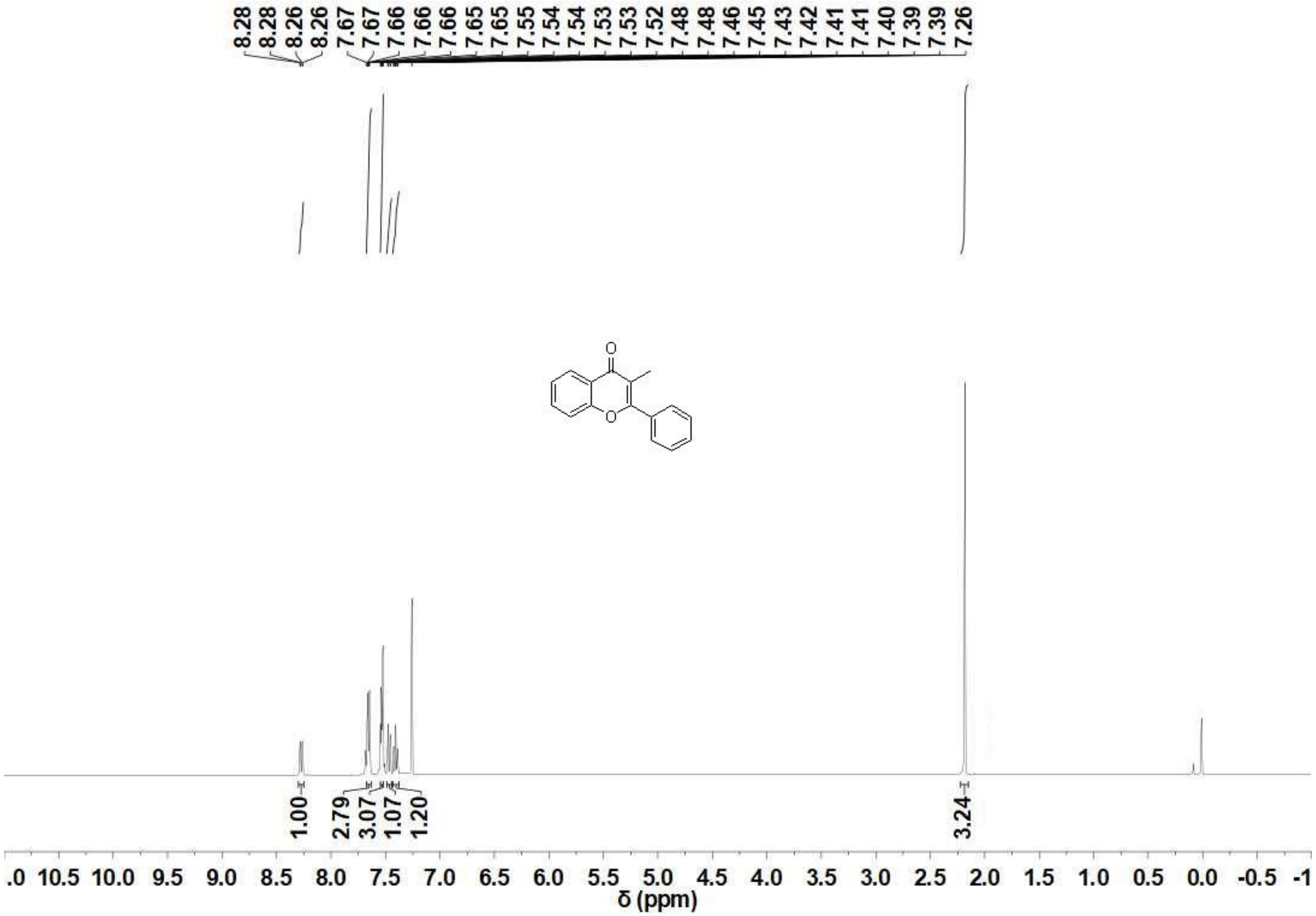

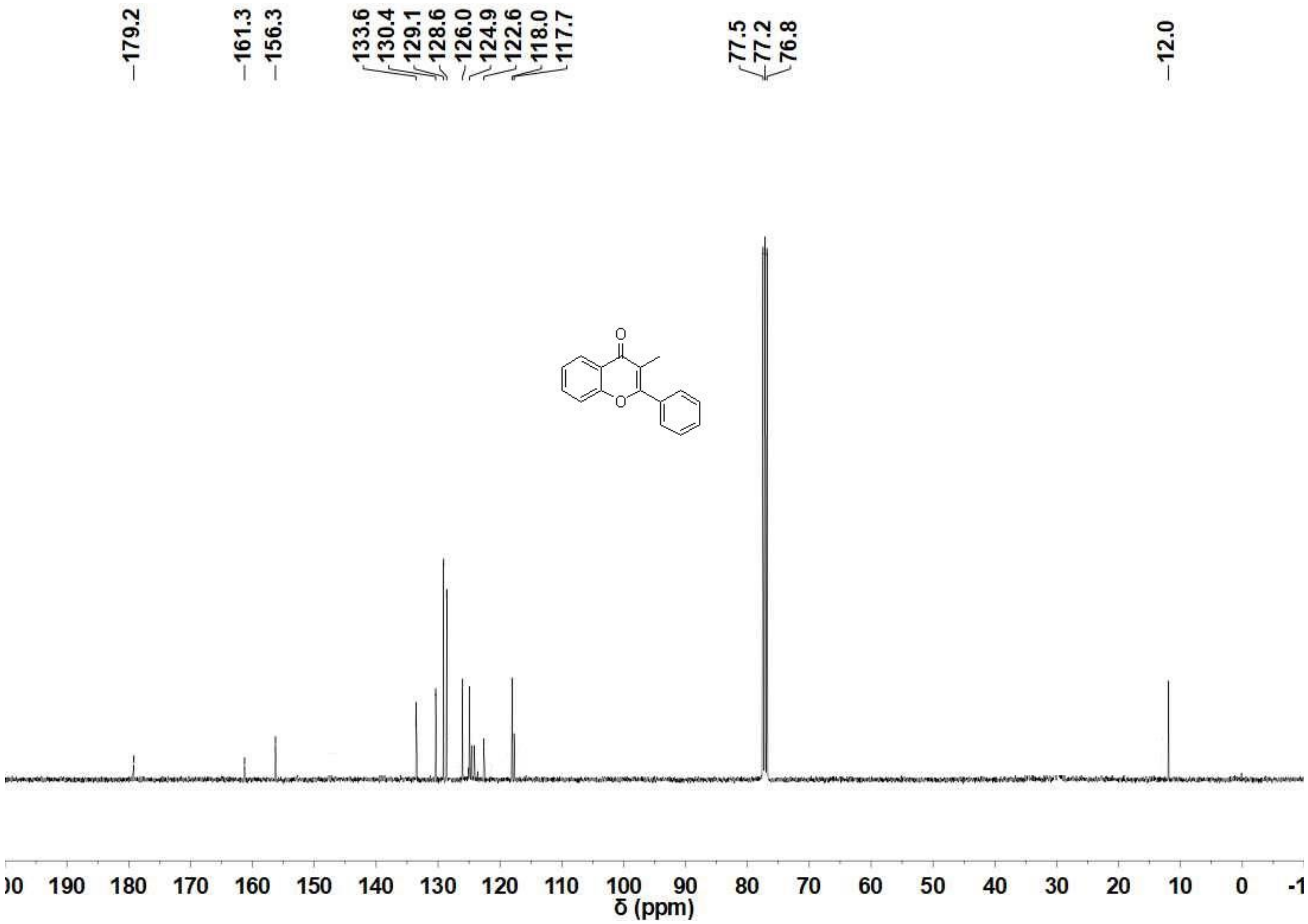
179.2
161.3
156.3
133.6
130.4
129.1
128.6
126.0
124.9
122.6
118.0
117.7
77.5
77.2
76.8
12.0
O
O
δ (ppm)

3-ethyl-2-phenyl-*4H*-chromen-4-one, **5ab**
400 MHz, $CDCl_3$

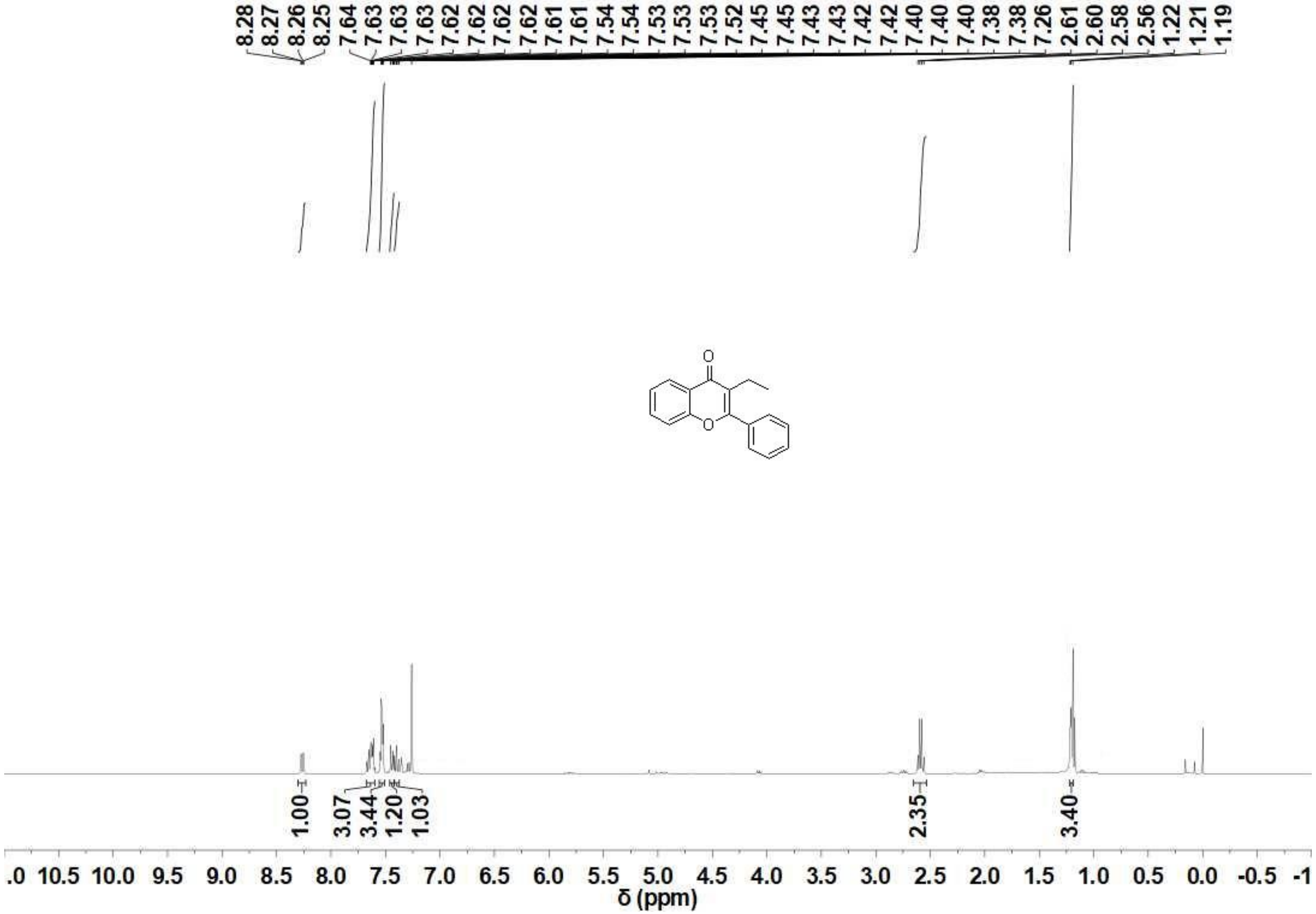

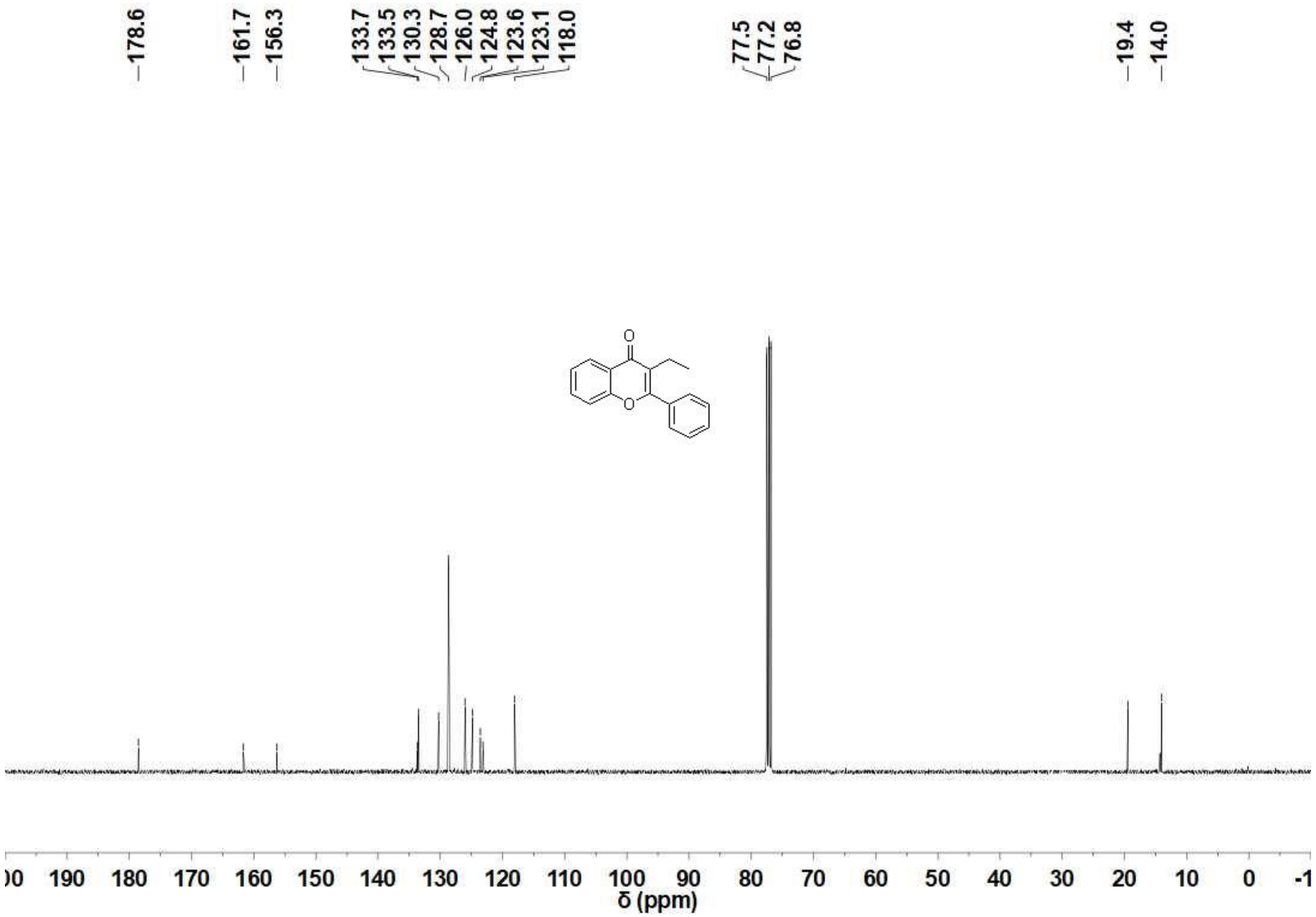
178.6
161.7
156.3
133.7
133.5
130.3
128.7
126.0
124.8
123.6
123.1
118.0
77.5
77.2
76.8
19.4
14.0
O
O
0 190 180 170 160 150 140 130 120 110 100 90 80 70 60 50 40 30 20 10 0 -1
δ (ppm)

3-benzyl-2-phenyl-*4H*-chromen-4-one, **5ac**
400 MHz, $CDCl_3$

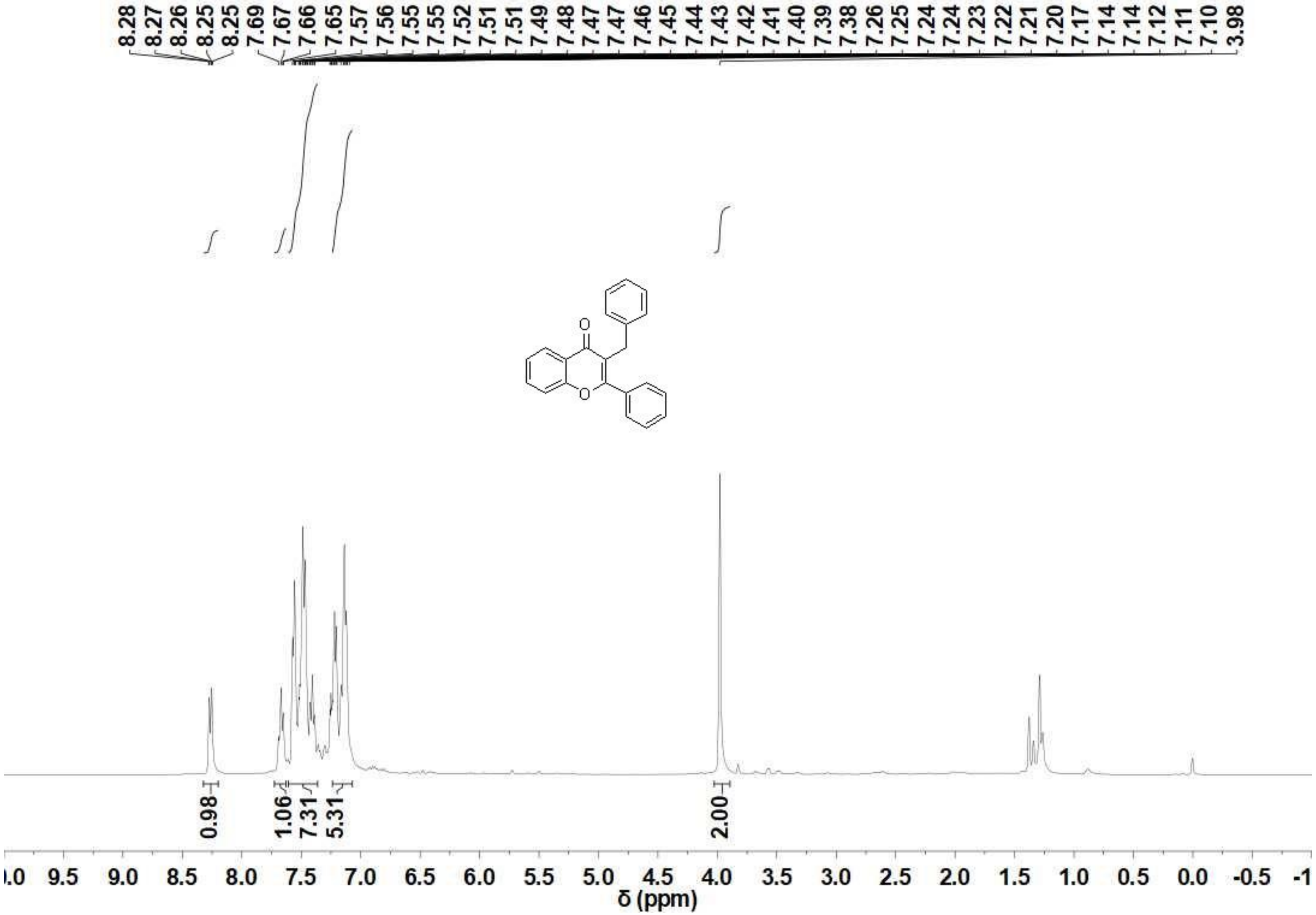

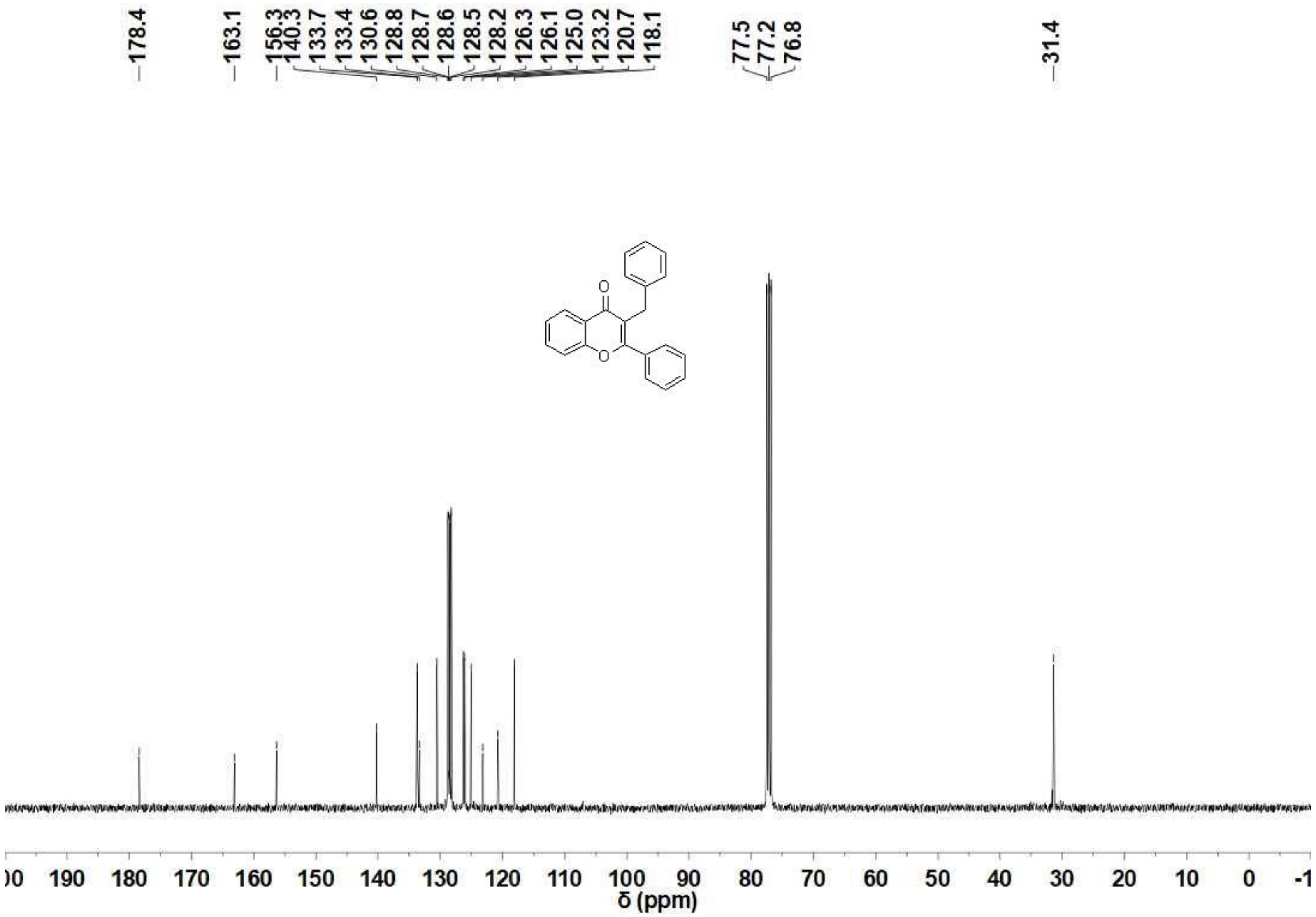
178.4
163.1
156.3
140.3
133.7
133.4
130.6
128.8
128.7
128.6
128.5
128.2
126.3
126.1
125.0
123.2
120.7
118.1
77.5
77.2
76.8
31.4
O
O
)0 190 180 170 160 150 140 130 120 110 100 90 80 70 60 50 40 30 20 10 0 -1
δ (ppm)

3-methyl-2-(*p*-tolyl)-*4H*-chromen-4-one, **5ia**
400 MHz, $CDCl_3$

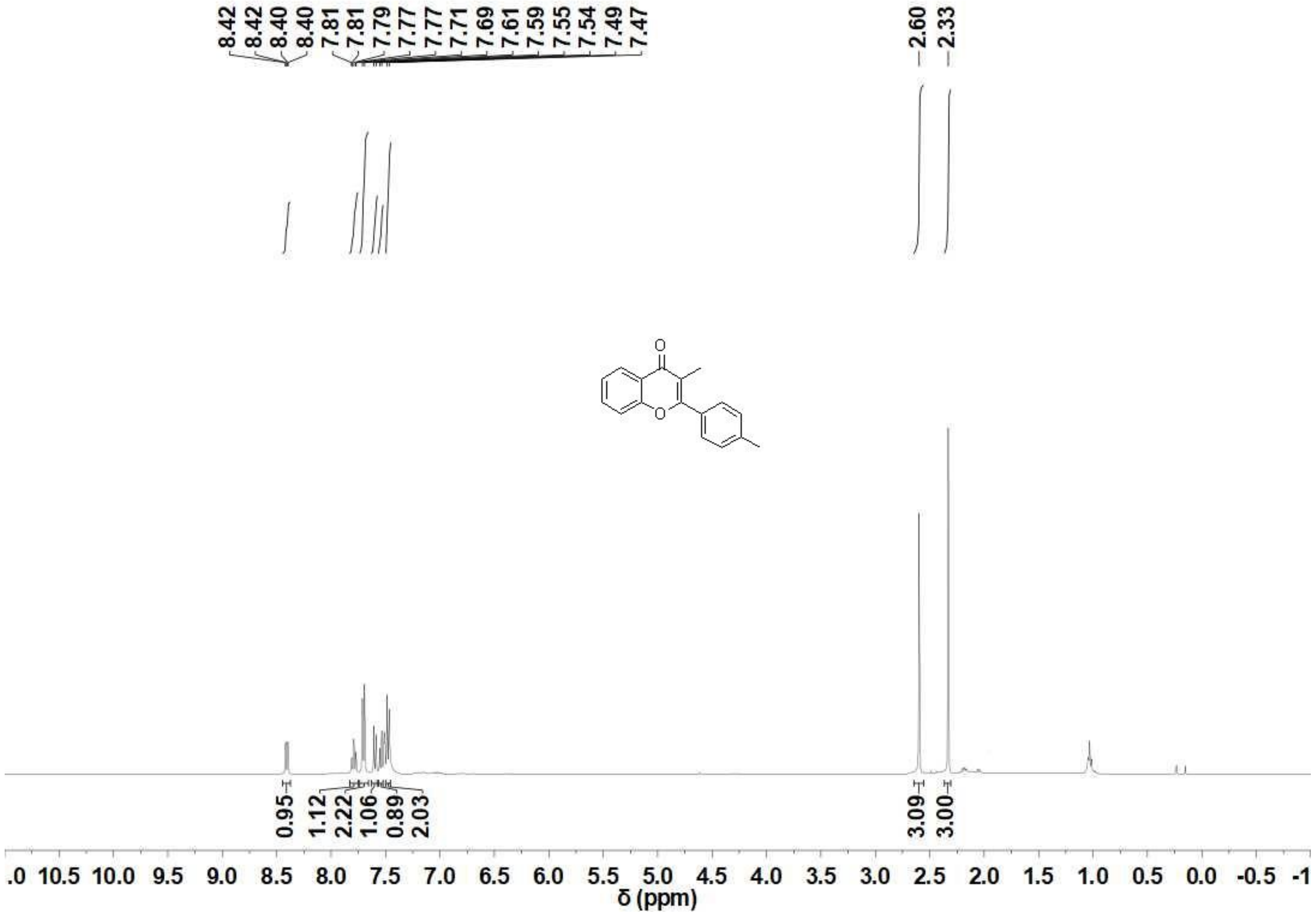

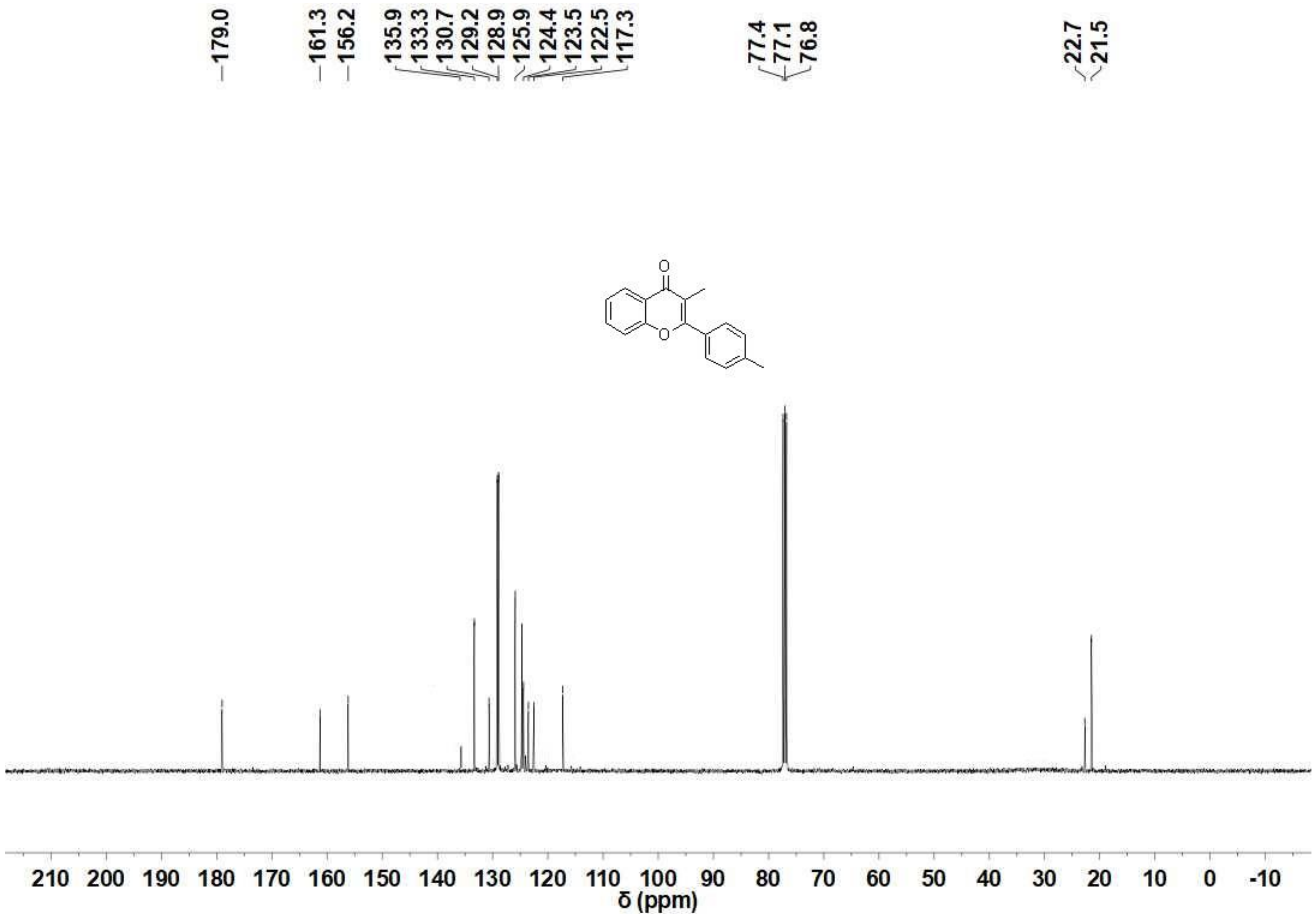
179.0
161.3
156.2
135.9
133.3
130.7
129.2
128.9
125.9
124.4
123.5
122.5
117.3
77.4
77.1
76.8
22.7
21.5
O
210 200 190 180 170 160 150 140 130 120 110 100 90 80 70 60 50 40 30 20 10 0 -10
δ (ppm)

3-benzyl-2-(*p-tolyl*)-*4H*-chromen-4-one, **5ic**
400 MHz, $CDCl_3$

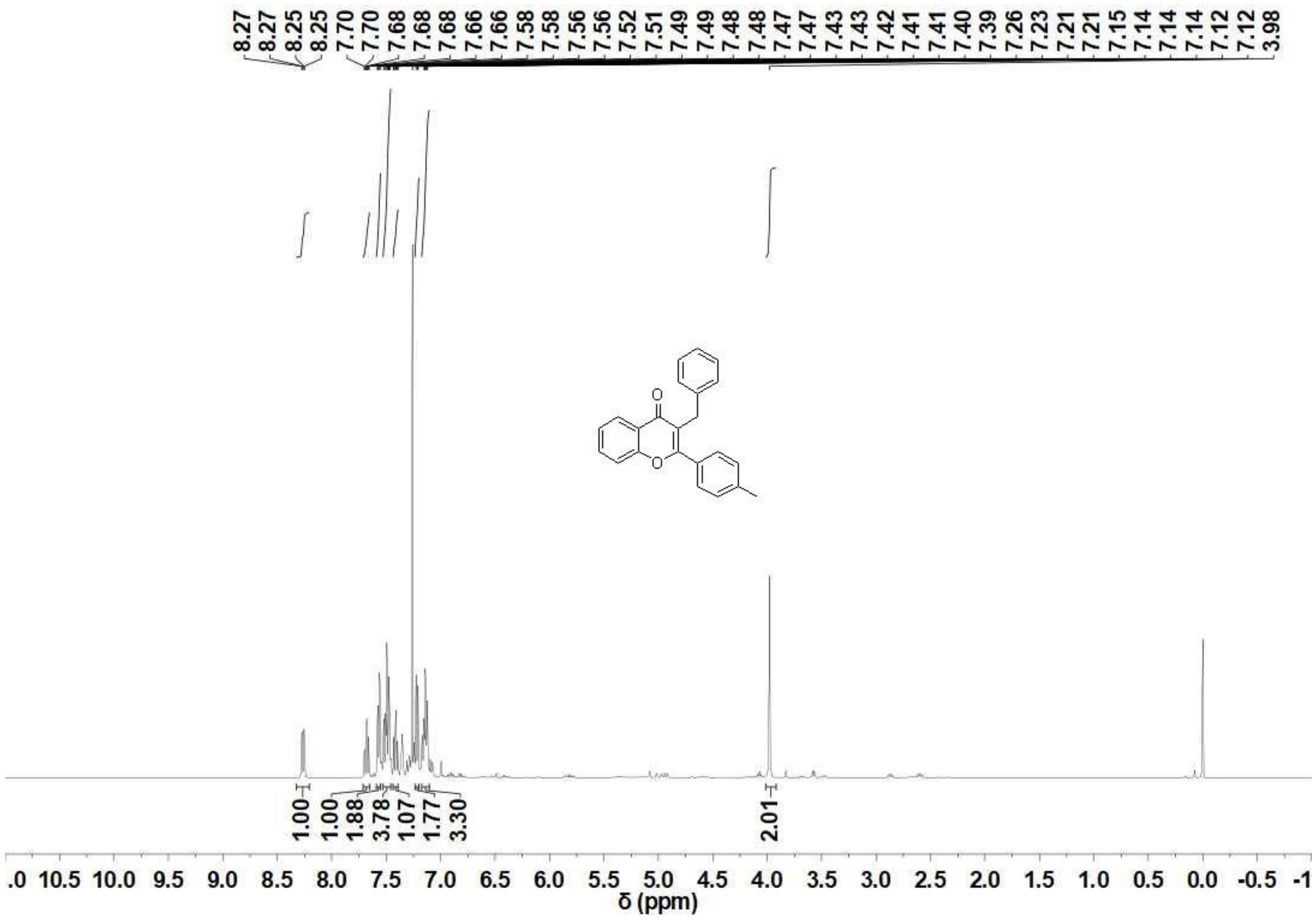

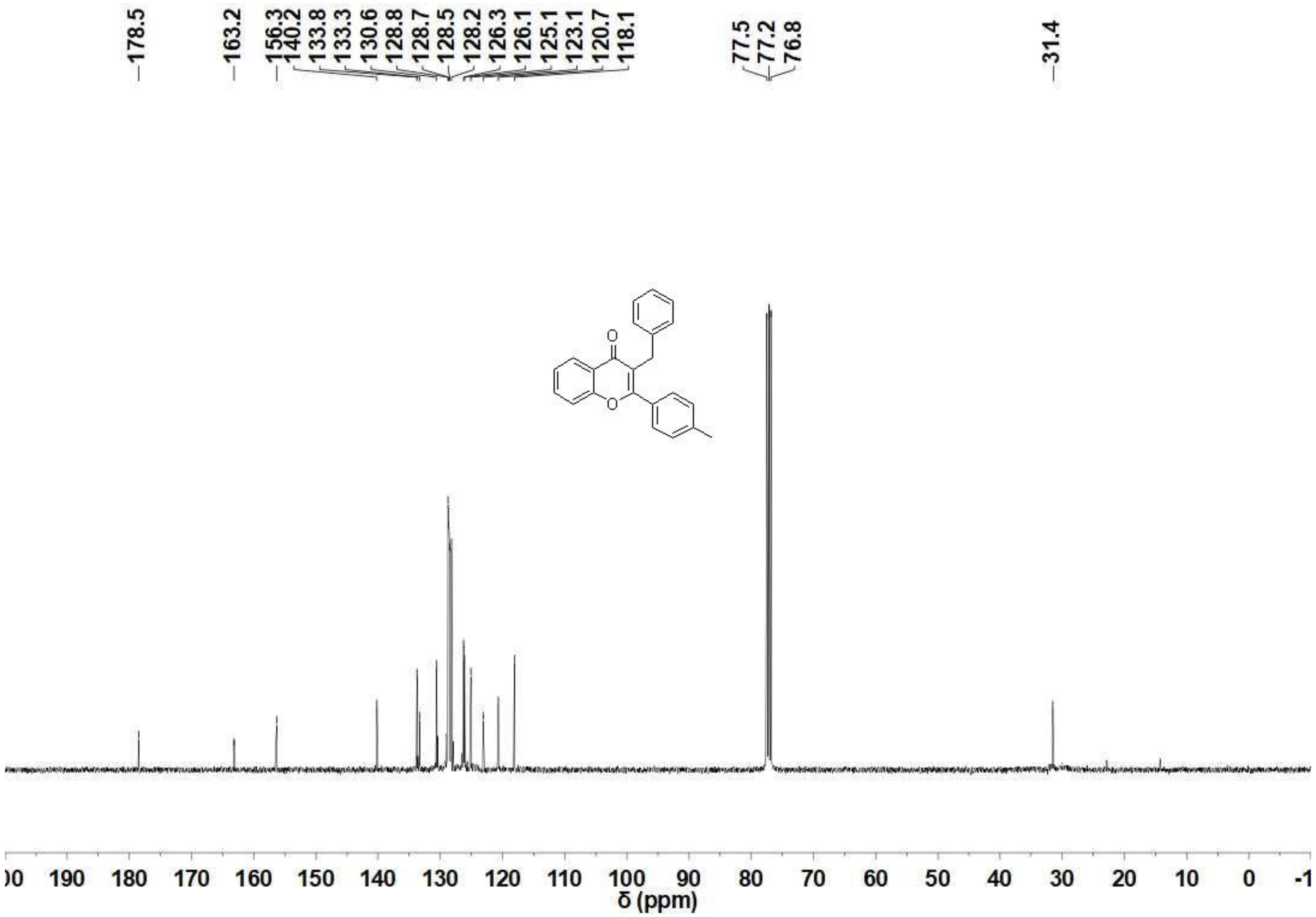

178.5
163.2
156.3
140.2
133.8
133.3
130.6
128.8
128.7
128.5
128.2
126.3
126.1
125.1
123.1
120.7
118.1
77.5
77.2
76.8
31.4
O
O
)0 190 180 170 160 150 140 130 120 110 100 90 80 70 60 50 40 30 20 10 0 -1
δ (ppm)

3-methyl-2-(*m*-tolyl)-*4H*-chromen-4-one, **5ea**
400 MHz, $CDCl_3$

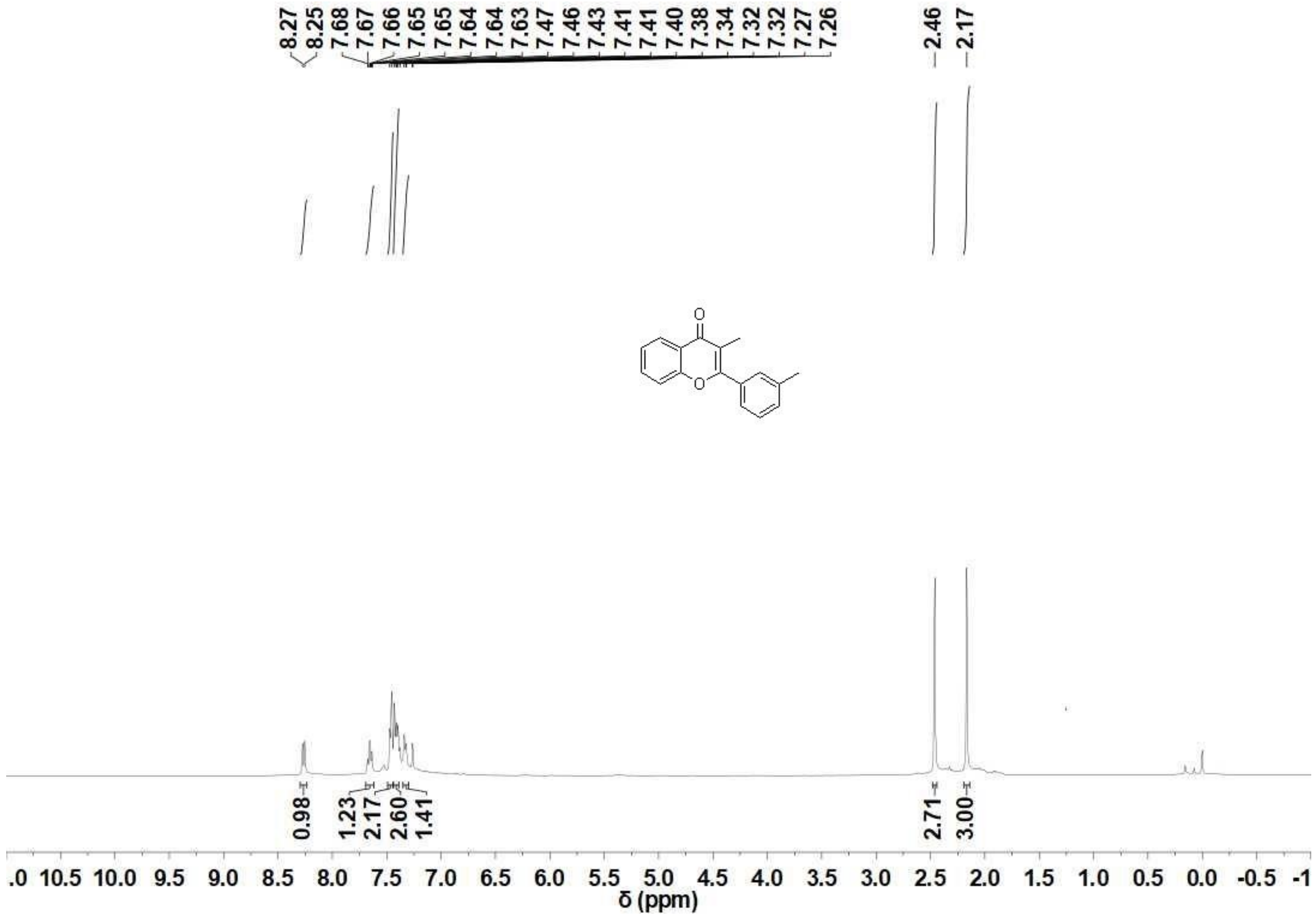

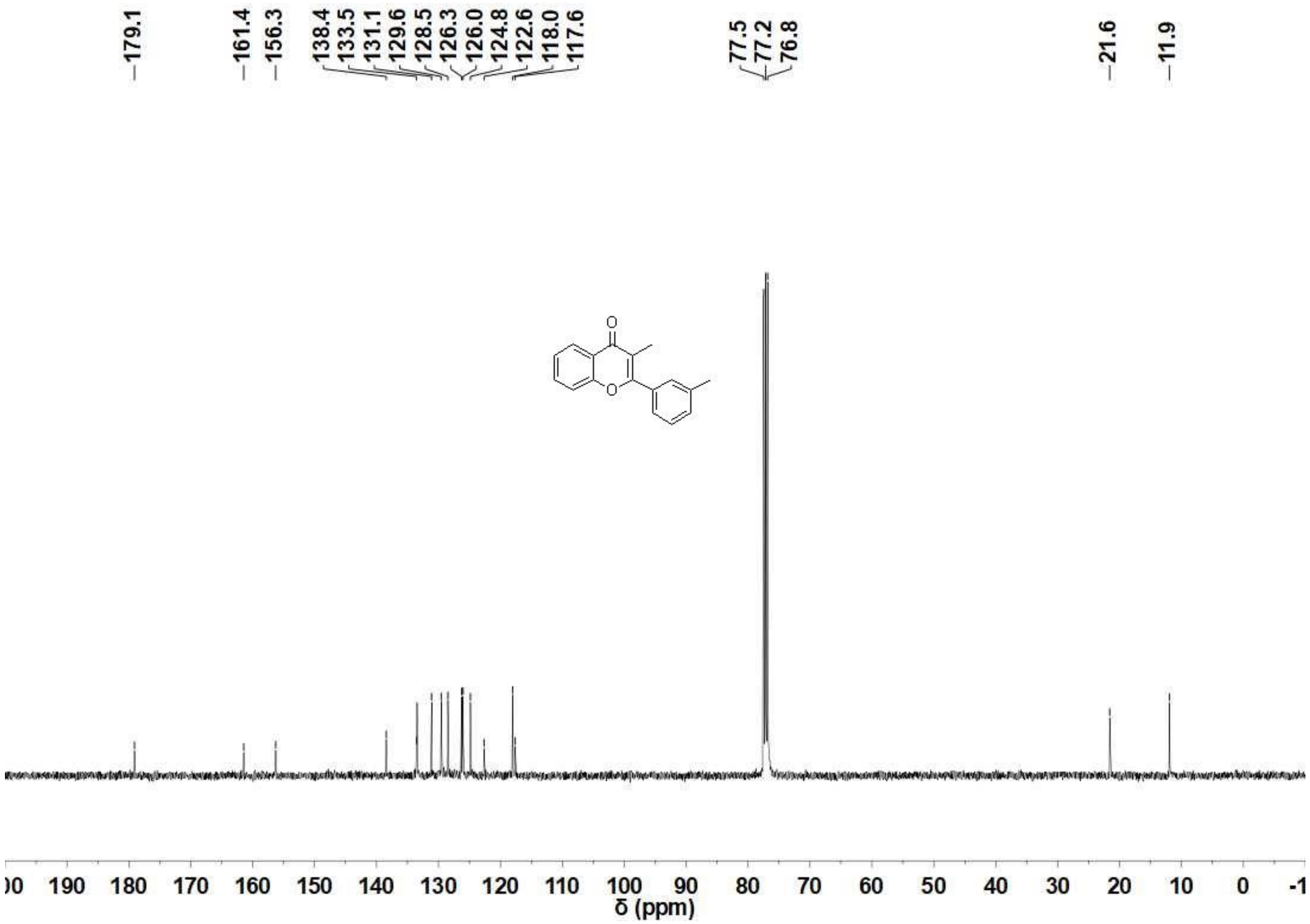
179.1
161.4
156.3
138.4
133.5
131.1
129.6
128.5
126.3
126.0
124.8
122.6
118.0
117.6
77.5
77.2
76.8
21.6
11.9
O
O
00 190 180 170 160 150 140 130 120 110 100 90 80 70 60 50 40 30 20 10 0 -1
δ (ppm)

2-(4-methoxyphenyl)-3-methyl-4H-chromen-4-one, **5lb**
400 MHz, $CDCl_3$

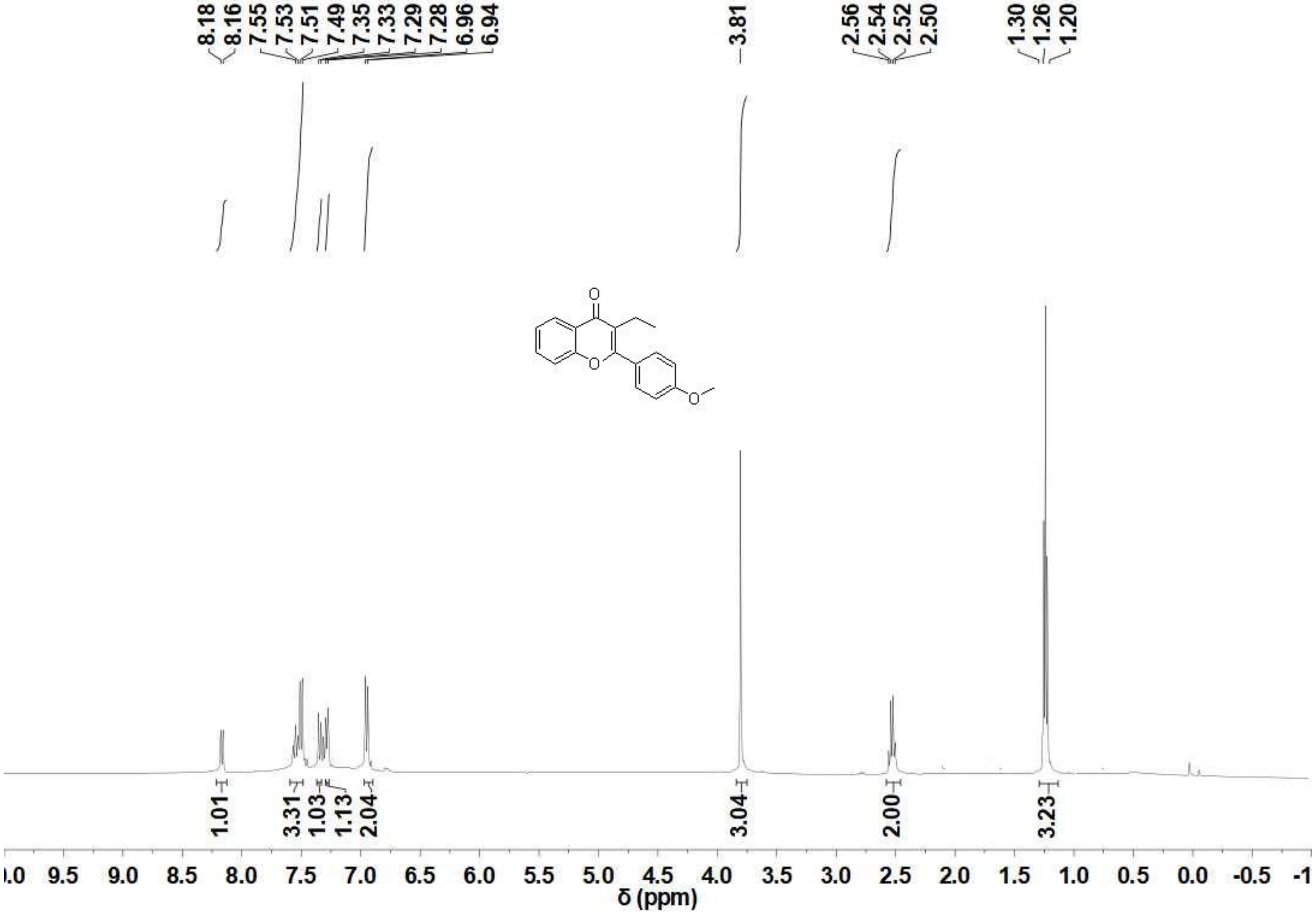

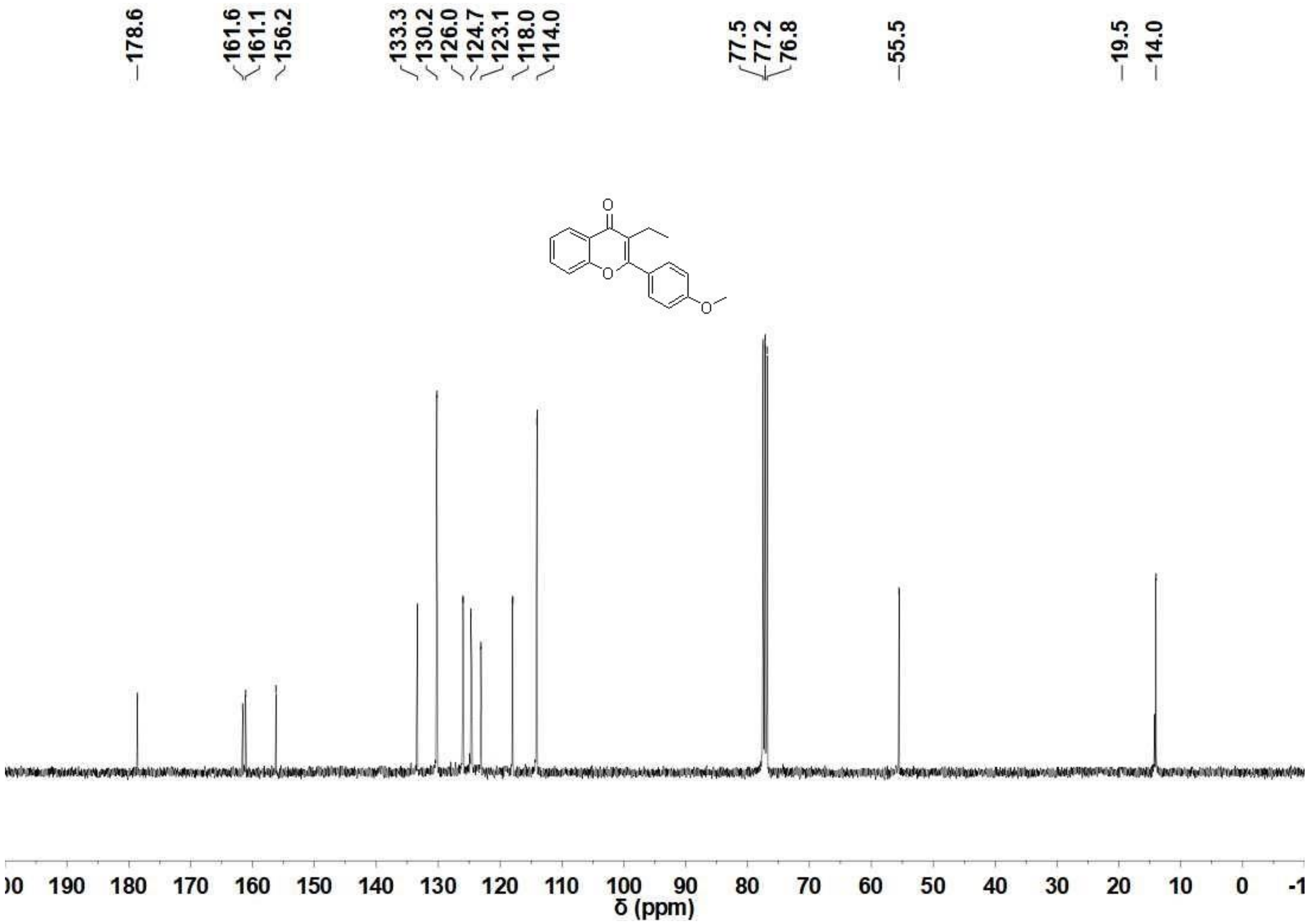
178.6
161.6
161.1
156.2
133.3
130.2
126.0
124.7
123.1
118.0
114.0
77.5
77.2
76.8
55.5
19.5
14.0
δ (ppm)

2-phenyl-*4H*-chromen-4-one-2-^{13}C, [^{13}C]**4aa**

400 MHz, $CDCl_3$

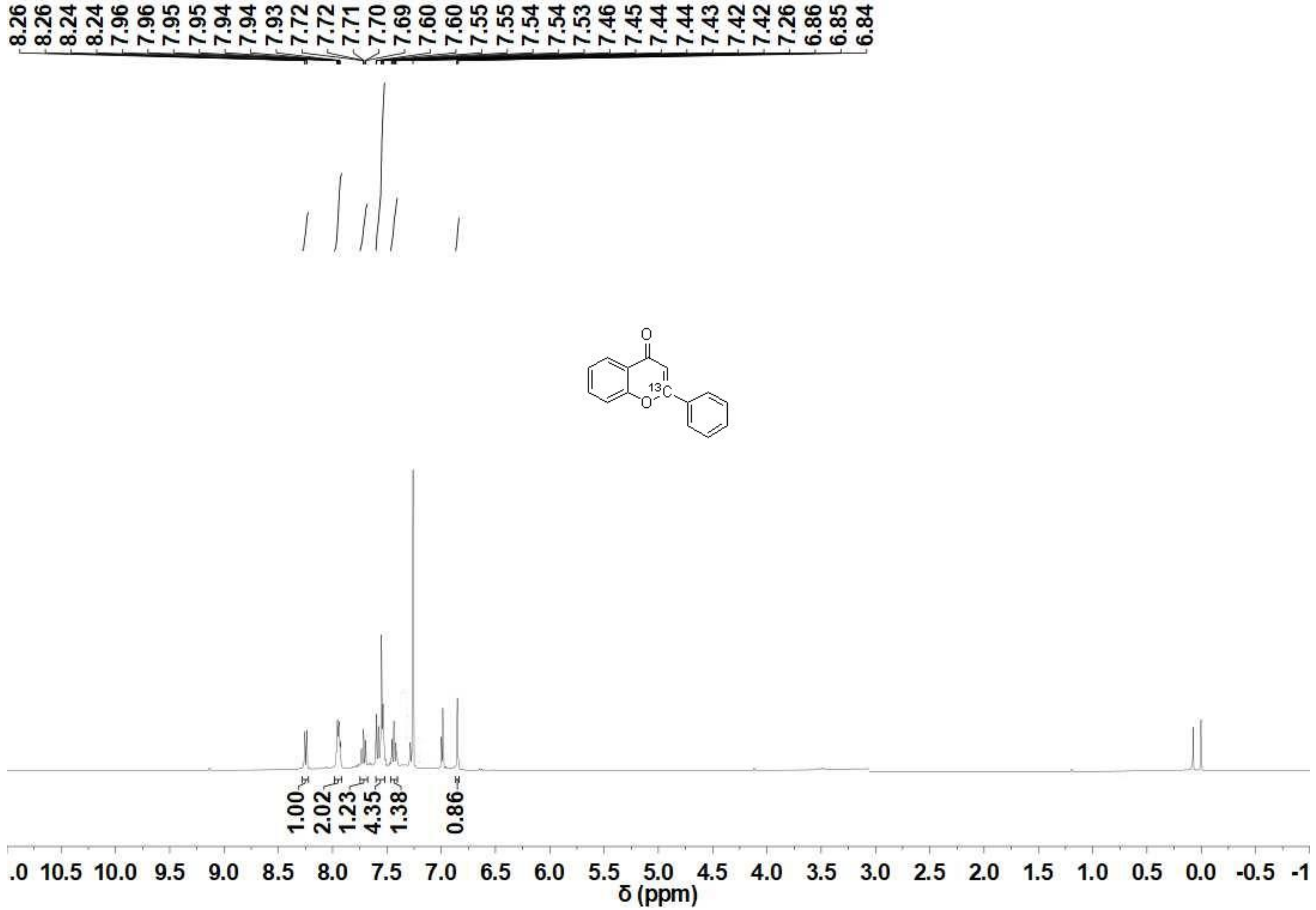

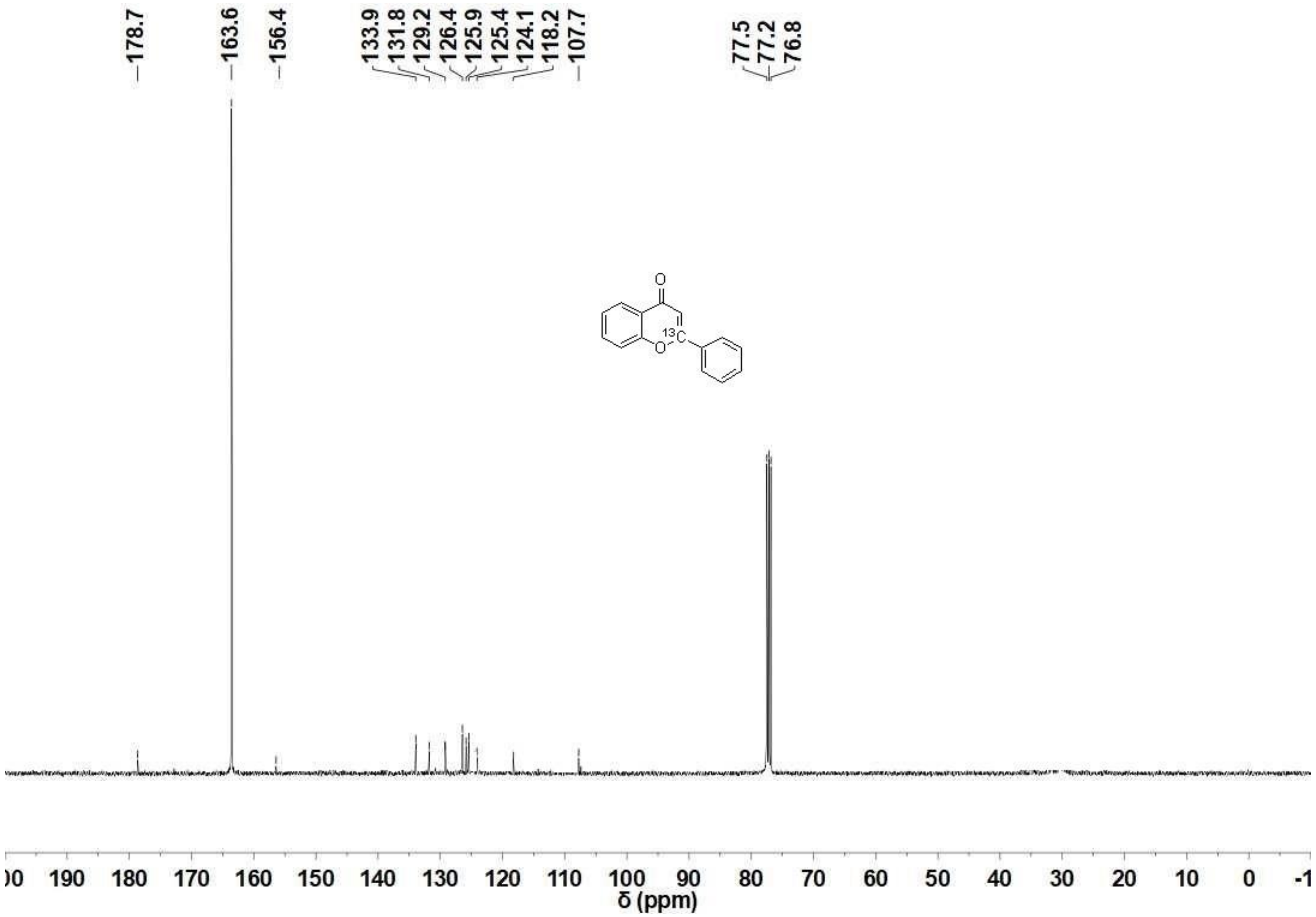
178.7
163.6
156.4
133.9
131.8
129.2
126.4
125.9
125.4
124.1
118.2
107.7
77.5
77.2
76.8
O
13C
O
00
190
180
170
160
150
140
130
120
110
100
90
80
70
60
50
40
30
20
10
0
-1
δ (ppm)

HRMS spectra of [^{13}C]**4aa**

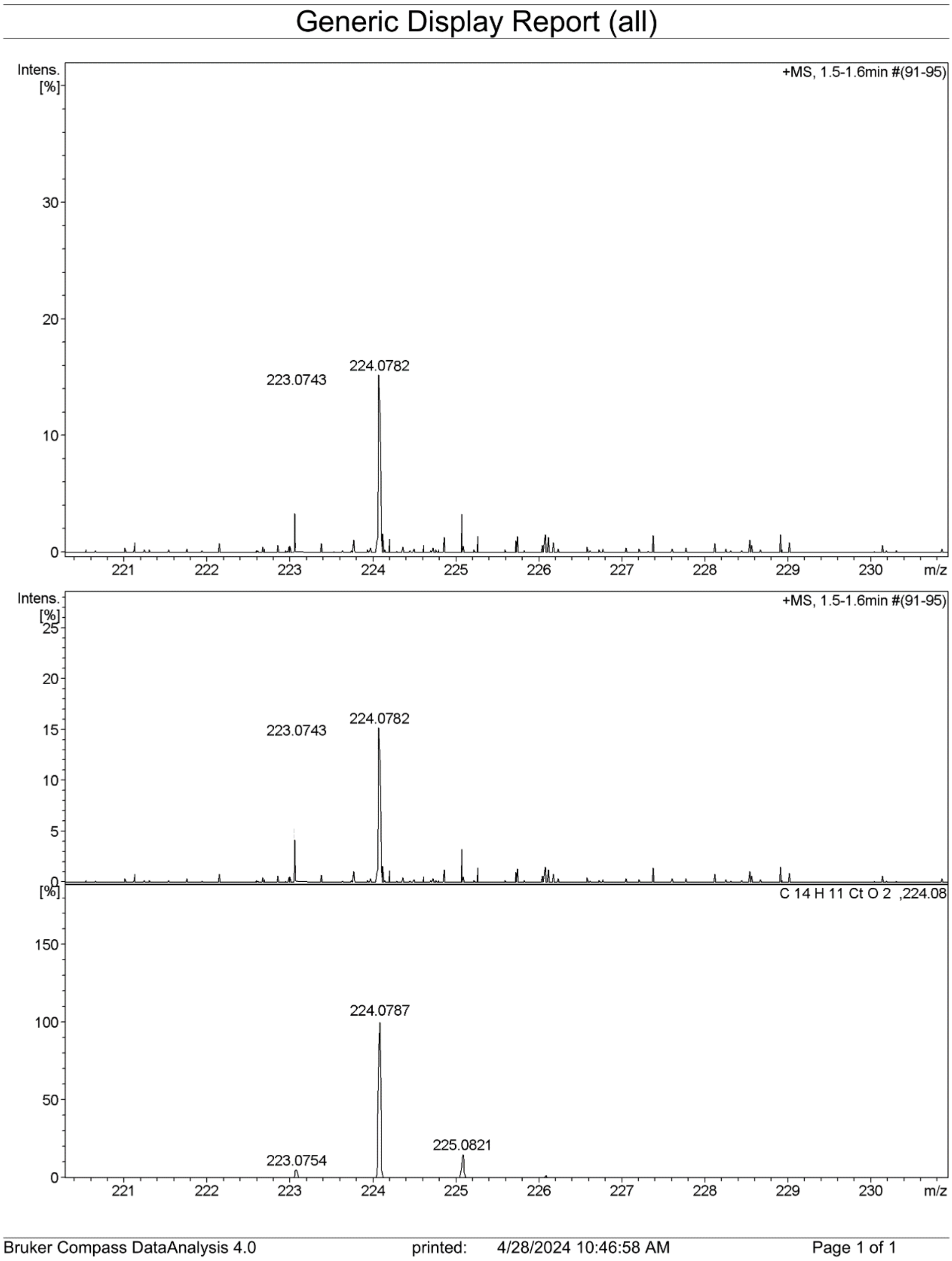

3-methyl-2-(p-tolyl)-*4H*-chromen-4-one-4-^{13}C, [^{13}C]**5ia**
400 MHz, $CDCl_3$

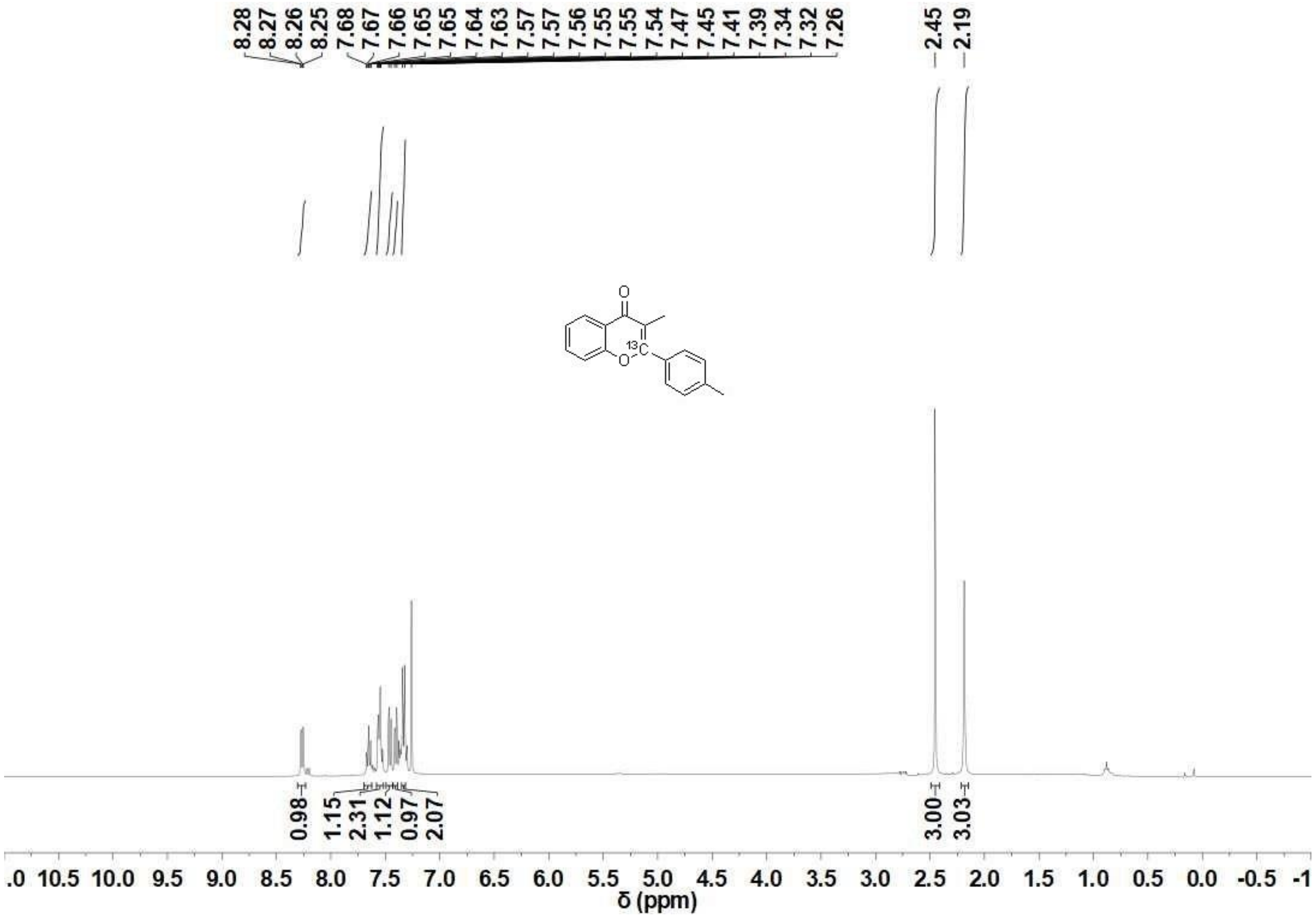

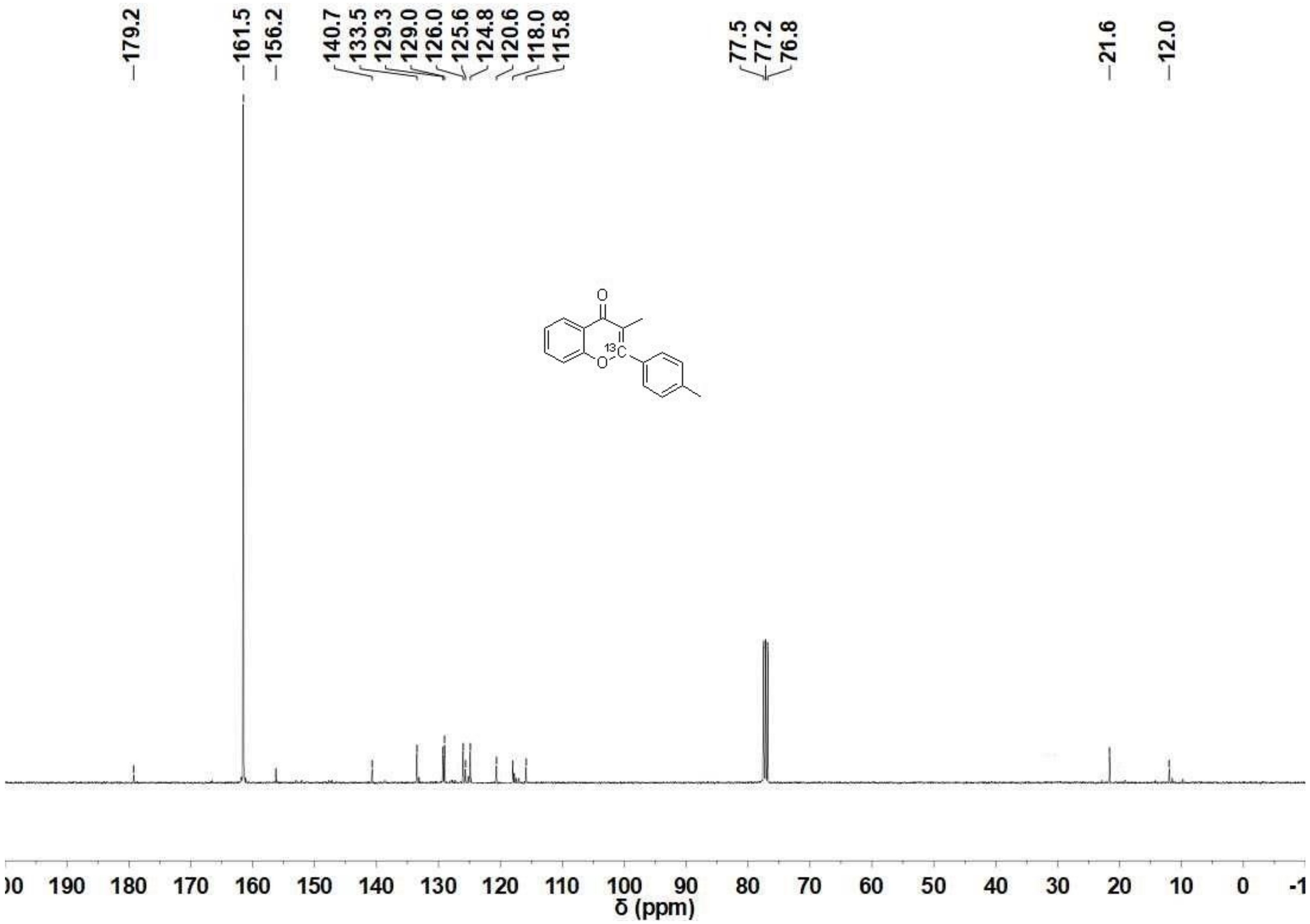
179.2
161.5
156.2
140.7
133.5
129.3
129.0
126.0
125.6
124.8
120.6
118.0
115.8
77.5
77.2
76.8
21.6
12.0
O
13C
O
190
180
170
160
150
140
130
120
110
100
90
80
70
60
50
40
30
20
10
0
δ (ppm)

HRMS spectra of [^{13}C]**5ia**

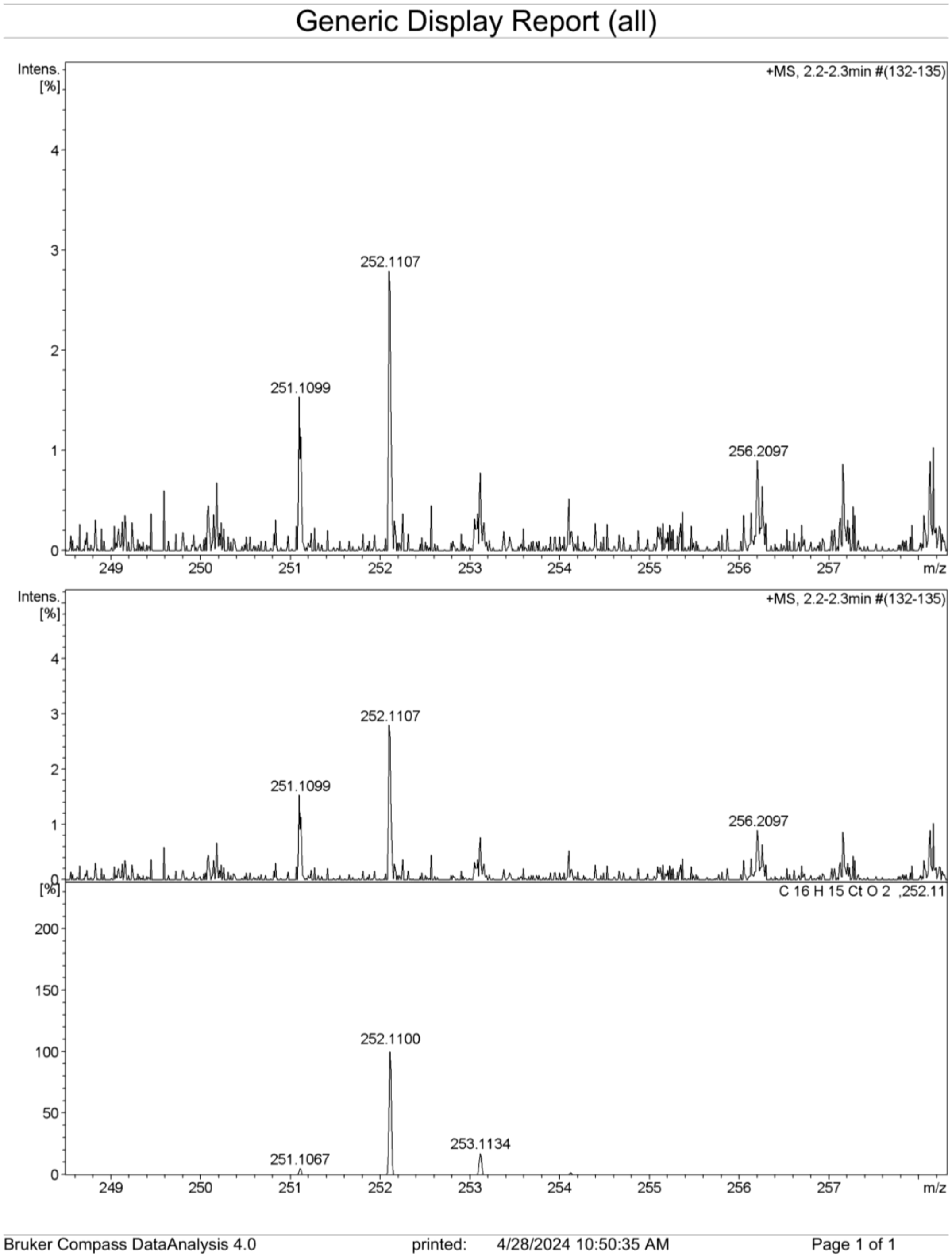

3-ethyl-2-(4-methoxyphenyl)-*4H*-chromen-4-one-2-^{13}C, [^{13}C]**5lb**
400 MHz, $CDCl_3$

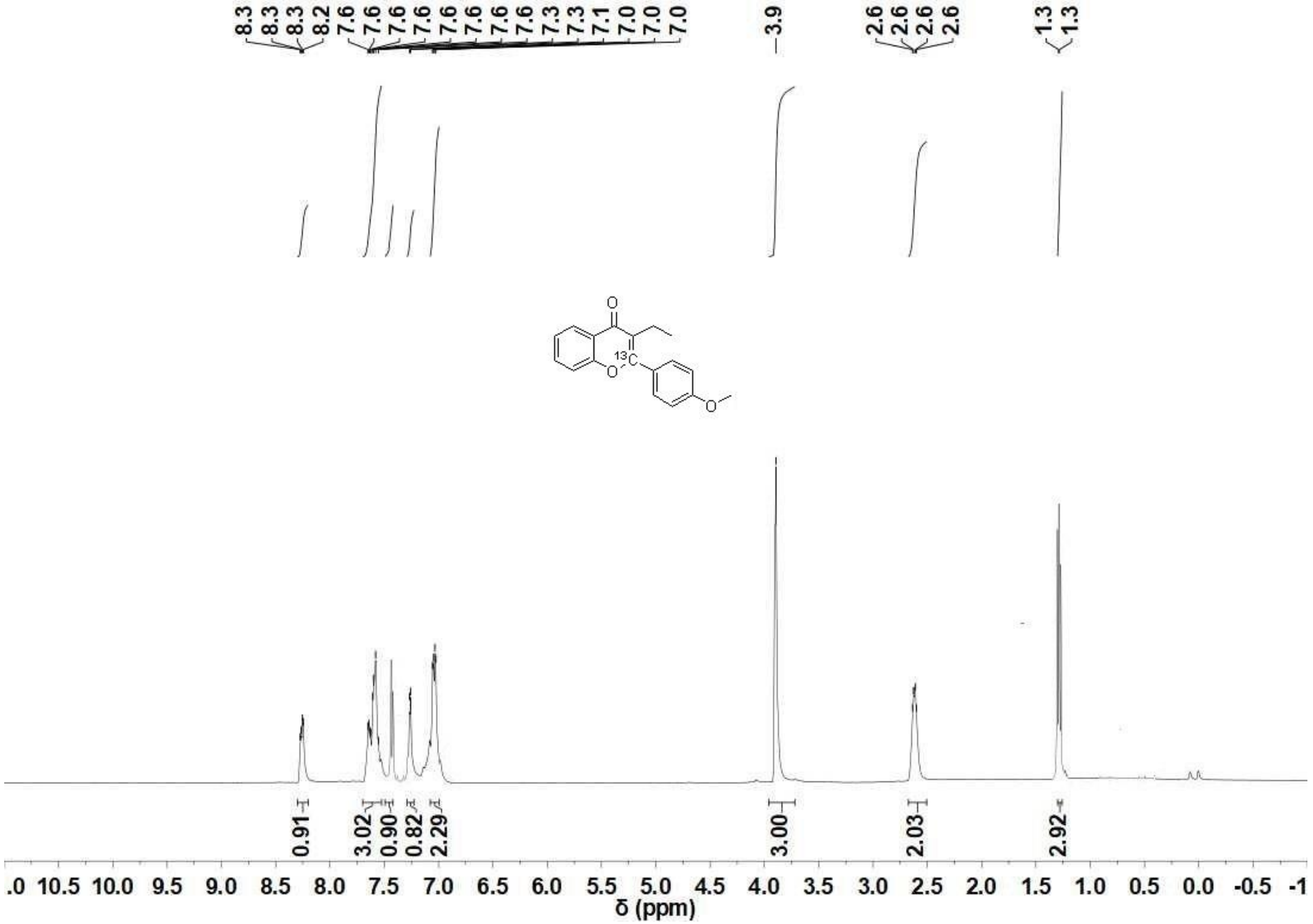

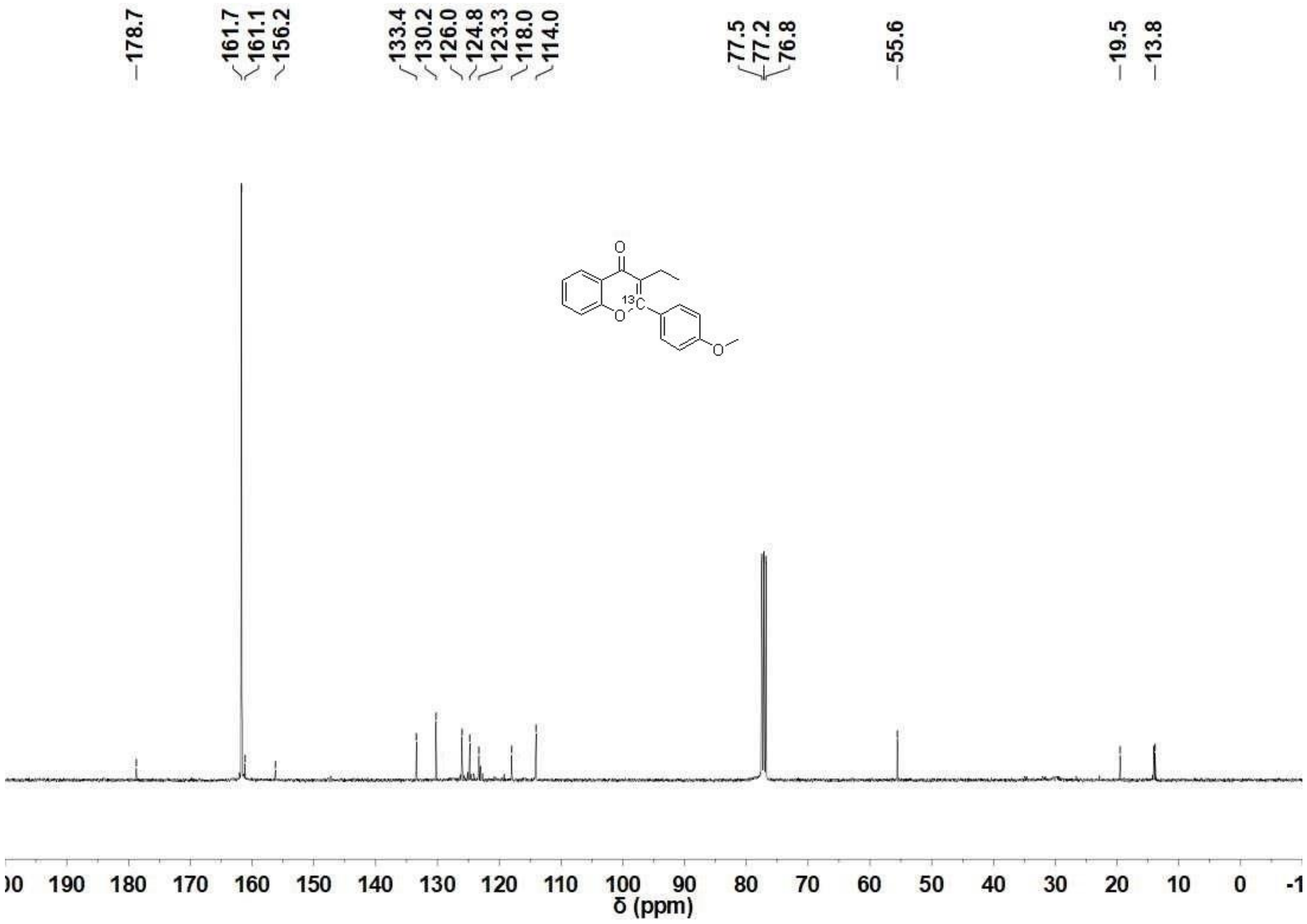
178.7
161.7
161.1
156.2
133.4
130.2
126.0
124.8
123.3
118.0
114.0
77.5
77.2
76.8
55.6
19.5
13.8
O
13C
O
O
190
180
170
160
150
140
130
120
110
100
90
80
70
60
50
40
30
20
10
0
δ (ppm)

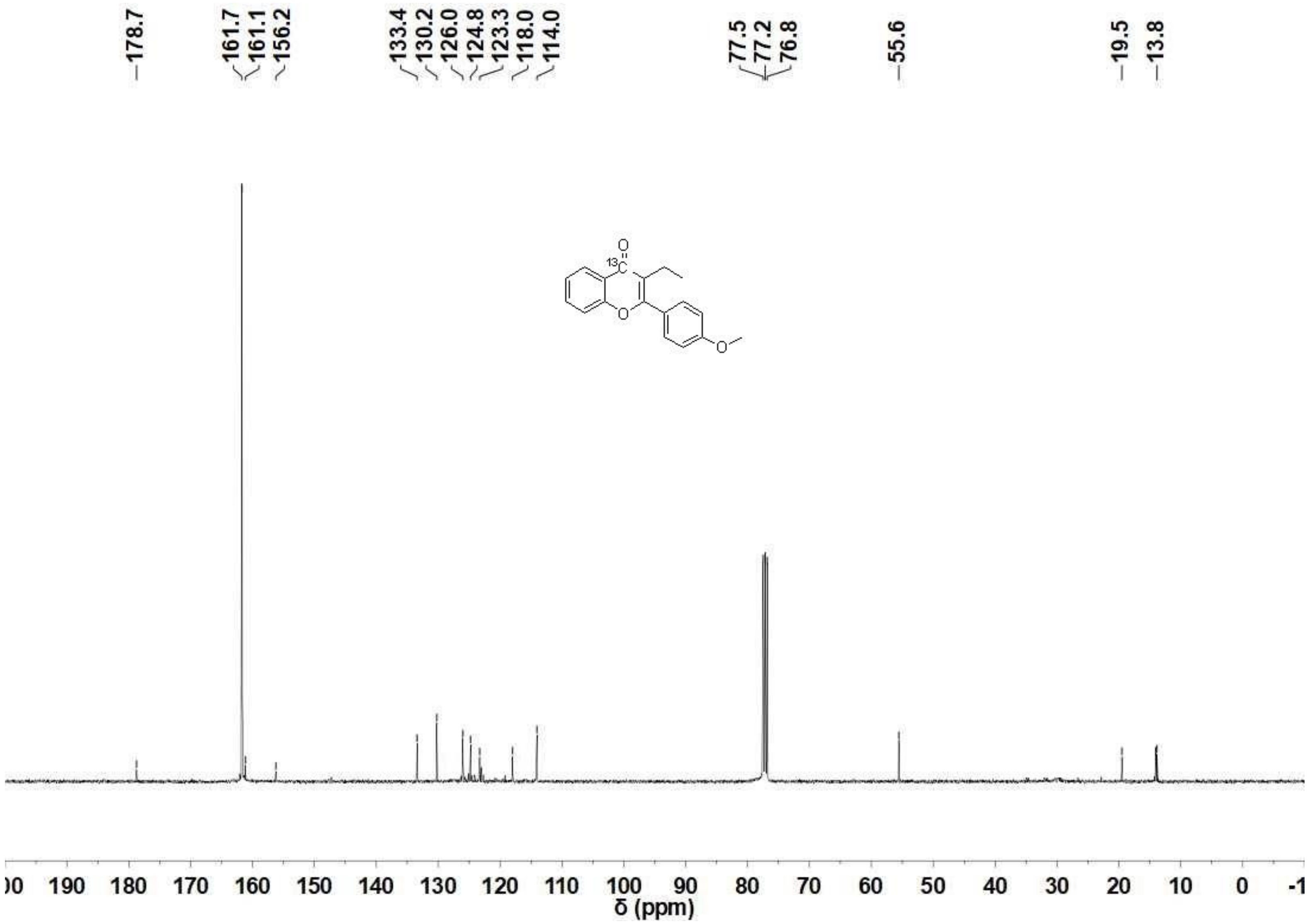
178.7
161.7
161.1
156.2
133.4
130.2
126.0
124.8
123.3
118.0
114.0
77.5
77.2
76.8
55.6
19.5
13.8
O
13C
O
O
δ (ppm)

HRMS spectra of [^{13}C]**5lb**

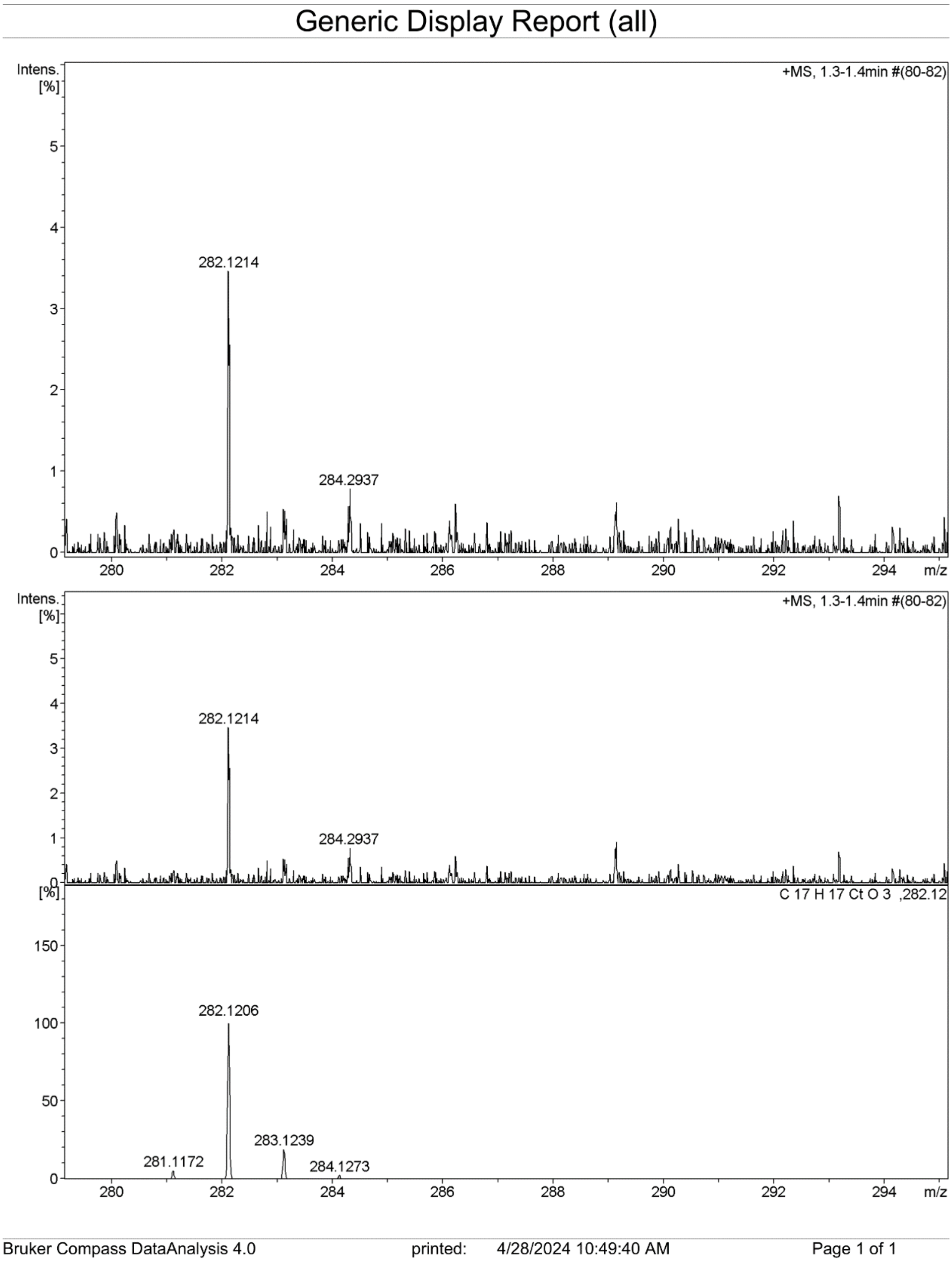

3-hydroxy-2-phenyl-*4H*-chromen-4-one, **1A**

400 MHz, $CDCl_3$

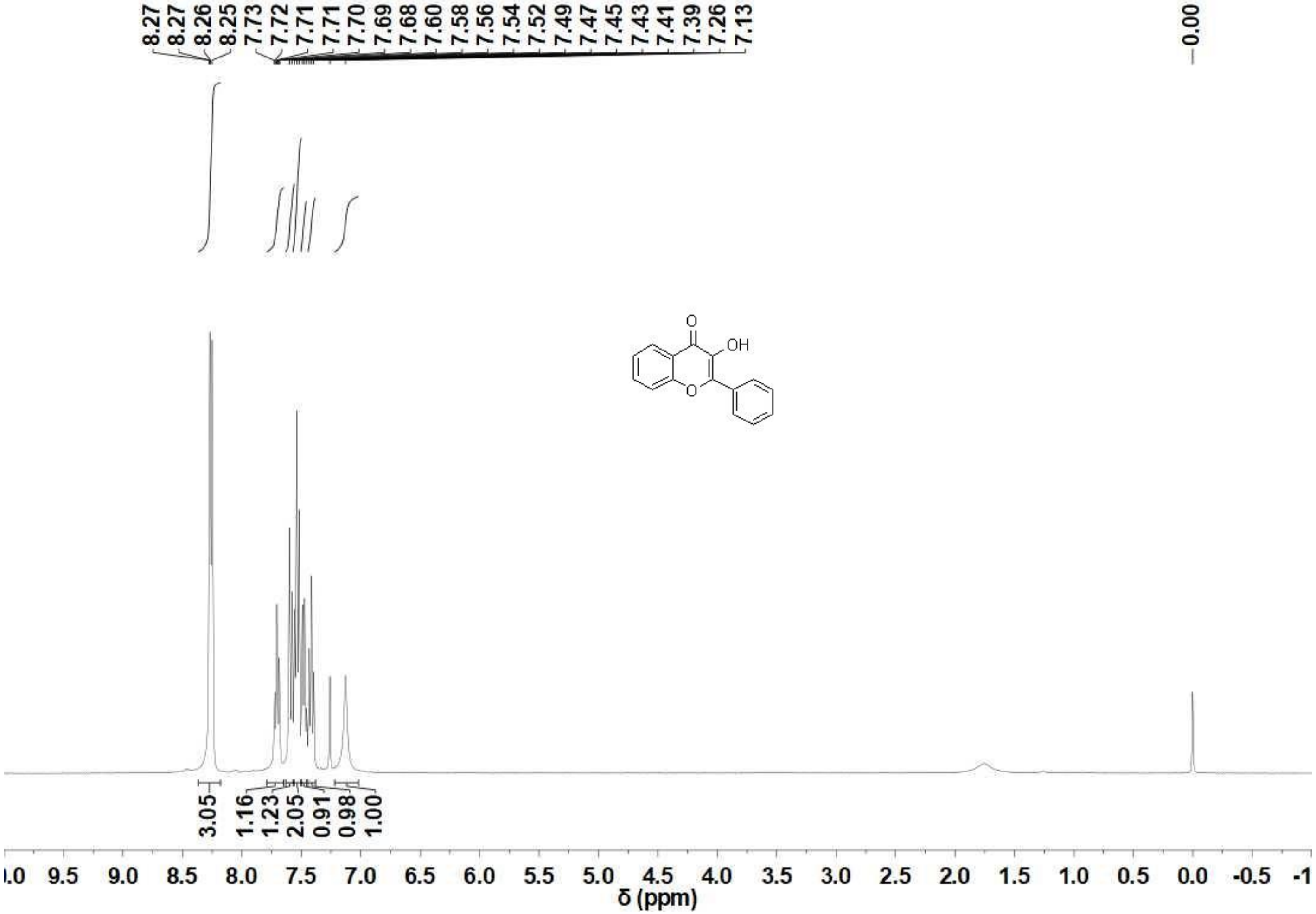

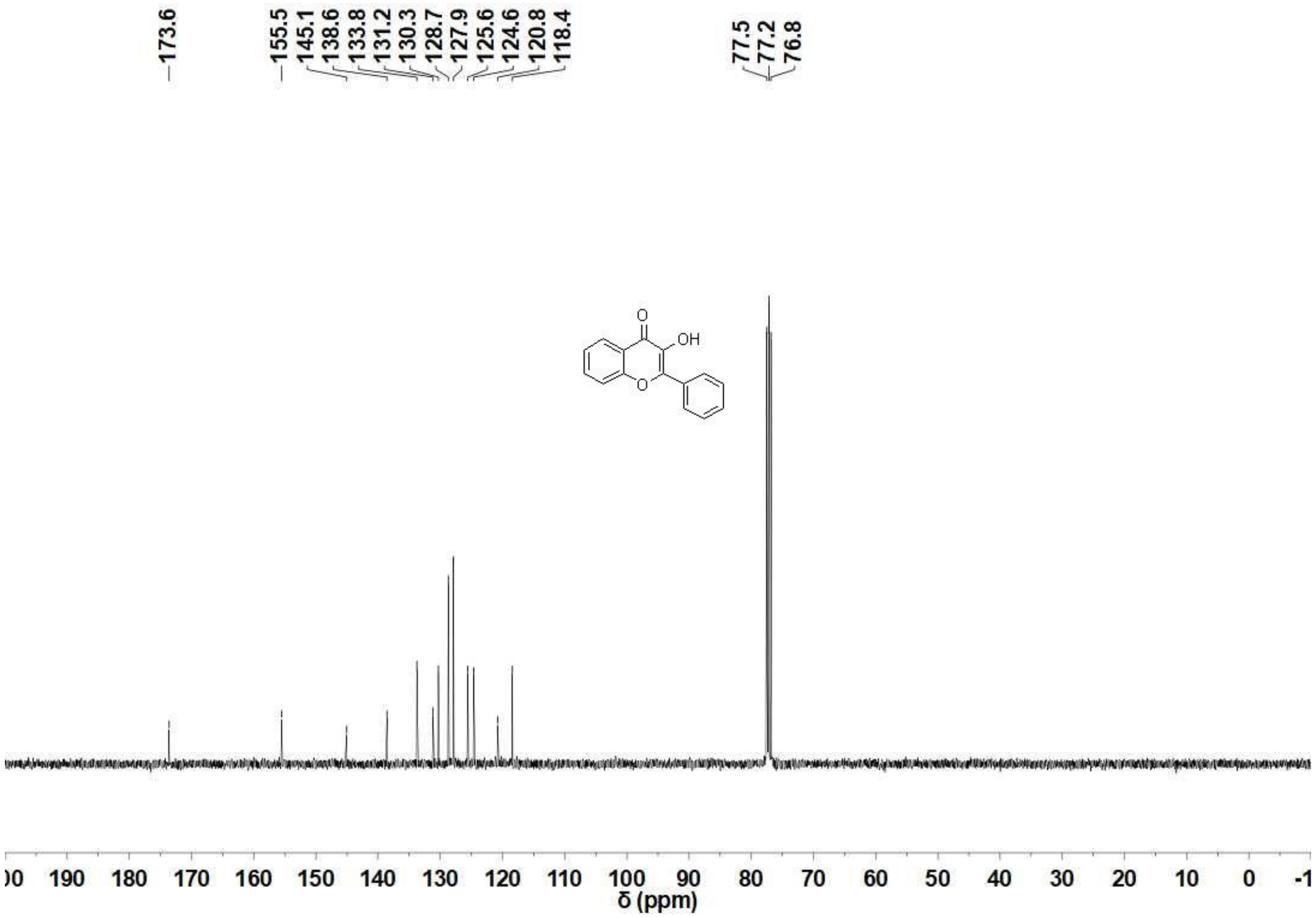
173.6
155.5
145.1
138.6
133.8
131.2
130.3
128.7
127.9
125.6
124.6
120.8
118.4
77.5
77.2
76.8
O
OH
O
190 180 170 160 150 140 130 120 110 100 90 80 70 60 50 40 30 20 10 0
δ (ppm)

2-phenylchroman-4-one, **1B**
400 MHz, $CDCl_3$

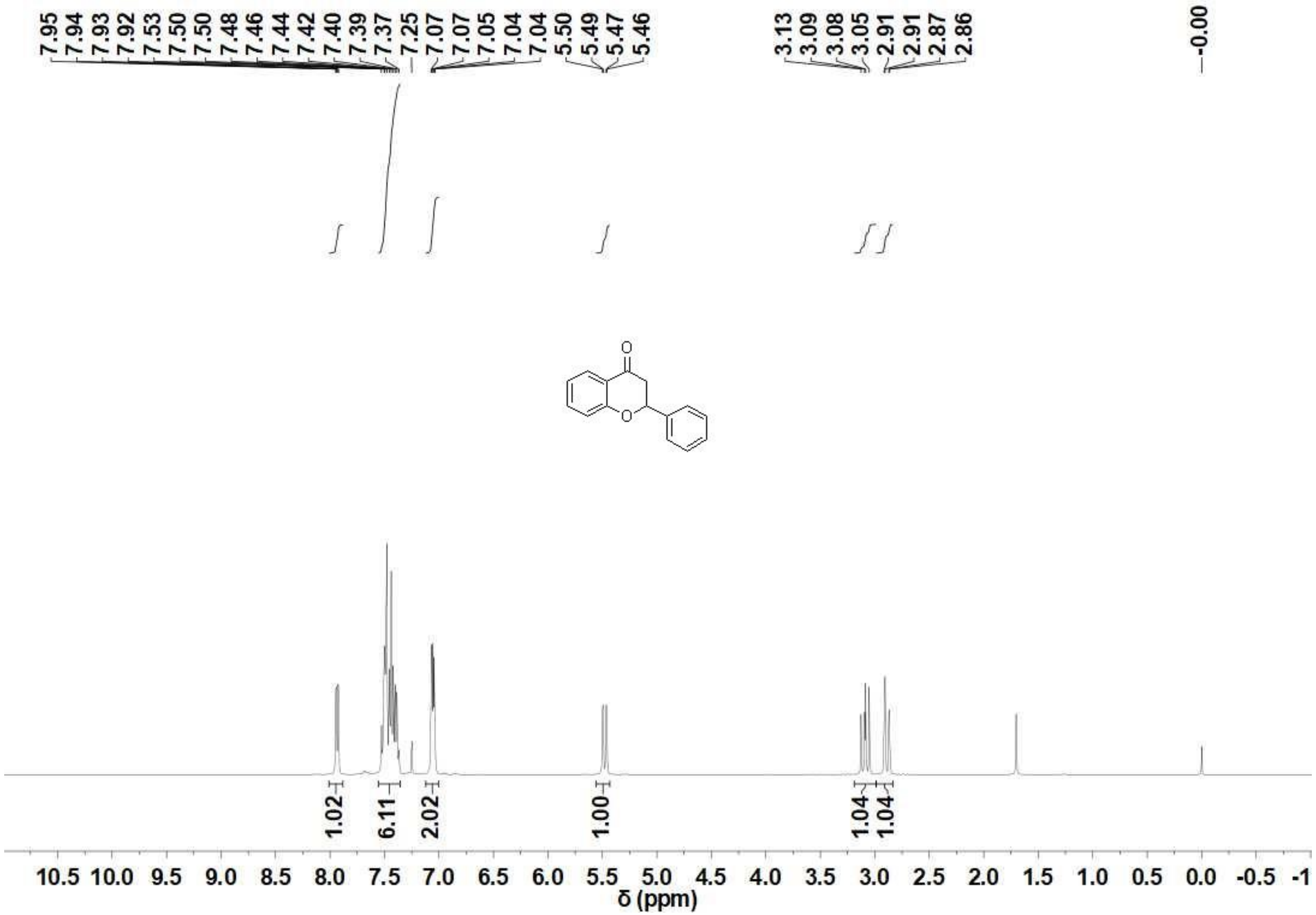

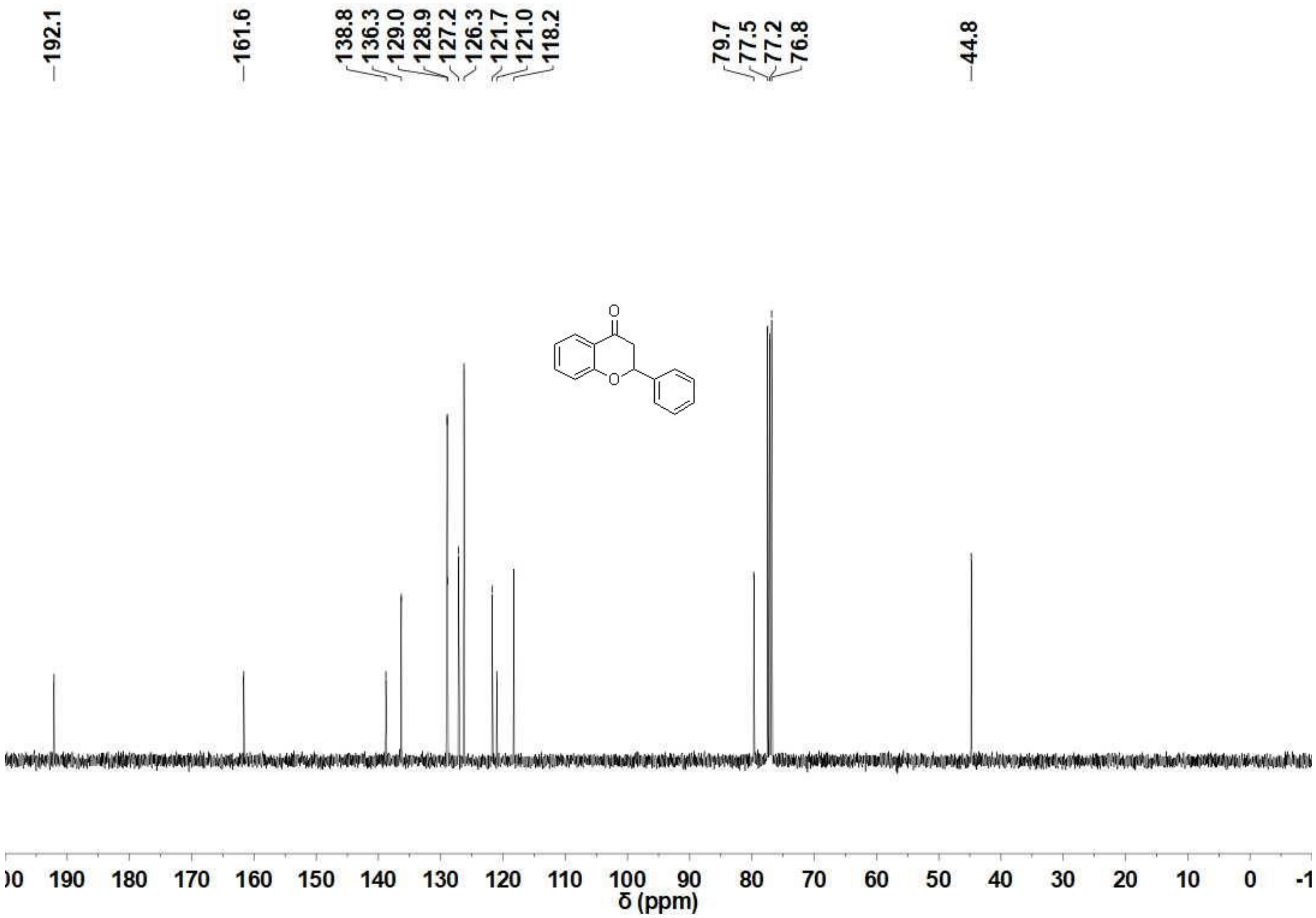
192.1
161.6
138.8
136.3
129.0
128.9
127.2
126.3
121.7
121.0
118.2
79.7
77.5
77.2
76.8
44.8
O
O
190 180 170 160 150 140 130 120 110 100 90 80 70 60 50 40 30 20 10 0
δ (ppm)
